# Olfactory function in the trace amine-associated receptor family (TAARs) evolved twice independently

**DOI:** 10.1038/s41598-021-87236-5

**Published:** 2021-04-08

**Authors:** Milan Dieris, Daniel Kowatschew, Sigrun I. Korsching

**Affiliations:** Institute for Genetics, University At Cologne, Zülpicher Str. 47A, 50674 Cologne, Germany

**Keywords:** Evolution, Genetics, Neuroscience

## Abstract

Olfactory receptor families have arisen independently several times during evolution. The origin of *taar* genes, one of the four major vertebrate olfactory receptor families, is disputed. We performed a phylogenetic analysis making use of 96 recently available genomes, and report that olfactory functionality has arisen twice independently within the TAAR family, once in jawed and once in jawless fish. In lamprey, an ancestral gene expanded to generate a large family of olfactory receptors, while the sister gene in jawed vertebrates did not expand and is not expressed in olfactory sensory neurons. Both clades do not exhibit the defining TAAR motif, and we suggest naming them taar-like receptors (*tarl*). We have identified the evolutionary origin of both *taar* and *tarl* genes in a duplication of the serotonergic receptor 4 that occurred in the most recent common ancestor of vertebrates. We infer two ancestral genes in bony fish (TAAR12, TAAR13) which gave rise to the complete repertoire of mammalian olfactory *taar* genes and to class II of the *taar* repertoire of teleost fish. We follow their evolution in seventy-one bony fish genomes and report a high evolutionary dynamic, with many late gene birth events and both early and late gene death events.

## Introduction

Trace amine-associated receptors (TAARs) were discovered in 2001 as a subgroup of mammalian aminergic receptors^1^. An initial phylogenetic analysis showed the presence of the family in teleost fish^2^ and subsequently their function as olfactory receptors was revealed^3,4^. Another phylogenetic study found the TAAR repertoire sizes in five teleost species to be several-fold larger than those of tetrapods and suggested a subdivision of the TAAR family in three classes^5^. Of these three TAAR classes, only class II is known to serve an olfactory function in both tetrapods and teleosts. Class I is represented by a single, non-olfactory gene in tetrapods and class III is not present in tetrapods^5^.

Class II TAARs have been shown to generate aversive behaviour in rodents^6^ as well as in zebrafish, an early-derived teleost species^7^. However, class II TAARs appeared to be lost in more modern fish (neoteleosts), based on an earlier study in five fish genomes^5^. In the meantime many more genomes have become available and some isolated studies have described the *taar* gene repertoire of individual fish species^8–11^. However no systematic study of taar gene evolution has been performed since and in particular the evolution of class II TAARs is not well understood. Moreover, there exists some controversy about the evolutionary origin of the TAAR family, which variously has been described to originate in jawed vertebrates, vertebrates, and even non-vertebrate chordates^4,5,8^.

To identify the founding gene which gave rise to the TAAR family and to understand the evolution of class II *taar* genes, we have performed a phylogenetic analysis in 96 deuterostome genomes. These species cover a wide evolutionary range, from the sister group of chordates to non-vertebrate chordates to jawless vertebrates to cartilaginous fish to a broad range of bony fish species representing many of the major phylogenetic subdivisions in this most numerous clade of all vertebrates.

We report that the TAAR family originated in vertebrates as a duplication of the much older serotonergic receptor 4. Within the TAAR family olfactory functionality has arisen twice independently, once within jawless fish and once within jawed vertebrates. Class II *taar* genes appear together with class I genes in the ancestor of jawed vertebrates. Bony fish possess two ancestral class II genes, *taar12*, and *taar13*, both with orthologs in the tetrapod lineage. These genes show evolutionary late gene expansions, sometimes at the species level. TAAR12 was found to be absent in neoteleosts, consistent with earlier hypotheses^5^, but TAAR13 was detected in a minority of neoteleost species, suggesting several independent gene death events.

## Results

### Absence of *taar* and *taar*-like genes in non-vertebrate chordates and hemichordates suggests origin of the family in vertebrates

**Figure 1:**
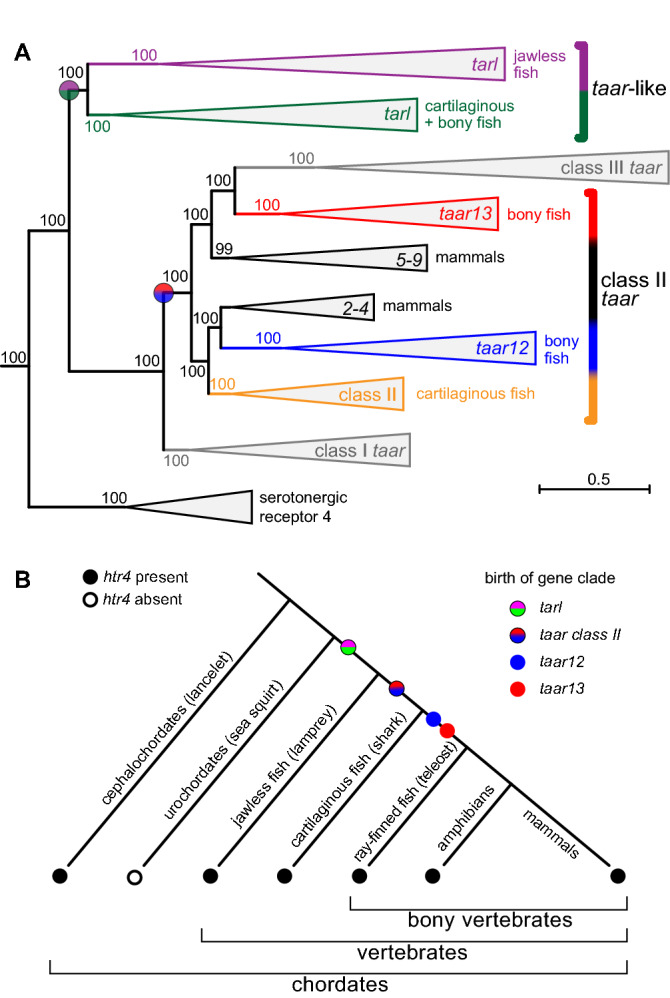
Phylogenetic tree shows the independent origin of jawed fish *taar* and jawless fish *tarl* genes. (**A**) A phylogenetic tree of *taar* and *taar*-like (*tarl*) genes was constructed using MAFFT for alignment and maximum likelihood algoritm PhyML-aLRT. For details see Methods. Clades are collapsed as indicated. A magenta/green bicolour circle denotes the ancestral node of *taar*-like genes (magenta, lamprey; green, cartilaginous and bony fish); a red/blue bicolour circle denotes the ancestral node of class II *taar* genes (red, *taar13* clade; blue, *taar12* clade). Representatives of all major aminergic receptor clades were used as out-group (SI Table 1), only serotonergic receptor 4, the closest relative, is shown. Numbers indicate % branch support. Scale bar, number of amino acid substitutions per site. (**B**) A chordate species tree with predicted gene birth events for *tarl* and *taar* clades. Presence of *htr4* is denoted by filled black circles, its birth is outside the chordate clade.

To investigate the origin of the *taar* gene family we searched in 96 deuterostome genomes. These species cover a wide evolutionary range, from the sister group of chordates (14 species) to non-vertebrate chordates (six species) to jawless vertebrates (two species) to cartilaginous fish (three species) to early-diverging bony fish (one species) and to a broad range of teleost species (70 species) representing many of the major phylogenetic subdivisions. As queries we used zebrafish TAAR13c, a member of teleost class II TAARs, validated TAARs from earlier-derived species, and HTR4. For validation of candidates we performed maximum likelihood phylogenetic analysis, using aminergic receptors as closest possible out-group, and representative zebrafish and mouse *taar* genes as reference group. To reliably identify the gene of origin for the ancestral *taar* gene and to obtain a stable tree topology we found it necessary to include a large number of aminergic receptors in the phylogenetic analysis, representing all major subgroups, cf.^12^ with several genes each. This analysis allowed us to unambiguously identify *taar* genes and to distinguish them from *taar-like* genes and from aminergic receptors with maximal branch support (Fig. 1).

Without exception, serotonergic receptor 4 (*htr4*) emerged as closest relative of known *taar* and *taar-like* genes, for bony vertebrates consistent with and extending earlier results^12^ (Fig. 1). We identified *htr4* in all four lancelets (cephalochordates) analysed, but not in the two ciona species (urochordates) (Fig. 1, SI Fig. 1, SI Table 1). Since urochordates are the closest relatives of vertebrates^13^ this suggests a loss of the *htr4* gene in urochordates. Next, we searched the sister group of chordates^14^ for the presence of *htr4-like* genes, including two hemichordate genomes (acorn worms) and 12 echinoderm genomes (star fish, sea urchins). *htr4* was identified in both species of acorn worms, but not in echinoderms, consistent with a secondary loss of this gene in echinoderms (SI Fig. 1, SI Table 1). Thus, *htr4* was present in the most recent common ancestor (MRCA) of all deuterostomes. Since at the same time no *taar* or *taar-like* genes were found in any of the non-vertebrate chordate, hemichordate and echinoderm species examined (SI Table 1), we conclude that the *taar/tarl* clade originated in the MRCA of vertebrates as a duplication of the much older *htr4* gene, possibly as result of the first whole genome duplication in vertebrates^15^.

**Table 1:** Number of *taar* and *tarl* genes in aquatic vertebrates.

				Class II *taar* genes		
Abbr	Scientific species name	Vernacular name	*tarl*	*taar*12	*taar*13	Other
*Jawless and cartilaginous fish*						
Cm	*Callorhinchus milii*	Elephant shark	1	0	0	3
Le	*Leucoraja erinacea*	Little skate	1	0	0	1
Lec	*Lethenteron camtschaticum*	Arctic lamprey	51	0	0	0
Pm	*Petromyzon marinus*	Marine lamprey	32	0	0	0
Rt	*Rhincodon typus*	Whale shark	2	0	0	4
*Early-derived ray-finned fish*						
Lo	*Lepisosteus oculatus*	Spotted gar	1	3	1	1
*Earlier-derived teleosts (e.g. eels, cyprinids, salmon)*						
Aa	*Anguilla anguilla*	European eel	1	7	5	0
Aj	*Anguilla japonica*	Japanese eel	2	9	9	0
Am	*Astyanax mexicanus*	Mexican cave-fish	1	3	1	0
Ar	*Anguilla rostrata*	American eel	2	13	8	0
Cc	*Cyprinus carpio*	Common carp	2	15	5	0
Ch	*Clupea harengus*	Herring	1	0	1	0
Dr	*Danio rerio*	Zebrafish	1	12	5	0
El	*Esox Lucius*	Northern pike	2	0	1	0
Ip	*Ictalurus punctatus*	Channel catfish	1	2	8	0
Lw	*Leuciscus waleckii*	Amur ide	1	1	2	0
Pp	*Pimephales promelas*	Fathead minnow	1	5	2	0
Pyn	*Pygocentrus nattereri*	Red-bellied piranha	1	5	4	0
Sa	*Sinocyclocheilus anshuiensis*	Anshui sõõrhuul	2	4	5	0
Sf	*Scleropages formosus*	Dragonfish, Asian bonytongue	2	0	4	0
Sg	*Sinocyclocheilus grahami*	Golden-line barbel	1	6	5	0
Sr	*Sinocyclocheilus rhinocerous*	Ninasarv-sõõrhuul	2	7	7	0
Ss	*Salmo salar*	Atlantic salmon	3	0	3	0
*Neoteleosts (e.g. cod, stickleback, fugu, mudskipper), without Ovalentaria*						
Af	*Anoplopoma fimbria*	Sablefish	1	0	2	0
Bp	*Boleophthalmus pectinirostris*	Giant blue-spotted mudskipper	1	0	0	0
Cr	*Cottus rhenanus*	Rheingroppe	1	0	0	0
Cs	*Cynoglossus semilaevis*	Tongue sole	1	0	0	0
Dl	*Dicentrarchus labrax*	European seebass	1	0	2	0
Ga	*Gasterosteus aculeatus*	Three-spined stickleback	1	0	1	0
Gm	*Gadus morhua*	Atlantic cod	2	0	3	0
Hc	*Hippocampus comes*	Tiger tail seahorse	1	0	0	0
Lac	*Lates calcarifer*	Asian sea bass	2	0	2	0
Lb	*Labrus bergylta*	Ballan wrasse	1	0	4	0
Lc	*Larimichthys crocea*	Yellow croaker	1	0	3	0
Mim	*Miichthys miiuy*	Mi-iuy croaker	1	0	0	0
Moa	*Monopterus albus*	Asian swamp eel	1	0	0	0
Mom	*Mola mola*	Ocean sunfish	1	0	0	0
Ms	*Morone saxatilis*	Striped bass	1	0	1	0
Nc	*Notothenia coriiceps*	Black rockcod	0	0	0	0
Pa	*Pampus argenteus*	Silver pomfret	1	0	0	0
Pem	*Periophthalmus magnuspinnatus*	Giant-fin mudskipper	1	0	0	0
Po	*Paralichthys olivaceus*	Bastard halibut	1	0	0	0
Ps	*Periophthalmodon schlosseri*	Giant mudskipper	1	0	0	0
Py	*Pseudopleuronectes yokohamae*	Marbled flounder	1	0	0	0
Sea	*Sebastes aleutianus*	Rougheye rockfish	1	0	3	0
Sem	*Sebastes minor*	Akagaya	1	0	2	0
Ser	*Sebastes rubrivinctus*	Flag rockfish	1	0	2	0
Ses	*Sebastes steindachneri*	Yanaginomai	1	0	1	0
Sh	*Scartelaos histophorus*	Blue mudskipper	1	0	0	0
Sn	*Sebastes nigrocinctus*	Tiger rockfish	1	0	1	0
Tf	*Takifugu flavidus*	Yellowbelly pufferfish	0	0	0	0
Tn	*Tetraodon nigroviridis*	Spotted green pufferfish	1	0	0	0
To	*Thunnus orientalis*	Pacific bluefin tuna	2	0	0	0
Tr	*Takifugu rubripes*	Japanese pufferfish	1	0	0	0
*Ovalentaria (subgroup of neoteleosts, e.g. cichlids, killifish, medaka)*						
Ac	*Amphilophus citrinellus*	Midas cichlid	1	0	0	0
Al	*Austrofundulus limnaeus*	Järvikilli	1	0	0	0
Cn	*Cyprinodon nevadensis*	Amargosa pupfish	1	0	0	0
Cv	*Cyprinodon variegatus*	Sheepshead pupfish	1	0	0	0
Fh	*Fundulus heteroclitus*	Mummichog	1	0	0	0
Hb	*Haplochromis burtoni*	Burton's mouth-breeder	1	0	0	0
Km	*Kryptolebias marmoratus*	Mangrove killifish	1	0	0	0
Mc	*Mchenga conophoros*	Happy	1	0	0	0
Mz	*Maylandia zebra*	Zebra mbuna	1	0	0	0
Nb	*Neolamprologus brichardi*	Lyretail cichlid	1	0	0	0
Nf	*Nothobranchius furzeri*	Turquoise killifish	1	0	0	0
Ol	*Oryzias latipes*	Medaka	1	0	0	0
On	*Oreochromis niloticus*	Nile tilapia	1	0	0	0
Pf	*Poecilia Formosa*	Amazon molly	1	0	0	0
Pl	*Poecilia latipinna*	Sailfin molly	1	0	0	0
Pom	*Poecilia Mexicana*	Shortfin molly	1	0	0	0
Pr	*Poecilia reticulata*	Guppy	1	0	0	0
Pun	*Pundamilia nyererei*	Nyereres Viktoriabuntbarsch	1	0	0	0
Stp	*Stegastes partitus*	Bicolor damselfish	1	0	0	0
Xc	*Xiphophorus couchianus*	Monterrey platyfish	1	0	0	0
Xh	*Xiphophorus hellerii*	Green swordtail	0	0	0	0
Xm	*Xiphophorus maculatus*	Southern platyfish	1	0	0	0

Species are given by scientific and vernacular name, and organized by phylogenetic groups from earlier-diverged to later diverged as indicated. For relative position of groups see Fig. 8.

Known chordate *htr4* genes are multi-exonic, but the splice sites in non-vertebrate chordates (lancelets) are unrelated to those of vertebrates, which are faithfully conserved in mammals, bony fish, cartilaginous fish and jawless fish (SI Table 2), suggesting that these intron gains have occurred in the MRCA of vertebrates. The two acorn worm *htr4* genes are mono-exonic (SI Table 2), suggesting as the most parsimonious explanation that the *htr4* gene was still mono-exonic at the time of birth of the *taar/taar-like* clade. Another possibility would be birth by retroposition of an already multi-intronic vertebrate *htr4* gene, which however does not change the time of origin of the *taar/taar-like* clade in the MRCA of vertebrates.

### Olfactory functionality appears to have arisen twice independently in the *taar/taar*-like family

There has been some controversy concerning the time of birth of the TAAR family, which some authors placed at the origin of vertebrates, e.g.^4^, while others argued for a later origin within the MRCA of cartilaginous and bony fish^5^. Even an origin in non-vertebrate chordates has been suggested^8^. Those studies generally were done with a very limited number of different species, and in particular there was a considerable dearth of early-derived species, as most of those genomes have become available only recently. Here we have performed a phylogenetic analysis of all TAAR-related genes using hemichordate, non-vertebrate chordate, jawless, cartilaginous and bony fish genomes, together with a representative collection of aminergic receptors including those from early-derived species. The resulting phylogenetic tree shows maximal branch support in all basal nodes (Fig. 1). The tree topology suggests a birth of the *taar/taar-like* clade as a duplication of the *htr4* gene in the MRCA of vertebrates, as discussed above. Another duplication of this ancestral gene gave rise to a monophyletic clade containing all unequivocally designated *taar* genes and another clade containing genes whose assignment is not settled. Several genes in the latter clade have previously been described as TAAR^8^, TAAR-V^4^ or TAAR*^12^. Here we are referring to these genes as *taar-like* (trace-amine associated receptor-like, or *tarl*) to indicate both the relatedness to *taar* genes as well as their difference to them.

**Figure 2:**
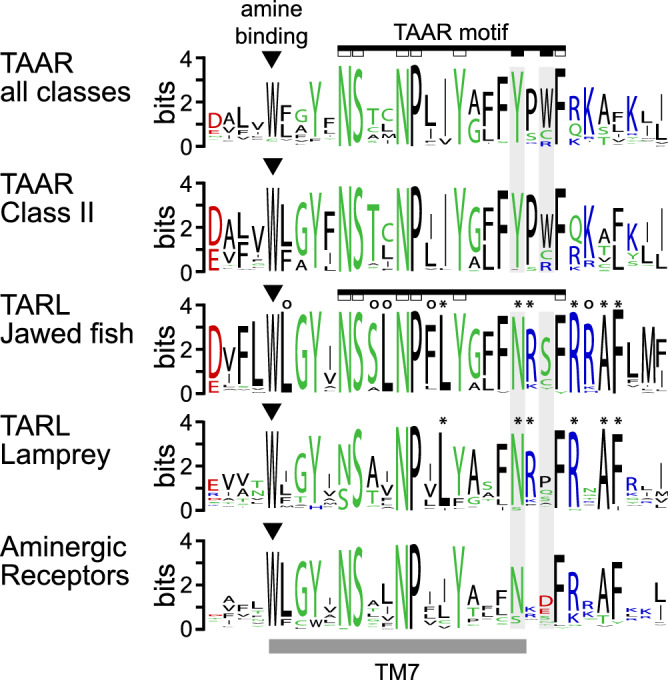
Absence of the TAAR-specific fingerprint motif in *taar*-like genes. The characteristic fingerprint motif (black bar) of *taar* genes^16^ and the respective homologous sequence regions in *taar-*like genes (TARL) and aminergic receptors (AmR) are vertically aligned. Grey bar, transmembrane region 7. Note that the positions of the motif denoted by empty rectangles are highly conserved in all gene groups shown. In stark contrast, the two TAAR-specific positions of the motif (Y and W, filled rectangles) are differently conserved-N instead of Y (*tarl* genes from jawed and jawless fish, and aminergic receptors)-S instead of W (*tarl* genes from jawed fish)—or not conserved in non-*taar* genes. Asterisks, additional amino acids are conserved in all *tarl *genes; empty circles, amino acids only conserved in *tarl* genes from jawed fish.

A careful phylogenetic study, which delineated and renamed the TAAR family of several mammalian species has described the presence of a characteristic fingerprint motif in taar genes as 100% sensitive and specific^16^. This motif was found to be strongly conserved in teleost fish *taar* genes as well^5^, and in fact in all jawed fish TAARs we examined here (Fig. 2). The corresponding sequence in the clade previously designated as TAAR-V or TAAR*^4,12^ is highly conserved as well, but lacks the critical residues of the TAAR motif and in fact shows a clearly different motif (Fig. 2), supporting the placement of these genes as a separate *tarl* clade. The differentially conserved amino acids are located in the C-terminal region, just adjacent to TM7. This region generally is relevant for binding interactions to downstream signalling molecules^17^ and it may be expected that TAAR and TARL differ in that respect.

Lamprey possess only the *tarl* clade, not the *taar* clade, whereas cartilaginous and bony fish possess both clades (Fig. 1). This would be consistent with an origin of the ancestral *taar* gene as a duplication of the older *tarl* gene in the 2^nd^ vertebrate whole genome duplication, which happened in jawed vertebrates, after the divergence from jawless vertebrates^15^, although an earlier birth in the MRCA of all vertebrates and subsequent loss in jawless vertebrates cannot be excluded currently.

**Figure 3:**
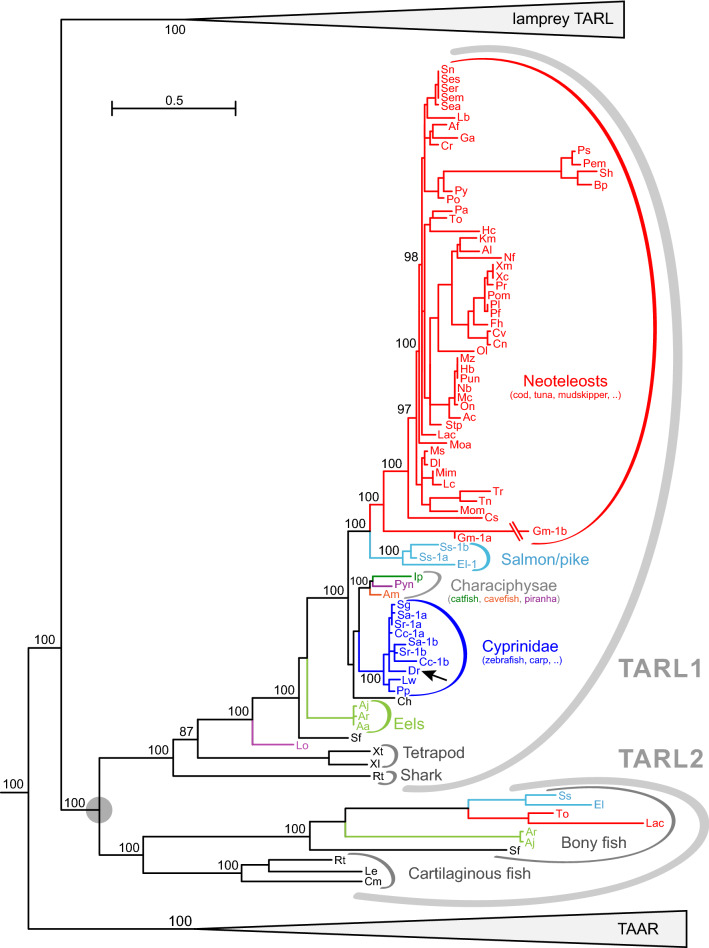
Phylogenetic tree of cartilaginous and bony fish *taar*-like genes. Phylogenetic tree of *taar* and *taar*-like genes, with all nodes collapsed (grey triangles) except bony and cartilaginous fish *taar*-like genes (ancestral node denoted by grey circle). Gene set and tree construction are same as in Fig. 1. Species are indicated by the initials of their Latin names, see Table 1 for full names, gene names as indicated. Note a basal duplication of the ancestral *tarl* gene into tarl1 and tarl2 clades. Late gene duplications are denoted by letters, e.g. Cc-1b stands for *tarl1b* of crucian carp. Note that this gene tree closely follows the corresponding species tree, with cartilaginous fish TARL occupying basal nodes, and TARL from earlier-derived bony fish (spotted gar, eel) basal in the bony fish TARL clade. Black arrow, zebrafish TARL1, whose expression is shown in Fig. 4. Without exception, neoteleost TARL are situated in the most-derived sub-clade (red). Numbers indicate % branch support for basal nodes (cutoff 80%). Scale bar, number of amino acid substitutions per site.

**Figure 4:**
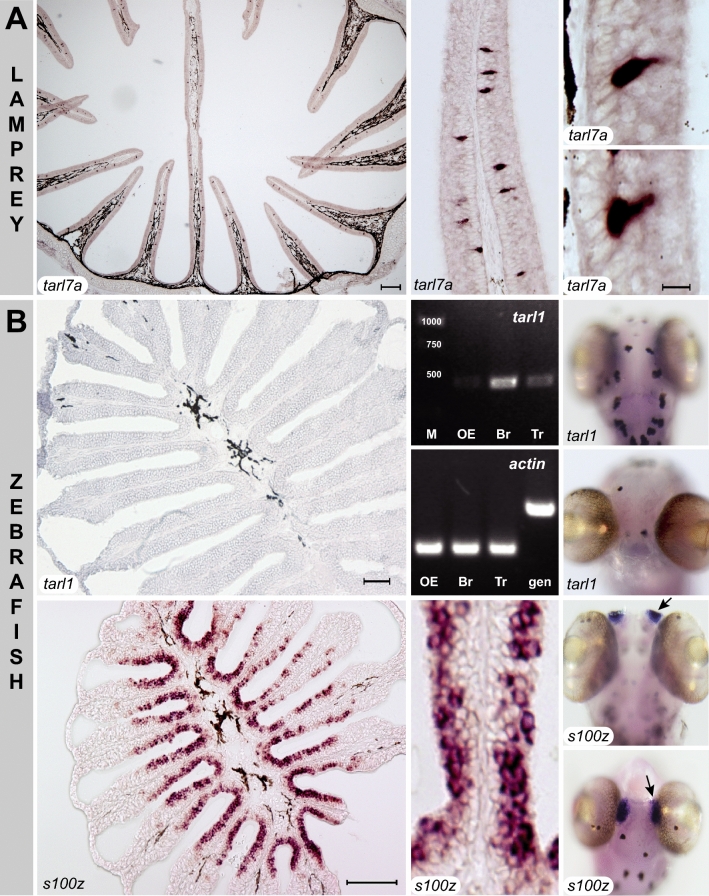
Unlike lamprey *tarl,* zebrafish *tarl* is not expressed in the nose. (**A**) Lamprey (*Lampetra fluviatilis*) *tarl7a* probe was used to perform chromogenic in situ hybridization of horizontal cryostat sections of olfactory epithelia. Scale bars: left panel 200 μm, middle panel 50 μm and right panels 10 μm. Note expression in sparse cells within the olfactory lamellae, characteristic for olfactory receptors. (**B**) Middle column, top panel, RT-PCR for Dr-*tarl1* with primer pair 1 (see Materials and Methods) shows extremely faint signal in the OE, a strong signal in the brain (Br) and moderate expression in the trunk (Tr). Dr-*tarl1* primer pair 2 gave same results (data not shown); M, marker, numbers refer to bp. Middle panel, beta-actin was used as control, and shows equal loading in all tissues as well as absence of genomic DNA in the cDNA preparation (gen, genomic DNA). Both in situ hybridization of horizontal cryostat sections of olfactory epithelia (left panel, scale bar 50 μm) and whole mount in situ hybridization of 5 dpf zebrafish larvae (top right panel, dorsal view; bottom panel, frontal view) show absence of tarl1 expression in the nose. In situ hybridization results shown are for probe 1 corresponding to primer pair 1 (see Materials and Methods). All but one panel have been modified from the PhD thesis of one author^43^. Bottom row, as a positive control, *S100z* expression is shown in the adult nose (left and middle panel, scale bars 100 and 20 μm, respectively) and in 5 dpf larvae, same orientation as for *tarl1* expression. The nose (black arrows) is visible as two anterior bluish spots (top panel, dorsal view) and medially adjacent to the eyes (bottom panel, frontal view).

Some genes in the lamprey clade have been shown to be expressed in the olfactory epithelium in the characteristic sparse pattern associated with olfactory receptor genes^18^. We therefore wished to see, whether bony fish *tarl* (Fig. 3) would be expressed in a similar fashion. To this end, we examined expression of the single *tarl* gene in zebrafish (*tarl1*) in different tissues by RT-PCR, using two independent sets of primers (Fig. 4 and data not shown). *Tarl1* was detected in all tissues examined, including a very weak signal in olfactory epithelium, but with the strongest expression in brain (Fig. 4). Theoretically a very weak signal in the nose could result from very rare olfactory sensory neurons expressing *tarl1*, but in situ hybridization of adult olfactory epithelium as well as larval whole mounts could not visualize any labelled cells in the zebrafish nose, whereas another gene serving as positive control (*S100z*) resulted in robust staining of olfactory sensory neurons (Fig. 4). This is in clear contrast to the strong and specific expression of a lamprey *tarl* gene (*Lf-tarl7a*) in the olfactory lamellae (Fig. 4). Thus the weak RT-PCR signal is more likely to result from broad expression at very low transcript levels, which would not be visible in the less sensitive in situ hybridization. Thus, in contrast to lamprey TARLs zebrafish TARL1 does not appear to function as olfactory receptor. The comparatively strong expression of *tarl1* in brain may suggest a role as a trace amine receptor.

In contrast, many publications have shown the olfactory nature of the *taar* genes proper, with one interesting exception. In all species examined so far, the most basal *taar* gene (TAAR1), is not expressed in the nose^3,5^. Thus the most parsimonious explanation for the origin of olfactory function in the *taar/tarl* clade implies two independent recruitment events, one within the *taar* clade after the first gene duplication, and one within the *tarl* clade after the divergence of jawed and jawless vertebrates. Alternatively one would have to postulate that the duplicated *htr4* gene already acquired olfactory functionality, but did not expand in the typical manner of olfactory receptor families until two independent losses of olfactory functionality occurred, one in the ancestral *taar* gene, and one in the ancestral *tarl* gene of jawed vertebrates, a somewhat unlikely scenario. Thus it appears most likely that olfactory functionality arose twice independently, once within jawed fish and once within lamprey, respectively.

### Lamprey *taar*-like genes show frequent gene gains characteristic for olfactory receptor repertoires, with a family size rivalling that of *taar* genes in bony fish

**Figure 5:**
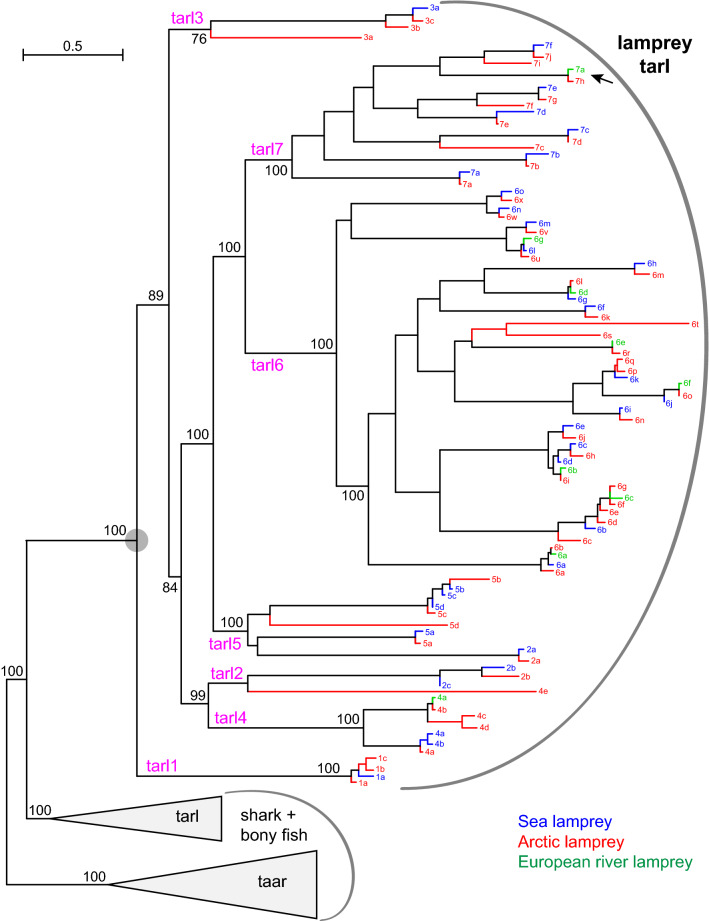
Phylogenetic tree of lamprey *taar*-like genes. Phylogenetic tree of *taar* and *taar*-like genes, with all nodes collapsed (grey triangles) except lamprey *taar*-like genes (ancestral node denoted by grey circle). Gene set and tree construction are same as in Fig. 1. European river lamprey, *Lampetra fluviatalis*; Arctic lamprey, *Lethenteron camtschaticum*; Sea lamprey, *Petromyzon marinus*. Genes are indicated by colour code for the species as shown and the unique part of the gene name, e.g. 1a (red) denotes *tarl1a* of the arctic lamprey. Magenta, predicted ancestral genes. Note close arctic lamprey orthologs for all *tarl* genes from European river and sea lamprey. Black arrow, European river lamprey *tarl7a*, whose expression is shown in Fig. 4. Numbers indicate % branch support for basal nodes (cutoff 80%). Scale bar, number of amino acid substitutions per site.

We analysed two lamprey genomes (*Petromyzon marinus*, sea lamprey and *Lethentheron camtschaticum*, arctic lamprey). We identified 32 sea lamprey *tarl* genes (Table 1), four more than previously reported^8^, possibly due to improvements in the genomic sequence. For arctic lamprey we report 51 genes, 59% more than in the sea lamprey (Table 1). Nine European river lamprey *tarl* genes (partial sequences^18^) were included in the phylogenetic analysis. All lamprey *tarl* genes belong to a monophyletic clade, with maximal branch support for the clade and for the segregation from jawed fish *tarl* and *taar* genes (Figs. 1, 5)*.*

The European river lamprey *tarl* sequences are without exception very close orthologs of the corresponding arctic lamprey genes, with 94–99% identity (mean value 98%) at the amino acid level (Fig. 5, SI File 2). Often, but not always, close orthologs to arctic lamprey are also found in sea lamprey *tarl* (Fig. 5), with 86–97% identity (mean value 94%). Divergence times are 13 million years between European river lamprey and arctic lamprey and 16 million years between arctic lamprey and sea lamprey^19^, suggesting a somewhat slower evolution than that of bony fish TAARs, *cf*. identity between fugu and tetraodon orthologs in the range of 69–83% (mean value 74%)^5^, with an evolutionary distance of 42 million years^19^. In several cases we observe species-specific gene duplications, both in arctic and in sea lamprey. Additionally there are several arctic lamprey tarl genes without a sea lamprey ortholog (and a single vice versa case). The phylogenetic position of these ortholog-less genes is consistent with gene death events in the respective other lamprey species.

The large gene expansion from a single ancestral gene, the frequent gene death events, as well as the species-specificity of several *tarl* genes are reminescent of the properties of the four main vertebrate olfactory receptor families (OR, TAAR, V1R, V2R)^21^. This is consistent with the expression in sparse olfactory epithelial cells reported for three of these genes (river lamprey^18^) and confirmed for one of them by us (Fig. 4). These patterns are reminiscent of those observed for OR receptors in lamprey^8,20^ and TAAR receptors in bony fish^5^.

### Slow evolution of *taar*-like genes in jawed vertebrates

In stark contrast, the sister clade of lamprey *tarl* genes in bony fish shows a single duplication event, which occurred early in the evolution of jawed fish (Fig. 3). Since the chromosomal location of TARL1 and TARL2 is different (SI Table 1), the most likely explanation is that it originates from the whole genome duplication observed in the MRCA of jawed vertebrates^15^. Most fish species analysed have retained at least one *tarl* gene (77 species), but only 9% (7 species) have retained both copies (Fig. 3). There is no correlation of retention of both genes with the salinity of the respective species' ecological environment, i.e. both some fresh water and some sea water species have retained both copies. Furthermore, *tarl* genes from jawed vertebrates show much smaller species differences than either lamprey *tarl* or jawed vertebrate *taar* genes. Additionally, as discussed above, zebrafish *tarl1* is only present in very low levels in the nose, and in situ hybridization did not visualize the characteristic expression pattern of olfactory receptors^27^ (Fig. 4). Taken together these results do not support a role of bony fish TARL as olfactory receptors.

### Two ancestral *taar* gene duplications in the common ancestor of tetrapods and teleosts

In contrast, the olfactory function of *taar* genes has been demonstrated in many species^3,6,22^ and frequent gene gains within the TAAR family have been shown in particular for two bony fish species, zebrafish and stickleback^2,4,5^, with over one hundred different *taar* genes in zebrafish^5^. In mammals TAARs represent a rather small group of olfactory receptors with 6 genes in humans and 15 in mice^2^. Here we have examined the early events in the evolutionary history of the TAAR family by a thorough search in 74 jawed vertebrate genomes including cartilaginous fish and early-derived bony fish using mouse as tetrapod reference genome. The first duplication of the original *taar* gene (sister gene to the original *tarl* gene) appears to have occurred before the divergence of cartilaginous and bony fish, resulting in class I and class II *taar* clades, respectively (Fig. 1). This result is consistent with earlier phylogenetic analyses using a limited set of genomes^5^.

**Figure 6:**
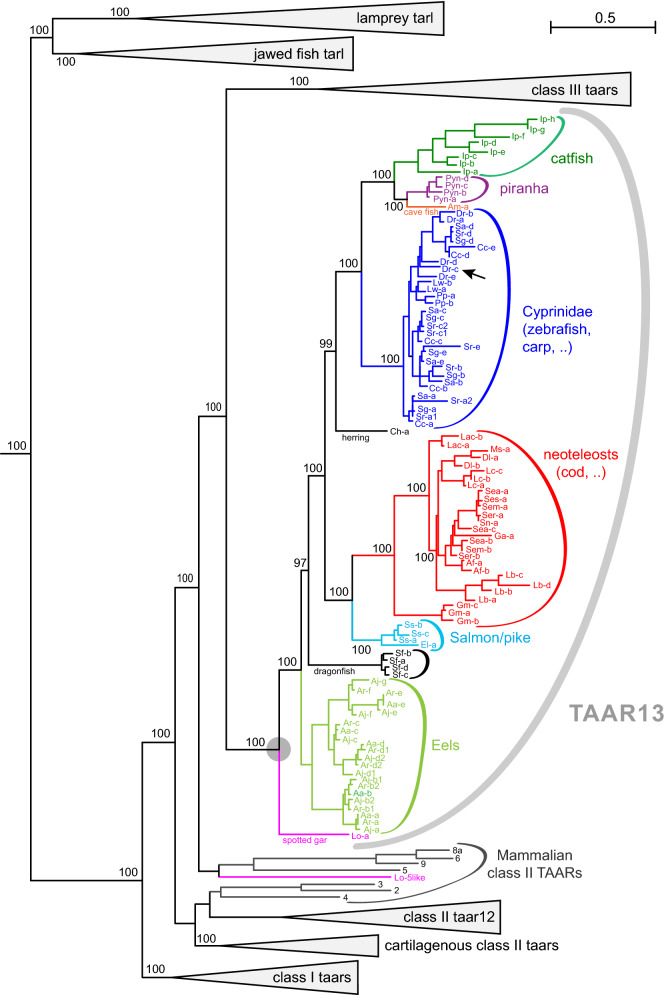
Phylogenetic tree of *taar13* genes. Phylogenetic tree of *taar* and *taar*-like (*tarl*) genes, with all nodes collapsed (grey triangles) except class II *taar13* genes (ancestral node denoted by grey circle) and mammalian class II *taar* genes. Gene set and tree construction are same as in Fig. 1. Species subgroups are visualized by colour code as indicated. Species are indicated by the initials of their Latin names, see Table 1 for full names. Genes are denoted by species abbreviation and unique part of gene name, e.g. Dr-c (black arrow) denotes *taar13c* of zebrafish, *Danio rerio*. Note a “mammalian-like” *taar* gene of the early derived ray-finned fish, spotted gar (Lo-5like, denotes Lo-*taar5like*). Numbers indicate % branch support for basal nodes (cutoff 80%). Scale bar, number of amino acid substitutions per site.

**Figure 7:**
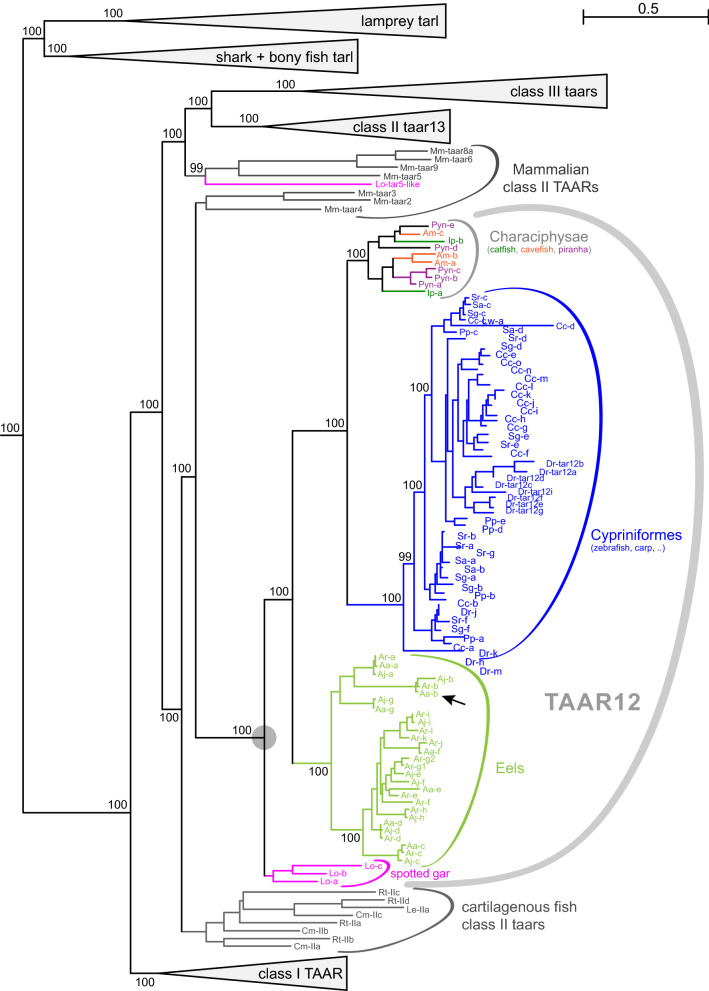
Phylogenetic tree of *taar12* genes. Phylogenetic tree of *taar* and *taar*-like genes, with all nodes collapsed (grey triangles) except class II *taar12* genes (ancestral node denoted by grey circle), cartilaginous and mammalian class II *taar* genes. Gene set and tree construction are same as in Fig. 1. Species are indicated by the initials of their Latin names, see Table 1 for full names. Species subgroups are visualized by colour code as indicated. Genes are denoted by species abbreviation and unique part of gene name, e.g. Aa-b (black arrow) denotes *taar12b* of *Anguilla anguilla*, the European eel. Note large gene expansions in eels and carp. Numbers indicate % branch support for basal nodes (cutoff 80%). Scale bar, number of amino acid substitutions per site.

Further duplications of the ancestral class II *taar* gene may, however, only have occurred after the divergence of cartilaginous and bony fish, since all class II TAARs from three cartilaginous fish species (2 sharks, one ray) have a monophyletic origin (Figs. 6, 7). We observed 3–4 class II *taar* genes in a true shark (whale shark) and a chimera (elephant shark), two of them pseudogenes in each species (SI Table 1). The evolutionary distance between these two species is about 400 million years^19^, but the phylogenetic tree does suggest only a single lineage-specific gene birth event in the whale shark lineage (Fig. 7). Together with a high percentage of pseudogenes this may suggest a reduced importance of the TAAR family in cartilaginous fish olfaction.

The initial *taar* class II gene duplication in the bony fish lineage resulted in the ancestral *taar12* and *taar13* genes of bony fish, and has occurred before the divergence of the tetrapod (sarcopterygian) lineage from that of ray-finned fish (actinopterygii) over 400 million years ago, since both genes have tetrapod ortholog groups, *taar2-4* for *taar12* and *taar5-9* for *taar13* (Figs. 6, 7). Branch support for the *taar12* and *taar13* clade is maximal. A later duplication of the ancestral *taar13* gene within the actinopterygian lineage (ray-finned fish) generated the so-called class III *taar* genes, *cf*.^5^, explaining their absence in tetrapods. An earlier analysis of the evolution of *taar12* and *taar13* in five teleost genomes has suggested a loss of these genes, i.e. class II TAARs, in more modern teleosts^5^. The current analysis was undertaken in part to re-examine that interpretation based on analysis of numerous currently available ray-finned species genomes.

### Early and late losses of the ancestral *taar12* gene result in absence of this gene in the vast majority of analysed species

No behavioral responses have so far been linked to teleost receptors from the *taar12* clade, but a mammalian member of this clade, TAAR4, appears to mediate predator avoidance in rodents via high-affinity detection of 2-phenylethylamine^23^. Interestingly, 2-phenylethylamine also constitutes a high-affinity ligand for zebrafish TAAR12h^24^.

TAAR12 is present as a small subfamily of three genes in the earliest diverging ray- finned fish species, for which a genome is available (spotted gar, Fig. 7). All gene duplications leading to this subfamily have occurred after the divergence from teleost fish since all *taar12* genes of spotted gar constitute a monophyletic clade. TAAR12 is conserved in all examined earlier-derived teleosts except herring and dragonfish (also called bonytongue), who have lost it (Fig. 7). A clear segregation with maximal branch support into *taar12* genes from eels, *Cyprinidae* (e.g. zebrafish and carp), and *Characiphysae* (e.g. piranha and catfish), respectively, shows that TAAR12 has existed as a single gene (Fig. 7) at least until these taxonomic clades diverged about 270 mya^19^. The Characiphysae lineage shows few gene duplications—an ancestral one plus one in cavefish and two in piranha, resulting in two, three, and five genes for catfish, cavefish, and piranha, respectively). Much more frequent gene duplications in cyprinids and eels have resulted in the largest TAAR subfamilies observed in any species, with thirteen and fifteen different *taar12* genes in eel and carp, respectively (Fig. 7, SI Table 1). Often, but not always, closely related direct orthologs are present in each of the three eel species examined, suggesting on one hand that most *taar12* gene duplications occurred before the divergence of these three species (20 mya^19^), and on the other hand some late gene losses in individual eel species. One species- specific gene duplication (*Ar-taar12g1,2*) suggests that gene gains are a sustained feature of *taar* evolution.

**Figure 8:**
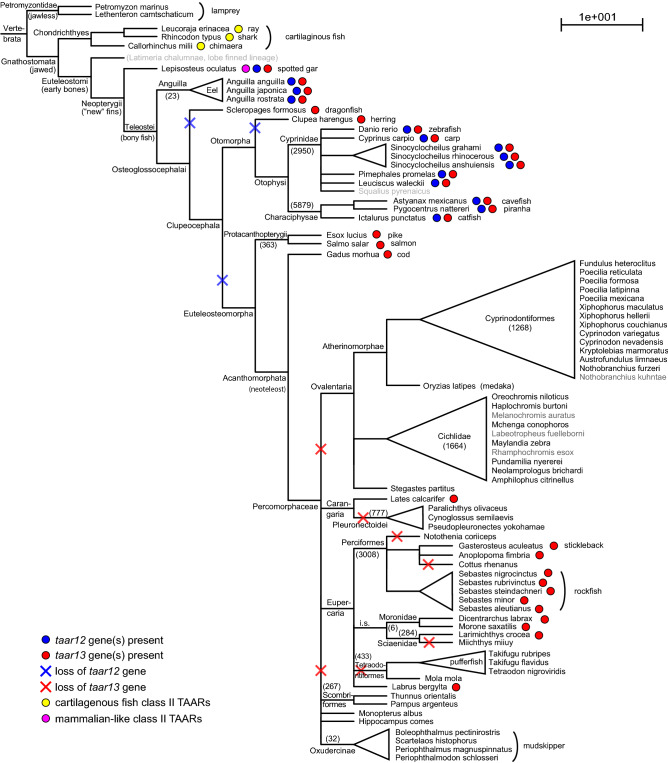
Gene gains and losses visualized in species tree. Phylogenetic species tree including all species whose genomes were analysed. Latin names are shown, for vernacular names see Table 1. Reference species and species with incomplete genome are shown in grey. The tree was drawn based on the phylogenetic relationships shown in^25^. Some higher order classifications are included for clarity, with the number of species in the respective clade given in parentheses^44^. Yellow dots, class II TAARs are present in the respective species; magenta dots, mammalian-like class II TAARs are present; red dots, *taar13* genes are present; blue dots, *taar12* genes are present. Crosses depict gene losses predicted according to maximum parsimony.

In stark contrast, not a single *taar12* gene was detected in any of 58 examined *euteleostomorphan* species, including northern pike, salmon, and cod, which are the most basal species within this clade^25^ (Figs. 7, 8). This strongly suggests that the ancestral *taar12* gene was lost early in the evolution of *Euteleostomorpha* (Fig. 8). This result is consistent with the interpretation gained from an earlier analysis of five species^5^ and pinpoints the loss of the ancestral *taar12* gene to shortly after the divergence of *euteleostomorphan* fish species (Fig. 8), which constitute the majority of extant fish species^25^.

### A high degree of conservation of the ancestral *taar13* gene in early-derived fish contrasts starkly with frequent losses in later-derived fish such as neoteleosts

The TAAR13 subfamily is of particular interest, since one of its members, zebrafish TAAR13c, has been described as a high-affinity receptor for the death-associated odour cadaverine, whose activation appears to drive aversive behavior^7^. Other members of the zebrafish TAAR13 family also process diamine stimuli^26^. We wanted to examine the evolution of potential receptors for this important odorant class throughout the fish lineage, especially because an earlier study of 5 teleost genomes suggested that TAAR13 was absent from later-derived, neoteleost species^5^.

We find that the ancestral *taar13* gene, similar to *taar12*, is already present in an early-derived ray-finned fish, spotted gar, as a single gene(Fig. 8). The TAAR13 subfamily is present in all bony fish species examined until the divergence of *Percomorphaceae*, i.e. also in those earlier-derived species that individually lost TAAR12 (dragonfish and herring), and in earlier-derived *Euteleostomorpha* such as salmon, pike and cod (Fig. 8). Subfamily size ranges from single gene (pike, herring, Mexican cave-fish) to often 3–5 genes (e.g. dragonfish, salmon, zebrafish, carp, piranha), and maximally reaches nine genes in an eel species (SI Table 1), somewhat less than observed for the size of the TAAR12 subfamily. Gene gains occur often late, at the family, and in some cases even genus and species level (e.g. within zebrafish or carp genus), similar to the situation for the TAAR12 subfamily (Fig. 8).

Unexpectedly, we also detected TAAR13c in eleven of 56 *Percomorphaceae* genomes examined, with maximal branch support in all major nodes (Fig. 8). These results supersede that of an earlier study, in which no TAAR13 was detected in four neoteleost (*Percomorphaceae*) genomes^5^. TAAR13 subfamily size ranges from one to four genes per species in this subdivision of neoteleosts, somewhat less than observed for the earlier-derived species discussed in the preceding paragraph. The phylogenetic position of TAAR13c-possessing species suggests at least six independent gene loss events within the *Percomorphaceae* clade, which comprises about 50% of all extant fish species.

## Discussion

Examining twenty early-diverged deuterostome species (six non-vertebrate chordates, two hemichordates, and twelve echinoderms) we pinpoint the origin of *taar* and *tarl* genes to a duplication of the *htr4* gene in the MRCA of vertebrates. This is consistent with earlier results suggesting vertebrate *htr4* as closest relative of *taar* genes^12^. It is instructive to compare the evolution of these three gene families (*htr4*, *tarl* and *taar*) from that point onwards. The *htr4* gene shows a single gene duplication in the MRCA of jawed vertebrates, with rare gene losses (SI Fig. 1), very similar to the evolution of bony fish *tarl* genes (Fig. 3), whereas lamprey *tarl* as well as bony fish *taar* genes are characterized by frequent gene birth and death events both early and late in evolution (Figs. 5,6, 7).

Most likely the *htr4* gene was still mono-exonic at the time of duplication, since vertebrate splice sites, while faithfully conserved down to lamprey, are different from those found in lancelets (SI Table 2). With the exception of a few rather late intron gains (SI Table 1,^5^) *taar* and *taar-like* genes generally have remained mono- exonic, like all other known chemosensory receptor families of the rhodopsin and rhodopsin-related GPCR subclasses^27^.

To examine the evolution of the *taar/tarl* gene family we have performed a comprehensive phylogenetic analysis of *taar* and *tarl* genes in 76 fish genomes. A small subset of class II TAAR repertoires we report here has been analysed previously, in all cases we report either the same^9,28^ or a larger size than previously published^8,10,29,30^, the latter possibly due to subsequent increases in the quality of the genomic databases.

Three species of jawless fish show a large gene expansion of *tarl* genes, clearly distinct by phylogenetic position and motif analysis from *taar* genes, consistent with an earlier analysis for sea lamprey^5^. Expression of lamprey *tarl* genes in sparsely distributed olfactory neurons is consistent with an olfactory function (our results and^21^). The sister group in jawed fish shows an opposing pattern both with respect to near absence of gene duplications and lack of expression in the nose, consistent with a non-olfactory function, possibly as trace amine receptor in brain, the tissue with the highest expression levels in zebrafish.

The earliest occurrence of *taar* genes proper was observed in cartilaginous fish, of which two species, a true shark, and a ray, were newly examined. Both species show 400 million years of evolutionary separation from elephant shark—despite its name not a true shark, but a chimera, and for which we previously reported three class II genes^5,28^. The MRCA of cartilaginous and bony fish already possessed one pair of *taar* genes, ancestral class I and class II gene, respectively (*cf*.^5^).

The subsequent duplication of the ancestral class II gene into the TAAR12 and TAAR13 node occurred within the bony fish lineage, but before the split between tetrapod and teleost lineage about 430 mya^19^. TAAR13 emerges as a single gene in an early-derived ray-finned fish, spotted gar, and orthologs were found in all ray- finned fish species that were earlier-diverging than neoteleosts. Within neoteleosts, we observed a scattered presence of TAAR13, necessitating as most parsimonious explanation at least six independent gene loss events (Fig. 8). These new data supersede the earlier interpretation of a general TAAR13 loss in neoteleosts, based on an analysis of four neotelost genomes^5^. Gene birth events in the TAAR13 lineage occur late, are sometimes species- or genus-specific, often at the level of families, and result in maximally a dozen *taar13* genes (in an eel species, SI Table 1).

The second class II subfamily, TAAR12, shows a similar evolutionary gene birth and death pattern, with somewhat more frequent losses compared to TAAR13. Gene losses already occur in earlier-derived fish, and are spread out over a large evolutionary time scale. Gene birth events occur late, as in TAAR13, and result in maximal subfamily size of 15 genes (common carp, SI Table 1). No other class II subfamilies have been found in any of the ray-finned and teleost fish species examined, suggesting that the class II of *taar* genes in bony fish consisted of only two genes, *taar12* and *taar13* for a long evolutionary period.

Judging from the evolutionary position of *taar12* and *taar13* gene duplications together with the degree of divergence attained between duplicates these gene gains appear to have occurred mostly in the Cretaceous and Tertiary period, cf.^19,25^. We note a considerable asymmetry of gene expansions between TAAR12 and TAAR13, e.g. catfish has only two *taar12* genes, but seven *taar13* genes, whereas carp has fifteen *taar12* genes, and only five *taar13* genes.

Gene losses appear to have occurred during a long time period (Fig. 8), with the potentially earliest events (loss of TAAR12 in the dragonfish and herring lineages) during the Permian and Triassic periods (267 and 222 mya, respectively^19^, and the latest gene losses at the species level occurring in the last 3–20 million years (*Anguilla*, *Sebastes* and *Sinocyclocheilus* genera, cf.^19^). While incompleteness of the respective genome sequence in the database can not be excluded with certainty as cause for any individual inferred gene loss, the frequency of such events at different evolutionary levels and the clear difference between the evolutionary fate of the *taar12* compared to the *taar13* gene, argue for the majority of these gene losses to accurately describe the evolutionary history of the TAAR family. This is supported by the absence of correlation between genome coverage and presence/absence or number of taar genes found (SI Fig. 2).

Overall, a complex picture of class II TAAR evolution emerges, characterized by frequent losses as well as evolutionary late gene gains. We note that the number of gene gains and losses inferred here from an analysis of 74 species may increase with future analysis of additional genomes, e.g. if taar genes are found in a previously negative clade, or are missing in a new species of a previously positive clade. None of these cases leads to a decrease, i.e. our inferences represent a lower limit estimate.

The TAAR family size is very small in cartilaginous fish (sharks, ray) and early-derived ray-finned fish (spotted gar), but reaches maximal size already in the earliest-diverging teleost fish examined (eel). A larger receptor repertoire often is taken to indicate a larger importance of the family. However a small repertoire size can be effectively counterbalanced by a broader ligand spectrum of those receptors, *cf*.^31^. Thus, a larger repertoire might be suggestive of a more fine-grained detection of TAAR ligands. TAARs in general have been found to respond to a broad range of amines^24^ and several behavioural effects of such amines have been observed both in rodents^6^ and in aquatic animals^7,26,32^. Some local regularities in ligand spectra for mammalian TAARs have been observed, e.g. those in the TAAR12 clade respond preferentially to tertiary amines, whereas those in the TAAR13 clade respond to primary amines^33^. However, even small differences in amino acid sequence may result in distinct differences in ligand tuning. Three of the five closely related zebrafish *taar13* genes have been shown with characteristically different response profiles to several diamines and polyamines^7,26^. This is most likely an evolutionary late expansion of function, since neither of these zebrafish *taar13* genes possesses a direct ortholog even in the most closely related species analysed here, common carp and a genus of cave-fish endemic to China, *Sinocyclocheilus*. The ancestral ligand of the TAAR/TARL family might have been serotonin, an aromatic amine – as shown here, the family originated as a duplication of a serotonergic receptor—but already in lamprey an aliphatic polyamine was found as a specific ligand for a TARL receptor^32^. Interestingly, so far behavioural responses mediated via TAAR receptors have been aversive, e.g.^6,7^, whereas the lamprey TARL receptor mediates attraction^32^. Overall, not enough is known currently to understand the evolution of TAAR ligand binding and function. The current study sets the framework for an examination of the evolution of ligand binding in the TAAR and TARL families. In particular the loss of TAAR12 in all and TAAR13 in most of the neoteleost fish species would suggest either compensation by class I and/or class III TAARs or, alternatively, a corresponding change in ecological requirements of more modern fish.

## Materials and methods

### Sequence data acquisition

In this study we examined 81 fish genomes available in the NCBI genome databank at the time of data acquisition. Five genomes lacking full genomic coverage were excluded from further analysis, resulting in 76 fish genomes analysed. The dataset covers important branches in the evolution of fish, including jawless, cartilaginous, and a large variety of ray-finned fish reaching from the spotted gar (*L. oculatus*) as the most ancestral species to many neoteleost species. Moreover we investigated the genomes of six non-vertebrate chordates, two hemichordates and 12 echinoderms to pinpoint the evolutionary origin of the TAAR family. Wherever possible we used genome assemblies, otherwise we examined the whole-genome shotgun sequence contigs, see (SI Table 1).

Searches were performed using tBLASTn^34^, with the amino acid sequence of *D. rerio* TAAR13c as initial query sequence. In some cases amino acid sequences from other class I-III *taar* genes, *tarl* and *htr* genes were used as queries. Candidates with a minimal sequence length of 200 amino acids were selected for validation by phylogenetic analysis (see below). The search was continued until at least ten consecutive non-class II *taar* genes were found or until an e-value of -10 was reached, whichever came first.

### Phylogenetic analysis

In a first step a reference tree was constructed using known class I-III TAARs and a selection of ORs and aminergic receptors as out-groups^5^. Candidate genes were added batch-wise to this reference tree and evaluated according to their position in the tree. Alignment of amino acid sequences was performed using MAFFT version 7^35^. Aligned sequences were stripped of gap positions at 90% tolerance level (≥ 90% gaps at that position) using Gap Strip/Squeeze version 2.1.0. (https://www.hiv.lanl.gov/content/sequence/GAPSTREEZE/gap.html). The phylogenetic tree was calculated according to^36^ using a Maximum likelihood algorithm, PhyML-aLRT with smart model selection^37^, SPR setting for tree optimization and chi square-based aLRT for branch support^38^ available online^39^. Class II TAARs were identified by their position among the known class II members from zebrafish. TARLs were identified initially by their unique position in the tree, between Class I TAARs and a family of serotonin receptors (*htr4*) that were used as out-group. Additionally *D. rerio* TARL was used as query for a tBLASTn search in all genomes examined. TARL genes from jawed fish are highly conserved and were easily identifiable by their high sequence similarity, usually ~ 95% identity among closely related species, but at least ~ 85% even among remotely related species.

Genomic sequences of all candidate genes were then extended up to 2 kb in the 5′ and 3′ direction to identify the complete coding region including start and stop codons. Amino acid sequence was predicted using ExPASy^40^. For incomplete sequences (as judged from multiple sequence alignment) an attempt was made to obtain the complete amino acid sequence by genewise prediction^41^ using up to 20 kb genomic region and the most closely related full length sequence as template. A few amino acid sequences could not be completed because of gaps in the databanks (see SI Table 1).

After validation of all candidate genes, a PhyML tree was constructed using all validated sequences (205 class II TAARs, 84 lamprey TARLs, and 86 jawed-fish TARLs) together with a selection of reference TAARs (classes I, II, and III) and a total of 102 aminergic receptors (including 17 *htr-4* genes) as out-group. Tree construction was as described above. The tree file for Fig. 1 is given in SI File 1. For a complete list of all amino acid sequences used see SI File 2.

For bony vertebrates, newly predicted *taar* and *tarl* genes were named according to phylogenetic position in relation to already named genes, wherever possible. For jawless fish we named according to phylogenetic position, with numbers referring to subfamilies and letters to individual genes in the subfamily, e.g. *Lec-tarl1a* corresponds to the least-derived gene in the *tarl1* subfamily, see Fig. 5.

### Inference of gene birth and death events

Gene tree topology mostly reproduced that of the taxonomic tree, with e.g. eel genes always more basal than cyprinid genes, and genes from spotted gar, an early-derived ray-finned fish, always more basal than those of teleost fish (e.g. eels, cyprinids). Thus inferring gene birth and death events was mostly straight-forward. For example, TAAR12 was found in eels, catfish, and zebrafish, but not in herring, so it was concluded to be lost in the herring lineage (Fig. 8). All eel *taar12* genes are monophyletic, so all *taar12* gene gains in eels have occurred after the segregation of this lineage from other lineages examined here (Fig. 7). In these cases, the tree topology fit to the most parsimonious explanation requiring least gene birth/death events. In a few exceptions we (manually) considered maximal parsimony to infer gene birth/death events, not the tree topology, since in our experience a small number of genes in a particular clade can distort its position in the tree. This is the case for the class II TAARs of cartilaginous fish, which group with TAAR12, but would be expected to lie ancestral to both TAAR12 and TAAR13. The other case concerns a 'mammalian-like' *taar* gene of the spotted gar, TAAR5, which would have to be considered a third ancestral *taar* gene beyond *taar12* and *taar13*, if its topological position is taken at face value. Availability of further genomes will allow to further refine the tree topology.

### RT-PCR

Total RNA was extracted from zebrafish tissues (OE, brain, trunk) using Trizol (Thermo Fisher) according to the manufacturer’s instructions. The SuperScript III kit (Thermo Fisher) was used to synthesize cDNA from tissue specific zebrafish total RNA samples according to the manufacturer’s instructions. After an RNase A (Roche) digestion step, cDNA concentration was determined with a NanoDropTM photometer and samples stored at -20 °C. Gene expression was probed by PCR amplifications using standard PCR protocols with the following primers: Dr_*actinb1* (forward, CCCCATTGAGCACGGTATTG; reverse, TCACACCATCACCAGAGTCC); Dr_*tarl1* (primer pair 1: forward, TTCACGAGTCGCCCTCTATC; reverse, ATAGGCCACCAACATGGTCA, primer pair 2: forward, AGCCTCCATTTTCCACCTGA; reverse, CCCATGATGATCCCTAGCGT). Because the *tarl* genes are mono-exonic it was not possible to design intron-spanning primers for Dr_*tarl1*. In order to exclude genomic DNA contamination of the cDNA sample we performed a control PCR run with intron-spanning primers of the *actin b1* gene side by side with the *tarl* gene. We did not detect any signs of genomic DNA contamination in these controls.

### In situ hybridization

Probes were prepared by PCR using for Dr_*tarl1* the primer pairs described above, and for Lf_*tarl7a* the following primer pair (forward, CCGCAACGCGTGGTCCTGAT; reverse, TCCTAAAGTTGAATAGATCCGTC). For cryosections of adult olfactory epithelium the protocol given in^42^ was followed, except permeabilization was performed in 0.2 HCL in DEPC-treated water for 10 min and the H_2_O_2_ step was omitted. For whole mount in situ hybridization of zebrafish larvae the larvae were raised in 45 mg/l N-Phenylthiourea to minimize pigmentation. The in situ hybridization then was performed as described^43^.

### Color presentation

The color scale of figures can be optimized for various forms of color blindness using the software tool visolve (https://www.ryobi.co.jp/products/visolve/en/download/).

### Animal handling and care statement

Animal handling and care was approved by the governmental animal care and use office (Landesamt für Natur, Umwelt und Verbraucherschutz Nordrhein- Westfalen, Recklinghausen, Germany, Protocol No. 8.87–51.05.20.10.217) and was in accordance with the German Animal Welfare Act as well as with the General Administrative Directive for the Execution of the Protection of Animals Act. Every effort was made to minimize animal suffering and to reduce the number of animals used during the study.

### Methods statement

All methods were carried out in compliance with local safety regulations and applicable ARRIVE guidelines.

## Supplementary Information

Supplementary Information

## Data Availability

All data generated or analysed during this study are included in this published article and its supplementary information files.
