# Supplementary material for: Olfactory function in the trace amine-associated receptor family (TAARs) evolved twice independently
Source: Sci Rep. 2021 Apr 8;11:7807. doi: 10.1038/s41598-021-87236-5 (PMC8032801; doi:10.1038/s41598-021-87236-5)
Supplement: Supplementary file 1 — Supplementary Information [file 41598_2021_87236_MOESM1_ESM.pdf]

SUPPLEMENTARY INFORMATION

Olfactory function in the trace amine-associated receptor family (TAARs) evolved twice independently

Milan Dieris[1], Daniel Kowatschew[1], and Sigrun I. Korsching[1]*

[1]Institute for Genetics, University at Cologne, Germany
*Corresponding author, sigrun.korsching@uni-koeln.de

SUPPLEMENTARY INFORMATION
Table of Contents

Olfactory function in the trace amine-associated receptor family (TAARs) evolved twice independently

Milan Dieris[1], Daniel Kowatschew[1], and Sigrun I. Korsching[1]*

[1]Institute for Genetics, University at Cologne, Germany
*Corresponding author, sigrun.korsching@uni-koeln.de

**Supplementary File 1**	Treefile for phylogenetic tree shown in Figure 1
**Supplementary File 2**	Amino acid sequences used in phylogenetic trees
**Supplementary Figure 1**	Phylogenetic tree for *htr4* in deuterostomes and inferred gene gain/loss events
**Supplementary Figure 2**	No correlation between genome coverage and detection of *taar* genes
**Supplementary Table 1**	Genomic location of all genes analysed
**Supplementary Table 2**	Intron position in *htr4, taar* and *tarl* genes

SUPPLEMENTARY INFORMATION
SI File 1: Treefile for phylogenetic tree shown in Figure 1

Olfactory function in the trace amine-associated receptor family (TAARs) evolved twice independently

Milan Dieris[1], Daniel Kowatschew[1], and Sigrun I. Korsching[1]*

[1]Institute for Genetics, University at Cologne, Germany
*Corresponding author, sigrun.korsching@uni-koeln.de

(Dr-hrh3:0.24676539,Dr-hrh4:0.31957204,(Rt-hrh3:0.41577716,(((((Dr-
chrm2a:0.23836639,Mm-chrm4:0.15876449)0.999850:0.30016013,(Pm-chrm:0.36384753,
(Mm-chrm1:0.27092826,(Dr-chrm3b:0.25374974,Rt-
chrm:0.14654995)0.999850:0.06305782)0.996034:0.05868278)0.935389:0.05795016)0.99
9850:0.08692529,((Cin-chrm:0.90410508,Csa-
chrm:0.38530661)0.999850:0.30891539,Csa-
chrm:0.75119528)0.822525:0.04832224)0.913698:0.04206302,Bbe-
chrm:0.62263541)0.999850:0.30296837,((Mm-hrh1:0.39115068,Dr-
hrh1:0.47328549)0.999850:0.59562034,((((Alu-hrh2:0.06917687,Bfl-
hrh2:0.02587605)0.999850:0.48651330,((Le-hrh2:0.23961964,Rt-
hrh2:0.11097566)0.999850:0.19464467,(Rat-hrh2:0.36840916,(Dr-
hrh2b:0.58668493,Dr-
hrh2:0.37509001)0.999850:0.18502646)0.306735:0.07434492)0.999850:0.36009870)0.99
9850:0.30122204,((((Alu-drd1:0.02611853,Bfl-drd1:0.07043786)0.999850:0.52964915,
((Pm-drd1-li:0.14750967,(Cm-drd1:0.05162892,(Mm-drd1a:0.13497337,Rt-
drd1:0.07698318)0.937383:0.02947751)0.999850:0.10111717)0.502579:0.03646106,(Mm-
drd5:0.17413011,(Le-drd5:0.14196214,Rt-
drd5:0.26380078)0.999850:0.09521429)0.808687:0.03142950)0.999850:0.37198577)0.99
9850:0.28416326,(Alu-adrb:0.58475677,((Cin-adrb:0.14988489,Csa-
adrb:0.20795392)0.999850:0.62433763,(Lec-adrb:0.36176346,(Rat-adrB:0.32673892,
(Mm-adrB1:0.27618311,(Cm-adrb1:0.07820835,Rt-
adrb:0.13868889)0.999850:0.12767985)0.999850:0.20560585)0.999850:0.15101689)0.99
9850:0.22256097)0.999850:0.26777162)0.998122:0.13475217)0.846944:0.03065414,
((Dr-htr6:0.34249903,(Mm-htr6:0.28831387,Rt-
htr6:0.21947107)0.879382:0.14452807)0.999850:0.95530190,(((Csa-htr2:0.63733999,
(Dr-htr2b:0.36358147,Rat-
htr2A:0.34696960)0.999850:0.29260830)0.999850:0.65214280,(Cin-AmR:2.56284993,
(Csa-AmR2:1.83930779,Csa-
AmR1:2.18937513)0.652523:0.27755418)0.935928:0.34853373)0.976307:0.15996515,
(((Lec-htr4:0.01447018,Pm-htr4:0.00000054)0.999850:0.31774254,(Cm-htr4-
li:0.10684636,((Lo-htr4-li:0.06786645,(Ga-htr4lik:0.13298571,(Aj-htr4-
li:0.05030884,Dr-htr4-
li:0.16972196)0.000000:0.00000162)0.999850:0.05322457)0.999850:0.13535638,(((Le-
htr4:0.06002770,(Cm-htr4:0.12449484,Rt-
htr4:0.05276104)0.544556:0.01172978)0.999850:0.10337016,(Mm-htr4:0.12240354,(Np-
htr4:0.01628215,(Xt-htr4:0.01129608,Xl-
htr4.S:0.01570625)0.999850:0.05670638)0.999850:0.04268385)0.999850:0.04918023)0.
999850:0.03513551,(Lo-htr4:0.03415163,(Ga-htr4:0.07239665,Dr-
htr4:0.06816315)0.985516:0.03280411)0.999850:0.08625416)0.999850:0.10721738)0.73
9533:0.04739138)0.999850:0.14849016)0.999850:0.43652049,((((Mm-
taar1:0.47286937,Dr-tar1b:0.65063192)0.992733:0.13289159,(Ga-tar21a-:0.85976240,
(Ga-tar27-I:0.42004456,(Dr-tar10a:0.40339781,Dr-
tar11:0.70454644)0.999850:0.16877051)0.737848:0.04487660)0.999850:0.08751675)0.9
99048:0.11637536,((((Cm-cls2a:0.26362720,Rt-
cls2b:0.42091354)0.982990:0.07348347,(Cm-cls2b:0.29718849,(Rt-cls2a:0.38477040,
((Cm-cls2c:0.22800950,(Le-cls2a:0.24803889,Rt-
cls2d:0.17367670)0.999850:0.18426999)0.999850:0.20575142,Rt-
cls2c:0.52690554)0.459606:0.01598639)0.999850:0.05975314)0.999850:0.07420491)0.9
99850:0.09443532,(((Lo-tar12a:0.18054532,(Lo-tar12b:0.12938879,Lo-
tar12c:0.27394026)0.999850:0.08321977)0.748286:0.02949065,((((Aj-
tar12c:0.00814908,(Ar-tar12c:0.00876208,Aa-
tar12c:0.01656724)0.997964:0.00866131)0.999850:0.14404883,((Ar-
tar12d:0.00395044,(Aj-tar12d:0.00943753,Aa-
tar12d:0.00000028)0.000000:0.00000028)0.999850:0.03718766,((Aj-
tar12h:0.00804933,Ar-tar12h:0.01113030)0.999850:0.11564816,((Ar-
tar12f:0.11504179,((Ar-tar12e:0.02987765,Aa-
tar12e:0.11768555)0.941465:0.01560763,(Aj-tar12f:0.06683651,(Aj-
tar12e:0.03433596,(Ar-tar12g1:0.01079682,Ar-
tar12g2:0.03091654)0.917417:0.02159438)0.985757:0.01390477)0.999850:0.02282665)0
.999850:0.01267671)0.999850:0.01191076,((Aa-tar12f:0.00480622,Ar-
tar12j:0.02355438)0.999850:0.14515184,(Ar-tar12k:0.03907353,(Ar-
tar12l:0.03786379,(Aj-tar12i:0.00545890,Ar-
tar12i:0.00000035)0.999850:0.01991054)0.999850:0.03340989)0.999850:0.06410289)0.
988824:0.01320005)0.994335:0.01490175)0.998461:0.01964048)0.995160:0.03455531)0.

999850:0.12777468,((Aa-tar12g:0.00000019,Aj-
tar12g:0.00784727)0.999850:0.13180745,((Aa-tar12b:0.00573801,(Ar-
tar12b:0.00812923,Aj-tar12b:0.06735604)0.966324:0.00502469)0.999850:0.24749391,
(Aj-tar12a:0.00271621,(Aa-tar12a:0.00811185,Ar-
tar12a:0.00000001)0.960572:0.00267639)0.999850:0.07767642)0.999850:0.05604820)0.
951539:0.03876976)0.999850:0.15498306,((Dr-tar12m:0.23793387,(((Dr-
tar12h:0.02570174,Dr-tar12k:0.07649903)0.999850:0.05092943,(Cc-
tar12a:0.02397292,(Pp-tar12a:0.04788727,(Sg-tar12f:0.00000093,Sr-
tar12f:0.00000001)0.544221:0.00592596)0.563879:0.00937587)0.991819:0.01610977)0.
999850:0.07134487,((Dr-tar12j:0.06492526,(Cc-tar12b:0.03705748,(Pp-
tar12b:0.06894633,((Sg-tar12b:0.02395853,Sg-
tar12a:0.00295171)0.984267:0.00307609,((Sa-tar12b:0.02677800,Sa-
tar12a:0.00000031)0.998194:0.00317267,(Sr-tar12g:0.07091753,(Sr-
tar12a:0.00000001,Sr-
tar12b:0.00592408)0.971666:0.00296089)0.000000:0.00000018)0.999850:0.00591075)0.
999850:0.01432180)0.674955:0.00429501)0.999850:0.01958542)0.999850:0.02512534,
(((Pp-tar12d:0.04091879,Pp-tar12e:0.04646179)0.999850:0.03658701,((((Dr-
tar12g:0.08008580,(Dr-tar12e:0.01488872,Dr-
tar12f:0.01813567)0.999850:0.06589440)0.999850:0.03126015,(Dr-tar12i:0.13223268,
(Dr-tar12c:0.02624894,(Dr-tar12d:0.00000030,(Dr-tar12a:0.04514789,Dr-
tar12b:0.07332299)0.999850:0.08360211)0.999850:0.07349344)0.999850:0.01518082)0.
999850:0.02343658)0.999850:0.04842838,((Cc-tar12f:0.10882398,((Sr-
tar12e:0.03397657,(Sg-tar12e:0.04401597,Cc-
tar12g:0.04360600)0.992603:0.00945485)0.999850:0.03326054,((Cc-
tar12h:0.02079783,(Cc-tar12i:0.05243736,(Cc-tar12j:0.03979989,(Cc-
tar12k:0.01399765,(Cc-tar12l:0.00463105,Cc-
tar12m:0.03379334)0.999850:0.02308023)0.887640:0.01068143)0.854200:0.01163611)0.
785441:0.00638242)0.999850:0.04926907,(Cc-tar12n:0.05298771,Cc-
tar12o:0.02590717)0.999850:0.03107037)0.987571:0.01294766)0.927221:0.00968768)0.
999850:0.02436811,(Cc-tar12e:0.06577515,(Sg-tar12d:0.01614013,(Sr-
tar12d:0.03170200,Sa-
tar12d:0.01619842)0.865510:0.00638801)0.999850:0.07440204)0.979219:0.01334920)0.
999850:0.01539858)0.999850:0.02294015,Lw-
tar12a:0.06612126)0.722060:0.00708304)0.999850:0.05850233,(Pp-tar12c:0.05255924,
(Cc-tar12d:0.40881748,(Cc-tar12c:0.02588532,(Sg-tar12c:0.00816445,(Sa-
tar12c:0.02014348,Sr-
tar12c:0.01689451)0.998338:0.00585038)0.999850:0.01793162)0.999850:0.03496443)0.
977413:0.02777406)0.999850:0.04822927)0.999850:0.02972271)0.999850:0.04268421)0.
993519:0.04152698)0.999850:0.22361345,((Ip-tar12a:0.17138516,((Pyn-
tar12a:0.02009761,(Pyn-tar12b:0.02068766,Pyn-
tar12c:0.03280130)0.999850:0.05976457)0.999850:0.07537298,(Am-
tar12a:0.07706711,Am-
tar12b:0.09407007)0.999850:0.07985748)0.999850:0.03983179)0.999850:0.05569566,
(Pyn-tar12d:0.20535798,(Ip-tar12b:0.19621199,(Am-tar12c:0.07799056,Pyn-
tar12e:0.11281833)0.991016:0.01677973)0.999850:0.06658912)0.999850:0.03937316)0.
999850:0.08589536)0.999850:0.21912920)0.999850:0.10690216)0.999850:0.27679921,
(Mm-taar4:0.45766329,(Mm-taar2:0.34412764,Mm-
taar3:0.27603507)0.999850:0.31178757)0.688286:0.04163315)0.998602:0.05435799)0.9
97888:0.07804196,((Lo-tar5lik:0.66061107,(Mm-taar5:0.56053515,(Mm-
taar9:0.15928548,(Mm-taar6:0.14327703,Mm-
taar8a:0.12944958)0.999850:0.15251723)0.999850:0.46925230)0.999850:0.13908051)0.
993268:0.07608524,((Dr-tar14f:0.98757554,(Dr-tar15a:0.82108800,((Ga-
tar22a-:0.35568860,(Tr-tar28a-:0.78518174,(Ga-tar24-I:0.28871230,(Ga-tar23-
I:0.60883589,(Ga-tar25a-:0.16826710,Ga-
tar26a:0.33962781)0.999850:0.06959879)0.971852:0.05020380)0.881394:0.04426853)0.
926899:0.06929933)0.999850:0.46976838,(Dr-tar16a:0.64229491,((Dr-
tar19a:0.12204469,Dr-tar20a:0.11122322)0.999850:0.10733284,(Dr-
tar18a:0.22245523,Dr-
tar17a:0.29441052)0.716708:0.06273417)0.999850:0.34693412)0.962490:0.11711218)0.
912624:0.08623902)0.999850:0.19686514)0.999850:0.35523809,(Lo-tar13a:0.26726476,
((((Aj-tar13a:0.00779644,(Ar-tar13a:0.00000014,Aa-
tar13a:0.00381538)0.950006:0.00325376)0.999850:0.05271148,((Ar-
tar13b1:0.00970426,Aj-tar13b2:0.01536613)0.999850:0.01245774,(Aa-
tar13b:0.00692736,(Ar-tar13b2:0.00311520,Aj-

tar13b1:0.01680001)0.984206:0.00989090)0.994834:0.00990322)0.981299:0.00989772)0
.999850:0.11519119,(((Aj-tar13d1:0.03220012,(Ar-tar13d2:0.00931293,(Aj-
tar13d2:0.01346970,(Ar-tar13d1:0.01360259,Aa-
tar13d:0.00537461)0.999043:0.01017662)0.995490:0.00848632)0.999850:0.04700179)0.
999850:0.04112657,(Aj-tar13c:0.00875369,(Aa-tar13c:0.00417586,Ar-
tar13c:0.00822931)0.000000:0.00000040)0.999850:0.01235655)0.857921:0.01756646,
((Aj-tar13f:0.01745246,(Aj-tar13e:0.00368234,(Aa-tar13e:0.00000001,Ar-
tar13e:0.00000013)0.999850:0.01617792)0.999850:0.10280276)0.994569:0.01079701,
(Ar-tar13f:0.00805903,Aj-
tar13g:0.05992404)0.999850:0.01285237)0.999850:0.07848675)0.999850:0.06048049)0.
999850:0.04451128,(((Sf-tar13c:0.01625874,Sf-
tar13d:0.01386470)0.999850:0.01339674,(Sf-tar13a:0.01102512,Sf-
tar13b:0.03502087)0.987019:0.00638151)0.999850:0.28355183,(((El-
tar13a:0.14022865,(Ss-tar13a:0.00301377,(Ss-tar13c:0.03912318,Ss-
tar13b:0.01425134)0.999850:0.01483281)0.999850:0.04852575)0.999850:0.11595228,
((Gm-tar13b:0.04234700,(Gm-tar13a:0.00733455,Gm-
tar13c:0.03392359)0.736141:0.00799303)0.999850:0.18728793,(((Lb-
tar13a:0.03347673,(Lb-tar13b:0.03355368,(Lb-tar13d:0.12210882,Lb-
tar13c:0.01184002)0.999850:0.06299901)0.999850:0.06428006)0.999850:0.11256138,
(((Af-tar13b:0.03457169,Af-tar13a:0.00466834)0.999850:0.06594195,(Ser-
tar13b:0.02551372,((Sem-tar13b:0.00000107,Sea-
tar13b:0.01894113)0.999850:0.03623446,(Ga-tar13a:0.14153540,(Sea-
tar13c:0.04422314,((Sn-tar13a:0.00000015,Ser-
tar13a:0.00567326)0.858136:0.00688223,(Sem-tar13a:0.00423764,(Ses-
tar13a:0.01024607,Sea-
tar13a:0.00797969)0.000000:0.00323865)0.195938:0.00479574)0.999850:0.07038173)0.
949204:0.01349270)0.985289:0.01068051)0.536452:0.00731494)0.999850:0.02250446)0.
999850:0.02300753,((Lc-tar13a:0.00765520,(Lc-tar13b:0.00000016,Lc-
tar13c:0.04943454)0.999850:0.02896777)0.999850:0.08117975,(Dl-tar13b:0.02460219,
(Dl-tar13a:0.03654104,Ms-
tar13a:0.06694012)0.999850:0.06231328)0.999850:0.04585415)0.989539:0.01543483)0.
860158:0.01061630)0.972828:0.02922728,(Lac-tar13a:0.00452572,Lac-
tar13b:0.06418852)0
.999850:0.05161364)0.999850:0.13408344)0.999850:0.15944205)0.999850:0.10551301,
(Ch-tar13a:0.19757730,(((Cc-tar13a:0.01692920,(Sr-tar13a1:0.00557530,(Sg-
tar13a:0.00841202,(Sr-tar13a2:0.13591302,Sa-
tar13a:0.00841998)0.000000:0.00000019)0.000000:0.00000019)0.999850:0.01124200)0.
999850:0.02182515,(((Cc-tar13b:0.03206733,((Sa-tar13b:0.05292088,(Sg-
tar13b:0.04413214,Sr-tar13b:0.03287993)0.999850:0.01420815)0.999850:0.03264201,
(Sa-tar13e:0.00281525,(Sg-tar13e:0.00577988,Sr-
tar13e:0.14016125)0.862251:0.00264096)0.999850:0.00828842)0.844949:0.00376549)0.
999850:0.02273577,(Cc-tar13c:0.01508147,(Sr-tar13c1:0.01336076,(Sr-
tar13c2:0.00640174,(Sg-tar13c:0.00000016,Sa-
tar13c:0.00839394)0.000000:0.00000031)0.998726:0.00667546)0.999850:0.01211544)0.
999850:0.01569366)0.999850:0.01795155,(((Pp-tar13b:0.03096006,Pp-
tar13a:0.03582113)0.999850:0.03333564,(Lw-tar13a:0.02719176,Lw-
tar13b:0.04858004)0.696482:0.00602324)0.999850:0.01886724,(Dr-tar13e:0.07606128,
(Dr-tar13c:0.07441344,(Dr-tar13d:0.06238559,(((Cc-tar13d:0.00000001,Cc-
tar13e:0.09533489)0.999850:0.01711990,(Sg-tar13d:0.01100950,(Sr-
tar13d:0.00550158,Sa-
tar13d:0.00570233)0.973362:0.00294877)0.999850:0.01740367)0.999850:0.04845768,
(Dr-tar13a:0.01718376,Dr-
ar13b:0.06049293)0.999850:0.02521716)0.999850:0.04066857)0.667987:0.00744833)0.9
85131:0.01240770)0.999850:0.02386771)0.941923:0.00875673)0.999850:0.02030331)0.9
99850:0.16272222,((Am-tar13a:0.14339943,(Pyn-tar13a:0.00890797,(Pyn-
tar13b:0.04445325,(Pyn-tar13c:0.01727276,Pyn-
tar13d:0.02111060)0.999850:0.02772502)0.521059:0.01093092)0.999850:0.07365720)0.
999850:0.04888871,(Ip-tar13a:0.15731666,((Ip-tar13b:0.03472637,Ip-
tar13c:0.04162423)0.999850:0.05643079,((Ip-tar13e:0.06479198,Ip-
tar13d:0.03252145)0.999850:0.09204287,(Ip-tar13f:0.13469696,(Ip-
tar13g:0.00000348,Ip-
tar13h:0.03529954)0.999850:0.20623584)0.999850:0.10678970)0.999850:0.04925202)0.
999850:0.05621948)0.999850:0.09038305)0.999850:0.13110683)0.999850:0.09480200)0.
992587:0.04579693)0.999850:0.06393921)0.970204:0.02466895)0.999850:0.08719527)0.

999850:0.20141635)0.999850:0.10696582)0.999850:0.08932990)0.999850:0.12922095)0.
999850:0.42528632,(((Lec-tarl1a:0.00790559,(Pm-tarl1a:0.03964542,(Lec-
tarl1b:0.02222799,Lec-
tarl1c:0.02889833)0.999850:0.01642326)0.957502:0.01996955)0.999850:0.61564000,
(((((Lec-tarl4a:0.00289775,(Pm-tarl4b:0.01524309,Pm-
tarl4a:0.01439959)0.999850:0.01844590)0.999850:0.15947466,((Lec-
tarl4d:0.04011483,Lec-tarl4c:0.03838594)0.999850:0.09627386,(Lec-
tarl4b:0.00485790,Laf-
tarl4a:0.00819095)0.975954:0.01497276)0.999850:0.18046179)0.999850:0.44728635,
(Lec-tarl4e:0.82081613,(Pm-tarl2c:0.00362599,(Lec-tarl2b:0.10219182,Pm-
tarl2b:0.06021135)0.999850:0.12322465)0.999850:0.54875578)0.764686:0.11198326)0.
994446:0.07611584,((((Lec-tarl2a:0.03175478,Pm-
tarl2a:0.01494592)0.999850:0.74436496,(Lec-tarl5a:0.01146574,pm-
tarl5a:0.02067713)0.999850:0.44949316)0.466301:0.02596798,(Lec-
tarl5d:0.50822464,(Lec-tarl5c:0.02401233,(Pm-tarl5d:0.00000470,(Pm-
tarl5c:0.00527240,(Pm-tarl5b:0.00585350,Lec-
tarl5b:0.10955285)0.999850:0.02184534)0.999850:0.02783344)0.629689:0.01537419)0.
999850:0.45299650)0.985217:0.05885009)0.999850:0.09899452,((((Lec-
tarl6a:0.03486141,(Pm-tarl6a:0.01255669,(Laf-tarl6a:0.01062480,Lec-
tarl6b:0.00258494)0.999850:0.00865363)0.711376:0.01753955)0.999850:0.49363316,
(((Lec-tarl6c:0.06353737,(Pm-tarl6b:0.03189020,(Lec-tarl6d:0.02360581,(Lec-
tarl6e:0.01975643,(Lec-tarl6f:0.01336302,(Laf-tarl6c:0.03408358,Lec-
tarl6g:0.01055989)0.974021:0.00357735)0.999850:0.02325161)0.942163:0.00992531)0.
999850:0.03676819)0.999850:0.07315397)0.999850:0.33694385,(((Lec-
tarl6i:0.00000001,Laf-tarl6b:0.01219393)0.940314:0.02007576,(Pm-
tarl6d:0.00795676,(Lec-tarl6h:0.03153758,Pm-
tarl6c:0.01479214)0.999850:0.04047097)0.968951:0.00967778)0.942999:0.01484531,
(Lec-tarl6j:0.03565165,Pm-
tarl6e:0.02645303)0.999850:0.04295404)0.999850:0.30685157)0.999850:0.12712469,
((((Lec-tarl6n:0.03364649,Pm-tarl6i:0.00873626)0.999850:0.13403607,((Pm-
tarl6j:0.00000104,(Lec-tarl6o:0.00000001,Laf-
tarl6f:0.00534264)0.999850:0.04049846)0.999850:0.17363902,(Pm-tarl6k:0.03308196,
(Lec-tarl6p:0.02074477,Lec-
tarl6q:0.01692187)0.985698:0.00644355)0.905458:0.03179745)0.999850:0.08675412)0.
999850:0.33513899,((Lec-tarl6r:0.01696365,Laf-
tarl6e:0.00000001)0.999850:0.40159401,(Lec-tarl6s:0.26979955,Lec-
tarl6t:0.52131849)0.999850:0.09518288)0.786130:0.04924264)0.999850:0.10898678,
((Lec-tarl6k:0.03344189,Pm-tarl6f:0.02597226)0.999850:0.41710925,((Pm-
tarl6g:0.02229045,(Laf-tarl6d:0.01036545,Lec-
tarl6l:0.00386400)0.934531:0.01110469)0.999850:0.23529956,(Lec-
tarl6m:0.03817321,Pm-
tarl6h:0.02752742)0.999850:0.42848729)0.999850:0.12938913)0.999850:0.06464249)0.
999236:0.05994753)0.999850:0.07875798)0.999850:0.08810353,(((Lec-
tarl6u:0.01664756,(Pm-tarl6l:0.00715776,Laf-
tarl6g:0.02226184)0.848347:0.00379002)0.999850:0.04210666,(Lec-
tarl6v:0.02597421,Pm-tarl6m:0.02783418)0.999850:0.05641701)0.999850:0.44722365,
((Lec-tarl6w:0.01491882,Pm-tarl6n:0.03198923)0.990937:0.03148620,(Lec-
tarl6x:0.02784344,Pm-
tarl6o:0.03180105)0.999850:0.03940143)0.999850:0.38916381)0.708841:0.03646831)0.
999850:0.26440039,((Lec-tarl7a:0.00298804,Pm-
tarl7a:0.03134323)0.999850:0.47454781,((Lec-tarl7b:0.00491188,Pm-
tarl7b:0.06725972)0.999850:0.57383671,((Lec-tarl7c:0.26523731,(Lec-
tarl7d:0.00000046,Pm-tarl7c:0.03434303)0.999850:0.36000327)0.999850:0.24896426,
(((Lec-tarl7e:0.00385461,Pm-tarl7d:0.10895670)0.999850:0.22244149,(Lec-
tarl7f:0.13617660,(Lec-tarl7g:0.01788309,Pm-
tarl7e:0.01781698)0.999850:0.17792632)0.999850:0.16972834)0.999850:0.12654497,
((Lec-tarl7h:0.01028640,Laf-tarl7a:0.01343017)0.999850:0.36945889,(Lec-
tarl7i:0.13667722,(Lec-tarl7j:0.02482053,Pm-
tarl7f:0.02478946)0.999850:0.14603864)0.999850:0.12706298)0.999850:0.18717182)0.
960833:0.05683235)0.999850:0.08169440)0.868813:0.09213515)0.999850:0.13505762)0.
999850:0.08933318)0.999850:0.09295030)0.838359:0.03628224,(Lec-
tarl3a:0.42814318,(Lec-tarl3b:0.06226201,(Lec-tarl3c:0.02679438,pm-
tarl3a:0.04443766)0.999850:0.07369104)0.999850:0.50007288)0.755827:0.12345724)0.
891077:0.09024302)0.999850:0.31353267,(((Cm-tarl2:0.31176007,(Le-

tarl2:0.23565455,Rt-tarl2:0.15758857)0.996185:0.07620260)0.999850:0.24853472,
(Sf-tarl2:0.49918619,((Aj-tarl1b:0.01137974,Ar-
tarl2:0.00786869)0.999850:0.44616302,((Lac-tarl2:0.29187657,To-
tarl2:0.05991528)0.999850:0.15417700,(El-tarl2:0.16937719,Ss-
tarl2:0.07597668)0.999850:0.14826643)0.999850:0.31692142)0.788585:0.08886947)0.9
99850:0.42984240)0.999850:0.11059132,(Rt-tarl1:0.50923367,((Xl-
tarl1:0.09910400,Xt-tarl1:0.10775413)0.999850:0.33285118,(Lo-tarl1:0.10323922,
(Sf-tarl1:0.12563762,((Aa-tarl1:0.00000029,(Ar-tarl1:0.00261749,Aj-
tarl1a:0.00259138)0.000000:0.00000029)0.999850:0.12027907,((Ch-tarl1:0.10262641,
(((Pp-tarl1:0.01858616,Lw-tarl1:0.03155365)0.999850:0.01436198,(Dr-
tarl1:0.04156523,((Cc-tarl1b:0.05632397,(Sr-tarl1b:0.00812572,Sa-
tarl1b:0.02616002)0.958536:0.00270398)0.979026:0.00787914,(Cc-tarl1a:0.00266708,
(Sr-tarl1a:0.00000016,(Sa-tarl1a:0.00533238,Sg-
tarl1:0.01067265)0.000000:0.00000016)0.999850:0.00266459)0.000000:0.00000057)0.9
66661:0.00586143)0.994466:0.01341841)0.999850:0.05853927,(Am-tarl1:0.03081778,
(Pyn-tarl1:0.04687452,Ip-
tarl1:0.09794416)0.826720:0.00821863)0.999850:0.02440495)0.996596:0.01749963)0.5
32334:0.01292080,((El-tarl1:0.13722932,(Ss-tarl1a:0.00927277,Ss-
tarl1b:0.06437863)0.999850:0.02658755)0.999850:0.08105330,((Gm-
tarl1a:0.00127420,Gm-tarl1b:0.42766721)0.999850:0.18518614,(Cs-tarl1:0.19091618,
(((Mom-tarl1:0.06765157,(Tn-tarl1:0.04186389,Tr-
tarl1:0.07625170)0.999850:0.07433674)0.999850:0.02487177,((Lc-
tarl1:0.02112798,Mim-tarl1:0.00000090)0.999850:0.02825699,(Dl-
tarl1:0.00783973,Ms-
tarl1:0.00511366)0.999850:0.00918174)0.279593:0.00100708)0.879229:0.00953156,
(Moa-tarl1:0.07080778,((Lac-tarl1:0.03037344,((Stp-tarl1:0.02971128,((Ac-
tarl1:0.02695655,(On-tarl1:0.00524976,(Mc-tarl1:0.00000033,(Nb-tarl1:0.00000013,
(Pun-tarl1:0.00000001,(Hb-tarl1:0.00000001,Mz-
tarl1:0.00000001)0.000000:0.00000001)0.999850:0.00262868)0.999850:0.00264770)0.0
00000:0.00000014)0.999850:0.01458179)0.999850:0.04581122,(Ol-tarl1:0.09434025,
(((Cn-tarl1:0.00530484,Cv-tarl1:0.00521121)0.999850:0.04867995,(Fh-
tarl1:0.03502035,((Pf-tarl1:0.00000001,Pl-tarl1:0.00000001)0.997502:0.00267535,
(Pom-tarl1:0.00000008,(Pr-tarl1:0.01357552,(Xc-tarl1:0.00000020,Xm-
tarl1:0.00535488)0.999850:0.01090505)0.998810:0.00537351)0.000000:0.00000008)0.9
99850:0.02757681)0.999850:0.01472702)0.999850:0.04587902,(Nf-tarl1:0.11003680,
(Al-tarl1:0.04784582,Km-
tarl1:0.03982676)0.987146:0.00895903)0.994477:0.01576753)0.999850:0.03594556)0.9
96106:0.01527418)0.936378:0.00416069)0.999850:0.01098941,(Hc-tarl1:0.12617853,
(To-tarl1:0.03909135,Pa-
tarl1:0.04788713)0.930459:0.00584203)0.998936:0.01260964)0.999850:0.00560543)0.2
59426:0.00086413,((Po-tarl1:0.01189317,(Py-tarl1:0.02412886,((Bp-
tarl1:0.02920724,Sh-tarl1:0.04842456)0.999850:0.04217209,(Pem-
tarl1:0.01728817,Ps-
tarl1:0.00603865)0.981887:0.02646949)0.999850:0.30504727)0.422686:0.01350496)0.9
99850:0.03716897,((Cr-tarl1:0.01990385,(Ga-tarl1:0.06524715,Af-
tarl1:0.03384383)0.984652:0.00556149)0.999850:0.01158457,(Lb-tarl1:0.07349284,
(Sea-tarl1:0.00255684,((Sem-tarl1:0.00000001,Ser-
tarl1:0.00000001)0.000000:0.00000001,(Ses-tarl1:0.00000001,Sn-
tarl1:0.00000001)0.000000:0.00000001)0.992038:0.00262522)0.999850:0.02345040)0.7
88387:0.00291703)0.998026:0.00537921)0.903680:0.00269475)0.978453:0.00649630)0.9
97087:0.00854861)0.966276:0.02031550)0.999850:0.06799505)0.999850:0.03122851)0.9
99850:0.05678853)0.999850:0.10555999)0.308116:0.01911131)0.999850:0.12027883)0.9
99850:0.14090067)0.872587:0.06596837)0.999850:0.18434663)0.999850:0.10044485)0.9
97197:0.08460709)0.999850:0.18657277)0.999850:0.29189112)0.939191:0.07364608)0.7
29914:0.07585926)0.988627:0.08820500)0.983243:0.08872837,((Bbe-
adra1a:0.64679315,((Lec-adra1a:0.23458074,(Mm-adra1b:0.23533049,Le-
adra1b:0.19923495)0.999850:0.06357804)0.911721:0.04397243,(Mm-
adra1a:0.23300679,Cm-
adra1a:0.24326976)0.999850:0.11099775)0.999850:0.36898840)0.999850:0.41588493,
((((Cin-htr7:0.00000395,Csa-htr7:0.04612367)0.999850:0.68010327,(Mm-htr7:0.
10076905,Dr-htr7:0.15283205)0.999850:0.47019512)0.999850:0.40484244,((csa-
htr5:0.25016607,Cin-htr5:0.12237950)0.999850:0.86712258,((Dr-htr5ab:0.05212589,
(Lec-htr5:0.35410227,Mm-
htr5A:0.20915757)0.544329:0.03384083)0.999850:1.04410697,((Pm-

htr1A:0.19502890,Dr-htr1a:0.14727096)0.999850:0.46560421,((Dr-htr1fb:0.38708825,
(Rt-htr1:0.20752853,Le-htr1:0.41538056)0.999850:0.25324809)0.999850:0.33727289,
(Cm-htr1:0.57417121,(Mm-htr1b:0.16449281,(Rt-htr1b:0.09488161,Cm-
htr1:0.08615613)0.999850:0.07376908)0.999850:0.18173144)0.999194:0.13595500)0.99
9850:0.28573477)0.884827:0.07835355)0.816981:0.10703021)0.996015:0.12455004)0.48
5399:0.10806823,((Bfl-adra2:0.70551685,(Cin-adra2:1.17609312,(Lec-
adra2:0.32341982,(Mm-adra2a:0.24795619,Rt-
adra2a:0.32128957)0.999850:0.16394205)0.999850:0.29020162)0.999850:0.26229161)0.
999850:0.21639601,(((Mm-drd2:0.06640658,Dr-drd2:0.11280664)0.999850:0.13877443,
(Dr-drd3:0.16357002,Mm-drd3:0.24148729)0.999850:0.12045762)0.999850:0.38127885,
(Bfl-drd4:0.74278694,(Mm-drd4:0.39720084,Dr-
drd4a:0.30691641)0.999850:0.43839955)0.999850:0.19418636)0.999850:0.25689291)0.9
99850:0.16094289)0.969684:0.06486368)0.994392:0.09246650)0.999850:0.29462271)0.9
99850:0.22886504)0.999850:0.94884052)0.921125:0.08144813);

SUPPLEMENTARY INFORMATION
SI File 2: Amino acid sequences used in phylogenetic trees

Olfactory function in the trace amine-associated receptor family (TAARs) evolved twice independently

Milan Dieris[1], Daniel Kowatschew[1], and Sigrun I. Korsching[1]*

[1]Institute for Genetics, University at Cologne, Germany
*Corresponding author, sigrun.korsching@uni-koeln.de

Amino acid sequences of *taar* and *taar*-like genes (*tarl*) from jawless, cartilaginous and bony fish (for full species names see SI Table 1)

Table of Contents

TAAR-like receptors (TARL)....2
 Jawless fish TARL....2
 Cartilaginous fish and tetrapod TARL....18
 Bony fish TARL....19
TAAR receptors class II from bony fish and cartilaginous fish species....34
 Cartilaginous fish class II TAAR....34
 Bony fish class II TAAR....35
Reference TAAR sequences from all classes (class I, II, III) in several bony vertebrate species (zebrafish, stickleback, fugu and mouse)....69
Aminergic receptors used as outgroup (species names, gene IDs: Suppl. Table 1)....77
Deuterostome serotonergic receptor 4....97

Most *taar* and *tarl* genes are monoexonic. Intron gains occurred rarely, and are indicated here by alternating blue and black color for the exons, with amino acids from codons spanning two exons in red. Frame shifts and stop codons are indicated by exclamation marks and asterisks, respectively.

TAAR-like receptors (TARL)

Jawless fish TARL

Lethenteron camtschaticum

>Lec-tarl1a

MDTPTSSVAAPSSPASSNSSWEAPPPLPHCALVQGSFSCPGSALSSAQRAAWLATMSIAVASAVLGN
LLVVASVLHFRRLQTRTNAFAASLAVADLLVGLLVMPFKMTRSAYGCWFYGTAFCAAHTCLDIALCT
ASILHLACIAFDRHVAVCDPLRYAQRVTARHVTAMVALSWCCAAVISVVPVSLGWNVIGVPDEVVAA
SCADSCDFLLGAPYAVGSSVISFFGPAAFVVVAYARILREARRQGRAIACEQRRHQRQQEKQQQNED
QQQGRQEDTAGEGGLGGARGEMTGSEGIIGKGDGIGGIDERIGGRAEGIGTVGKIGSVFDGGRPTAA
AGKSIWAGRAADAKSERNATKTLSIVVGVFLASWLPFFLVNVSDPLLGYSIDPRVWEAVTWLGYANS
AANPVIYGIFSPNFRAAFRAIAARSAFRAGSRDAQLGF*

>Lec-tarl1b

MDPSTSSDAAPSSPASSNSSWEAPPPLPHCALVQGSFSCHGSALSSAQRAACLAMMSVAVASAVLGN
LLVVASVLHFRRLQTRTNAFTASLAVADLLVGLLVMPFKMTRSAYGCWFYGTAFCVAHTCLDIALCT
ASILHLTCIAFDRHVAVCDPLRYAQRVAARHVTAMVALSWCCGAVISVVTVSLGRNVIGVPDEVVAA
SCADSCDFLLGAPYAVGSSVISFFGPAAFVVVAYARILREARRQGRAIACEQRRHQRQQEKQQQNED
HQQGRQEDRAGEGGLGGARGEMTGSEGIIGKGDGIGGI!
ERRGGRAEGIGTVGKIGSVHEGGRPTTAAAGKSTRTVGRAVDAKSEHNATKTLSIVVGVFLASWLPF
FLVNVSDPLLGYSIDPRAWEAVTWLGYANSAANPVIYGIFSPNFRAAFRAIAVRSTFRAGSRDAQLG
F*

>Lec-tarl1c

MDTPSSSAAAPPSPASSNSSWETPPPLPHCALVQGSFSCRGSALSSAQRAACLATMSVAMASAVLGN
LLVIASVLHFRRLQTRTNAFAASLAVADLLVGLLVMPFKMTRSAYGCWFYGTAFCVAHTCLDIALCT
ASILHLACIAFDRHVAVCDPLRYAQRVAARHVTAMVALSWCCGAVISVVTVSLGWNVIGVPDEVVAA
SCADSCDFLLGAPYAVGSSVISFFGPAAFVVVAYAQILREARRQGRAIACEQQRHQRQQGKQQQQQN
EDQQQGRQEDRAGGGRGGARREITGSGGIVGKRDEIGGLEERRGGRVEGVGTVGKIGLVLEEGIPTA
AAGKSTRAVGRAVDAKSERNATKTLSIVVGVFLASWLPFFLMNVSDPLLGYSIDLRAWEAVTWLGYA
NSAANPVIYGMFSPNFRAAFRAIAARSTFRAGSRDAQLGF*

>Lec-tarl2a

VTFPAMALLIATWNASSPNDVLARYYGRTFCKAHTCLEYVFTTASILHLGAIAFDRHTAICDPLRYR
QRITPRRVAHLLAASWLLPFLYVPPVSLGWNVVGVEEAARRLSCPDSCVVLKNVGFALVDTCCAFFA
PTALMLVAYARIYRVARRQARQIAASSTGTVSVVAASVKQQQQQQQQNNLQQQGNEQQATFRRTMR
REHNATKTLAIILGAFVVCWCPYFVASAVDPFVGFSTEPTLLSATLWLCYANSALNPVLYAAFNRRF
RAAFSRIFCRGKGNAAVVVMVAAAWRDRRRPRPPPPACLEEESDS*

>Lec-tarl2b

SSLTPSPLHPPLTPVLSPHPPLSHPPPLSLTRRYTARVTSGTVAAMLVACWLGPLLYVAPIMLGLQS
VGAEALLALACPDDCPFVMSATFSLAATSCSFVAPMIIMLAAYARIYRVARRHARSIALRPPAPPGG

SDGAKGMAAASSSSSAPRRWRQEYNATKTLGVILGAFFALWLPFFSAAVADGLPGGPRVDGTTWHCV
TWLGYVNSAVNPLLYASLNRAFRRAFGILFSPRELAKGRRNADFTD*

>Lec-tarl3a

RQLQTRTNAFTLSLAVADLLVGLLVMPFSATRSLYGCWFFGATFCKVHTCMDFLFTTSSIFHLCAIA
FDRHVAICDPLRYGARVSRRTVALLVALCWLVALLYVVPIMLGWNLVGIEEEVARSSCPGNCDLYLN
TAFALTSAACGYYAPMVGMVVAYARVYSVARAQSRRVRAEANRDRGV**D**ERAGGKRKARTSLKREHNA
TKTLGIIMGVFLVLWLPYYASSIASAFSASSAIDPVAWDAFFWMGYSNSAINPVLYACFNRSFRAAF
RLLFSRRAFKASTRRLDLSES*

>Lec-tarl3b

RQLQTRTNAFTLSLAVADLLVGLLVMPFSATRSLYGCWFFGRIFCKVHTCLDVLLSTASIAHLGAIA
FDRHTAICDPLRYHQRLTANRAATLVALCWVGSALLAVGPIMLGWNTVGIEELVASLSCPDACPFFM
NAPFAIAGSTCSFFGPSFIMVVTYMRIYRVALRQARAVRNVVSVASVARQRCDIDSTPEQGRDQRRE
RGATKTLGVIMGTFVICWLPFFSLTLLDPFTGFLAEPWVWEAATWLGYVNSALNPILYPAFNRTFRR
AFYLVFTCAWRSRNVRRSDLAE*

>Lec-tarl3c

RQLQTRTNAFTLSLAVADLLVGLLVMPFSATRSLYGCWFFGRIFCKVHTCLDILLSTASIAHLGAIA
FDRHTAICDPLRYHQRLTAGRAAALVALCWVGSALLAVGPILLGWNTVGIEELVASSCPDACVLLMN
APFAIAAATYCFFAPSFVMVVTYMRIYRVALRQARAVRNVVPVSSVAWEHCDINSRPEKKTDQRRER
SATKTLGIIMGAFVTCWLPYFSLTLLDPFTGFLAEPWVWESTAWLAYINSALNPILYPAFNRAFRQA
FRLVFTCAWRSRNVRRANLA*

>Lec-tarl4a

MYMGKYASPALALNSSVQNGTFQSHCVLTQFNLVCRQPTVPPEYAEVILIAMIISIILNMFGNTLII
AAILYFKQLQVLPNAFTFSLSVADILVGVVVMPFYMNQCVYNCWFYGHAFCKVHHFLHAFLPTASTM
HLCCIGYDRYLAICDPFHYQERFTRRTAAIALAATWLGSLLYVVPIMLDWIVIGIEEMVAERTCPDN
CIFFMNKVFSSCGAFFSFMLPMGIMTVTYAKIYRIARKQARSIGAQQHGMLGQTALGFNKQQWAAMK
REHNATKTVSIILGIFMICWAPFFSTIVIDPYLNYTTKPVVWEMLNWIGHVNSAINPFLYGGFNRTF
RNAIRIIFSRRLLQPGIRSAEL*

>Lec-tarl4b

MNSSLALGLMDGDPGHNASHVEPLLCLIRESGLRCRPARLSARTANIIFIAMIFSMVLNFLGNMTII
TAISYFKQLQVLPNTFTFSLAVADIMVGVVVMPFYMTQCVYDCWFYSHAFCKVHHFLDAFISTASTM
HLCCIAYDRYLAICDPMHYRERFTWKTAALLLAAAWLGSLLYVVPVMLGWIVIGIEDMMASRTCPDN
CVFYMNKVFSSCGAFFSFVVPMTIMTLAYVKIYRVARKQARSISVQQQGSQGKSGFNKQQWAAMKRE
HNATKTVSIILGVFMFCWAPFFSTIVNDPWIDFKTNSIFWDIVNWFGHVNSAINPYLYGGFNRTFRN
AIRIMFSRKFWQPGSRSAEL*

>Lec-tarl4c

MNSSLALGLMGGDPDHNASNVEPLICLIRESGLRCRPARMSARIANIVLIIMIVTTVLNVLGNMTII
MAISYFKQLQVLPNTFTFSLAVADIMVGVVVMPFYTTQCVYDCWFFGQKFCKVHHFLDAFMCTASTW
HLCCIAYERYLAICDPMHYRQRFTWKTAALLLATAWLGTVLYVVPVMLGWIVIGIEDMMASRTCTDN
CIFYMNKPFSSCGAVITFMVPTTVMTLAYAKIYRVARKQARSISIQQQGSQGKSGFNKQQWAAMKRE

HNATKTVSIILGVFIFCYAPFFYTIVKDAWIDFRTNSFTWDMVNFFGHVNSAINPYLYGGFNRAFRN
AIRIMFSRKFWQPGSRSAEL*

>Lec-tarl4d

MNSSLALGLMDGDPDHNASNVEPLICLIRESGLLCRPARMSARIANIILLIMIVTMVLNVLGNMTII
MAISYFKQLQVLPNTFTLSLAVADIMVGVVVMPFYTTQCVYDCWFYGQTFCKVHHFLDAFVCTASTW
HLCCIAYERYLAICDPMHYRERFTWKTAALLLVTIWLGTVFQVVPVMLGWIVIGIEDLMASRICKNN
CIFYMNKTLSSCGAFLTFVVPVTVMTLAYAKIYRVARKQARSISIQQQGSQGKSGFNKQQWAAMKRE
HNATKTVSIILGVFIFCYAPFFYTIVKDPWIDFRTNSFTWDMVNWFGHVNSAINPYLYGGFNRAFRN
AIRIMFSRKFWQPGSRSAEL*

>Lec-tarl4e

MAVWLATSSVLHLSSIAYDRYVAICQPLRHAERASRGRTAVMPLGRWFVVMHHNAPIVMGLMPPDIE
DAVASASCPDKCVVHMNANFGVSDALYCFVATMSVMGVARRRARSVHGAATSKSCGPKASGGTITVQ
WASLKREHNATKTLAIYIFWLPYFSVGTVEPFKDFTMNANTWELVVWPSYTNSTINPLLYA

>Lec-tarl5a

MNSTLIKCIITYYNLNCTTPHLSYTHGLAVQGVLLFFIISTILGNMLVIASIMYFKQLQSHTNSLTL
SLAAADLLVGLVVMPFSMMRTVHSCWFYGALFCKLHCSLDYIFSTVSILHLSCVAFDRYIAICDPLH
YTSRVTNNTVAFMLIICWTCPLVYFAPFLLGWNIIGIEEMVQSRICADTCAIVKNVWFAYVDTCCAF
ITPMAIMITTYAKIYRVARRQARQVSAASVALQLNGHERQHQWALMKREHNATKTLGIIMGGFLFFW
LPNFVASLIDPVNDFQTDPLVWNIVVWLGYINSTINPLLYASFNRPFRKAFKIMFSLEITHLSTRNK
DLD*

>Lec-tarl5b

MAQSVLKLNSTNLTNVSSNRCVLNAFQLLCPPLDLSYASSVLVQTALVICIVAIAVGNTLVIVSIAY
FRKLQSRMNVFTLSLALTDFLVGVLIMPFSMLKTVHNNCWFFARWFCKIHTSLDYVICTVSILHISC
IAYDRYNAICHPLCYTQRVNRAKVVVVLGGCWVAPVIYVAPIMLGWNIIGIEQIVASMTCPDSCVMF
KNIPFMVMDTVCAVILPMILMGVAYAKIYRVVRDHSRRIHSQHQQQVDGNALKREHNATKTLGLIMG
AFCVCWLPYCVVNVVDVFNNYQTSRWVWSVVTWLGYINSGFNPVLYALFSRPFRRAFAIVFSKRIFT
SDTRNMDLFRED*

>Lec-tarl5c

MAQSTMEPNFTNIANLTNTSSGSCVLNAFQLMCPPPEMSYASSVIVQTALVICIVAIAVGNTLVIAS
IAYFRELQSRMNVFTLSLALADFLVGVLIMPFSMLKTVHNCWFFGRWFCKIHTSLDYIFSTASIVHI
SCIAYDRYNAICHPLCYTQRVNRVKVVVMLGGCWVAPVIYVAPIMLGWNIIGIEEIVASMTCPDSCV
MFKNTTFVILDTVCAFMVPMSIMGVAYGKIYKVARDQSRRVHSQHQQQVDGNALKREHNATKTLGLI
MGAFFLCWLLYIVVSLVDVFVHFQTSGEVWSVVVWLGYINSGFNPVLYALFNRPFRRAFAIIISKRI
FTSDKRNMDLSRED*

>Lec-tarl5d

ATSVALQVLFVLFMAGTVVGNALVIAAVSLFGHLRTRTNAFTLSLAAVDALIALVVMPFSSTRTVRG
GCWAYGGLFCKLHHALDYSLCTSSIVHLSCIAFDRYTAICHPLRYQALVNRRSVAAMLAFAWLGPTF
YVVPFLLHWNTVGIEAMVVARTCPGDCVLYKNVGFVFLDTTCAFFVPMGVMIVAYAKIYRVARRQAR
QIHASRAQVTVRGWVSG*

>Lec-tarl6a

MENGTVGPYDHCIDSFYHLNCSTPTLSVPERTVALSAITTLILATILGNLLIITSIAYFRQLQTRTN
IMALSLAVADLLVGLTVMPYSMMKAVYKCWFYGQFFCNLQFFLDYTLTNSSIIHLGCIAYDRYVAIC
DPLRYSQRVTNHTVVIMLLISWFAPALFSSPILVNFNPEWSRDIIEIISCPNQCLFFVSTWLVSVVG
VCPYVLSLLTMSYVYARIYVVARRQGHQISSVSLQVHAQQQQQQVEHTNFRQKSTAMKREHSAAKT
LGSIIGFYLLSWLPFYMVVLFFPNFKNSSAAVRITTWIGYISSAINPVLYASLNRPFRSAFVAVISC
KVLSSSRARTMDLSGVK*

>Lec-tarl6b

MENRTVGPYDHCIDSFYHLNCSSPTFSMSERTAALSAITTLILATVLGNLLIITSIAYFRQLQTRTN
IMALSLAVADLLVGLTVMPYSMMKAVYKCWFYGQFFCNLQYFLDYMLTNSSIMHLGCIAYDRYVAIC
DPLRYSQRVTNHTVVTMLLISWFGPALFSSPILVNFNPEWSRGIIEIISCPNQCLFFVSTWLVSVVG
VCPYVLSLLTMSYVYARIYVVARRQGRQISSVSLQVHAQQQQQQQQAEPTNIRQKWAAMKREHSAA
KTLGSIIGVYLLSWLPFYMVVLFFPNFQNSSAAVRITTWIGYISSAINPVLYATLNRPFRSAFVAVI
SCKVLSSTRARTMDLSGVK*

>Lec-tarl6c

MCKKNATGETKAAGRGAGPPTPSCAFSEPQRGDLFLVLSVVNLHVSLHRSSRSKAMPNTTTTITARQ
CIDFHGYNCTGPSLPEHSRPVVLVLISIFITATILSNLLIISSVAFFRQLQTHTNILALSLAVSDFG
IGALVMPLRMVGTVYNCWYYDYSFCNVFYFVDYTLCNSSIFHLSCIAYDRYVAICDPLRYTQRVTDR
TLVAMLLISWLGAALFTSPSLLSFSPQLAPSSIEREGCPDDCLFNVDVALHFFYGASPYFITMLVIM
AVYARIYHVARRQARQIAASSSTKVQQSGGDGNEQQRVSNMRREHNATVTLGSIIAVFFLSWLPFFI
IVLVFPFVGNFDVEYEVATWMGYISSAVNPLLYASFNRQFRGAFRKILSLTMFRAGARNAKLSE*

>Lec-tarl6d

MANTTKTLQCIDYHGYNCSGPSLPEKSRPIILIIISFFIVVTILSNMLIISSVIIFRQLQTHTNILA
MSLAVSDFGLGSIVMPLRMMGTVYNCWYYDYSFCNLLYFVDYTLCNSSIFHLSCIAYDRYVAICDPL
RYAQRVTKRTLVAMLLICWFGAALFTSPSLLSFSPQLAPSSIERVGCPDDCLFNVDIALHFFYGAFP
YFLTMLVITAMYARIYHVARTQARQIKAATTKVQSQGQQRVRNMRREHNATVTLGSIIVSFFLSWMP
FFIIVLVFPFVGNCDVEYEVATWMGYISSAVNPLLYASFNRQFRGAFRKILSLRIFRAGERNFKLSE
*

>Lec-tarl6e

MANTTKTLQCIDYHGYNCSGPSLPEKSRPIILIIISFLIVVTIFSNMLIISSVIIFRQLQTHTNILA
MSLAVSDFGLGSIVMPLRMVGTVYHCWYYDYSFCNVLYFVDYTLCNSSIFHLSCIAYDRYVAICDPL
RYAQRVTKRTLVAMLLICWFGAALFTSPSLLSFSPQLVPSAIKRVGCPDDCLFSVDVSLHFFYGACP
YFLTMLVITAMYARIYHVARTQARQIKAATTKVQSQGLQRVSNMRREHNATVTLGSIIVAFFLSWMP
FFIIVLVFPFVGNCDVEYEVATWMGYVSSAVNPLLYASFNRQFRGAFRKILSLRIFRAGERNFQLSE
*

>Lec-tarl6f

VLYFVDYTLCTSSIFHLSCIAFDRYVAICDPLRYAQRITKRTLVAMLLICWFGAALFTSPSLLSFSP
QLVPSAIKRVGCPDDCLFSVDVTLHFFYGACPYFLTMLVITAVYAHIYHVARTQARQIKAATTKVQS

QGQQRVSNMRREHNATVTLGSIIGAFFLSWLPFYIIVLVFPFVGNCDVEYEVATWMGYISSAINPLL
YASFNRQFRGAFRKILSLRIFRAGERNFKLSE*

>Lec-tarl6g

MANTTKTLQCIDYHGYNCSGTSLPEKSRPIILIIISFFIVVTILSNMLIISSVIIFRQLQTHTNILA
MSLAVSDFGLGSIVMPLRMVGTVYNCWFYDYSFCNVLYFVDYTLCTSSIFHLSCIAFDRYVAICDPL
RYAQRITKRTLVAMLLICWFGAALFTSPSLLSFSPQLIPSAIKRVGCPDDCLFSVDITLHFFYGACP
YFLTMLVITAMYARIYHVARTQARQIKAATTKVQSQGQQRVSNIRREHNATVTLGSIIGAFFLSWLP
FYIIVLVFPFVGDCDVEYEVATWMGYISSAVNPLLYASFNRQFRGAFRKILSLRIFRAGERNFKLSE
*

>Lec-tarl6h

MDNSTTGSPCSLHYKNYNCTGPSLSEEDRIFTLAIIIFFIIATILGNLLIIVAIACFRQLQTHANTL
ALSLAVSDFLIGLLIMPYRMMHAVYQCWFYASFFCHLHYFLDYTLTTSSIIHLSCIAYDRYVAICDP
LRYPTRVTDRTVAVMLALCWLGAAIFTTPALLSLSPTLSRGTIERGGCPDNCMFIVNLGLHFAYGMC
PYFLSMFIILLIYSKIYRVARRQARKVIAGAPTESQRTEVANGGSSGGGGGGDEAAKRWKSRMKREH
NAAKTLGSIIACFMLSWLPYDILVIVFPFYKNTSVAYEVVCWIGYISSTVNPILYASINRPFRTAFG
RILRLDFFSAEVRHLELSNH*

>Lec-tarl6i

MDNSTTVSPCSLHYEYYNCTGPSLSEEDRIFTLAIIIFFIIATILGNLLIIVAIACFRQLQTHANTL
ALSLAVSDLFIGLLIMPFSMMHAIYQCWFYASFFCHLHYFLDYTLTTSSIIHLGCIAYDRYVAICDP
LRYPTRVTDRTVAVMLALCWLGAAIFTTPTLLSLSPTLSRGTIERGGCPDSCMFTVDLGLHFAYGMC
PYFLSMFIILLIYAKIYRVARRQARRVTAGVPIHAQRVEVANGGSSGGGHDGDEAAKRWKSTMKREH
NAAKTLGSIIGCFMLSWLPYYILVIVFPFYKNTSTAYEVVSWIGYISSTVNPILYASINRPFRTAFG
RILRLDFFSAEARHLELSNH*

>Lec-tarl6j

MDNSTTGSPCSLHYEYYNCTGPSLSEEDRILALAIIIFFIIATILSNLLIIVAIGFFRQLQTYANIL
ALSLAVSDLLIGLLIMPFSMMHAVYECWFYASFFCHLHYFLDYTLTTSSSIHLGCIAYDRYVAICDP
LRYRTRVTNRTVAVMLVLCWIGAAILTTPTLLSLSPTLSRGTIERGGCPDSCMFTVDVGLHFAYGIC
PYFLSMFIILLIYAKIYRVARRQARRVTAGMPIHAQTVEVANGGSSGGSDGDELARRWKSTMKREHN
AAKTLGSIIACFMLSWLPYDIMVMVFPFYKNTSAVYEVMSWIGYISSTINPILYASINRPFRTAFGR
ILYLDVFSSDARHLELFEH*

>Lec-tarl6k

MYNNTDNRCAELYHGYNCTGPSLTPTNLCAVVILLSSFIVATVLGNLLIICSIAFFKQLQTFSTTLA
FSLALSDFLVGLVIMPFAVMKTAYRCWFCAGAFCNAHHFLDYMFTNSSIFHITCIAFERYVAISDPL
RYGARVTRKTMAAMLLLCWLGSALFSSPSLVSLLSKAVSGGLIQRVRCPDDCVFSVYIGIQAVIGIS
PYFISLVVIVLVYGRIYSIARRQAQKVNSEGSRRRGQEAAASTDTLHKWRAMRREHNATKTLGVIIF
FFLLSWLPYNVTVIVFEFVAYASDVYQVTLWIGYISSAFNPILYACFNRPFRTAFHRILRLKVFTLA
ERDFALSTVQDGGH*

>Lec-tarl6l

MNHTSAPSARNACVTFRSFNCTGPSLPENYRALLLAILAFFTLATILGNLLIISSIAFFRQLQSFPN
VLALSLAISDFLVGLVIMPLAVTKVIYNCWFYARLYCNVHYFLDFAFTNCSILHLCSIALNRYVAIS
DPLRYESRVTRRTLVVMLVTCWLGSAVSSSPILLSLSPTLSVGTIERISCPNDCVFDINIGIMVVIG
YCPYFASLVVIVCIYGKIYRIARAQVIKIRAQQGGGGGGGSHRTDNDNAEALVKLRTMKREHSATKT
LGVIIGFFLISWLPYYVIVLASLALTDTLFAYRVALWIGYISSAFNPILYAAFNRPFRNAFRLMFRL
KVFGEGARDTDLSGRLK*

>Lec-tarl6m

MANLTDTHASPGCIHYLDAFNCTGPTMSLPALSASLAIISFFVVATVLGNLLIMSTIAFFKQLQTST
NVLALSLAVTDFLIGLIIMPMAMTKEMFNCWFYGDVFCNVHFFVDYMLTNCSMLHLGSIALQRYVAV
SDPMHYATRMTSRSLAAIIGFCWLGSAVFSSPILLSLSPALSEQTVARKSCADNCLFYVHMGIMFVV
GYCPYFASLLVIVCVYAKIYRIARNQMRKIRSVACKVEETTRQVIKREHNATKTLGVIIFFFLLSWL
PYYLIVLSSMAVKNTMSAYRVALWAGYISSAFNPLLYAFFNLHFRNAFKMMLGCKIFAPGARHTDLN
FLNKATNDRN*

>Lec-tarl6n

MDNSSLASSPSCVISFSNLNCTPTSLKSKHDRAVAVFTISLFIVITILSNLLIVSAVALFRQLQTQA
NALALSLAVSDLLVGVVIMPLSLTKSVYKCWFYAPLLCHVHFFCDYWFTTASVLHLSCIAYDRYVAI
CDPLRYQQRMNRRALVVMLLFCWLCSALLSTPILLSLSPTLSQGTIAKNGCPDDCRFFISVGILFAI
GMWPYFTSVLLVVLMYGRIYRVARGQARKIAAMDRASSGLGGGDEAGRAAASRWASMKREHSAAKTL
GAILAAFIISWLPYYIIAIMLTVDESVILPYKVSVWLGYMNSAINPILYAAFNTQFRRAFKAILTFN
TYPSRAADDEMSSTKN*

>Lec-tarl6o

MENTTSTSTSKCVISYYGLNCTKMDFSKSESALIVGVMSFFIGTTILSNLLIISAVAFFRQLQTRTN
TLALSLAVSDLLVGVVIMPLAMIKAIYKCWFYVQWFCNVQFFCDYWFTTASVLHLSCIAYDRYVAIC
DPLRYAQRVTRRTLTFMLLFCWIVSAIISTPILLSMSPTLAKGRIEMVGCPNDCRFLISVGILFTIG
MVPYFTSVLLIMLMYGRIYQVARSQARKIGTQSHGLPGVASETTSRWTNMKREHNATKTLGSIIAAF
IISWLPFYILAIVFTMYQDIFLPYKISFWIGYMNSTINPILYAVFNKQFRHAFKTMLSFKVFSSAAR
DKEMN*

>Lec-tarl6p

MAENSSSSSSSNSGAEGLSECVVSYYGLNCTKMNFSKSESALIVGIMSFFIAVTILSNLLIISAVAF
FRQLQTRANALALSLAFSDLLVGVVIMPLAMVKSVYKCWFYARWLCNVQFFWDYWFTTASVLHMSCI
AYDRYVAICDPLRYQQRVTRRTLTFMLFFCWIASALLSTPILLSMSPTLSKGRIETVGCPDDCSFLI
SVGILFAIGMWPYFMSVLLIVLMYGRIYRVARGQARKIAAMDRASSVLGGGEEAGRAAASRWASMKR
EHNATKTLGAIIVAFIISWLPFYINAVIFTMDESVLLAVWIGYMNSAINPILYATFNKQFRHAFKEI
LSLNVFLSPADRNKEMQ*

>Lec-tarl6q

MAENSSSSSSSNSGAEGLSECVVSYYGLNCTEMNFSKSESALIVGIMSFFIAVTILSNLLIISAVAF
FRQLQTRANALALSLAFSDLLVGVVIMPLAMVKSVYKCWFYARWLCNVQFFWDYWFTTASVLHLSCI
AYDRYVAICDPLRYQQRMNRRALVVMLLFCWIASALLSTPILLSMSPTLSKGRIETVGCPDDCRFLI
SVGILFAIGMWPYFTSVLLIVLMYGRIYRVARGQARKIAAMDRASSGLGGGEEAGRAAASRWASMKR

EHNATKTLGAIIAAFIISWLPFYIIAVIFTMDESVLLAYKVAVWIGYMNSAINPILYATFNKQFRHA
FKEILSLNVFLSPADRNKEMQ*

>Lec-tarl6r

MQNTSVPQVHKECVIFADFNCTGPTQVREVRLVLLVVFAIFITATILGNLLIITAIASFTKLQTHAN
FLALSLAVSDLLVGVFIMPLSMMKTVYDCWFYASVLCNAHYFLDYTLTTATILHITCIAYDRYVAIC
DPLRYPTRVTVRTIAGLLVLCWLGAAIFSSPILLSFSPTLSRGNIERASCPDDCVFSVSFGILVTIG
MGPYFTAIHVVLLMYAVIYRVARRQSRKVAAEAASSSAADSSAVKNMKREHSAAKTLGAIIGTFMLS
WLPYYVVAMMSFTVDGLFIPYRVAMWIGYCSSAINPLLYASFNRPFRTAFRDIFRLRVFSGVVRRDS
GLSERSHGQPMAR*

>Lec-tarl6s

MPNDSDDAASSTCVSFNHLNCTGPSLPPLSRHLVLALLLIFIAATILGNLLIIAAIASFRQLQTRAN
ALALSLAVSDLLVGVLCMPSSTLKTIYNCWFFPDVLCHWHFFADYMFTTASVLHVTCIAFDRYVAIT
DPLRYAARVTPRTLVAMLVFCWLGSALVSSPILLSLSNGDALSGPTIHRILCPNDCFFYISIGIMLV
IGMGPYFASMLVVVAVYARIYAVARGQARKIAASASGSQMRGHADESANSASVKSMKREHNATKTLG
AIICSFLLSWLPFYIITIISMFVNDMFLSYRITLWLGYISSIVNPFLYALLNRQFRAAFRTLLSVKV
FSVLGSRGADLMGVKKS*

>Lec-tarl6t

MANSSCTISFYDLNCTGPSLSVRDRAIVLTLVALVNATTVAGNLLLIATVSFFRRLRSWPNVMALSL
AVSDLLVGTLVMPLATVKAVYSCWFYADLLCHWHFFLDFALTTCSILHVACIAYDRYVAVCDPLRYA
ARVTERTVGAMLALCWLGSALVSAPILFSLSPALSPNTIHRILCPDDCFFYVHGLIIFVIQMGPYIV
SVAFVVALYGQIYRAARLQARRIGGVGGGGKSSSAEATAAARSEHKATKQLGIIIGCFLLSCLPFYL
VDVVSLVDEALFVPFRITVLSGYISSALNPLLFAKFNRQLRAGFAMVLRAQLFRPGARDTDFSGGGR
RGE*

>Lec-tarl6u

MTYSSLNNTTGHCVELFENQICSKPKLSYEDRIVAIAVLSFFISATIFGNCLIITAIAFFRQLQTNT
NALALSLAVSDLLVGAIIMPFSVNRSVYQCWFHGRVFCKVHFFLDYAICNASILCLGCIAFDRHVAI
CDPLRYAQRVTRGTLASMIVLIWVGAALLSAPILLSLSETWAKGIIEITGCLDGCHFAVHVILTLVV
GILPYFIIMLLMLLVYGRIYGVARRQSRQINNSSGSRASDADSRSKWAAMRREHNATLTLGMIVGFY
LVSWLPYFTASVLEMAFGISTSALVWATVTWFGYANSVVNPVLYATFNRPFRTAFRIIFSSELFAPG
ARRTDLHGLKVAGG*

>Lec-tarl6v

MTYTSSNNTTGHCVESFENLICSKPKLSDEDRIVAMAVLSFLISATIFGNCLIITTIAFFRQLQTNT
NALALSLAVSDLLVGAIVMPFSALRSVHQCWLHGHIFCRVHYYLDYTFCNASILCLGCIAFDRHVAI
CDPLRYTQRVTRGTLAFMLALIWAGAALLSAPTLVSLDETWARGLMEIAGCPDGCHFAMHVGLTLVV
GILPYFIIMLLMLLVYGRIYGVARRQSRQINNSSGSRASDADSRSKWVAMRREHNATLTLGMIVGFY
LVSWLPYFTASVLEMAFGVTASALVWSTVMWFGYANSVVNPILYATFNRPFRTAFRIIFSSELFAPG
ARDADLYGLKVTGG*

>Lec-tarl6w

MMEANSTLGECAAPFLQFNCTGPGLEPTDQALVLSILILFIAATIIGNLLIACAVLRFRQLQTRTNA
VTTSLAVADLLVGAVIIPCSMTHEVYRCWFFGQIFCRIHYFMDYWFTNASILHLGCIAFDRYVAICD
PLRYQQRVTNRTVALMLLMCWLCSALFSAPILVSLSDVLSNGVMDRTSCPDECVFIVNLGLTFVIGI
CPYFLSFIILSLAYAKIFRVARAQASQIHAAGKHGADSSSSASIVGDANSKRTWLAMKREHNATKTL
GMIIGFFLLSWLPFYIISVVDVIIDYQTNAVLRRTVTWVGYFSSAVNPVLYASFNRPFRTAFRDLVS
CRAFAVGARNKDLFGS*

>Lec-tarl6x

MPKTNSTLGKCAAPFLQLNCTGPGLEPTDQAFVLSILILLIVATIIGNLLVACAVLRFRELQTRTNA
VTISLAVADLMVGAVIIPCSMTHEIYRCWFFGQTFCRIHYFMDYWFTNASILHLGCIAFDRYVAICD
PLRYQQRVTNRTVALMLLMCWLCSALFSAPILVSLSDVLSRGVIERTSCPDECVFIVNFGLMFMIGI
CPYFLSLIILSLAYAKIFRVARAQASQIHAAGKHGADSSSSASIVGDANSKRTRLAMKREHNATKTL
GMIIGFFLLSWLPFYIISVMDVIIDYQTDAVMWGTVTWIGYFSSAVNPILYASFNRPFRNAFLDIVS
CRAFTCRRGT*

>Lec-tarl7a

MFWLHLRTSMDNQSDVEAPGLLCVNSYNNLSCSSPRLPATQRTLALVVLISFSACTVVGNSLIVLSI
LYFRQLQTRTNAFTLSLALADMLVGLLVMPYSMMRAVYSCWFYGSFFCKVHTWFDFTFTSSSVIHLS
CISIDRYIAISDPLRYPKRLGPP**KV**ALMLVVCWSTTLAYGLPFFSGWTVLGIEEVIARGSCRDACPV
VGNMAFAFTNAFFAYLIPLCIMVTSYAKIFRIAREQAHKIRANSAHDPGVESSSSSARQQWAAMKRE
HNATKTLGIIMGVFLLFWLPYCSVAVIDPIIGYQTSATSWDVANWIAYVNSAINPVLFASFNRSFRG
AFRLIFTCRVSSSTYRNADLFNLNDSAN*

>Lec-tarl7b

MSPPPDVPTLLSPRNQTTDANRSATTAAAASMSPSERLVALSVMTTLIACTVAGNLLVVAFIAYFRH
LQTRTNAFALSLALADFLVGLLIMPYSTMRSVDGCWPYGGVFCRVHTWLDFSLTTSSVVHLCCISYD
RYVAVADPLRYPQRVTRRAVALLLLCCWLSVPVYSWPVMSGWYSVGVPDGPGAFAGCREVCPVSVSA
TFGLANMLCAYAAPMLLMSLTYAKIYRLARAQARKIDAATAGLRRRGPADGAADGADGDGRRWKAAM
RREHNATKTLGVIAGVFVLCWVPYFVLSATDSLLDDATRRAAADISNWLTYVNSTLNPVLIVALNRS
FRNAFRATVACRVFAPGFRATDLFNYDDSER*

>Lec-tarl7c

MNPLPSFQLSPSPPPLLQLLLSTPTSSSSPPPPTPNSNHCVPSLYGLSCTPSDLSQTGRALLLTILF
LAILITLAGNLLIIASIAFFRQLQTRSNVLVASLAAADLLVGSLIMPFSAMRTADNCWPYGDLFCRL
HTWLDFSATCSSIINLACISAERYISVSDPLRYRQRVTPRVLIAMLTLSWSGLAVYGATFLAGWNVA
GMEGVIAAESCPHSCRVFMNAATTFANVGAYVAPMLVMAAANVKTYGVARDQARKVHASGPLDAHGR
SRWEAMKREHNATKTMGIIMGVFILLWLPYILLASTEPMLGYGTTPATWEAVNWLPYINSTVNPVLL
ASFNRSFNGAFRVILGGKATRPGARGTDLYNLRDRVG*

>Lec-tarl7d

MPFSAARTAYNCWFYGDLFCRLHTWLDFSTCTSSIANLACISLERYASIAEPLRYRQMVTPRVLAAM
LLLSWSGLPLYGATFMLGWNLVGIKRQVAESSCRHDCRVYMNPASTATNVLVAYVMPMLLMIVANAK
IHRLARAQARRIEGSAMVSGRDDRASTKREHNATRTLGIITGAFILLWMPYFILVASEPLLGYGTSR
AAWEVVCWLPYLNSAVNPVLLMVFNRSFNGAFRLALKGRVLKSGCRGIDLFNFRDGNM*

>Lec-tarl7e

MNESGSNDSSRRCVWTFHQFSCTSSDLSAAERATLLAILLSISAITVAGNLLTITSILYFRQLQTRT
NVLTLSLAVADLLVGLLIMPFSAMRSVHNCWFYGRVFCRVHTWLDYTFCTSSIVHLSCISFDRYVAI
SDPLRYGDRITHAALAAMLALSWISFPIDGLVFMLQWNLVGMEEETRRSCPDDCKVILNLPYAVANT
ACAFVLPMLLMALAYGRIYRLARVQSRKINSLGAQVQKMSGDAECLSKWNAMKRDHNATITLGIIIG
MFIAIWMPYFVVSATEPMVGYQAGPVAWEVINWFTYLNSTVNPILFAAFNRSFRWAFRLLVTCSAFR
PGIRSADLFNFKEGPA*

>Lec-tarl7f

MSSHANTTSPACGLTFYNFNCSRASVTSSSERAQMMSLLLSISAITVAGNLLTITSILYFRQLQTRT
NALTLSLAVADLLVGLLIMPFSVMRSVHNCWFYGRVFCRVHTWLDYTFCTSSIVHLSCISFDRYVAI
SDPLRYEQRVTRRACARMLVCSWLCLLVYGLAFMLEWNLVGLEEEVAHVCPDDCPVLLNLPFAMANT
IFACVVPMMLMTLAYGRIYQLARQQARKITSAAMGSNADSARSSLRREHSATITMGIIVGVFISLWI
PYFVVSVTEPIFGYQAGSVTWEVINWFTYINSTANPILFAAFNRPFRNAFYLILSGRMFASSCRGVD
LFDVPRALAKARG*

>Lec-tarl7g

METLNESIRNSSLLCVEVFHNFHCTSNDISEAERATLITIIIPAIAITIFGNLLTVVSILYFRQLQT
RTNVLTLFLAVADLLVGLLIMPFSAMRSVYNCWFYGWTFCKMHSWFDYTLCTLSVLLLSCISFDRYV
AISDPLRYHQRITNRTCALMLLFCWLCLPFYGVVFMMEWNLVGLEEEVAQICPDDCPVLLNLPFAMA
NTIFGCVVPMMLMTLAYGRIYQLARQQARKIASTAMGSNADSARSSLRREHSATITMGIIVGVFISL
WMPYFVVSTTESIFGYQASSLAWEFINWFTYINSTVNPILFAAFNRPFRNAFYLILSGRIFSSSYRG
VDLFNVQQGSKVNQGAKRCVSTLIGKKSDR*

>Lec-tarl7h

MLQETVQKVVTCNVSFYNLNCTLSELTQPQRVALICVFTIIITITVVGNILTIVSILYFRQLQTRTN
VLALSLALADFLVGCLIMPFSVMRTAYSCWFYGQLMCRIHTWLDYTFTTCSIFNLACISIDRYVAIS
DPLRYDQRVTYRILAVMLTICWSNIIPYGVSYMLKLNINGIESVVAAKSCPDNCYVFMNVPFGLANS
MGAYVLPMLFIMAAYSRIYVMARNQAKRISSLGDQVRASNASDLTMQSKWNAMKRDHNATKTLGMIM
VVLFIVWLPFIVVVAMEPVIGYRMDSTVWDVANWFTYFNSTMNPILFASFNNSFRNAFYLIMSGKIL
RGSYRGTDLFNFRRLEK*

>Lec-tarl7i

MKTHNATNTSVAPVCVVTFFNFNCSPPDLGDGERDILIGGFSLVIATTALGNILTITSILYFRQLQT
RTNAFAISLATADLLVGVMVMPYAVTRTAYTCWFYGKTFCKMHTWFDYTLTTSSILHLACISIDRYV
AISDPLRYEQRVTKKLVGRMLVFCWTSFIIYGLSYMLEWNIAGIEDIVASSTCPHNCPVFMNVQFAL
TNTLCAYVTPMLLMLAAYAKVYVMARAQARKISIAMLQTRSADAAVRSRWSAMKREHSATKTLGIIM
GAFVIFWVPFFFVAASEPLLGYASDPLVWDVANWFTYINSTMNPILFAAFNRSFRNAFYLIVSGKIL
RGSYRGTDLFSFKRGGGVAEMA*

>Lec-tarl7j

MSLHNESFPSVLCVVTFATFNCTPSPMTHVERALLMGTFTIAVAMTITGNMLTISSILYFRQLQTRT
NVLALSLALADLLVGLLVMPFASTRTAYGCWFYGRTFCKVHTWFDYTLCTSSILNLMCISLERYVAI
CDPLRYARRVTGATLAAMLVLCWSGVVAYGLTYLLEWNITGIEDSVQRTTCPDNCPVFMNVQFALTN

TLCAYVTPMLLMLAAYAKVYAMARAQARKISIAMLQTRSADAAVRSRWSAMKREHSATKTLGIIMGA
FVIFWVPFFVVAASEPLLGYASDPLVWEVANWFTYINSTMNPILFAAFNRSFRNAFYLIVSGNILRG
SYRGTDLFSFNDLVK*

Lampetra fluviatilis

>Laf-tarl4a

VMPFYMTQCVYDCWFYSHAFCKVHHFLDAFISTASTMHLCCIAYDRYLAICDPMHYRERFTWKTAAL
LLAAAWLGSLLYVVPVMLGWIVIGIEDMMESRTCPDNCVFYMNKVFSSCGAFFSFVVPMTIMTLAYA
KIYRVARKQARSISVQQQGGQGKSGFNKQQWAAMKREHNATKTVSIILGVFMFCWAPFFSTIVNDPW
IDFKTNSIFWDIVNWFGHVNSAINPYLYGGFNRTFRNAIRIMFSRKFWQPGSRSAEL

>Laf-tarl6a

MANRTVGPYDHCIDSFYHLNCSSPTFSMSERTAALSAITTLILATVLGNLLIITSIAYFRQLQTRTN
IMALSLAVADLLVGLTVMPYSMMKAVYKCWFYGQFFCNLQYFLDYMLTNSSIMHLGCIAYDRYVAIC
DPLRYPQRVTNHTVVTMLLISWFGPALFSSPILVNFNPEWSRGNIEIISCPNQCLFFVSTWLVSVVG
VCPYVLSLLTMSYVYARIYIVARRQGRQISSVSLQVHAQQQQQQQAEPTNIRQKWTAMKREHSAAK
TLGSIIGVYLLSWLPFYMVVLFFPNFQNSSAAVRITTWIGYISSAINPVLYATLNRPFRSAFVAVIS
CKVLSSTRARTMDLSGVK

>Laf-tarl6b

FFCYLHYFLDYTLTTSSIIHLGCIAYDRYVAICDPLRYPTRVTDRTVAVMLALCWLGAAIFTTPTLL
SLSPTLSRGTIERGGCPDSCMFTVDLGLHFAYGMCPYFLSMFIILLIYAKIYRVARKQARRVTVGVP
IHAQRVEVANGGSSGGGHDGDEAAKRWKSTMKREHNAAKTLGSIIGCFMLSWLPYYILVIVFPFYKN
TSTAYEVVSWIGYISSTVNPILYASINRPFRTAFGRILRLDFFSAEARHLELSNH

>Laf-tarl6c

ICDPLRTAQRITKRTFVAMLFICWFGAAFFTSPSLLSFSPQLVPSAIKRVGCPDDCIFSVDVTLHFF
YGACPYFLTMLVITAMYARIYHVARTQARQIKAATTKVQSQGQQRVSNMRREHNATVTLGSIIGAFF
LSWLPFYIIVLVFPFVADCDVEYEVATWMGYISSAV

>Laf-tarl6d

ISDPLRYESRVTRRTLVVMLVTCWLGSAVSSSPILLSLSPTLSVGTIERISCPNDCVFDINIGIMVV
IGYCPYFVSLVVIVCIYGKIYRIARAQVIKIRAQQGGGGGGGGHRTDNDNAEALVKLRTMKREHSAT
KTLGVIIGFFLISWLPYCVIVLASLALTDTLLAYRVALWIGYISSAF

>Laf-tarl6e

MQNTSVPQVHKECVIFADFNCTGPTQVREVRLVLLIVFAFFITATILGNLLIITAIASFTKLQTHAN
FLALSLAVSDLLVGVFIMPLSMMKTVYDCWFYASVLCNAHYFLDYTLTTATILHITCIAYDRYVAIC
DPLRYATRVTVRTIAALLVLCWLGAAIFSSPILLSFSPTLSRGNIERASCPDDCVFSVSFGILVTIG
MGPYFTAIHVVLLMYAVIYRVARRQSRKVAADSSAVKNMKREHSAAKTLGAIIGTFMLSWLPYYVVA
MMSFTVDGLFIPYRVAMWIGYCSSAINPLLYASFNRPFRTAFRDIFRLRVFSGVVRRDSGLSERSHG
KAMAR

>Laf-tarl6f

ICDPLRYAQRVTRRTLTFMLLFCWIVSAIISTPILLSMSPTLAKGRIEMVGCPNDCRFLISVGILFT
IGMVPYFTSVLLIMLMYGRIYQVARSQARKIGTQSHGLPGVASETTSRWTNMKREHSATKTLGSIIA
AFIISWLPFYILAIVFTMYQDIFLPYKISFWIGYMNSTI

>Laf-tarl6g

ICDPLRYAQRVTRGTLASMIVVIWVGAALLSAPILLSLSETWAKGIIEITGCPDGCHFAVHVILTLV
VGILPYFIIMLLMLLVYGRIYGVARRQSRQINNSSGSRASDADSRSKWAAMRREHSATLTLGMIVGF
FLVSWLPYFTASVLEMAFGISTSAFVWATETWFGYANSVV

>Laf-tarl7a

MLQETVQKVVTCNVSFYNFKCTLSELTQPQRVVLIWVFTIIITITVVGNILTIVSILYFRQLQTRTN
VLALSLALADFLVGCLIMPFSVMRTAYSCWFYGQLMCRIHTWLDYTFTTCSIFNLACISIDRYVAIS
DPLRYDQRVTYRILAVMLTICWGNIIPYGVSYMLKLNINGIESVVAAKSCPDNCSVFMNVPFGLANS
MGAYVLPMLFIMAAYSRIYVMARNQAKRISSLGDQVRASNASDLTMQSKWNAMKRDHNATKTLGMIM
VVLFIVWLPFIVVVATEPVIGYRMDSTVWDVANWFTYFNSRMNPILFASFNNSFRSAFYLIMSGKIL
RGSYRGTDLFNFRRLEK

Petromyzon marinus

>Pm-tarl1a

MDPPSSSVAAPSSPASSNSSWAAPPPLPHCALVQGSFSCPGSALSSAQRAAWLAMMSVAMASAVLGN
LLVVASVLHFRRLQTRTNAFTASLAVVDLLVGLLVMPFKMTRSAYGCWFYGTAFCAAHTCLDIALCT
ASILHLACIAFDRHVAVCDPLRYAQRVTARHVAAMVALSWCCGAVISVVTVSLGWNVVGVPDEVVAA
SCADSCDFLLGAPYAVGSSVCSFFGPAAFVVVAYARILREARRQGRAIACEQWRHQRQQEKQQQQNE
DQQQGRQEDGVGEGGRGGARGEMTGNGGVVGKRYEIGGGRAEGLGTVGKIGSVLEGGRLTTAPAGKA
TQAVGRAADAKSERNATKMLSIVVGIFLASWLPFFLMNVSDPLLGYSIDPRAWEAVTWLGYANSAAN
PVIYGIFSPNFRAAFRVIAARSAFRAGSRDVQLGF*

>Pm-tarl2a

QVTFRAMALLTATWNATSPNDVLARYYGRTFCKAHTCLEYVFTTASILHLGAIAFDRHTAICDPLRY
RQRITPRRVAHLLAASWLLPFLYVPPVSLGWNVVGVEAAARRLSCPDSCVVLKNVGFALVDTCCAFF
APTALMLVAYAKIYRVARRQARQIAASSTGTASVVTASVKQQQQQQQNHLQEQGNEQQATFRRTMKR
EHSATKTLAIILGAFVVCWCPYFVASAVDPFVGFSTEPALLSATLWLCYANSALNPVLYAAFNRRFR
AAFSRIFCRGKGNAAVVVMVAAAWRDRRRPRPPPPASPEEESDS*

>Pm-tarl2b

*RSLCESMALCVTLYESMALHVSMSLWTCSAKPPSLSLSRRYTARVTPGAVAAMLVACWLGPLLYVA
PIMLGLQSVGAEASLALACPDDCPFVMSATFSLAATSCSFVAPMIIMLAAYARIYRVARRHARSIAS
RPPAPPGGGDGAKGMAAASSS!
SAPRRWRQEYNATKTLGVILGAFFALWLPFFSAAVADGLPGGPRVDGTTWHCVTWLGYVNSAVNPVL
YASLNRAFRRAFGILFSPRELAKGRRDAEFTD*

>Pm-tarl2c

MDKHQTNHAAVAADMVYTNSTATPVDAEPGPTCVVLQGAFNCTGPGPLLPPLPSPLAALALAAALL!
LSAAAVLGNALVVLAVAYFARLR!
PTNALALSLAVADLLVGAVVVPFSATRAVRGCWLHGATFCQLHTCLDVMLSTASIVHLSCIAFDRYV
AICDPL**R**YTARVTPGAVAAMLVACWLGPLLYVAPIMLGLQSVGAEASLALACPDDCPFVMSATFSLA
ATSCSFVAPMIIMLAAYARIYRVARRHARSIASRPP!TPWWRRRGQGDGSG!
RLSSAPRRWRQEYNATKTLGVILGAFFALWLPFFSAAVADGLPGGPRVDGTTWHCVTWLGYVNSAVN
PVLYASLNRAFRRAFGILFSPRELAKGRRDAEFTD*

>pm-tarl3a

LLPSRLSTGRQLQTRTNAFTLSLAVADLLVGLLVMPFSATRSLYGCWFFGRTFCKVHTCLDVLLSTA
SIAHLGAIAFDRHTAICDPLRYDQRLTAGRAAALVAICWVGSALLAVGPILLGWNTVGIEELVASSC
PDACVLLMNAPFAIAGSTCSFFVPSFVMVVTYLRIYRVALHQARAVRNVVSVSSVAWEHCDINSRPE
KRTDQRRDRSATKTLGIIMVAFVTCWLPYFSLTLLDPFTGFLAEPWVWESTAWLAYMNSALNPILYP
AFNQAFRQAFRLVFTCAWRLRNVRRANLA*

>Pm-tarl4a

MYMGKYASPALALNSSIQNGTFQSHCVLTQFNLVCRQPTVPPEYAEVILIAMIMSIILNMFGNALII
AAILYFTSSLSVADILVGVVVMPFYMN*CVYNCWFYGHAFCKVHHFLHAFLPTASTMHFCCIGYDRY
LAICDPFHYQERFTRRTAVIALAATWLGSLLYVVPIMLDWIDIEEMVAERTCPDNCIFFTNKVFSSC
GAFFSFVLPMGIMTVTYAKIYRIARKQARSIGAQQHGMLGQTALGFNKQQWAAMKREHNATKTVSII
LGIFICWAPFFGTIVIDPYLNYTTKPVVWEMLNWIGHVNSAINPFLYGCFNRTFRNAICIIFSRRLL
QPGIRSAEL*

>Pm-tarl4b

MYMGKYASPALALNSSVQNGTFQSHCVLTQFNLVCRQPTVPPEYAEIILIAMIMSIILNMFGNALII
AAILYFKQLQVLPNVFTFSLSVADILVGVVVMPFYMNQCVYNCWFYGHAFCKVHHFLHAFLPTASTM
HLCCIGYDRCLAICDPFHYQERFTRRTAVIALAATWLGSLLYVVPIMLDWIVIGIKEMVAERTCPDN
CIFFTNKVFLSCGAFFSFVLPMGIMTVTYAKIYRIARKQAHSIGAQQHGMLGQTALGFNKQQWAAMK
REHNATKTVSIILGIFMICWAPFFGTIVIDPYLNYTTKPVVWEMLNWIGHVNSAINPFLYGGFNRTF
RNAICIIFSRRLLQPGIRSAEL*

>pm-tarl5a

MNSTLIKCIITYYNLNCTTPHLSYTHGLVVQGVLLFFIISTILGNMLVIASIIYFKQLQSHTNSLTL
SLAAADLLVGLVVMPFSMMRTVHSCWFYGKLLCKLHCSLDYIFSTVSILHLSCVAFDRYVAICDPLH
YTSRVTNNTVAFMLILCWTCPLVYFAPFLLSWNIIGIEEMVQSRICADTCAIVKNVWFAYVDTCCAF
ITPMAIMITTYVKIYRVARRQARQVSAASVALQLNRDERQNQWALMKREHNATKTLGIIMGGFLFFW
LPNFVASLIDPVNDFQTDPLVWNIVVWLGYINSTINPLLYASFNRPFRKAFKIMFSLEITHLSTRNK
DLD*

>Pm-tarl5b

MAQSVLKLNLTNLTNVSSNSCVLNAFQLLCPPPEMSYASSVLVQTALVICIVAIAVGNTLVIASIAY
FRELQSRMNVFTLSLALADFLVGVLIMPFSMLKTVHNCWFFGRWFCKIHTSLDYIFSTASIVHISCI
AYDRYNAICHPLCYTQRVNRAKVVVMLGGCWVAPVIYVAPIMLGWNIIGIEEVVASMTCPDSCVMFK
NTTFVLLDTVCAFMVPMSIMGVAYGKIYRVARDQSRRVHSQHQQQVDGNALKREYNATKTLGLIMGA

FCVCWLPYCVVNVVDVFNNDQTSRWVWSVVAWLGYINSGFNPVLYGLFNRPFRRAFAIVLSKRIFTS
DTRNVDLFRED*

>Pm-tarl5c

MAQSVLKLNLTNLTNVSSNSCVLNAFQLLCPPPEMSYASSVLVQTALVICIVAIAVGNTLVIASIAY
FRELQSRMNVFTLSLALADFLVGVLIMPFSMLKTVHNCWFFGRWFCKIHTSLDYIFSTASILHLSCI
AYDRYNAICHPLCYTQRVNRAKVVVMLGGCWVAPVIYVAPIMLGWNIIGIEEVVASMTCPDSCVMFK
NTTFVLLDTVCAFMVPMSIMGVAYGKIYKVARDQSRRVHSQHQQQVDGNALKREHNATKTLGLIMGA
FCVCWLPYCVVSLVDVFVHFQTSGVVWSVVAWLGYINSGFNPVLYGLFNRPFRRAFAIVLSKRIFTS
DTRNVDLFRED*

>Pm-tarl5d

MEPNFTKIANLTNSSSGSCVLNAFQLLCPPPEMSYASSVLVQTALVICIVAIAVGNTLVIASIAYFR
ELQSRMNVFTLSLALADFLVGVLIMPFSMLKTVHNCWFFGRWFCKIHTSLDYIFSTASIVHISCIAY
DRYNAICHPLCYTQRVNRAKVVVMLG

>Pm-tarl6a

MENRTVGPYDHCVDSFYHLNCSSPTFSMSERTAALSAITTLILATVLGNLLIITSIAYFRQLQTRTN
IMALSLAVADLLVGLTVMPYSMMKAVYKCWFYGQFFCNLQYFLDYMLTNSSIMHLGCIAYDRYVAIC
DPLRYSQRVTNHTVATMLLISWFGPALFSSPILVNFNPEWSRGIIEIISCPNQCLFFVSTWLVSVVG
VCPYVLSLLTMSYVYARIYVVARRQGRQISSVSLQVHAQQQQQQVEHTNTRQKWTAMKREYSAAKT
LGSIIGFYLLSWLPFYMVVLFFPNFQNSSAAVRITTWIGYISSAINPVLYATLNRPFRSAFVAVISC
KMLSSTRARTMDLSGVK*

>Pm-tarl6b

MANTTKTLQCIDYHGYNCSGPSLPEKSQPIILIIISFFIVVTILSNLLIISSVIIFRQLQTHTNILA
MSLAVSDFSLGSMVMPLRMMGTVYNCWYYDYSFCNVLYFVDYTLCTSSIFHLSCIAYDRYVAICDPL
RYAQRVTKRAIVAMLLISWLGAALFTSPSLLSFSPQLAPGAIEREGCPDDCLFSVDVSLHFFYGACP
YFITMLVIMAVYARIYHVARRQARQIKAATTKVQSQGQQRVSSMRREHNATVTLGSIIAAFFLSWLP
FYIIVLVFPFVSNFDVEYEVATWMGYISSAVNPLLYASFNRQFRGAFRKILSLRIFRAGERNFRLSE
*

>Pm-tarl6c

MFCGLVFLALDNSTTMDNSTTGSLCSLHYENYNCTGPSLSEEDRILTLAIIIFFIIATILGNLLIIV
AIAFFRQLQTHANILALSLAVSDFFIGLLIMPFRMMHAVYQCWFYASFFCHLHYFLDYTLTTSSIIH
LSCIAYDRYVAICDPLRYPTRVTDRTVAVMLALCWIGAAIFTTPALLSLSPTLSRGTIERGGCPDNC
MFIVNLGLHFAYGMCPYFLSMFIILLIYSKIYRVARRQARRVTADVPMEAQRTEVANGGSSGGSGGG
DEAAKRWKSTMKREHNAAKTLGSIIGCFMLSWLPYDILVIIFPFYKNTSVAYEVVCWIGYISSTVNP
ILYASINRPFRMAFGRILRLDFFSAEARHLELSKH*

>Pm-tarl6d

MDNSTTGSPCSLHYEYYNCTGPSLSEEDRALTLAIIIFFIIATILGNLLIIVAIAFFRQLQTHANTL
ALSLAVSDLLIGLLIMPFSMMHAVYECWFYASFFCHLHYFLDYTLTTSSIIHLGCIAYDRYVAICDP
LRYPTRVTDRTVAVMLALCWIGAAIFTTPTLLSLSPTLSRGTIERGGCPDSCMFTVDLGLHFAYGMC
PYFLSMFIILLIYSKIYRVARRQARRVTAGVPIHAQRVEVANGASSGGGRDGDEAAKRWKSTMKREH

NAAKTLGSIIGCFMLSWLPYYILVIVFPFYKNTSTAYEVVCWIGYISSTVNPILYASINRPFRTAFG
RILRLDFFSAEARHLELSKH*

>Pm-tarl6e

MDNSTTGSPCSLHYEYYNCTGPSLSEEERTLTLAIIIFFIIATILGNLLIIVAIGFFRQLQTHANIL
ALSLAVSDLLIGLLIMPFSMMHAVYECWFYASFFCHLHYFLDYTLTTSSTIHLGCIAYDRYVAICDP
LRYPTRVTDRTVAVMLALCWLGAAILTTPTLFSLSPTLSRGTIERGGCPDSCMFTVDVGLHFAYGMC
PYFLAMFIILLIYARIYRVARRQARRVTAGMPIHSQRGEVANGGSSGGSDGDEAARRWKLTMKREHN
AAKTLGSIIACFMLSWLPYDIMVMVFPFYKNTSAVYEVMSWIGYISSTINPILYASINRPFRIAFGR
ILYLDVFSSEARHLELFQH*

>Pm-tarl6f

MYNNTSNPCAALYHGYNCTGPSLPPTNLCAVVVLLSSFIAATVLGNLLIICSIAFFKQLQTFPTTLA
LSLALSDFLVGLVIMPFAVMKTAYRCWFCASAFCNAHHFLDYMFTNSSIFHITCIAFERYVAISDPL
RYRARVTRKTMAAMLLLCWLGSALFSSPSLVSLMSKSASGGLIQRVRCPNDCVFSVYIGIQAVIGIS
PYFISLVVIVLVYGRIYSIARRQAQKINSEGSRRRGQDAAAASAACTDALHKWKAMRREHNATKTLG
VIIFFFLLSWLPYNVTVIVFEFVAYASDVYQVTLWIGYISSAFNPILYACFNRPFRTAFHRILRLKV
FTLADRDFAFSTAQDGGH*

>Pm-tarl6g

MNHTSAPSTRNPCVTFRSFNCTGPSLPENYRALLLAILAFFTLATILGNLLIISSIAFFRQLQSFPN
VLALSLAFSDFLVGLVIMPLAVTKVIYNCWFYARLYCNVHYFLDFTFTNCSILHLCSIALNRYVAIT
DPLRYESRVTRRTLVVMLVTCWLGSAVSSSPILLSLSPTLSVGTIKRISCPDDCVFDINIGIMVVIG
YCPYFASLVVIVCIYGKIYRIARAQVTKIRAQQGGGAGGGAGAGGGGGPRTDKDNAEALLKLRTMKR
EHSATKTLGVIIGFFLISWLPYYVIVLASLALADTLLAYRVALWIGYISSAFNPILYAAFNRPFRNA
FRLMFRLKVFGEGARDTDLSGRLK*

>Pm-tarl6h

MANLTDTHASAGCILYMDAFNCTGPTMPLPARSASLAVISLFVVTTVLGNLLIISTIAFFKQLQTST
NVLALSLAVTDFLIGLIIMPMAMTKEMFNCWFYGDVFCNVHFFIDYMLTNCSMLHLGSIALQRYVAI
SDPLHYATRMTRRSLAAIIGFCWLGSAVFSSPILLSFSSALSEQTVARKSCADNCLFYVHMGIMFVV
GYCPYFASLLVIVCVYAKIYRIARNQMRKIRSVACKVEETTLQVIKREHNATKTLGVIIFFFLVSWL
PYYLIVLSSMAVKNTMSVYRVALWAGYISSAFNPLLYASFNLHFRNAFKMMLSCKIFAPGARHTNLN
YLNKATNDHN*

>Pm-tarl6i

MDNSSLASSPSCVISFSNLNCTPTSLNSKHDRAVAVFTISLFIAVTILSNLLIVSAVALFRQLQTRA
NALALSLAVSDLLVGVVIMPLSLTKSVYKCWFYAPLLCHVHFFCDYWFTTASVLHLSCIAYDRYVAI
CDPLRYQQRINRRTLVFMLLFCWLCSALLSTPILLSLSPTLSQGTIAKNGCPDDCRFFISVGILFAI
GMWPYFTSVLLVVLMYGRIYRVARGQARKIAALSRASSGLGGGEEAGRAAASRWASMRREHSAAKTL
GAIIAAFIISWLPYYIIAIVLTVDESVILPYKVSVWFGYMNSAINPILYATFNKQFRRAFKAILTFN
TFPSRAADDEMSSTKN*

>Pm-tarl6j

MENTTSTSASKCVVSYYGLNCTKMDFSKSETALIVGIMSFFIATTILSNLLIISAVAFFRQLQTRTN
TLALSLAVSDLLVGVVIMPLAMIKSVYKCWFYVQWFCNVQFFCDYWFTTASVLHLSCIAYDRYVAIC
DPLRYAQRVTRRTLTFMLLFCWIASAIISTPILLSMSPTLAKGRIEMVGCPNDCRFLISVGILFTIG
MVPYFTSVLLIMLMYGRIYHVARSQARKIGTQSHGLPGVSSEATSRWANMKREHNATKTLGSIIAAF
IISWLPFYILAIVFTMYEDIFLPYKISFWIGYMNSTINPILYAVFNKQFRHAFKTMLSFKVFSSTAR
DKEMN*

>Pm-tarl6k

MAENSSSSSSSNSGSGAEGSSECVVSYYGLNCTQMDFSKSESALIVGIMSFFITVTIISNLLIISAV
VFFRQLQTRANALALSLAVSDLLVGVVIMPLAMVKSVYKCWFYARWLCNVQFFWDYWFTTASVLHLS
CIAYDRYVAICDPLRYAQRVTRRTLTFMLLFCWIASALLSTPILLSMSPTLSKGRIETVGCPDDCRF
LISVGILFTIGMWPYFTSVLLIMLMYGRIYRVARGQARKIAAMDRTSSGLGGGEEAGRVAASRWASM
RREHSATKTLGAIIAAFIISWLPFYIIAVIFTMDESVLLAYKVAVWIGYMNSAINPILYATFNKQFR
HAFKEILTLNVFLSPADRNKEMQ*

>Pm-tarl6l

MTYSSSNNTTGHCVELFENQICSKPKLSYEDRIVAMAVLSFFISATIFGNCLIITAIAFFRQLQTNT
NALALSLAVSDLLVGAIIMPFSVNRSVYQCWFHGRVFCKVHFFLDYAICNASILCLGCIAFDRHVAI
CDPLRYAQRVTRGTLASMIVLIWVGAALLSAPILLSLSETWAKGIIEITGCPDGCHFAVHVILTLVV
GILPYFIIMLLMLLVYGRIYGVARRQSRQINNSRGSRASDADSRSKWVAMRREHSATLTLGMIVGFY
LVSWLPYFTASVLEMAFGISTSALVWATVTWFGYANSVVNPILYATFNRPFRTAFRIIFSSEMFAPG
ARGADLHGLKVAGG*

>Pm-tarl6m

MTYTSSNNTTGHCVESFENLICSKPKLSDKDRIAAMAVLSFLISATIFGNCLIITTIAFFRQLQTNT
NTLALSLAVSDLLVGAIVMPFSASRSIHQCWFHGRTFCRVHYYLDYTFCNASILCLGCIAFDRHVAI
CDPLRYAQRVTRGTLASMLALIWVGAALLSAPTLVSLDEAWARGLMEIAGCPDGCHFAVHVGLTLVV
GILPYFIIMLLMLLVYGRIY

>Pm-tarl6n

MMEANSTLGECHVPFLEFNCTGPGLEPTDQALVLSILILFIAATIIGNLLIVCAVLRFRQLQTRTNA
VTTSLAVADLMVGAVIIPCSMTHEVYRCWFFGQTFCRIHYFMDYWFTNASILHLGCIAFDRYVAICD
PLRYQQRVTNHTVVLMLLMCWLCSALFSAPILISLSDVLSNGVMDRTSCPDECVFIVNLGLTFLIGI
CPYFLSLFILSLAYAKIFRVARVQASQIHAAGKHGADSSSSASIVGDANSKRTWLAMKREHNAAKTL
GMIIGFFLLSWLPFYVISVVDVIIDYQTNAVLRRTVTWVGYFSSAVNPVLYASFNRPFRNAFRDIVS
CRAFAVGARNKDLFGS*

>Pm-tarl6o

MPETNSTLGKCAAPFLQLNCTGPGLEPTNQAFVLSIIILLIVVTIIGNLLVACAVLRFRQLQTRTNA
VTTSLAVADLMVGAVIIPCSMTQEVYRCWFFGQTFCRIHYFMDYWFTNASILHLGCIAFDRYVAICD
PLRYQQRVTNRTVVLMLLMCWLCSALFSAPILISLSDVLSRGVMERTSCPDECVFIVNFGLMFIIGI
CPYFLSLITLSLAYAKIFSVARVQASQIHAAGKHGADSSSSASIVGDANSKRTWLAMKREHNATKTL
GMIIGFFLLSWLPFYIISVVDVIIHYQIDAVMWGTVTWIGYFSSAVNPILYASFNRPFRNAFRDIVS
CRAFTSRRGT*

>Pm-tarl7a

MFWLHIRTSMDNQSDVEAPRLLCVNSYNNLSCSSPRLPAPQRTLALVVLISFSACTVVGNSLIVLSI
LYFRQLQTRTNAFTLSLALADMLVGLLVMPYSMMRAVYSCWFYGSLFCKVHTWFDFTFTSSSVIHLS
CISIDRYIAVSDPLRYPKRLGPS**KV**ALMLVVCWSTTLAYGLPFFSGWTVLGIEQVIARGSCRDACPV
VGNMAFAFTNAFFAYLIPLCIMVTSYAKIFRIAREQVNKIRANSAHVPGVEFSSSSTRQQWAAMRRE
HNATKTLGIIMGVFLIFWLPYCSVAVIDPIIGYQTSATSWDVANWIAYVNSAINPVLFASFNRSFRG
AFRLIFTCRVSSSTYRNADLFNLKDSAN*

>Pm-tarl7b

PRSSRGTIAGNLMVMAFIAYFRHLQTRTNALALSLALADFLVGLLIMPYSTMRSVDRCWPYGGGVFC
RVHTWLDFSLTTSSVVHLCCISYDRYVAVADPLRYPQRVTRRAVALLLLCCWLSVPVYSWPIMSGWY
SVGVPDGPGAFPGCREVCPVSVNATFGLANMLCAYAAPMLLMSLTYAKIYRLARAQARKIDAATAGL
RRLGPADGAADDGDGGRWKAAMRREHNATKTLGVIAGVFVLCWVPYFVLSASDSLLDDATRRAAADI
SNWLTYVNSTLNPVLVVALNRSFRNAFRATVACRVFAPGFRTADLFNYDGSER*

>Pm-tarl7c

*VYRSLCESRQLQSRSNVLVASLAVADLLVGLLIMPFSAARTAYNCWFYGDLFCHLHTWLDFSTCTS
SIANLACISLERYASIAEPLRYRQMVTPRVLAAMLLLSWSGLPLYGATFMLGWNLVGIKRQVTESSC
RHDCRVYMNPASTATNVLVAYVTPMLLMIVANAKIHRLARAQVRRIEGSAMVSGRDDRASTKREHNA
TQTLGIITGTFILLWMPYFILVASEPLLGYGTSRAAWEVVCWLPYLNSAVNPVLLMVFNRSFNGAFR
LALKGGVLKSKCRGIDLFNFRDGNM*

>Pm-tarl7d

MNESGSIDSSRRCVWTFHQVSCTSSDLSAAERATLLAILLSISAITIAGNILTIASILYFRQLQTRP
NVLTLSLAVADLLVGLLIMPFNAMSSMHNCLFYGRVSCRVHTWLDYIFCTSSIVHLSCISFDRYVAI
SDPLRYSDSVTHAALAAMLALSWFSFPIDGLVFMMQWNLVGLEEETRRSCPDDCEVILNLPYAVAIT
TCAFVLTMLLMALAYDRIYRLARVQSREINSLGAQVQKASSDGECLSKWNAMKRDHNATITLGIIIG
IFIVIWMPYFVVSATEPMVGYQAGPVAWEVINWFTYLNSMANPILFAAFNMSYRWAFRLLVMCSAFR
PGIRGVDLFNFMERPA*

>Pm-tarl7e

METLNESIRNSSLLCVEAFHNFHCTYNDVSEAERAILIAIIIPAIAITIFGNLLTVVSILYFRQLQT
RTNVLTLFLAVADLLVGLLIMPFSAMRSVYNCWFYGWTFCKIHSWFDYTLCTLSVLLLSCISFDRYV
AISDPLRYHQRITNRTCALMLLFCWLCLPFYGLVFMLEWNLVGLEEELAQICPDDCPVLLNLPFAMA
NTIFGCVVPMILMTLAYGRIYQLARQQARKITSTAMGSNVDSARSSLRREHSATITMGIIVGVFISL
WMPYFVVSTTESIFGYQASSLAWEFINWFTYINSTVNPILFAAFNRPFRNAFYLILSGRIFSSSYRG
VDLFNVQHGSKVNQGAKRCVSTLISKESDR*

>Pm-tarl7f

MPLHNESFPSVLCVVTFATFNCTPSQMTNVERALLMGAFTIAVATTITGNMLTISSILYFRQLQTRT
NVLALSLALADLLVGLLVMPFASTRTAYGCWFYGRTFCKVHTWFDYTLCTSSILNLMCISLERYVAI
CDPLRYARRVTGATLVAMLVLCWSGVVAYGLAYLLEWNIAGIEDSVQQTTCPDNCPVFMNVQFALTN
TLCAYVTPMLLMLAAYANVYAMARAQARKISIAMLQARSADAAVRSRWSAMKREHSATKTLGIIMGA

FVVFWVPFFVVAASEPLLGYASDPVVWEVANWFTYINSTMNPILFAAFNRSFRNAFYLIVSGKIMRG
SYRGTDLFSFNGSGK*

Cartilaginous fish and tetrapod TARL

>Cm-tarl2

MDTSHSPPFNYTLSCNSSESVPYTARQVIQCVTTLSVLTIITLLTVLGNVLVIASIAYFTKLQTPTN
AFILSLATADILVGLVVMPFSMVKAVFGWYFGRIFCKIHSIMDVMLCTSSIVNLSCIAFDRYFAVCY
PLKYRFTMSRKRVTFLLLICWILPALVSIVPLVLDLHVIGLEDSFEQLDSQDCVFVVNIPYSVTASI
IAFYLPMPVILVAYAKIFLVATNQVREIAARQQGMNLEMIQQNSSIRKERKAAKTLGIIIGCFLLCW
VPFFTLNIIDPLLGYQADSILIEVFLWLGYLNSGLNPFLYAFFNKSFRRAFSMIISCKIITKDCHYN
SLAGSTKLNTRLKTISRQFTLTSELFDEEE*

>Le-tarl2

MLSAVIICPTSQVVNVLIYNCSAHRNSSAIVPRATWQIIEQVFILTVLTI!
ILGAFLGNLLVIVSIIYFTKLRTVTNAFLLSLAVADFLVGIIVMPFSMIKVMFGWYFGKAFCTIHTV
MDVMLCTSSIMNLSCIAFDRYYAV*YPLRYRFRVSQKRVTTLLLVCWVLPGLVSFVPLLLGLHLTGL
ETIHSQLDPHDCVFIVNIPYALCASVIS!YLPMLVMLAAYGKIFQV!
IQARQIYTMENGSHEMDATRFHHSTSTKKERKAAKTLGVIIGCFLFCWLPFFTANIANALLGYQVHH
IVLEVFVWLGYVNSALNPLLYALFNKSFRRAFGLIL

>Rt-tarl1

MQNTSGLFKVSTSSGWRMADRCLSSIGSPAVRTGLYLFIMVIMAATLAGNFLVVLAVVYFKQLRSAT
NAFVLSLSIADFFVGIMVMPYSMSRTIEGCWHFGPTFCQIHSSLDVMFCTASILHLSCIALDRYYAI
CDPLVYRCKMSPSRVVVLLLACWVVPAAISVIPIMLQLHRLGVDPDLLTNRSCVLMVNQTYAIWASL
ISFYLPMVVMLVAYWKIYQAARKQAIQINAMDNQVQGYASGLATAHVRKQSNKRERKAAKTLGIIIG
IFLSSWLPFFMANIVDPFLGYKIDRGTWEVLVWLGYSNSSMNPILYGVVNRSFRRAFLIIISCKICV
SRSLQKHRLIQQ*

>Rt-tarl2

MFSAIIISTSSQEMNTTPFNNWTTHSNSSGKASSATWQVIDQVVVFSVLTVIIAGTIFGNLLVIASI
AYFTKLRTPTNAFIVSLAVADFLVGIIVMPFSMAKLVFGWYFGKAFCKIHTIMDVMLCTSSIMNLSC
IAFDRFYAVCYPLKYRFRMSQKRVTVLLLICWFVPALMSFVPLLLDVHLRGLEAILLPFDPHSCVFM
VNIPYAVSASVIAFYFPMLVMLVAYGKIFLVARIQARQVYTIENGLEGRNTLTRCQNSSMKKERKAA
KTLGIIMGCFLICWLPFFTTNIVNPLLGYPAHHILLEVFLWLGYVNSTLNPLLYAFFNQSFRRAFYM
VIGCKILASDCQNNNLSDSIRQNTQLATLSR*

>Xl-tarl1

MLNTSKWLQNASRSTNSPDESCSSVVSSSTFKLILYSLTGGLIVTTFLGNILVITSIAYFKHLHSPT
NSFVLSLAVADFLVGGLVMPFSMIRTIEGCWYFGSVLCRLHSSLDVMLCSASILHLSCIAFDRYYAV
CNPLLYSYKMSTRRVSVLICTCWVIPMLISFAPIMLGLHLLGMEHLLQEGTCLFVVNHIYSVSASLI
TFYCPMTIMFVAYCKIYRAARKQALQIHDMKRNVTTQYGGDCSMKKRKYSLKRERKAAKTLGVIMGL

FLFFWSPFFTANIVDPLIGYKMGMVGWEVFLWLGYVNSALNPFLYGLLHKSYRRAFFMIIGCKTCYS
ATSLNIDLSSTKQEKVKRKTTDLH*

>Xt-tarl1

MAVASTTASSPMDSCSSVVSSSTSKLILYLLTSGLIMTTFLGNILVISSIVYFKQLRSPTNSFVLSL
AVADFLVGVMVMPYSMVRSIEGCWYFGSGFCRLHSSLDVMLCSASILHLSCIAFDRYYAVCNPLLYG
YKMSTRRVSILICTCWFIPVLISFAPIMLGLHLLGMEHLWQEGACLFVVNQIYSVCASLIAFYCPMI
IMLVAYCRIYRAARNQALRIHAMERNVSSGNASDGPMKKRKYSLKRERKAAKTLGVIMGLFLLFWTP
FFTANIVDPLIGYKMGTVEWEVCLWLGYVNSALNPFLYGLFHKSYRRAFFMIVGCQTCYSGTSQNIE
LSTAKQEKVRRQMNVLH*

Bony fish TARL

>Aa-tarl1

MANTSAAANVSVDTGGDSTPCVPWRSQGSRVALYMFIMAGIACTVVGNFLVVLAIAYFKRLQSPTNS
FVMSLAVADFLVGLIVMPYSMVRTVEGCWYFGPTFCEVHSSLDVMLCTASIFHLSCIAFDRYYAVCN
PLVYAFKMSRVRVGLLIVVCWVVPLLISFGPIMLGLHKVGIDIQLPEGICAFLVNRVYAVMASLVAF
YLPMVIMLVAYWKIYKAAKRQAMQISAMEHQMTVHGLNSAGRKQKQRQNCMRRERKAAKTLGIIMGA
FLLFWLPFFTTNVVDPFIEYRTAGVVWDVFLWLGYVNSSLNPFLYAFFNRSFRRAFFMIMGCRICLP
GSPASIDLSLTKRDANEHIEN*

>Ac-tarl1

MDNSTLGWLGDDNTSLQLELQPCALLRHQVSRIFLYAFLSFGIICTVVGNFLVVLSIAYFKQLQSPT
NCFVMSLAVADCLVGLVVMPYSMIRTVEGCWYFGSLFCQIHSSLDVMLCTASIFHLSCIAFDRYYAV
CNPLVYSLKMSHKRVALFIAVCWVVPILISFGPIMLDLHTAGVNILIPKDLCVFLVNRIYAVMASLV
AFYLPMAIMLVAYWKIFKAAKRQARQISAMESQMAAGVGKDSSKKKRHRNAMKRERKAAKTLGIIMG
VFLIFWMPFFTVNIVDPFIEYSTEVIVWDLFLWLGYINSSLNPFLYGLFNRSFRRAFLMFMGCRVCL
PGSSSGMELSHTRKEANECADQS*

>Af-tarl1

MDNGSLGLLGDANTSLQNELQSCITLRNQVSRIFLYAFLSVGIVCTVVGNFLVVLSIAYFKQLQSPT
NSFVMSLAVADCLVGLVVMPYSMIRTVEGCWYFGVQLCKLHSSLDVMLCTASIFHLSCIAFDRYYAV
CNPLVYSLKMSRSRVALLIVICWAVPTLISFGPIMLDLHIADVDILLPKNVCVFLVNRIYAVMASLV
AFYLPMAVMLIAYWKIFKAAKRQARQISAMESQMAAGVGKDSSKKQRHRNTMKRERKAAKTLGIIMG
VFLIFWMPFFTVNIVDPFIEYSTEVVVWDIFLWLGYINSSLNPFLYGFFNRSFRRAFLMFIGCRVCL
PGSSPGMELSHTKERGK*

>Aj-tarl1a

MANTSAAANVSVDTGGDSTPCVPWRSQGSRVALYMFIMAGIACTVVGNFLVVLAIAYFKRLQSPTNS
FVMSLAVADFLVGLIVMPYSMVRTVEGCWYFGPTFCEVHSSLDVMLCTASIFHLSCIAFDRYYAVCN
PLVYAFKMSRVRVGLLIVVCWVVPLLISFGPIMLGLHKVGIDIQLPEGICAFLVNRVYAIMASLVAF
YLPMVIMLVAYWKIYKAAKRQAMQISAMEHQMTVHGLNSAGRKQKQRQNCMRRERKAAKTLGIIMGA

FLLFWLPFFTTNVVDPFIEYRTAGVVWDVFLWLGYVNSSLNPFLYAFFNRSFRRAFFMIMGCRICLP
GSPASIDLSLTKRDANEHIEN*

>Aj-tarl1b

MPCVSAFIFPAAERSHNHTETGFSNCSAGDHDSMESMKVLLLFLLLPIIVFAILGNLFIVLSVAYFR
QLQTSTNAFIVSLATADFLVGALVMPFSLVRSVDRWRFGQLFCTAHFLLDVILCTASIFNLSCVALD
RYLAVCDPLRYPARMSAARVAKLLLLSWLIPVSGSCLLVVADLHTQSGEGVLTGRGGEECFPEMRAP
YGVMASVLSFFLPMAFVVAAYGRIFQEAQRQARQIQAAEAHVRQSHPAQSPSVPQATPGIAARKAGK
ATRTLGILLGGFLLCWLPFFSVNVGHPLCGRPVGRQVQECVLWLGYANSALNPVLYAFVNRAFRRSF
VILLACGILGRRLQDGPLDPGASRNTGPELGSVAR*

>Al-tarl1

MDNISLEWTREANTSLQTEMSCATLRNQVSHIFLYTFLSAGIVSTVVGNFLVVLSIAYFKQLQSPTN
CFVMSLAVADCLVGLLVMPYSMIRTVEGCWYFGSLFCKLHSSLDVMLCTASIFHLSCIAFDRYYAVC
NPLVYSLKMSPNRVALLIAVCWAVPMLISFGPIMLDLHIADIDIPIPEDMCVFLVSRIYAVMASLVA
FYLPMAVMLVAYWKIFKAAKRQAQQINAMESQMAAGVGKDSSKKKRHRNTMKREGKAAKTLGIIMGV
FLIFWMPFFTINIMDPFINYSTEVVVWDVFLWLGYINSSLNPFLYVYFNRCFRRAFLMFIGCKVCLP
GISPGMELSHSKKEAN*

>Am-tarl1

MANSSDWLEDSNASLVGDLEACTTVRSPGSRIALYIFIIIGIFCTVVGNFLVVLAIAYFKQLQSHTN
SFVMSLAVADFLVGLVVMPYSMVRTVEGCWHFGATFCHLHSSLDVMLCTASIFHLSCIAFDRYYAVC
NPLVYSFKMSRSRVALLIVVCWAVPLLISFGPILLGLHKLGVNVSLPENVCVFLVNRVYAVIASLVA
FYLPMATMLFAYWKIYKAAKRQAMQISAMEAQMAAGVGKDSSKKQKHRNSMRRERKAAKTLGIIMGV
FLLFWLPFFTVNIVDPFIDYSTAGVIWDVFLWLGYVNSSLNPFLYGFFNRSFRRAFLMIMGCRICLP
GSAPGMDLSLSRKDANERTENQ*

>Ar-tarl1

MQCFFFGRFQEAMANTSAAANVSVDTGGDSTPCVPWRSQGSRVALYMFIMAGIACTVVGNFLVVLAI
AYFKRLQSPTNSFVMSLAVADFLVGLIVMPYSMVRTVEGCWYFGPTFCEVHSSLDVMLCTASIFHLS
CIAFDRYYAVCNPLVYAFKMSRVRVGLLIVVCWVVPLLISFGPIMLGLHKVGIDIQLPEGICAFLVN
RIYAVMASLVAFYLPMVIMLVAYWKIYKAAKRQAMQISAMEHQMTVHGLNSAGRKQKQRQNCMRRER
KAAKTLGIIMGAFLLFWLPFFTTNVVDPFIEYRTAGVVWDVFLWLGYVNSSLNPFLYAFFNRSFRRA
FFMIMGCRICLPGSPASIDLSLTKRDANEHIEN*

>Ar-tarl2

MPCVSASIFPAAEQSHNHTETGFSNCSAGNHDSTESMKVLLLFLLLPIIVFAILGNLFIVVSVAYFR
QLQTSTNAFIVSLATADFLVGALVMPVSLVRSVDRWRFGRLFCTAHFLLDVILCTASIFNLSCVALD
RYLAVCDPLRYPARMSAARVTKLLLLSWLIPVSGSCLLVVADLHTQSGEGVLTGRGGEECFPEMRAP
YGVMASVLSFFLPMAFVVAAYGRIFQEAQRQARQIQAAEAHVRQSHPAQSPSVPQATPGIAARKAGK
ATRTLGILLGGFLLCWLPFFSVNAGHPLCGRPVGRQVQECVLWLGYANSALNPVLYAFVNRAFRRSF
VILLACGILGRRLQDGPLDPGASRNTGPELGSVAR*

>Bp-tarl1

MANHTSETPASFNFTDVLELDCSILRNQTSQVFLYIFLSLVIICTVVGNSLVVLSIAYFKRLQSATN
FFVLSLAVADCLVGLIVMPYSMVRTIEGCWIFGPLFCQIHSSLDVMLCTSSIFHLSCIAFDRYYAVC
NPLCYSLKMSNNRVVFLIILCWGVSSLISFGPVMLELHVANTDIEIPKDSCVFVVNQIYAVLASVVS
FYLPMLTMLLAYWKIYKVARKQARQISAMESQMGKDTSKKKRHRNAMKREGKAAKILGIVMGVFLLF
WMPFFTINVVDPFIGYRTEAVVWDVFLWLGYLNSSLNPLLYGFFNRSFRRAFLMFIGCRVCLHQPLS
WVELSQTKKDVQNEQRN*

>Cc-tarl1a

MENSSEWSNISLDSDFEPCATLRSSGSRVALYVFIIAGIVCTVVGNFLVVLAIAYFKQLQCPTNSFV
MSLAVADFLVGLVVMPYSMVRTVEGCWYFGPTFCHLHSSLDVMLCTASIFHLSCIAFDRYYAVCNPL
VYSFKMSRKRVGLLIVVCWAIPFLISFGPILLGLHKLGMDVPLTENMCIFLVNRVYAVMASLVAFYL
PMVTMLVAYWKIYKAAKRQAMQISAMEAQMAAGVGKDSSKKQKHRNSIRRERKAAKTLGIIMG!
FLLFWLPFFTVNIVDPFIEYGTAAVIWDVFLWLGYVNSSLNPFLYGFFNRSFRRAFLMIMGCRICLH
GTAQGMDLSHSKRDANERTDNQ*

>Cc-tarl1b

MENSSEWINISLDSEFEPCATVRSSSSRVALYIFIITGITCTVIGNFLVVLAIAYFKQLQCPTNSFV
MSLAVADFLVGLVVMPYSMVRTVEGCWYFGPTFCHIHSSLDVML*TASIFHLSCIAFDRYYAVCNPL
VYLFKMSRKRVGLLIVVCWAIPFLISFGPILLGLHKLGMDVPLPANVCIFLVNRVYAVMASLVAFYL
PMVTMSVAYWKIYKAAKRQAMQISAMEAQMAEGVGKDSSKKQKHSNSIRRERKAAKTLGIIMGGFLL
FWLPFFTVNIVDPFIEYGTAAVIWDVFLWLGYVNSSLNPFLYGFFNRSFRRAFLMIMGCRICLHGTT
QGIDLSNSKRDASEHTDKK*

>Ch-tarl1

MNASWLDESNASVTGVIESCAILRNPGTRVALYTFFLAGIVCTVVGNFLVVLAIAYFKQLQSPTNCF
VMSLAVADFLVGLVVMPYSMVRTVEGCWHFSATFCQLHSSLDVMLCTASIFHLSCIAFDRYYAVCNP
LVYSFKMSRARVALLIVVCWAVPLLISFGPILLGLHKAGVAIPLPENMCVFLVNRIYAVMASMVAFY
LPMAIMLVAYWKIYKAAKRQAMQISAMESQMAAGVGKDSSKKQRHRNSLRRERKAAKTLGIIMGVFL
LFWLPFFTVNIVDPFIDYSTAGEVWDVFLWLGYVNSSLNPFLYGLFNRSFRRAFFLIMGCRICLPGS
NPSMDLSHSKRDGNDN*

>Cn-tarl1

MENGSREWPGNSNASVQIELLACVTMRSHISRIFLYTFLSVGIVCTVVGNFLVVLSISYFKQLQSPT
NSFVLSLAVADCLVGLLVMPFSMIRTVEGCWYFGSLFCKFHSSLDVMLCTASIFHLSCIAFDRYYAV
CNPLVYSLKMSNSNVALLIAICWTVPMLISFGPIMLDLHIANVDILIPSDLCVFLVSRVYAVMASLV
AFYLPMAVMLVAYWKIYKAAKRQSKQISAMESQMAAGVGKDSSKKKRHRNTMKREGKAAKTLGIIMG
VFLVFWMPFFTVNIVDPFIEYSTEVVVWDVFLWLGYINSSLNPFLYGFFNHCFRRAFIMFIGCRVCL
PGISPGMELSHSKKVKN*

>Cr-tarl1

MCFDRLNLTMDNSSLGLLGDANTSLQIELESCITLRNQVSRIFLYVFLSVGIVCTVVGNFLVVLSIA
YFKQLQSPTNSFVMSLAVADCLVGLVVMPYSMIRTVEGCWYFGVLLCQLHSSLDVMLCTASIFHLSC
IAFDRYYAVCNPLVYSLKMSHSRVALLIVICWAVPTLISFGPIMLDLHIAGVDILLPKDVCVFLVNR
IYAVMASLVAFYLPMAIMLIAYWKIFKAAKRQAMQISAMESQMAAGVGKDSSKKRRHRNTMKRERKA

AKTLGIIMGVFLIFWMPFFTVNIVDPFIEYSTEVIAWDIFLWLGYINSSLNPFLYGLFNRSFRRAFQ
MFMGCRVCLPGSSPGMELSHTKKEANERAD*

>Cs-tarl1

MDYGSLGLSRDYNRSFQIEIESCPAPRSQASRVLLYAVFFAVITCTVFGNFLVVLSIAYFKQLQSPT
NSFVMSLAVADCLVGLIVMPYSMVRTVDRCWYFGALFCRIHSSLDVMLCTASIFHLSSIAFDRYYAV
CNPLIYSLKMSRSRVALLIVACWVVPMFISFAPIMLDLHVAGVNVYLPEELCLFLVNRIYAVMASVV
AFYLPMVIMLAAYWSIFKAARRQAQQISVIESQMAAGVGKDTSRKRRHRNTIKRERKAAKTLGIIMG
VFLIFWMPFFTVNILDPFIDYSTEVVVWDVSLWLGYINSSLNPFLYGFFNRSFRRAFLMFLGCKVCL
PGTSSGMELSQTRKEPSGRTDKA*

>Cv-tarl1

MENGSREWPGNSNASVQIELLACVTMRSHISRIFLYTFLSVGIVCTVVGNFLVVLSISYFKQLQSPT
NSFVLSLAVADCLVGLLVMPFSMIRTVEGCWYFGSLFCKFHSSLDVMLCTASIFHLSCIAFDRYYAV
CNPLVYSLKMSNSNVAMLIAICWTVPMLISFGPIMLDLHIANVDILIPSDLCVFLVSRVYAVMASLV
AFYLPMAVMLVAYWKIYKAAKRQSKQISAMESQMAAGVGKDSSKKKRHRNTMKREGKAAKTLGIIMG
VFLIFWMPFFTVNIVDPFIEYSTEVVVWDVFLWLGYINSSLNPFLYGFFNHCFRRAFIMFISCRVCL
PGISPGMELSHSKKEKN*

>Dl-tarl1

MDNSSLGLLGDVNTSLKTEIQSCTTLRNQASRIFLYAFLSVGIVCTVVGNFLVVLSITYFKQLQSPT
NSFVMSLAVADCLVGLLVMPYSMIRTIEGCWYFGVLFCRLHSSLDVMLCTASIFHLSCIAFDRYYAV
CNPLLYSLKMSRSRVALLIVVCWAVPMLISFGPIMLDLHIAGVDVLLPEDVCVFLVNRIYAVMASLV
AFYLPMAIMLVAYWKIFKAAKRQARQISAMESQMAAGVGKDSSKKQRHRNTMKRERKAAKTLGIIMG
VFLIFWMPFFTVNIVDPFIEYSTEVVVWDIFLWLGYINSSLNPFLYGLFNRSFRRAFLMFMGCRVCL
PGSSPGMELSHTRKEANERADQP*

>Dr-tarl1

MENTSEWSNNSLDSDFEACATLRSFGSRVALYLFIITGILCTVVGNFLVVLAIAYFKQLQCPTNSFV
MSLAVADFLVGLVVMPYSMVRTVEGCWYFGPTFCHLHSSLDVMLCTASIFHLSCIAFDRYYAVCNPL
VYSFKMSRKRVGLLIVVCWAIPFLISFGPILFGLHKLGVDIPLPENMCVFLVNRIYAVMASLVAFYL
PMVTMLVAYWKIYKAAKRQAMQISAMEAQMAAGVGKDSSKKQKHRNSIRRERKAAKTLGIIMGVFLL
FWLPFFTVNIIDPFIEYGTAVVIWDVFLWLGYINSSLNPFLYGFFNRSFRRAFLMIMGCRICLHGSA
QGMDLSHSKRDGNERTDN*

>El-tarl1

MDNSSLGWSADIDVVSNTSSSPRGSVEDLCATSARRRASRVTLYAFLTAGIVCTVVGNFLVVLAIAY
FKQLQSPTNSFVTSLAVADCLVGLVVMPYSMVRTAEGCWLFGTVFCRIHSSLDVMLCTASIFHLSCI
AFDRYYAVCNPLVYHQKMSQGRVALLIVACWAVPLFISFGPIMLGLHEAVAPAAPDNTCVFLVNRIY
AVAASLVAFYLPMGVMLAAYWKIYRAAKRQAMQISAMESQFSAGVGKDSSKEQRHRNAMRRERKAAQ
TLGIIMGVFLLFWLPFFTVNIVDPFVDHGTAAEVWEVFLWLGYVNSSLNPFLYGLFNRSFRRAFVMI
MGCRICAQSLSPGMDLSKEGNEHTNK*

>El-tarl2

MMFLASNRSTTDSCVNLSAPGGHSESTSGTLQSTTVKVCVVCILLPIPVLAILGNILIWASVVRFRS
LQTPTNSFIVSLATADFLVAVLVMPFSLVGSVGTWCFGQNFCVAHFLLDLTLCTTSIFNLSCVALDR
YVAVCDPLHYPARMSTRRVTLLLFLSWLIPLLVSSLCVSLGTYSLNAPSGHRGAQQESQTCVAQFHT
PYAVAVSTVCFFIPVLFMLFAYGKIFMAAQRQARWIHAIENQVRQLHTAQNPSRDDPNQPHPARRVG
ARVGGFSIRKEKKAARTLGLIMGVFLLCWLPHFSVNIAFSLWGTRISPVVLDAFMWLGYANSSLNPF
IYASFNKDFRHAFAAILGFRILGRRIRGCLVATQEVSRRVQTGVTMETISK*

>Fh-tarl1

MENSSLAWPGNDSTSLQIELPSCATLRSQISRILLYAFLSAGVVCTVVGNFLVVLSISYFKQLQSPT
NSFVLSLAVADCLVGLLVMPFSMIRTVEGCWYFGPLFCKFHSSLDVMLCTASIFHLSCIAFDRYYAV
CNPLVYSLKMSNSHVAQLIAICWTVPMLISFGPIMLDLHIAYVDIFIPSDVCVFLVNRIYAVMASLV
AFYLPMAIMLVAYWKIFKAAKRQAKQISAMESQMASGVGKDSSKKKRHRNTMKREGKAAKTLGIIMG
VFLIFWMPFFTVNIVDPFIEYSTEVVVWDVFLWLGYINSSLNPFLYGFFNRCFRRAFLMFLGCRVCL
PGISPGMELSHSKKGIN*

>Ga-tarl1

MENISLDLLGEGNTSLQNELYSCLTLRNQVSRIFLYAFLSVGIVCTVVGNCLVVLSIAYFKQLQSPT
NSFVMSLAVADCLVGLLVMPYSMIRTMEGCWYFGVLLCQLHSSLDVMLCTASIFHLSCIAFDRYYAV
CNPLLYSLKITRGRVALLIVICWAVPTLISFGPIMLDLHIAGVDIPLPGDVCVFLVNRIYAVMASLV
AFYLPMAIMLIAYWKIFKAAKRQAVQISAMESQMAAGVGKDSSKKQRHRNAMKRERKAAKTLGIIMG
VFLIFWMPFFTVNIVDPFIEYTTEVVVWDTFLWLGYINSSLNPFLYGFFNRSFRRAFLMFMGCRVCL
PGTSPGMELSHTRKEANEHSDQP*

>Gm-tarl1a

MDNSSLDPRTPEPSDTSLHGTAPPCTTERHQASRLLLYSLLSAAVVCTVVGNLLVVLSIAYFKQLQS
PTNCFVMSLAVADSLVGLVVMPFSMLRTLDGCWRLGALFCRLHSSLDVMLCSASIFHLSCIAFDRYY
AVCNPLLYSLVMSRDRVAALIVGCWAIPMLISFGPIMGGLHAVGVDVPLPPDVCVLLVNRAYAVTAS
LVAFYLPMAVMLVAYWKIYKAAKRQAMQISAMESQMAAGVGKDSSKKQRHRNAMRRERKAAKTLGII
MGVFLLFWMPFFTVNIVDPFIEHSTHALVWDVFLWLGYINSSLNPFLYGLFNRSFRRAFLMIMGCRI
CLPGASPRIDLSQSQEGAERTDQQ*

>Gm-tarl1b

MFERPPRHRPPATRSGTPGPPPARSTASPAPPWSAPWVGNLLVVLSIAYFKQLQSPPTASSCPLAVA
DSLVGLVVDALQQLRTWTALRAARAPLLPPCHPAWKSCCAAASISTSAASPSTATTPLSTPLPLLAG
DVAGPRGRRLIVGCWAIPMLISFGPIQWGASTRSAWMSPAPDVCVLLVNRAYAVTASLVAFYLPMAV
MLVAYWKIYKAAKRQAMQISAMESQMAAGVGKDSSKKQRHRNAMRRERKAAKTLGIIMGVFLLFWMP
FFTVNIVDPFIEHSTHALVWDVFLWLGYINSSLNPFLYGLFNRSFRRAFLMIMGCRICLPGASPRIV
DLSQSQEGC*

>Hb-tarl1

MDNSTLGWLGDDNTSLQLELQSCTLLRHRVSRIFLYAFLSVGIICTVVGNFLVVLSIAYFKQLQSPT
NCFVMSLAVADCLVGLVVMPYSMIRTVEGCWYFGSLFCRIHSSLDVMLCTASIFHLSCIAFDRYYAV
CNPLVYSLKMSHRRVALLIAVCWLVPILISFGPIMLDLHVAGVDMVIPKDLCVFLVSRIYAVMASLV
AFYLPMAIMLVAYWKIFKAAKRQARQISAMESQMAAGVGKDSSKKKRHRNAMKRERKAAKTLGIIMG

VFLIFWMPFFTVNIVDPFIEYSTEVVLWDLFLWLGYINSSLNPFLYGLFNRSFRRAFLMFMGCRVCL
PGSSPGMELSHTRKEANECAEQS*

>Hc-tarl1

MDNGSFEDGANTTLDINQDSCAMMRNPVFRFILYGFFFIGIICTVVGNFLVVLSISYFKQLQSPTNS
FVMSLAVADCLVGLVVMPYSMIRTVEGCWFFGSLFCQLHSSLDVMLCTASIFHLSCIAFDRYYAVCN
PLVYSLKMSPSRVAFLIVICWVVPILISFGPIMLGLHIAGVDILLPQEVCVFLVNRVYAVMASLIAF
YLPMAIMLVAYWKIFKAARRQARQISAMESQMAAGVGKDSSKKKKHRNTMKRERKAAKTLGIIMGVF
LIFWMPFFTVNIVDPFINYSTEVVIWDIFLWLGYINSSLNPFLYGFFNRSFRKAFFMFMGCSVCRPG
ISPGMELSHTRKEINDCADK*

>Ip-tarl1

MANSSEWMDSSNESFVGDLEACAKARSPSLRVALYSFIIIGIFCTVVGNFLVVLAIAYFRQLRSHTN
SFVMSLAVADFLVGLVVMPYSMIRTVEGCWNFGKTFCQLHSSLDVMLCTASIFHLSCIAFDRYYAVC
NPLVYSFKMSRNRVALLIVICWAVPLLISFGPILLGLHELGVDVPLPENVCILLVNRVYAIIASLVA
FYLPMATMLVAYWKIYKAAKRQAMQISALETQMATGVGKNSSKKQKHRNYLRRERKAAKTLGIIMGV
FLLFWLPFFTVNIVDPFIEYRTTSVIWDVFLWLGYVNSSLNPFLYGFFNRSFRRAFLMIMGCVICLP
GSAPIMDLSQSKKDTNERTEKQ*

>Km-tarl1

MDNSSLEWSGDGNASLRTELQSCATLRNQVSRIFLYAFLSAGIVSTVVGNFLVVLSIAYFKQLQSPT
NSFILSLAVADCLVGLLVMPYSMIRTVEGCWYFGFLFCKLHSSLDVMLCTASIFHLSCIAFDRYYAV
CNPLVYSLKMSPGRVALLIAVCWAVPMLISFAPIMLNLHIADTDIQIPEDVCLFLVSRIYAVMASLV
AFYLPMAVMLVAYWKIFKAAKRQAKQISAMESQMAAGVGKDSSKKKIHRNTMKREGKAAKTLGIIMG
VFLIFWMPFFTVNIVDPFIEYSTEVVVWDVFLWLGYINSSLNPFLYVFFNSCFRRAFLMFIGCKVCL
PGISPGMELSHSRKEAN*

>Lac-tarl1

MDNSSLELLGDANASLQIELQSCTTLRNQVSRVFLYSFLSVGIVCTVVGNFLVVLSIAYFKQLQSPT
NSFVMSLAVADCLVGLVVMPYSMIRTVEGCWYFGALFCQLHSSLDVMLCTASIFHLSCIAFDRYYAV
CNPLVYSLKMSRNRVALLIVICWAVPMLISFGPIMLDLHIAGVDILLPKDMCLFLVNRIYAIMASLV
AFYLPMAIMLVAYWKIFKAAKRQARQISAMESQMAAGVGKDSSKKQRHRNTMKREKKAAKTLGIIMG
VFLIFWMPFFTVNIVDPFIEYSTEVVVWDIFLWLGYINSSLNPFLYGFFNRSFRRAFLMFMGCRVCL
PGSSPGMELSHTRKDANERADQP*

>Lac-tarl2

MLGSCCIVISIPGTNISENYSLTNDNSDSILFHSTTVTVCVLCLLLPIPIFAIMGNLFIMAAAAHFQ
SLHAPTNALVVSLAVADFLVAVLVIRLGCIFCQVHFMLDLTLCTPSIFNLKTCGHSAGIRLWSSPSF
ASPSACTPRHLLLC*AAACSRIFRPAWPHFMSPLPLQPQLSPPFPWVSCCLRVWEHFHGSHKQARWI
HAIEHHTGQLQMNLSPMRANSTRQVQKKNERKAAKMLGLILGVFLFWWLPFFCMNVVHPLKGYSTSP
LVLEASLWLEYANSSLNPFLSTLFNKSYRHVFVATLDCWIVRRQFRAGLDSSQICVVLILETISR*

>Lb-tarl1

MDNVSLGLVADTNTSLQDELYSCHTLRNQASRIFLYAFLSVGIVCTVVGNFLVVLSIAYFKQLQSPT
NSFVMSLAVADCLVGLVVMPYSMIRTVEGCWYFGVLFCGLHSSLDVMLCTASIFHLSCIAFDRYYAV
CNPLVYSLKMSHCRVTFFIVVCWVVPMLISFGPIMLNLHIAGVNIVLPNDVCVFLVNRVYAVMASLV
AFYLPMAIMLIAYWKIFKAAKRQAMQISAMESQMAAGVGKDSSKKQKHRNTMKRERKAAKTLGIIMG
VFLLFWMPFFTVNIVDPFIDYSTEGVVWDIFLWLGYINSSLNPFLYGFFNRSFRRAFLMFIGCRVCL
PGSSPWMELSHTRKEANERADKR*

>Lc-tarl1

MDNGSSGLLGDVNTSQAELTSCATVRSPACRIFLYAFLSVAIVCTVVGNFLVVLSIAYFKQLQSPTN
SFVMSLAVADCLVGLLVMPYSMIRTVEGCWYFGFLFCRLHSSLDVMLCTASIFHLSCIAFDRYYAVC
NPLLYSLKMPHSRVALLIVVCWAVPMLISFGPIMLDLHIAGVDVILPKDVCVFLVNRIYAVMASLVA
FYLPMAIMLVAYWKIFKAAKRQARQISAMESQMAAGVGKDSSKKQRHRNAMKRERKAAKTLGIIMGV
FLIFWMPFFTVNIVDPFIEYSTEVFVWDIFLWLGYINSSLNPFLYGFFNRSFRRAFLMFMGCRVCLP
GSSPGMELSHTRKDANERADQP*

>Lo-tarl1

MANISEWLDSMNSSGYTFYEPCIPWRSRSSRIILYIFIIAGIICTVFGNFLVVLSIAYFKQLQSPTN
SFVMSLAVADFLVGLIVMPYSMIRTIEGCWYFGPVFCEIHSSLDVMLCTASIFHLSCIAFDRYYAVC
NPLVYGFKMSKTRVTLLIITCWVVPLLISFAPILLGLHKLGIETKLPHDVCYFLVNQVYAITASFVA
FYLPMFIMLVAYWKIYKAAKRQAMQINAVENQMTIQNGAVNSTKKQKQRNSMKRERKAAKTLGIIMG
AFLIFWLPFFTTNVIDPFIEYQTDMVIWEVFLWLGYVNSSLNPFLYGFFNRSFRRAFFMIIGCKICL
PGSPPSIDLSNTKKEGNERTIS*

>Lw-tarl1

MIMGIHLERMCLVYHQVEQHGKLLEWSNISLDADFEACATLRSSGSRVALYVFIIAGIVCTVVGNFL
VVLAIAYFKQLQCPTNYFVMSLAVADFLVGLVVMPYSMVRTVEGCWYFGPTFCHLHSSLDVMLCTAS
IFHLSCIAFDRYYAVCNPLVYSFKMSLKRVGLLIVVCWAVPFLISFGPILLGLHKLGVDVPLPEN!
CIFLVNRVYAVMASLVAFYLPMVTMLVAYWKIYKAAKRQAMQISAMESQMAAGVGKDSSKKQKHRNS
IRRERKAAKTLGIIMGVFLLFWLPFFTVNIVDPFIEYGTAGVIWDVFLWLGYVNSSLNPFLYGFFNR
SFRRAFLMIMGCRICLFGTAQGMDLSHSKRVPVSAQTTSRL*

>Mc-tarl1

MDNSTLGWLGDDNTSLQLELQSCTLLRHQVSRIFLYAFLSVGIICTVVGNFLVVLSIAYFKQLQSPT
NCFVMSLAVADCLVGLVVMPYSMIRTVEGCWYFGSLFCRxxxFNRSFRRAFLMFMGCRVCLPGSSPG
MELSHTRKEANECAEQS

>Mim-tarl1

MDNGSLGLLGDVNTSQAELTSCATVRNQACRIFLYAFLSVAIVCTVVGNFLVVLSIAYFKQLQSPTN
SFVMSLAVADCLVGLLVMPYSMIRTVEGCWYFGVLFCRLHSSLDVMLCTASIFHLSCIAFDRYYAVC
NPLLYSLKMPRSRVALLIVVCWAVPMLISFGPIMLDLHIAGVDIILPKDVCVFLVNRIYAVMASLVA
FYLPMAIMLVAYWKIFKAAKRQARQISAMESQMAAGVGKDSSKKQRHRNTMKRERKAAKTLGIIMGV
FLIFWMPFFTVNIVDPFIEYSTEVFVWDIFLWLGYINSSLNPFLYGFFNRSFRRAFLMFMGCRVCLP
GSSPGMELSHTRKEANERADQP*

>Moa-tarl1

MNNSNLGLLGDANISLQTELQSCTTLRNQASRIFLYTFLSLGIVCTVVGNFLVVLSIAYFKQLQSPT
NSFVMSLAVADCLVGLVVMPYSMVRTVEGCWHFGTLFCQLHSSLDVMLCTASILHLSCIAFDRYYAV
CNPLVYSLKMSHGRVALLIIVCWAVPMLISFGPIMLELHIADVDILFSKDICVFLVNRIYAVMASLV
AFYLPMAIMLVAYWKIFKAAKRQARQISAIESQMAAGVGKDSSKKQRHRNTIKREKKAAKTLGIIMG
VFLIFWMPFFTVNIVDPFIEYSTEGIVWDIFLWLGYMNSSLNPFLYGFFNRSFHKAFLMFMGCRVCL
PGSFPGMELSHTRKEANEHADQP*

>Mom-tarl1

MGGLTTFLLIWGLRGLLRKLFEISHFIKKLSFTTVCFDRFDKTMDNSSLGFPADDKISPQTELKSCS
TLRNEASRIFLYTFLSVGIICTVVGNFLVVLSIAYFKQLQSPTNSFVMSLAVADCLVGFLVMPYSMI
RTVEGCWYFGVLFCRLHSSLDVMLCTASIFHLSCIAFDRYYAVCNPLLYSLKMSRSRIALLVLICWA
VPMLISFGPIMLDLHISGVEVQLPIDVCVFLVNRIYAVIASLVAFYLPSSIMLVAYWKIFKAAKRQA
MQISAMESQMAAGVGKDSSKKQRHRNTMKRERKAAKTLGIIMGVFLIFWMPFFTINIVDPFIDYSTE
LVVWDVFLWLGYINSSLNPFLYGFFNRSFRKAFLMFMRCRVCLPGSSPGMELSHTRKEAN*

>Ms-tarl1

MDNSSLGLLGDVNVNTSLKIELQSCTTLRNQASRIFLYAFLSVGIVCTVVGNFLVVLSIAYFKQLQS
PTNSFVMSLAVADCLVGLLVMPYSMIRTIEGCWYFGVLFCRLHSSLDVMLCTASIFHLSCIAFDRYY
AVCNPLLYSVKMSRSRVALLIVVCWAVPMLISFGPIMLDLHIAGVDVLLPEDVCVFLVNRIYAVMAS
LVAFYLPMAIMLVAYWKIFKAAKRQARQISAMESQMAAGVGKDSSKKQRHRNTMKRERKAAKTLGII
MGVFLIFWMPFFTVNIVDPFIEYSTEVVVWDIFLWLGYINSSLNPFLYGFFNRSFRRAFLMFMGCRV
CLPGSSPGMELSHTRKEANERADQP*

>Mz-tarl1

MDNSTLGWLGDDNTSLQLELQSCTLLRHRVSRIFLYAFLSVGIICTVVGNFLVVLSIAYFKQLQSPT
NCFVMSLAVADCLVGLVVMPYSMIRTVEGCWYFGSLFCRIHSSLDVMLCTASIFHLSCIAFDRYYAV
CNPLVYSLKMSHRRVALLIAVCWLVPILISFGPIMLDLHVAGVDMVIPKDLCVFLVSRIYAVMASLV
AFYLPMAIMLVAYWKIFKAAKRQARQISAMESQMAAGVGKDSSKKKRHRNAMKRERKAAKTLGIIMG
VFLIFWMPFFTVNIVDPFIEYSTEVVLWDLFLWLGYINSSLNPFLYGLFNRSFRRAFLMFMGCRVCL
PGSSPGMELSHTRKEANECAEQS*

>Nb-tarl1

MDNSTLGWLGDDNTSLQLELQSCTLLRHRVSRIFLYAFLSVGIICTVVGNFLVVLSIAYFKQLQSPT
NCFVMSLAVADCLVGLVVMPYSMIRTVEGCWYFGSLFCRIHSSLDVMLCTASIFHLSCIAFDRYYAV
CNPLVYSLKMSHRRVALLIAVCWLVPILISFGPIMLDLHVAGVDMLIPKDLCVFLVSRIYAVMASLV
AFYLPMAIMLVAYWKIFKAAKRQARQISAMESQMAAGVGKDSSKKKRHRNAMKRERKAAKTLGIIMG
VFLIFWMPFFTVNIVDPFIEYSTEVVLWDLFLWLGYINSSLNPFLYGLFNRSFRRAFLMFMGCRVCL
PGSSPGMELSHTRKEANECAEQS*

>Nf-tarl1

MDNNSLQWIQDLNASLQNELQSCSTLRNQISRIFLYTFLSLGVVSTVVGNFLVVLSIAYFKQLQSPT
NYFVMSLAVADCLVGLLVMPYSMMRTVEGCWYFGALFCKLHSSLDVMLCTASIFHLSCIAFDRYYAV
CNPLVYSLKMSPNRVALLIAVCWVIPMLISFGPIMLDLHTADVGIQIPENVCMFLVSRVYAIMASSV
AFYLPMVVMLVAYWKIFKAAKRQAKQISAMESQMASGVGKDSSKKKRHRNTMKREGKAAKTLGIIMG

VFIIFWMPFFTLNIVDPFTDYSTEVVIWDVFLWLGYINSSLNPFLYGLFNSCFRRAFLLLIGCRVCL
PGISSGMELSHSKKETKQ*

>Ol-tarl1

MCVLDRFHLTMDNSSLDWGECTNRSELESCTTMRNQFSRIFLYSFFTIGIVSTVVGNFLVVLSIAYF
KQLQSPTNCFVMSLAVADCFVGLVVMPYSMIRTVEGCWYFGSLFCKLHSSLDVMLCTASIFHLSCIA
FDRFYAVCNPLIYSLKMSQSRVALLITICWAVPMLISFGPIMLDLHIAGVDIRIPEDLCVFLVSRIY
AVMASLVAFYLPMAIMLVAYWKIFKAAKRQALQISAMESQMAAGVGKDSSKKRWHRITMRRERKAAK
TLGIIMGVFLIFWMPFFTLNIVDPFIGYTTEVVVWDIFLWLGYINSSLNPFLYGFFNRSFRRAFLMF
IGCRVCLPGSSPGMELSQTRKEANDCTD*

>On-tarl1

MDNSTLGWLGDDNTSLQLELQSCTLLRHQVSRIFLYAFLSVGIICTVVGNFLVVLSIAYFKQLQSPT
NCFVMSLAVADCLVGLVVMPYSMIRTVEGCWYFGSLFCRIHSSLDVMLCTASIFHLSCIAFDRYYAV
CNPLVYSLKMSHRRVALLIAVCWLVPILISFGPIMLDLHVAGVDMLIPKDLCVFLVSRIYAVMASLV
AFYLPMAIMLIAYWKIFKAAKRQARQISAMESQMAAGVGKDSSKKKRHRNAMKRERKAAKTLGIIMG
VFLIFWMPFFTVNIVDPFIEYSTEVVLWDLFLWLGYINSSLNPFLYGLFNRSFRRAFLMFMGCGVCL
PGSSPGMELSHTRKEANECAEQS*

>Pa-tarl1

MDNSSLGMHDYANTSLQIELQSCTSSKNQVSRIFLYSFFSIGIVCTVVGNFLVVLSIAYFKQLQSPT
NSFVMSLAVADCLVGLVVMPYSMIRTVEGCWYFGSLFCQLHSSLDVMLCTASIFHLSCIAFDRYYAV
CNPLVYSLKMSHSRVALLIVVCWAVPMLISFGPIMLDLHIAGVNILLPKNVCVFFVNRIYAVMASLV
AFYLPMAIMLVAYWKIFKAAKRQARQISAMESQMAAGVGKDSIKKQRYRNTMKRERKAAKTLGIIMG
VFLIFWLPFFTVNIVDPFIEYSTEVVIWDICVWLGYINSSLNPFLYGFFNRSFRRAFLMFIGCRVCL
PGSFPGMDLSHTRKEANECIDQPS*

>Pem-tarl1

MGKKSLCKLLDFSLCSSRFAIMANQTLEMPESLNSTNLAELDSCSTLRNRTFQLQVFLYTFLSLVII
CTVVGNSLVVLSIAYFKRLQSPTNFFVLSLAVADCLVGLVVMPYSMVRTIEGCWIFGAVFCQIHSSL
DVMLCTASIFHLSCIAFDRYYAVCNPLCYSLKMSNNRVLFLIVICWVISLLISFGPVMLELHVASTD
VQIPKDSCVFMVNQIYAVLASVVSFYLPMLTMLLAYWKIYKVARRQARQISAMEGQMGKDTSKKQRH
RNAMKREGKAAKILGIVMGVFLLFWMPFFTINVVDPFIGYRTEAVVWDVFLWLGYLNSSLNPLLYGF
FNRSFRRAFLMFIGCRVCLHQPLSWVELSQTRRDLPNERRN*

>Pf-tarl1

MENSSLAWPGNDNASLPIELPSCATLRSQISRISLYAFLSVGTGCTVVGNFLVVLSISYFKQLQSPT
NSFVLSLAVADCLVGLLVMPFSMIRTVEGCWYFGPLFCRLHSSLDVMLCTASIFHLSCIAFDRYYAV
CNPLVYSLKMSNSHVALLIAICWTVPMLISFGPIMLDLHVADVDIFIPSDVCVFLVSRVYAVMASLV
AFYLPMAVMLVAYWKIFKAAKRQAKQISAMESQMAAGVGKDSSKKKRHRNTMKREGKAAKTLGIIMG
VFLIFWMPFFTVNIVDPFIEYTTEGVVWDVFLWLGYINSSLNPFLYGFFNRCFRRAFLMFLSCRVCL
PGISSGMELSHSKKEKN*

>Pl-tarl1

MENSSLAWPGNDNASLPIELPSCATLRSQISRISLYAFLSVGTGCTVVGNFLVVLSISYFKQLQSPT
NSFVLSLAVADCLVGLLVMPFSMIRTVEGCWYFGPLFCRLHSSLDVMLCTASIFHLSCIAFDRYYAV
CNPLVYSLKMSNSHVALLIAICWTVPMLISFGPIMLDLHVADVDIFIPSDVCVFLVSRVYAVMASLV
AFYLPMAVMLVAYWKIFKAAKRQAKQISAMESQMAAGVGKDSSKKKRHRNTMKREGKAAKTLGIIMG
VFLIFWMPFFTVNIVDPFIEYTTEGVVWDVFLWLGYINSSLNPFLYGFFNRCFRRAFLMFLSCRVCL
PGISSGMELSHSKKEKN*

>Po-tarl1

MDNNSLGFLREANTSLQIELQSCATFRNQVSRIFVYAFLSVGIACTVVGNFLVVLSIAYFKQLQSPT
NSFVMSLAVADCLVGLVVMPYSMIRTVEGCWYFGALFCQLHSSLDVMLCTASIFHLSCIAFDRYYAV
CNPLIYSLKMSHNRVAFLIVVCWAVPMLISFCPIMLDLHIAGVDILLPKDVCVFLVNRIYAVMASLV
AFYLPMAIMLIAYWKIFKAAKRQARQISAMESQMAAGVGKDSSKKQRHRKAMKRERKAAKTLGIIMG
VFLIFWMPFFTVNIVDPFIGYSTEVVVWDVFLWLGYINSSLNPFLYGFFNRSFRRAFLMFIGCRVCL
PGSSPGMELSHTRKEANERAHQP*

>Pom-tarl1

MENSSLAWPGNDNASLPIELPSCATLRSQISRIFLYAFLSVGTGCTVVGNFLVVLSISYFKQLQSPT
NSFVLSLAVADCLVGLLVMPFSMIRTVEGCWYFGPLFCRLHSSLDVMLCTASIFHLSCIAFDRYYAV
CNPLVYSLKMSNSHVALLIAICWTVPMLISFGPIMLDLHVADVDIFIPSDVCVFLVSRVYAVMASLV
AFYLPMAVMLVAYWKIFKAAKRQAKQISAMESQMAAGVGKDSSKKKRHRNTMKREGKAAKTLGIIMG
VFLIFWMPFFTVNIVDPFIEYTTEGVVWDVFLWLGYINSSLNPFLYGFFNRCFRRAFLMFLSCRVCL
PGISSGMELSHSKKEKN*

>Pp-tarl1

MENSTEWSNVSLDADFEACATLRSSGSRAALYVFIVAGIICTVVGNFLVVLAIAYFKQLQCPTNYFV
MSLAVADFLVGLVVMPYSMVRTVEGCWYFGPTFCHLHSSLDVMLCTASIFHLSCIAFDRYYAVCNPL
VYSFKMSLKRVGLLIVVCWAVPFLISFGPIFLGLHKLGVDVPLPENVCIFLVNRVYAVMASLVAFYL
PMVTMLVAYWKIYKAAKRQAMQISAMESQMAAGVGKDSSKKQKHRNSIKRERKAAKTLGIIMGVFLL
FWLPFFTVNIVDPFIEYGTAGVIWDVFLWLGYVNSSLNPFLYGFFNRSFRRAFLMIMGCRICLYGTA
QGMDLSHSKRDASERTENQ*

>Pr-tarl1

MENSSLAWPGNYNTSLPVELPSCATLRSQISRIFLYAFLSVGTGCTVVGNFLVVLSISYFKQLQSPT
NSFVLSLAVADCLVGLLVMPFSMIRTVEGCWFFGPLFCRLHSSLDVMLCTASIFHLSCIAFDRYYAV
CNPLVYSLKMSNSHVALLIAICWTVPMLISFGPIMLDLHVADVDIFIPSDLCVFLVSRIYAVMASLV
AFYLPMAIMLVAYWKIFKAAKRQAKQISAMESQMAAGVGKDSSKKKRHRNTMKREGKAAKTLGIIMG
VFLIFWMPFFTVNIVDPFIEYTTEGVVWDVFLWLGYINSSLNPFLYGFFNRCFRRAFLMFLSCRVCL
PGISSGMELSHSKKEKN*

>Ps-tarl1

MANHTLEMPRSLNSTNLAELDSCSMLRTRTSQVFLYTFLSLVIICTVVGNSLVVLSIAYFKRLQSPT
NFFVLSLAVADCLVGLVVMPYSMVRTIEGCWIFGAVFCQIHSSLDVMLCTASIFHLSCIAFDRYYAV
CNPLCYSLKMSNNRVVFLIMICWVISLLISFGPVMLELHVASTDVQIPKDSCVFMVNQIYAVLASVV
SFYLPMLTMLLAYWKIYKVARRQARQISAMESQMGKDTSKKQRHRNAMKREGKAAKILGIVMGVFLL

FWMPFFTINVVDPFIGYRTEAVVWDVFLWLGYLNSSLNPLLYGFFNRSFRRAFLMFIGCRVCLHQPL
SWVELSQTRRDLPNERRN*

>Pun-tarl1

MDNSTLGWLGDDNTSLQLELQSCTLLRHRVSRIFLYAFLSVGIICTVVGNFLVVLSIAYFKQLQSPT
NCFVMSLAVADCLVGLVVMPYSMIRTVEGCWYFGSLFCRIHSSLDVMLCTASIFHLSCIAFDRYYAV
CNPLVYSLKMSHRRVALLIAVCWLVPILISFGPIMLDLHVAGVDMVIPKDLCVFLVSRIYAVMASLV
AFYLPMAIMLVAYWKIFKAAKRQARQISAMESQMAAGVGKDSSKKKRHRNAMKRERKAAKTLGIIMG
VFLIFWMPFFTVNIVDPFIEYSTEVVLWDLFLWLGYINSSLNPFLYGLFNRSFRRAFLMFMGCRVCL
PGSSPGMELSHTRKEANECAEQS*

>Py-tarl1

MDNGSLGFLGEANTSLQIELQSCATLRNQVSRTFQYAFLSIAITCTVVGNFLVVLSIAYFKRLQSPT
NSFVMSLAVADCLVGLVVMPYSMIRTVEGCWYFGAHFCQLHSSLDVMLCTASIFHLSCIAFDRYYAV
CNPLIYSLKMSNNRVAFLIVVCWAVPLLISFCPIMLDLHIAGVDILLPKDVCVFLVNRIYAVMASLV
AFYLPMGIMLIAYWKIFKAANRQARQISAMESQMAAGVGKDSSKKQRHRNAMKRERKAAKTLGIIMG
VFLIFWVPFFTVNIVDPFIGYSTEGVVWDVFLWLGYINSSLNPFLYGFFNRSFRRAFLMFIGCRVCL
PGSSPGMELSHTRKEANERAHQP*

>Pyn-tarl1

MANSSEWLEDSNTSFVGDFEPCATVRSPSTRVAMYTFIIVGIFCTVVGNLLVVLAIAYFKQLQSHTN
SFVMSLAVADFLVGLVVMPYSMVRTIEGCWHFGATFCQLHSSLDVMLCTASIFHLSCIAFDRYYAVC
NPLVYTFKMSRSRVALLIVVCWAVPLLISFGPILLGLHKLGVDISLPENVCVFLVNRVYAVIASLVA
FYLPMVSMLVAYWKIYKAAKRQAMQISAMEAQMAAGVGKDSSKKQKHRNSMRRERKAAQTLGIIMGV
FLIFWLPFFTVNIVDPFIEYTTTGVIWDVFLWLGYVNSSLNPFLYGFFNRSFRRAFLLIMGCRICLP
GSASGMDLSHSRKDANERTENQ*

>Sa-tarl1a

MENSSEWSNISLDSDFEPCATLRSSGSRVALYVFIIAGIVCTVVGNFLVVLAIAYFKQLQCPTNSFV
MSLAVADFLVGLVVMPYSMVRTVEGCWYFGPNFCHLHSSLDVMLCTASIFHLSCIAFDRYYAVCNPL
VYSFKMSRKRVGLLIVVCWAIPFLISFGPILLGLHKLGMDVPLPENMCIFLVNRIYAVMASLVAFYL
PMVTMLVAYWKIYKAAKRQAMQISAMEAQMAAGVGKDSSKKQKHRNSIRRERKAAKTLGIIMGVFLL
FWLPFFTVNIVDPFIEYGTAAVIWDVFLWLGYVNSSLNPFLYGFFNRSFRRAFLMIMGCRICLHGKA
QGMDLSHSKRDANERTDNQ*

>Sa-tarl1b

MENSSEWSNISLDSDFEPCATLRSSGSRVALYVFIITGIACTVVGNFLVVLAIAYFKQLQCRTNSFV
MSLAVADFLVGLVVMPYSMVRTVEGCWHFGPTFCHLHSSLDVMLCTASIFHLSSIAFDRYYAVCNPL
VYSFKMSRERVGLLIVVCWAIPFLIAFGPILLGLHKLGMDVPLPENVCIFLVNRVYAVMASLVAFYL
PIVTMLVAYWKIYKAAKRQVMQISAMEAQMAADVGKDSSKKQKHRNSIRRERKAAKTLGIIMGVFLL
FWLPFFTVNIVDPFIEYGTAAVIWD

>Sea-tarl1

MDNGTLGLLGDANTSLHIESCTTLRNQVSRIFLYAFLSVGIVCTVVGNFLVVLSIAYFKQLQSPTNS
FVMSLAVADCLVGLVVMPYSMIRTVEGCWYFGVLFCQLHSSLDVMLCTASIFHLSCIAFDRYYAVCN

PLVYSLKMSRSRVALLIVVCWAVPMLISFGPIMLDLHVAGVDILLPKDVCVFLVNRIYAVMASMVAF
YLPMAIMLIAYWKIFKAAKRQAMQISAMESQMAAGVGKDSSKKKRHRNTMKRERKAAKTLGIIMGVF
LLFWMPFFTVNIVDPFIEYSTEVVVWDIFLWLGYINSSLNPFLYGFFNRYFRRAFLMFMGCRVCLPG
TSPGMELSHTRKEANERTDQP*

>Sem-tarl1

MDNGTLGLLGDANTSLHIESCTTLRNQVSRIFLYAFLSVGIVCTVVGNFLVVLSIAYFKQLQSPTNS
FVMSLAVADCLVGLVVMPYSMIRTVEGCWFFGVLFCQLHSSLDVMLCTASIFHLSCIAFDRYYAVCN
PLVYSLKMSRSRVALLIVVCWAVPMLISFGPIMLDLHVAGVDILLPKDVCVFLVNRIYAVMASMVAF
YLPMAIMLIAYWKIFKAAKRQAMQISAMESQMAAGVGKDSSKKKRHRNTMKRERKAAKTLGIIMGVF
LLFWMPFFTVNIVDPFIEYSTEVVVWDIFLWLGYINSSLNPFLYGFFNRYFRRAFLMFMGCRVCLPG
TSPGMELSHTRKEANERADQP*

>Ser-tarl1

MDNGTLGLLGDANTSLHIESCTTLRNQVSRIFLYAFLSVGIVCTVVGNFLVVLSIAYFKQLQSPTNS
FVMSLAVADCLVGLVVMPYSMIRTVEGCWFFGVLFCQLHSSLDVMLCTASIFHLSCIAFDRYYAVCN
PLVYSLKMSRSRVALLIVVCWAVPMLISFGPIMLDLHVAGVDILLPKDVCVFLVNRIYAVMASMVAF
YLPMAIMLIAYWKIFKAAKRQAMQISAMESQMAAGVGKDSSKKKRHRNTMKRERKAAKTLGIIMGVF
LLFWMPFFTVNIVDPFIEYSTEVVVWDIFLWLGYINSSLNPFLYGFFNRYFRRAFLMFMGCRVCLPG
TSPGMELSHTRKEANERADQP*

>Ses-tarl1

MDNGTLGLLGDANTSLHIESCTTLRNQVSRIFLYAFLSVGIVCTVVGNFLVVLSIAYFKQLQSPTNS
FVMSLAVADCLVGLVVMPYSMIRTVEGCWFFGVLFCQLHSSLDVMLCTASIFHLSCIAFDRYYAVCN
PLVYSLKMSRSRVALLIVVCWAVPMLISFGPIMLDLHVAGVDILLPKDVCVFLVNRIYAVMASMVAF
YLPMAIMLIAYWKIFKAAKRQAMQISAMESQMAAGVGKDSSKKKRHRNTMKRERKAAKTLGIIMGVF
LLFWMPFFTVNIVDPFIEYSTEVVVWDIFLWLGYINSSLNPFLYGFFNRYFRRAFLMFMGCRVCLPG
TSPGMELSHTRKEANERADQP*

>Sf-tarl1

MANSSQWWAASEGPNASLHGDVEPCVPWRSPGSRVVLYALLAAGIVSTVVGNFLVVLAIAYFKQLQS
PTNSFVLSLAVADFLVGLVVMPYSMVRTVEGCWYFGSVFCEVHSSLDVMLCTTSIFHLSCIAFDRYY
AVCNPLVYALKMSRTRVALLIVTCWVLPMLISFGPIMLGLHKANMEVVPPEDSCSFLVNRVYAVTAS
LVAFYLPMATMLVAYWKIYKAARRQAMQISAMESQMTTSQGTDAGRKQKQRNSMRRERKAAKTLGFI
MGAFLLFWLPFFTTNIIDPFIEYCTAGVIWDIFLWLGYANSSLNPFLYGFFNRSFRRAFFMIMGCRI
CLPGSPPSADLSYTKRDGQDHPEN*

>Sf-tarl2

NLTEDNFSNCTARHCNSTAMKVCLLLVLVFIAISATLGNLLAVVPVAYFRPLHTPTNVFIVSLAVAD
FLVGALVIPLSLVRSVARWMFRRPFRTAHVLLDVTFCSASIFDLCCIALDRYLAVRNPLRYVLVSVL
VVTLGLRAHTLSEKPGAQAPATCTLMVNPPPTARLPLTPSFILPTSVMVTAYWKIFRAAQKQAQRIS
TMENQVRQMDQDQNRSEAQTSLSARLEMKAVKTLGIIMGAYLLCWLPFFSTNTVYPLHGYHVSSWTI
ELVLRLGYANSALNPILYAFFKTTFRQAFAIILGCGSHGLEQQNSNLSP*

>Sg-tarl1

MENSSEWSNISLNTDFEPCATLRSSGSRVALYVFIIAGIVCTVVGNFLVVLAIAYFKQLRCPTNSFV
MSLAVADFLVGLVVMPYSMVRTVEGCWYFGPNFCHLHSSLDVMLCTASIFHLSCIAFDRYYAVCNPL
VYSFKMSRKRVGLLIVVCWAIPFLISFGPILLGLHKLGMDIPLPENMCIFLVNRVYAVMASLVAFYL
PMVTMLVAYWKIYKAAKRQAMQISAMEAQMAAGVGKDSSKKQKHRNSIRRERKAAKTLGIIMGVFLL
FWLPFFTVNIVDPFIEYGTAAVIWDVFLWLGYVNSSLNPFLYGFFNRSFRRAFLMIMGCRICLHGTA
QGMDLSHSKRDANERTDNQ*

>Sh-tarl1

MANRTMEIPGPFNSTDLLELDCSMLRNYTSQIFLYVFLSFVIVCTVVGNSLVVLSIGYFKRLQSPTN
FFVLSLAVADCLVGLIVMPYSMVRTIEGCWLFGPLFCQIHSSLDVMLCTSSIFHLSCIAFDRYYAVC
NPLCYSLKMSNGRVIFLIISCWGVSSLISFGPVMLELHVAASADIEIPKDSCVFVVNPIYAVLASVV
SFYLPMLTMLLAYWKIYKVARKQARQISAMESQMGKDTSKKQRHRNAMKREGKAAKILGIVMGVFLL
FWMPFFTTNVVDPFIGYRTEAVVWDVFLWLGYLNSSLNPLLYGLFNRSFRRAFLMFIGCRVCLHQPL
SWVELSQTKKDVQNERRN*

>Sn-tarl1

MDNGTLGLLGDANTSLHIESCTTLRNQVSRIFLYAFLSVGIVCTVVGNFLVVLSIAYFKQLQSPTNS
FVMSLAVADCLVGLVVMPYSMIRTVEGCWFFGVLFCQLHSSLDVMLCTASIFHLSCIAFDRYYAVCN
PLVYSLKMSRSRVALLIVVCWAVPMLISFGPIMLDLHVAGVDILLPKDVCVFLVNRIYAVMASMVAF
YLPMAIMLIAYWKIFKAAKRQAMQISAMESQMAAGVGKDSSKKKRHRNTMKRERKAAKTLGIIMGVF
LLFWMPFFTVNIVDPFIEYSTEVVVWDIFLWLGYINSSLNPFLYGFFNRYFRRAFLMFMGCRVCLPG
TSPGMELSHTRKEANERADQP*

>Sr-tarl1a

MENSSEWSNISLDSDFEPCATLRSSGSRVALYVFIIAGIVCTVVGNFLVVLAIAYFKQLQCPTNSFV
MSLAVADFLVGLVVMPYSMVRTVEGCWYFGPNFCHLHSSLDVMLCTASIFHLSCIAFDRYYAVCNPL
VYSFKMSRKRVGLLIVVCWAIPFLISFGPILLGLHKLGMDVPLPENMCIFLVNRVYAVMASLVAFYL
PMVTMLVAYWKIYKAAKRQAMQISAMEAQMAAGVGKDSSKKQKHRNSIRRERKAAKTLGIIMGVFLL
FWLPFFTVNIVDPFIEYGTAAVIWDVFLWLGYVNSSLNPFLYGFFNRSFRRAFLMIMGCRICLHGTA
QGMDLSHSKRDANERTDNQ*

>Sr-tarl1b

MENSSEWSNISLDSDFEPCATLRSSGSRVALYVFIITGIVCTVVGNFLVVLAIAYFKQLQCPTNSFI
MSLAVADFLVGLVVMPYSMVRTVEGCWYFGPTFCHLHSSLDVMLCTASIFHLSCIAFDRYYAVCNPL
VYSFKMSRERVGLLIVVCWAIPFLISFGPILLGLHKLGMDVPVPENVCIFLVNRVYAVMASLVAFYL
PMVTMLVAYWKIYKAAKRQAMQISAMEAQMAAGVGKDSSKKQKHRNSIRRESKAAKTLGIIMGVFLL
FWLPFFTVNIVDPFIEYGTAAVIWDVFLWLGYVNSSLNPFLYGFFNRSFRRAFLMIMGCRICLHGTA
QGIDLSHSKRDANERTDNQ*

>Ss-tarl1a

MLTGAICSVLEMSHHTAKSCQNVHIMDVVCILTEQDQAMNNTSLGWPEDLDGDANLSSLHGDLEDFC
ATSSRHQASRVILYAFFTAGILCTVVGNFLVVLAIAYYKQLQSPTNSFVMSLAVADCLVGLVVMPYS
MVRTVEGCWLFGALFCRVHSSLDVMLCTASIFHLSCIAFDRYYAVCNPLVYHLKMSQGRVAFLIVVC
WAVPLLISFGPIMLGLHKAGVNMVPMPPEDACVFLVNRVYAVMASLVAFYLPMGVMLAAYWKIYKAA

KRQAMQISAMESQFSAGVGKDSSKKQRHRNAMRRERKAAKTLGIIMGVFLLFWLPFFTVNIVDPFID
YSTAVEVWEFFLWLGYVNSSLNPFLYGFFNRSFRRAFMMIMGCRICLSSSSPGMDLSKESNERKVNQ
*

>Ss-tarl1b

MLCVFLTEHDQAMDNTSLGWPEDLDGDVNLSSF*GDLEDFCATSSRHQASRVILYAFFTAGIVCTVV
DNFLVVLAIVYCKQLQSPTNSFVMSLAVADCLVGLVIMPYSMVCTVEGCWFFGALFCQVHSSLDIML
CTASIFHLSCIPFNRYYAMCNPLVYHLKMSQGRVAFLIVVCWAVPLLISFGPIMLGLHKAGVDMVPM
PPEDTCVFLVNRVYTVMASLVAFYLPMGIMLAAYWKIYKAAKRQAMQISAMESQFSAGVGKDSSKKQ
RHRNAMRRERKAAKTLGIIMGVFLLFWLPFFIVNIVDPFIDYSIVVEVWEFFLWLGYVNSSLNPFLY
GFFNRSFRRAFMMVMGCRICLSSSSPGLDLPKESNERKVNQ*

>Ss-tarl2

MWMAGVLSHGVLLSGSNSSTTDYINLTDTGGHNESTSALLRSTAFKVCVLCVLIPIPVFAILGNLLI
VASVGRFRNIQMPTNSFIVSLAMADLLVAVLVMPFSLVRSVDTWWFGRNFCVAHFLLDMTLCTSSIF
NLSCVALDRYVAVCDPLHYPTRMSPRRVTMLLLLSWLLPLLISSLCVSLSMYYLTPPTGYRGTQQES
PTCVAQIHTPYAVADSTVCFFIPVVFMLFAYGRIFMVAQRQARWIHAMENHSGQLHMDQNPTREDPA
RPDPARPDPARSDPARRVQARLGGFSIRKENKAARTLGLIMGVFLMCWLPYFSINIAFPLWGDRISP
IVLEASMWLGYANSSLNPFIYAFFNKDFRHAFVAILGCEILGRQIRGCLVSTQEISRQVHTVVTLET
ISK*

>Stp-tarl1

MDNSSLGWLEDANTSLQIELQPCSTLRNQISRIFLYAFLSVGIVCTVVGNFLVVLSIAYFKQLQSPT
NSFVMSLAVADCLVGLVVMPYSMIRTVEGCWFFGSLFCQLHSSLDVMLCTASIFHLSCIAFDRYYAV
CNPLVYSLKMSSSRVALLIAICWAVPMLISFGPIMLDLHVADVDILLPKDVCVFLVSRIYAVMASLV
AFYLPMAIMLVAYWKIFKAAKRQARQISAMESQMAAGVGKDSSKKKRHRNTMKRERKAAKTLGIIMG
VFLLFWMPFFTVNIVDPFIDYSTEVVVWDIFLWLGYINSSLNPFLYGFFNRSFRRAFLMFMGCRVCM
PGSSPGMELSHTRKEANECADQP*

>Tn-tarl1

MDNSSFELFGVTNSSLLSETPSCVTLRHLVSRIFLYTFFSFGIVCTVVGNFLVVLSIAYFKQLQSPT
NTFVMSLAVADCLVGLLVMPYSMIRTMEGCWYFGLLFCRLHSSLDVMLCTASIFHLSCIAFDRYYAV
CNPLLYSLKMSHGRVALLIFVCWSVPMLISFGPIMLDLHVSGVDIELPIDVCVFLVNRVYAVTASVV
AFYLPSVIMLIAYWKIFKAAKRQAMQISAMESQMAAGVGKDSSKKRRHRNNMKRERKAAKTLGIIMG
VFLIFWMPFFTINIVDPFIEYSTDGVVWDIFLWLGYINSSLNPFLYGFFNRSFRKAFLMLIGCRVCL
PGSSPGMELSHTRREAN*

>To-tarl1

MDNSSLGFHYDANTSLQIELQSCTTLRNQASRIFLYGFFSIGIVCTVVGNFLVVLSIAYFKQLQSPT
NSFVMSLAVADCLVGLVVMPYSMIRTVEGCWYFGDLFCQLHSSLDVMLCTASIFHLSCIAFDRYYAV
CNPLVYSLKMSRNRVALLIVICWAVPMLISFGPIMLDLHIAGVDILLPNNVCIFLVNRIYAVMASLV
AFYLPMAIMLVAYWKIFKAAKRQARQISAMESQMAAGVGKDSSKKQRHRNTMKREGKAAKTLGIIMG
VFLIFWMPFFTVNIVDPFIEYSTEVVVWDIFLWLGYINSSLNPFLYGFFYRSFRRAFLMFMGCKVCL
PGSSPGMDLSHTKKEENEHADQP*

>To-tarl2

MIHIPGVNTSDNNSLTTDGSDSALFHSNTIKVCVLCLLLPIPIFAIMGNLLIMAAVARFQSLRTPTN
AFVVSLAVADFLVAVLVMPFSLVRSIDGWYFGHCFCQAHFLLDMSFCTSSIFNLSCVALDRYIAVCD
PLHYLSRMSPKRVALLLLLCWILPLIISCLCVSFGMYTQSPPAAESSVTQQDTQTCQASFHIPYAFA
TSAISFFIPTGFMLFAYGKIFMAAQRQARWIHAIEHHTGQLQMNQSSMRTDPTRRVHVERYSLKKER
KAAKTLGLIMGVFLLCWLPFFCVNVVHPLKGYSINPLVLEASMWLGYANSSLNPFLYALFNKNYRHA
FVTMLGCGSLGRHLRAGLEYSHFFRQTHTVVTLETISR*

>Tr-tarl1

MNNSSFESSPSETESCLTLRHPASRILLYTFFSVGIICTVVGNLLVVLSIAYFKQLQSPTNTFVMSL
AVADCLVGLLVMPYSMIRTVEGCWYLGLLFCRLHSSLDVMLCTASIFHLSCIAFDRYYAVCNPLLYS
LRMSHSRVALLIFVCWSVPMLISFGPIMLELHIAGVDIQLPKDVCMFLVNQVYAVTASVVAFYFPSA
IMLIAYWKIFKAAKRQAMQISAMESQMAAGVGKDTSRKWQHRNNMRRERKAAKTLGIIMGVFLIFWM
PFFTTNIVDPFIEYGTEGVVWDVFLWLGYINSSLNPFLYGFFNRSFRKAFLMLMGCRVCLTGSSAGM
ELSHTPREAN*

>Xc-tarl1

MENSSLAWPGNDNASLHIELPSCATLRSQISRIFLYAFLSVGTGCTVVGNFLVVLSISYFKQLQSPT
NSFVLSLAVADCLVGLLVMPFSMIRTVEGCWYFGSLFCRLHSSLDVMLCTASIFHLSCIAFDRYYAV
CNPLVYSLKMSNSHVALLIAICWTVPMLISFGPIMLDLHVADVDIFIPSDLCVFLVSRVYAVMASLV
AFYLPMAIMLVAYWKIFKAAKRQAKQISAMESQMAAGVGKDSSKKKRHRNTMKREGKAAKTLGIIMG
VFLIFWMPFFTVNIVDPFIEYTTEGVVWDVFLWLGYINSSLNPFLYGFFNRCFRRAFLMFLSCKVCL
PEISSGMELSHSKKEKN*

>Xm-tarl1

MENSSLAWPGNDNTSLHIELPSCATLRSQISRIFLYAFLSVGTGCTVVGNFLVVLSISYFKQLQSPT
NSFVLSLAVADCLVGLLVMPFSMIRTVEGCWYFGSLFCRLHSSLDVMLCTASIFHLSCIAFDRYYAV
CNPLVYSLKMSNSHVALLIAICWTVPMLISFGPIMLDLHVADVDIFIPSDLCVFLVSRVYAVMASLV
AFYLPMVIMLVAYWKIFKAAKRQAKQISAMESQMAAGVGKDSSKKKRHRNTMKREGKAAKTLGIIMG
VFLIFWMPFFTVNIVDPFIEYTTEGVVWDVFLWLGYINSSLNPFLYGFFNRCFRRAFLMFLSCKVCL
PEISSGMELSHSKKEKN*

TAAR receptors class II from bony fish and cartilaginous fish species

Cartilaginous fish class II TAAR

Callorhinchus milii

>Cm-taarIIa

MINSTSGEKVENVQYCFEFVNDSCPKAIRFIPTCTMLYIFMV!
SIVITVLGNQMVTISISHFRQLHTPTNCLVLSLAIADFLVGLLVMPYSMLRLIETC*YFGNLFCK
IHSSLDMMMSTASIFHLFCIAIDCYYAVFDPLLYTTKITPPVITTFVAVSWVVPTIFAFGVVFSE
INLAGIENLLETSSCMGSCALLFNELSVSSGNRVFIPSIIMLGIYIKRFLVARKHARAIEGVTEK
NNEGKTNRISRNKEHKPAKTLGLVMGIFLIYWVPFFITTIIDPIINFSTPPIIFDTFVWFGYFNS
AFNPILYAFFYPWFHKALKLILSCRLFDSDSSTINLFPK*

>Cm-taarIIb

MQFCFEFVNTSCPKVIRSAAIYTVTYAVIIISTIITIVGNLEVIISIAHFKQLQTPTNYFILLLA
TTHFLIGLIILPYSMVRSVETCWYFGYVFCKIHSSLGMMLTTA*IFHLCFIAVDRYNAMCDPLLY
ITKITLPVLSMFITISWALPRVFAFGIVFSEINLKGMEDYFAAISCHGM!
VLIFNKQWGLLDPVIAFFAPSLVLVGMNIKIFLVARKHSRVTGNKSNKIHSTKENNTRISCRKEH
KNTKTLGIVMGVFLICWLPFFIDTIIDAYIKFTTPPVLVDAFVWFGF!
NSPLNPMIFCFFYPWFRKALKLILSFKIFNIDSFVSNLFPE*

>Cm-taarIIc

MNSINLENSEDLQYCFEFNMSCPKSIRSTTTTVTMYIFITISIVITILGNSVVMISILHFKQLQT
PTNYLVLSLAFVDFLMGFFVLPFSMVRSVETCWYFGDTFCDIHSTLDVVLTTVSIYNLCFIAIDR
YYAVCEPLLYSIKMTLPMTALIITLNWLFAIIYGSCVFLSEFTKKASGHYRTTISCKGSCIEYRF
GGHMDALIVLFIPTFIILGIYLKIYFVQRKHARKIGNMPNNINSKEEINVRVLQTKEKTAAKNQG
VVMGIFVLSWLPFYLSSIINPYLNFATPPILFEAFTWFGFFNSAFNPVLYAFFYPWFRTALKSIL
TCQILRPESSIMNLFPE*

Leucoraja erinacea

>Le-taarIIa

MNLTYLENSEDVQYCFQYVNASCPRVTRPIAIKATLYLIISLSIFISILGNLVVIASVLHFKQLQ
TPTNYLVLSLAVVDFLVASIVLPYSMIRSIETCWYFGHVFCKIHSVLEIVLTIVSIYNVCFIAID
RYYAICDPLLYSIKITVPVTFIILSLIWLFAIYYGFNVVFLDFSKKILSDYVPTMACEGSCIAYA
KFEGHMDSLIIFFIPILIIILGVNIKIFFVVKCKRGRKIGNLPNNDTGEINKETLHNKEQIAVKNQ
SAIMGIFTFSWLPFYVNSILNPYFDSLIPTPLDDVITWFGFFNSTLNPMLYAFLYPWYRRVLKLT
FSCQI

Rhincodon typus

>Rt-taarIIa

MNSTQIQDKADTEDCFEFVNTSCRKNSQSTAVHVALYMIFVIIVLITIISNLLVIISILHFRKLQ
TETNFLVLSLAVTDFLVGLVVLPYSTIRSVDTCWYFESIFCKIHSGLDMVLTITSI!
FIAIDRYYAVCKPLLYPTKITLPVIGLFVTFSCMFSIVYGFALIFSDANVYGIEDYISAISCHGS
CALIFNKLWGHLDPLIAVFVPVSVIVYIYLKIYFVARKHFRAIGNITHRLNSKQQNNTGGFQKKE
FKAAVKLGDVIGIFIICWLPFFIITIVDPYIGFSTPPALFEVFVWLGYFNSTCNPILYGFFYLWF
HKALKMIISCKIFDEHSPMMNVYLE*

>Rt-taarIIb

MQNPICAALVGNLQ*CFKFVNDSCSRAVMFTVTQVTMYIVLITSILITVFGNLMVIISISHFRQF
QTPTM!
FSSAATDFLVGFSVIPYSMVRSIETYWYFGSTFCKIHSSLDMMSTASIFHLCFITIDLYCAVCDP
LHYLTPLVNAIFTTVSW!
IPALFAFGVVFSEMNLWGIQDVIISNSRVGSCVHVFNHQWGILAPTIAFCIPGVIMLGIYINIYF
VARKHAKANENTNKRTNCAEENKRQRSRSKEVKTAKSLGIVIGICWTCWIPFFITPVIDPMINFS
TLPIIFDAFVWFGHFNSAFNPILCALLHPWF*RSLKLMLTWKILDFNSSTINLFLDWLPSEPCKK
NE

>Rt-taarIIc

SLSLILVTVLGNLLVIISISHFKQLHTPTYYLVLSLAVADLLLGLVVLPNCMGYSVETCWYVGNI
LCDVHIRLSVTLALVSINLLGFIAVSHYYAVCDPLLYTTKITLPAIITIIIFIWVFSIIYGFGLV
CSKIIRKGIEDYIRTVSCHGSCILAFNKLWGHLNPLIGFLGPSLTIFDIYIRIFIIARRHARVIR
NMTRKAQSEEEYSNLDFAGKEHNAAMKLSIVIVVYIICWLPYCMIIVVDPYINYSTSRVLFSATL
WFGFINSAFNPILYAFFYPWFCKAVKRILSCKILNLDSSTINLFPDN*

>Rt-taarIId

MNLSDHENSDNVQYCFEFVNTSCARVTRSIAINVALYIFIITSILITIFGNLVVIISVLHFKQLQ
TPTNFLLSSLAVVDFLVGFIVLPYSMVRSVEKCWYFGEVFCKIHSITEIVLTVVSIYTLCFIAID
RYYAMCDPLFYSVKITLPVTVMTIILIWLFAVFYGLSVFLLDFSKKSVDDYVAVRSCEGSCIAYH
KFEGHIDALIVFFIPIFIILGIYVKIFFVTRCKRCRKIENMPNDSHCTEENNVTIMHKKDQIVVR
KQDILIGIFTFSWLPFYVNSILNPYFDFLIPPVLDSVFAWFGFFNSTLNPLLYAFLYPWFRKSLK
LILSCQIFNTDPSTVNFLSE*

Bony fish class II TAAR

TAAR 12

Anguilla anguilla

>Aa-taar12a

MKSLGINLTGDSAETQYCFPYLKGSCPRSQTLIIIKAAMYMFMAFTILLTVCGNLLVIISISHFR
QLHSPTNLLILSLACIDCCLGSFIMPYSMIRSVENCWYFGEIICKMHACFDMMMSIASILHLCLI
SVDRYWAICKPLQYKSLVSMQKVACLIAIIWIFSFGFGFGVLLSKVHLAGMEEFIMMNSCAGTCF
VILNKEWGVVAALVAFFIPGTVMTSLYLKIFYVARSHARKINDSLAVTAEINNHKNRMSEQREKK
AAKTLGIVMGVFLLCWLPFFTTIVVDPFINFSTPNSVFDALVWLGYFNSTCNPLIYGLFYPWFQK
VFKIILSGQVFHRGSSSLNICSEM

>Aa-taar12b

MDSLGINMTGDSAETLYCFPHLERSCPRSQTFIIIKAAMYMFMAFSILMTVCGNLLVIISISHFR
QLHSPTNLIILSLAxSKRPSCIDWFLGAVIMPGSMIRTVENCWYYGEAACIILSSFDMMMSIASI
LHLCLISVDRYCAICKPFWYRSSVTMQKVACVIVIIWTFSFGFGFGVFLSNFPLADLEEFVMASP
CVGNCVLVIKGEWGVGSAVLSFFVPGIVMTSLYLKIFYVARSHARKINDSLALTGAVYNHKDHMS
EQREKKAAKTLGIVMGVFLLCWLPFFLTVIIETLHHFPNIFFVLDLLLWLGYLNSTCNPLIYGFF
YSWFQTVFKMIISGKVFHSGSSSFNIGTDHFK

>Aa-taar12c

MEILALNWTGDSAGTQYCFPHLKGSCLRSQTPIIIKVVMYMLMAVTILLTVCGNLLVIIVISHFR
QLHSPTNLLILSLACIDWFLGAFIMPYSMIRSVENCWYFGETFCKIHSSLDIMMTVASLLHLGLI
SVDRYLSICKPLQYSTSVTTRNVAALVVITWMFSFAFGFGVIHSNINRVGMEKLLMNNSCTGNCT
FFFNKEGGMMAIILGFFIPGSVMIALYMKIFCVAKEQARKIDCSLAMTRARCDKKCPSEHRERKA
AKTLAIALGVFLLCWLPLVMALIIDPFFDFSAPVVLFDAFLWLGYFNSTCNPVIYGFFFSCFQKV
FKIILSGKIFQNGSALLNIYEENY

>Aa-taar12d

VAALIGITWMFSFAFGFGVILSKVNLVGVEELFINSCTGTCILFFNKEGGMMAAFVGFFIPGSVM
IALYMKIFYVAKVQARKIKCSLAITRAQCDKKCTSEHRERKAAKTLAIVLGAFLLCWFPFVMVLI
IDPFFDFSTPAVVYDALVWLGYFNSTCNPLIYGFFYPWFQRVFKIIISGKVFQNGSSLLNIYSEN
Y*

>Aa-taar12e

LAFGFGVILSKVNLVGVEELFIDPCTGSCILFFSKERGMMAAFVAFFIPGTVMIALYMKIFYVAK
VQARKIKCSLAMTRAQCNKNGTSEQTERKAAKTLAIVLGVFLLCCLPFTMLLVVNSFLDYSAPTV
VFDALGWLTYINSTCNPLIYGFFYPWFKRVFKIILSGKVFQNGSSLLNIYEERN*

>Aa-taar12f

MEVLGRNLTGDSAETPYCFPKVKGSCLRSQTLILIKVAVYIFVAFIILMTVCGNLLVIISISHFR
QLHSPTNLLILSLAFTDWFLGGFVMPCSLIRSVENCWYFGETFCKIHSSLDITLGIASLLHLGLI
SVDRYLAICKPLQYRTSVTMHKVAALVGITWMFSFAXXFGVILSEINLVGIEELFINSCTGTCFY
VFNKQGGVIASFVTFFIPGTVMTGLYMKIFHVAKLQARKINCSMAMTRALHEKKDCTSEQKERKA
AKTLSIVLGVFLLCWLPFFLVLIVDPFLDFSTPAVVFDALSWLAYFNSTCNPLIYGFFYPWFRKV
FKMFLSGQIFQNGSSLLNIYAENKL

>Aa-taar12g

LLILSLACIDCCLGSFVMPYSMIRSVESCWYFGEIACKMHSAFDMTMSTASILHLCLISIDRYFA
ICKPLWYRSSVTMNKVTCLIVITWLFSFGFSFGVVFSMVNLVGMEELITMNSCTGNCLLIFNKQW
GVVAALVAFFIPGTVMTSLYLKIFYVARSHAKKINDSLSAPRAVTDKKNHSFEQREKKAAKTLAF
VMGVFVLCWLPFFVTLIVDPFINFSTPLSVADALIWLGYFNSTCNPLIYGFFYPWFQKVFKIIIS
GKVFQNGSSLLSIYIENV

Anguilla japonica

>Aj-taar12a

MKSLGINLTGDSAETQYCFPYLKGSCPRSQTLIIIKAAMYMFMAFTILLTVCGNLLVIISISHFR
QLHSPTNLLLILSLACIDCCLGSFIMPYSMIRSVENCWYFGEIICKMHSSFDMMMSIASILHLCLI
SVDRYWAICKPLQYKSLVSMQKVAWLIAIIWIFSFGFGFGVLLSKVHLAGMEEFIMMNSCAGTCF
VILNKEWGVVAALVAFFIPGTVMTSLYLKIFYVARSHARKINDSLAVTAEINNHKNRMSEQREKK
AAKTLGIVMGVFLLCWLPFFTTIVVDPFINFSTPNSVFDALMWLGYFNSTCNPLIYGFFYPWFQK
VFKIILSGQVFHRGSSSLNICSEM*

>Aj-taar12b

MDSLSINMTGDSAETQYCFPHLERSCPRSQTFIITKVAMYMFMAFSILMTVCGNLLVIISISHFK
QLHSPTNLLVLSLACIDWFLGAVIMPGSMIRTVENCWYYGEAACIILSSFDMMMSIASILHLCLI
SVDRYCAICKPFWYRSSVTMQKVACVIVIIWTFSFGFGFVVFLSNFPLADLEKSVMASPCVGNCV
LVIKGEWGVGSAVLSFFVPGIVMTLLYLKIFYVARSHARKINDSLALTGAVYNHKDHMSEQREKK
AAKTLGIVMGVFLLCWLPFFITVIIETLHHFPNIFFVLDLLLWLGYLNSTCNPLIYGFFYSWFQT
VFKMIISGKVFHIGSSSFNIGTDHFK*

>Aj-taar12c

MEILSLNWTGDSAETQYCFPHLKGSCLRSQTPIIIKAVMYMLMAVTILMTVCGNLLVIIAISHFR
QLHSPTNLLILSLAFIDWFLGAFIMPYSMIRSVENCWYFGETFCKIHSSLDIMMTVASLLHLGLI
SVDRYLSICKPLQYSTSVTTRNVAALVVITWMFSFAFGFGVIHSNINRVGMEKLLMKNSCTGNCT
FFFNKEGGMMAIILGFFIPGTVMTALYMKIFCVAKEQARKIDCSLAMTRARCDKKCPSEHRERKA
AKTLAIALGVFLLCWLPLVMALIIDPFLDFSAPVVLFDAFLWLGYFNSTCNPVIYGFFFSCFQKV
YKIILSGKIFQNGSSLLHIYEENY*

>Aj-taar12d

MGSLGRNLTGDSAGTQYCFPHLKGSCPRSQTLILIKAAMYMFMAFTILMTVCGNLLVIISISHFR
QLHSPTNLIILSLAFIDWFLGTFIMPYSMIRSVENCWYFGETFCKIHSSIDIMMSIASLLHLGLI
SVDRYLAICKPLQYRASVTMHKVAALIGITWMFSFAFGFGVILSKVNLVGVEELFINSCAGTCIL
FFNKEGGMMAAFVGFFIPGSVMIALYMKIFYVAKVQARKIKCSLAITRAQCDKKCTSEHRERKAA
KTLAIVLGAFLLCWFPFVMVLIIDPFFDFSTPAVVYDALVWLGYFNSTCNPLIYGFFYPWFQRVF
KIIISGKVFQNGSSLLNIYSENY*

>Aj-taar12e

MEILARNSTGDSEETQYCFPNLQGSCLRSQKLIFIKAAMYICMGFIILMTVCGNLLVIISISHFR
QLHSPTNLIILSLAFIDWFVGAFVMPYSMIESVENCWYFGETFCKIHSSIDIMMSIASLLHLGLI

SVDRYLAICKPLQYRTSVTMHKVATLIGITWMFSFAFGFGVILSKVNLVGVEELFINSCTGTCIV
FFNKEGGMMAAFVGFFIPGSVMIALYMNIFYVAKVQAWKINCSLAMTRARCDKKCTSEHTDRKAA
KTLAIVLGVFLLCWLPFSMVLVLDPFFDYSTPAVVFDALGWLGYFNSTCNPLIYGLFYPWFRRVF
KIILSGNVFQNGSSLLNIYVERN*

>Aj-taar12f

MKFLSINLTGDSEETKYCFPNLKGSCLRSQKLILIKVAMYFCMAFIILMTICGNLLVIISISHFR
QLHSPTNLIILSLAFIDWFLGAVLMPCSMIRSVEDCWYFGETFCKIHSSVDIMVSIASLLHLGLI
SVDRYLAICQPLQYRTSVTMHKVAALIGIIWMFSFAFGFGVILSKVNLVGVEELFINSCTGTCIV
FFNKEGGMMAAFVAFFIPGIVMIALYMNIFYVAKVQARKIKCSLAMTRARCDKNGTSEQTERKAA
KTLAIVLGVFLLCCLPFSMVLVLGPVFDYSTPAVVFDAVGWLGYFNSTCNPLIYGLFYPWFRRVF
KMILSGKVFQNGSSLLNIYVERN*

>Aj-taar12g

MENMGLNLTGVSAETQYCFPHLNGSCQRSQTLTIIKAAMYMFMACTILMTVFGNLLVIIGISHFR
QLHSPTNLLILSLACIDCCLGSFVMPYSMIRSVESCWYFGEIACKMHSAFDMTMSTASILHLCLI
SIDRYFAICKPLWYRSSVTINKVTCLIVITWLFSFGFSFGVVFSMVNLVGMEELITMNSCTGNCL
LIFNKQWGVVAALVAFFIPGTVMTSLYLKIFYVARSHAKKINDSLSAPRAVTDKKNHSFEQREKK
AAKTLAFVMGVFVLCWLPFFVTLIVDPFINFSTPLSVADALIWLGYFNSTCNPLIYGFFYPWFQK
VFKIIISGKVFQNGSSLLSIYIENV*

>Aj-taar12h

MKIIMQIFVCCRYYLEVSAKMEILALNLTGDSVETQYCFPHLKGSCLRSQTLILIKAAMYMFMAF
IILMTVCGNLLVIISISHFRQLHSPTNLIILSLACIDWFLGAVVMPGSMIKSVENCWYFGETFCK
IHYSTDVMMSTASLLHLGLISVDRYLAICKPLQYRTSVTMHKVAALVGITWMFSFAFGFGVILSK
INLVGVDELLINPCAGTCIMFVNREWGMTAAFVGFFIPGTIMVALYMKIFYVAKIQARKINCSLG
VTRAEQDKKDYSSEQREKKAAKTLGFVLGVYGLCWLPFVMTLVINPSFDLSTSAVVFDALGWLGY
FNSTCNPLIYGFFYPWFQRVFRIILSGKVFQNGSSLLNIYAENNL*

>Aj-taar12i

MEILVHNMTGDSAEMQYCVPHQKGSCLRSQTLIIIKVAMYICMGFIILMTVCGNLLVIISISHFR
QLHSPTNLIMLSLAFIDWFLGAFIMPCSMIRSVENCWYFGETFCKIHSSVDIMMSIASLLHLGLI
SVDRYLAICKPLQYRTSVTMHKVAALIGITWMFSFAFGFGVILSKMNLVGVEELFINACTGTCYL
FFNKQGGLVVAFLGFIMPCTVMVVLYMKILYVAKVQARKINCSLTMTGAKQDKKNCAPEQREKKA
AKTLAIVLGVFLLCWLPIMMVLIVDPFFNFSTPVVVFDGLMWLAYFNSSCNPLIYGYFYPWFRRV
FKIIISGKVFQNGSSLLNIYAENKR*

Astyanax mexicanus

>Am-taar12a

MNLTDAAADNVLLCFPHLPDSCPREHRFLVLKVVMYFLTLGTILMTVLGNLLVIITISYFKQLQS
PTNLIILSMALVDCMLGCLIMPFSLVRWLERCWFLGEIFCQIHSGLDMTLSIASILHLCLVSVDR
YMAICDPLSYKMNVTNFSAAVCIAVIWLFSVTFSFGVVLSKINIVGLEELLMQAACVGSCALIFN
RQWGVIVSLVAFFGPGAVMSALYLKIFHVARKQAKIMSERSTVRQTNSETKAHSSEHRERKAAKT

LGIVMGIFLLCWLPFFIVTITDPFLNFSTPIDVFDALVWFGYLNSTFNPLIYGFFYPRFQRAFKI
IISKVLHLNNANNLLL*

>Am-taar12b

MDSLIMNMTAAENVLLCFPHLPDSCPRAHRFLAFKVIMYFLMMASILMTVFGNLLVIITISYFKQ
LQSPTNLIILSLALVDCLLGCMVMPFSLVRWLERCWFLGEIFCQIHSGLDMTLSIVSILHLCLVS
VDRYMAICKPLSYRMKVTNGSVSVCIAVIWLFSLTYSFGVVLSKVNVIGLENLLVQAPCVGNCAL
IFNLQSGISLALVGFFVPGAVMTTLYLKIFQVARKQAKIMSERATVRQTNSETAVHSSEHRERKA
AKTLGIIMGIFLLCWLPFFLVTLTDPLLGFPTPLDVFDALVWFGYLNSTFNPLIYGFFHPRFQRA
FKIIISKVLHLNNANNLLL*

>Am-taar12c

MDTLFMNKTDVDNVLLCFPNQLDSCPRLQRFSVLKGAMYGFMLVAILMTVFGNLLVIFSISHFKQ
LQSPTNFIILSMALVDCLLGCLVMPFSMVRWVEGCWFLGDFFCQIHSSLDMTLSIASILHLCLVS
VDRYMAICDPLSYRMKVTNGSVAVWIVVTWLFSCLFSFGIVFSKVNLIGLDEQMLNSCVGNCVLI
FNKEWGVIAPLLNFYIPGTIMSCLYLKIFHVARKQAKMISDRTAVGLAGEKNQVSEQRERKAAKT
LGIVMGVFLLCWLPFFLTTVIDPFLGFSTPVDVFDALIWFGYFNSMFNPLIYGFFYPRFQKAFRI
IILRYVCRFTNSSNLVLQ*

Anguilla rostrata

>Ar-taar12a

MKSLGINLTGDSAETQYCFPYLKGSCPRSQTLIIIKAAMYMFMAFTILLTVCGNLLVIISISHFR
QLHSPTNLLILSLACIDCCLGSFIMPYSMIRSVENCWYFGEIICKMHSSFDMMMSIASILHLCLI
SVDRYWAICKPLQYKSLVSMQKVACLIAIIWIFSFGFGFGVLLSKVHLAGMEEFIMMNSCAGTCF
VILNKEWGVVAALVAFFIPGTVMTSLYLKIFYVARSHARKINDSLAVTAEINNHKNRMSEQREKK
AAKTLGIVMGVFLLCWLPFFTTIVVDPFINFSTPNSVFDALMWLGYFNSTCNPLIYGLFYPWFQK
VFKIILSGQVFHRGSSSLNICSEM*

>Ar-taar12b

MDSLGINMTGDSAGTQYCFPHLERSCPRSQTFIIIKAAMYMFMAFSILMTVCGNLLVIISISHFK
QLHSPTNLLVLSLACIDWFLGAVIMPGSMIRTVENCWYYGEAACIIFSSFDMMMSIASILHLCLI
SVDRYCAICKPFWYRSSVTMQKVACVIVIIWTFSFGFGFGVFLSNFPLADLEEFVMASPCVGNCV
LVIKGEWGVGSAVLSFFVPGIVMTSLYLKIFYVARSHARKINDSLALTGAVYNHKDRMSEQREKK
AAKTLGIVMGVFLLCWLPFFLTVIIETLHHFPNIFFVLDLLLWLGYLNSTCNPLIYGFFYSWFQT
VFKMIISGKVFHSGSSSFNIGTDHFK*

>Ar-taar12c

MEILAFNWTGDSAETQYCFPHLKGSCLRSQTPIIIKVVMYMLMAVTILLTVCGNLLVIIAISHFR
QLHSPTNLLILSLACIDWFLGAFIMPYSMIRSVENCWYFGETFCKIHSSLDIMMTVASLLHLGLI
SVDRYLSICKPLQYSTSVTTRNVAALVVITWMFSFAFGFGVIHSNINRVGMEKLLMKNYCTGNCT
FFFNKEGGMMAIILGFFIPGTVMTALYMKIFCVAKEQARKIDCSLAMTRARCDKKCPSEHRERKA
AKTLAIALGVFLLCWLPLVMALIIDPFLDFSAPVVLFDAFLWLGYFNSTCNPVIYGFFFSCFQKV
FKIILSGKIFQNGSSLLNIYEENY*

>Ar-taar12d

SMIRSVENCWYFGETFCKIHSSIDIMMSIASLLHLGLISVDRYLAICKPLQYRTSVTMHKVAALI
GITWMFSFAFGFGVILSKVNLVGVEELFINSCTGTCILFFNKEGGMMAAFVGFFIPGSVMIALYM
KIFYVAKVQARKIKCSLAITTAQCDKKCTSEHRERKAAKTLAIVLGAFLLCWFPFVMVLIIDPFF
DFSTPAVVYDALVWLGYFNSTCNPLIYGFFYPWFQRVFKIIISGKVFQNGSSLLNIYSENY*

>Ar-taar12e

MEILALNLTGDSAETQYCFPHQKGSCLRNQTLILLKVVMYICMAFIILMTVFGNLLVIISISHFR
QLHSPTNLIILSLAFIDWFVGAFVMPCSMIRSVEDCWYFGETFCKIHSSIDIMMSAASLLHLGLI
SVDRYLAICKPLQYRTSVTMHKVAALIGITWMFSFAFGFGVILSKVNLVGVEELFINSCTGTCIV
FFNKEAGMMAAFVAFFIPGTVMIALYMKIFYVAKVQARKINCSLAMTRAQCNKKCTSEQTERKVA
KTLAIVLGVFLLCWLPFSMMLIVDPFFDYSTPAVVFEAFGWLGYFNSTCNPLIYGFFYPWFKRVF
KIILSGKVFQNGSSLLNIY*

>Ar-taar12f

MEILALNFTEDSEETQYCFPNLKGSCLRSQTLILIKVAMYICMGFIILMTVCGNLLVIISISHFR
QLHSPTNLIILSLAFADWFLGAFVMPYTMITYVENCWYFGETFCKIHTSTDITLCVASLLHLGFI
SVDRYLAICKPLQYRTSVTMHKVAALIGIIWMFSFAFGFGVILSQVNLVSGEELFINYCTGSCIL
FFSKEKAMVAVFVAFFIPGSVMLALYMKIFYVAKVQARKINFSLAMTRAQCDKKCTSEHRERKAA
KTLAIVLGAFLLCWFPFVMVLVVDPFFDYSTPTVVFEAFGWLGGFNSTCNPLIYGFFYPWFRRVF
KIIISGKVFQNGSSLLNIYAERN*

>Ar-taar12g1

MEILARNLTGDSEETQYCFPNLQGSCLRSQKLIFIKVAMYICMASISLMTVCGNLLVIISISHFR
QLHSPTNLIILSLAFIDWFLGTFLMPCSMIRSVEDCWYFGETFCKIHTSTDIMLCVASLLHLGLI
SVDRYLAICKPLQYRTSVTMHKVATLIGITWMFSFAFGFGVILSKVNLVGVEELFINSCTGTCIL
FFNKEGGMMAAFVGFFIPGTVMIALYMKIFYVAKVQARKINCSLAMTRARCDKKCTSEHTDRKAA
KTLAIVLGVFLLCWLPFSMVLVLDPFFDYSTPAVVFDALGWLGYFNSTCNPLIYGLFYPWFRRVF
KIILSGKVFQNGSSLLNIYVERN*

>Ar-taar12g2

CWYFGETFCKIHTSTDIMLCVASLLHLGLISVDRYLAICKPLQYRTSVTMHKVAALIGITWMFSF
AFGFGVILSKVNLVGVEELFINSCTGTCIVFFNKEGGMMAAFVAFFIPGSVMIALYMKIFYVAKV
QARKINCSLAMTRARCDKKCTSEQTERKAAKTLAIVLGAFLLCWLPFSMVLVLDPFFDYSTPAVV
FDALGWLGCFNSTCNPLIYGLFYPWFRRAFKIILSGKVFQNGSSLLNIYEERN*

>Ar-taar12h

MKIIMQIFLCCRYYLEVSAKMKILALNLTGDSVETQYCFPHQKGSCLRSQTLILIKAAMYMFMAF
IILMTVCGNLLVIISISHFRQLHSPTNLIILSLACIDWFLGAVVMPGSMIKSVENCWYFGETFCK
IHYSTDVMMSTASLLHLGLISVDRYLAICKPLQYRTSVTMHKVAALIGITWMFSFAFGFGVILSK
INLVGVDELLINPCAGTCIMFVNKEWGMTAAFVGFFIPGTIMVALYIKIFYVAKIQARKINCSLG
VTRAEQDKKDYSSGQREKKAAKTLGIVLGVYGLCWLPFVMTLVINPSFDLSTSAVVFDALGWLGY
FNSTCNPLIYGFFYPWFQRVFRIILSGKVFQNGSSLLNIYAENNL*

>Ar-taar12i

MEILAHNMTGDSAEMQYCVPHQKGSCLRSQTLIIIKVAMYICMGFIILMTVC**GN**LLVIISISHFR
QLHSPTNLIMLSLAFIDWFLGAFIMPCSMIRSVENCWYFGETFCKIHSSVDIMMSIASLLHLGLI
SV**DRYLAICKPL**QYRTSVTMHKVAALIGITWMFSFAFGFGVILSKMNLVGVEELFINACTGTCYL
FFNKQGGLVVAFLGFIMPCTVMVVLYMKILYVANVQARKINCSLTMTGAKQDKKNCAPEQREKKA
AKTLAIVLGVFLLCWLPIMMVLIVDPFFNFSTPVVVFDGLMWLAYFNSSCNPLIYGYFYPWFRRV
FKIIISGKVFQNGSSLLNIYAENKR*

>Ar-taar12j

MEVLGRNFTGDSAETLYCFPQVKGSCLRSQTLILIKVVVYIFVTFIILMTICGNLLVIIAISHFR
QLHSPTNLLILSLAFTEWFLGGFVMPYSMIRSVENCWYFGETFCKIHSSLDITLGIASLLHLGLI
SVDRYLAICKPLQYRTSVTMHKVAALVGITWMFSFASGFGVILSEINLVGIEELFINSCTGTCFY
VFNKQGGVIASFVTFFIPGTVMTGLYMKIFHVAKLQARKINCSMAMTRALHEKKDCTSEQKERKA
AKTLSIVLGVFLLCWLPFFLVLIVDPFLDFSTPAVVFDALSWLAYFNSTCNPLIYGFFYPWFRKV
FKMFLSGQIFQNGSSLLNIYAENK*

>Ar-taar12k

MEIPAPNLTRDSAEIQYCFPNQKGSCLRSQTLILIKVTMYICMAFIILITVCGNLLVIISISHFR
QLHSPTNLIIILSLAFTDWFLGAFIMPCSMIRSVENCWYFGETFCKIHSSVDIMMSIASLLHLGLI
SVDRYLAICKPLQYRTSVTMHKVAALIGITWMFSFAFGFGVILSKVNLIGVEELFINSCTGTCIL
FFNKQGGLMAAFLGFIMPCTVMVVLYMHIFYVAKVQARKINCSLTMTGAKQDKKNCASEQREKKA
AKTLAIVLGVFLLCWLPFVMVLIVDPFFKFSTPAVVFDGLMWLAYLNSSCNPLIYGYFYPWFQRV
FKIIISGKVFQNGSSLLNIYAESK*

>Ar-taar12l

MGSLGLNLTGDSVETQYCFPHQKGSCPRSQTLILIKAAMYMFMAFTILMTVFGNLLVIISISHFR
QLHSPTNLIILSLAFIDWFLGTFIMPYSMIRSVENCWYFGETFCKIHSSIDIMMSIASLLHLGLI
SVDRYLAICKPLQYRTSVTMHKVAALIGITWMFSFAFGFGVILSKMNLVGVEELFINACTGTCYL
FFNKQGGLVVAFLGFIMPCTVMVVLYMKILYVANVQARKINCSLTMTGAKQDKKNCAPEQREKKA
AKTLAIVLGVFLLCWLPIMMVLIVDPFFNFSTPVVVFDGLMWLAYFNSSCNPLIYGYFYPWFRRV
FKIIISGKVFQNGSSLLNIYAE

Cyprinus carpio

>Cc-taar12a

MIANETDIENVFLCYPLRPDSCPSAHRLTVLKVVMYVFMLLMILTTVFGNLLIIISISHFKQLQS
PTHLIVQSLAACDCLLGSLVMPYSMVRSVEGCWYLGDVVCKVHSSLDMTFSISSILHLSLIAIDR
YWAICDPLRYRMRVTNNTVTVFITFTWLFSFVYSFSVVFTGVNNVGLEELILQISCFGGCVLFFN
KEWGLICALFVFLIPGTIMSSLYMSIFNVVKKHVKVLSEKVSVAPTVGSNCQTSSQRESKAAKTL
AIVMGVFYLCWLPFFTATAIDPFLNFVTPVDVFDALVWFGYFNSTCNPLIYGFFYPRFQKAFKIL
ISTYICGISKSHTLTFQ*

>Cc-taar12b

MTLNETGIYPENVFLCYPLLPDSCPRAHRLPALKVAMYVFMVLMILTTVFGNLLIIISISQFKQL
QSPTHLIVRSLAASDCLLGSLVMPYSMVRSVEGCWYLGDVVCKVHSSLDMTFSISSLIHLSLISV

DRYMAICDPLRYKMRVTNNIVSVFTTFTWLFSFLYSFSIVFSGVNKIGLESFIMQVYCVGSCVLF
FNKQWGLICSLLTFFLPGTIMSSLYMEIFHVARKHAKVMSERVTVMATGGLKIQSSTEREGKAAK
TLAIVMGVFYFCWLPFFTATAVDPFLNFVTPGDVFDALVWFGYFNSTCNPLIYGFFYPRFQKAFK
ILISTYICGCNDSSTLILE*

>Cc-taar12c

MCISFFGYWSLTNCTIFIILSLFMITKKIFLASRSNVMTLNKTDIYFENMFLCYPLLPNSCPKLH
RLTVVKVAIYIFMVLMILTTVFGNLLIIISISHFKQLQSPTHLIVRSLAASDCLLGSLVMPYSMV
RSVEGCWYLGDVVCTVHSSLDMSFCISSLLHLSLVSVDRYMAICDPLRYRMRVTNNTVTAFITFI
WLFSIFYSFFIVFSGVNKIGLESFIMQVYCVGSCVLFFNKQWGIICPILTFFLPGTIMSSLYMKI
FYVAQKHAKVMSERVTVMAAVELKSQSSVHRERKAAKTLAIVMGVFLFCWLPIFIATIIDSFLNF
VTPASVFDALVWFGYFNSTCNPLIYGFFYSRFQKAFKILISSCIYGFSDSITLTFE*

>Cc-taar12d

MTSNETDIVNMLLFYPLWSDSCLKLHHLAVVKVAMYVLIVLMILTTVFGTC*SLISISHFK!
TSFKCHPTHHRMYRLSELPATVSSVEGCWNLGDVVCKVHSSLDMSFCISSLLHLSLISVDRYTAI
CDPLRYKMRIKNNKVTALISFIWVFSLVYSFYVVFSGINRIGLESFFIQVYCMGSCVLFLNKEWG
IMRPILVFILPKTIMNSLYIKIFHVARKHTKVMSERVTGRLKSQSSAHRERNAAKALAIVIGVFC
SAGFLFFFYCYCTYFFTPAVVKVILWVRLPFH*

>Cc-taar12e

MTSNETQTENILLCYPLRPDSCPKLHCLTVVKVAMYVLMLLMILTTVFGNLLIIISISHFKQLQS
PTHLIVRSLAASDCLLGSLVMPYSMVRSVEGCWYLGDFVCKVHSSLDMSFCISSLLHLSLISVDR
YWAICDPLRYKMRVTNNTVTVFTTFIWLFSFLYSFYVVFSGVNTIGLELFIMQVYCVGSCVLFFN
KQWGLICPILTFFLPGTIMSSLYLKIFHVARKHAKVMSESVTGGLKSQSSAHRERKAAKTLAIVM
CVFLFCWLPYFTVTALDPFFNFFTPAVVFDALIWFAYLNSTCNPLIYGFFYPCFQSAFKILISTY
ICGNKDPNTTTFK*

>Cc-taar12f

MTSNETDTQIVFLCYPLRPDSCLKVPRYVMVKVAMYAFLVLMILTTVFGNLLIIISISHFKQLQS
PTHLIVRSLAASDCLLGSLVMPYSMVRSVEGCWYLGDFVCEVHSSLDMTFCISSILHLSLISVDR
YWAIFDPLRYKIRVTNNTVTVFTTFICLFSFLYSFSIVFSGVNKIGLESFIMQFYCVGSCVLVFN
KQWGIICPILTFLLPGMIMSSLYIKIFYVARKHAKVMSERVTGGLKSQSSAHRERKAAKTLAIVM
CVFLFCWLPNFTAAALDPFFNLWTPAAVFDALFWFAYFNSTCNPLIYGFFYPCFQKAFKILISTY
ICGIKHSNTLIF

>Cc-taar12g

MTSNDNQTENMFLCFPLRPDSCLKLHRLTVVKGAMYVLMVLMILTTVFGNLLIIISISYFKQLQS
PTHLIVRSLAASDCLLGSLVMPYSMVRSVEGCWYLGDFVCEVHSSLDMSFCISSILHLSLISVDR
YMAICDPLRYRMRVTNNTVTAFIIFIWLFSFVYSFSVVFSGITADGLEMLILQTYCVGSCVVFFN
KQWGIVCPILTFFLPGMIMSSLYLKIFYVAQKHAKVMSERVTGGMKSQSSAHRERKAAKTLAIVM
GVFLLCWLPYFTAAALDPFLNFWTPAVVFDALFWFAYFNSTCNPLIYGFFYPCFQKAFKILISTY
ICGNKHSNTLIFE*

>Cc-taar12h

MTSNKTQTENMFLCYPLRPNSCPKLHRLTVVKVAMYSFLVLMILTTVFGNLLIIISISQFKQLQS
PTHLIVRSLAASDCLLGSLVMPYSMVRSVEGCWYLGEFLCKVHSSLDMTFCMSSLLHLSLISVDR
YWAICDPLRYRMRVTNNTVTVFITFIWLFSFVYSFSVVFSGISAVGIEMVILQSYCVGNCVVFFN
KEWSIICPVLVFFLPGMIMSSLYVKIFYVARKHAKVMSERVTGGLKSQSSAHRERKAAKTLAIVI
GVYLFCWLPYFTAAVLDPFFYFWTPAVVFDALFWFAYFNSTCNPLIYGFFYPCFQKAFKILISTY
ICGIKHSNTLTFE*

>Cc-taar12i

MTSNLTQTENMFLCFPLRPNSCPKLHRLTVVKVAMYAFFVLMILTTVFGNLLIIISISHFKQLQSPTHLIV
RSLAASDCLLGSLVMPYSMVRSVEGCWYLGEFLCKVHTNLDMTFSMSSLLHLSLISVDRYWAICDPLRYKM
RVTNNTVTVFIAFIWLFSFVYSFSIVFSGISAVGIEMVILQSYCVGNCVMFFNKEWSIICPVLVFFLPGTI
MSSLYVKIFYVARKHAKVMSERVTGGLKSQSSAHRERKAAKTLAIVMGVYLFCCLPYFTASVLDPFFNYWT
PAVVFDALFWFAYFNTTCDPLIYGFFYPCFQKAFKILISTYFCGIKHLNTLIFVMNTALTLISGITINIYF
HVILFFSYMLKPFCKRVLLEAVLIFLLI*

>Cc-taar12j

MMTSNKTQTENMFLCYPLRPNSCPKLHHLTVVKVAMYAFFVLMILTTVFGNLLIIISISHFKQLQ
SPTHLIVRSLAASDCLLGSLVMPYSMVRSVEGCWYLGEFLCKVHSSLDMTFSMSSLLHLSLISVD
RYWAICDPLRYKMSVTNNTVTVFITFIWLFSFVYSFSVVFSGIGAVGLEMLILQSYCVGNCVVLF
NKEWSIICPVLVFFLLGMIMSSLYVKIFYVAQKHAKVMSERVTGGLKSQKSSV*

>Cc-taar12k

MTSNKTQTENMFLCYPLRPNSCPKTTRLTVVKVAMYAFFVLMILTTVFGNLLIIISISQFKQLQS
PTHLIVRSLAASDCLLGSLVMPYSMVRSVEGCWYLGEFLCKVHSSLDMTFCMSSLLHLSLISVDR
YWAICDPLRYKMRVTNNTVTVFITFIWLFSFVYSFSVVFSGINTVGLEMVILQSYCVGNCVVFFN
KEWSIICPVLVFFLPGTIMSSLYVKIFYVAQKHAKVMSERVTGGLKSQSSAHRERKAAKTLAIVM
GVFLFCWLPFFTASVLDTFLNFWTPAVVFDALFWFAYFNSTCNPLIYGFFYPCFQKAFKILISTY
ICGIKQSNTLIFE*

>Cc-taar12l

MMTSNKTQTENMFLCYPLRPNSCLKLHRLTVVKVAMYAFFVLMILTTVFGNLLIIISISQFKQLQ
SPTHLIVRSLAASDCLLGSLVMPYSMVRSVEGCWYLGDFLCKVNSSLDMTFCMSSLLHLSLISVD
RYWAICDPLRYKMRITNNTVTVFTTFIWLFSFVYSFSVVFSGISAVGLEMVILQRYCVGSCVVLF
NKEWSIICPVLIFFLPGTIMSSLYVKIFYVAQKHAKVMSERVTGGLKSQSSAHRERKAAKTLAIV
MGVFLFCWLPFFTASVLDTFLNFWTPAVVFDALFWFAYFNSTCNPLIYGFFYPCFQKAFKILIST
YICGIKQSNTLIFE*

>Cc-taar12m

MTSNKTQTENMFLCYPLRPNSCPKLHRLTVVKVAMYAFFVLMILTTVFGNLLIIISISQFKQLQS
PTHLIVRSLAASDCLLGSLVMPYSMVRSVEGCWYLGDFLCKVNSSLDMTFCMSSLLHLSLISVDR
YWAICDPLRYKMRITNNTVTVFTTFIWLFSFVYSFSVVFSGISAVGLEMVILQRYCVGSCVVLFN
KEWSIICPVLIFFLPGTIMSSLYVKIFYVAQKHAKVMSERVTGGLKSQSSAHRERKAAKTLAIVM
GVYLFCWLPYFTAAVLDPFFNYWTPAVVFDALFWFAYFNSACNPLIYGLFYSCFQKAFKILISTY
FCGIKHSNTFIFE*

>Cc-taar12n

MTSNETNTQIVFLCYPLWPDSCLKVQRNVVVKVAMYVFLLLMILTTVFGNLLIIISISHFKQLQS
PTHLIVRSLAASDCLLGSLVMPYSMVRSVEGCWYLGEFLCKVHSSLDMTFCMSSLLHLSLISVDR
YWAICDPLRYKMRVTNNTVTVFTTFIWLISFLYSFTVVFSGISAVGLEMLILQTYCVGNCVVFSN
KQWGLICPIFTFFLPGMIMSSLYMKIFHVARKHAKVMSERVTGGLKSQSSAHRERKAAKTLAIVM
GVYLFCWLPYFTAAVLDPFFNYWTPAVVFDALFWFAYFNSACNPLIYGLFYSCFQKAFKILISTY
ICGIKHSNTLIFKRILLSHLFQASQ*

>Cc-taar12o

MISKKMISVSGADVMTSNETQTENILLCYPFQSDSCPKLHRLTVVKVAMYVFMVLMILTTVFGNL
LIIISISHFKQLQSPTHLIVRSLAASDCLLGSLVMPYSMVRSVEGCWYLGDFLCKVHSSLDMTFC
ISSLLHLSLISVDRYWAICDPLRYKMRVTNNTVTVFTTFIWLISFLYSFTVVFSGISAVGLEMLI
LQTYCVGNCVVFSNKQWGLICPIFTFFLPGMIMSSLYMKIFHVARKHAKVMSERVTGGLKSQSSA
HRERKAAKTLAIVIGVYLCL*

Ictalurus punctatus

>Ip-taar12a

MEWIGSLITNATHANVDTALLCFPHEPDSCLRTARFVVIRFVMYILMLPTILMTVFGNMLVIITI
SYFKQLHSPTNFIILSLAVVDCLLGSMIMPFSMVRWVEGCWFLGEIFCKIHSSLDMTLSIVSILH
LCLVSIDRYMAITDPLSYKMKVTNDRVVVGIAVIWLFSFSFSFGVVLSNVNLDGLEDLLMLSSCV
GNCALIFNRQWGVIAALVAFFVPGTIMSCLYLKIFHVARKQAKVMAERTTIGNCETKNRSSEQRE
RKAAKTLGIVMGIFLLCWLPFFIMTIADPFLNFSTPVDVFDVLVWFGYLNSTCNPLVYGFFYPRF
QKAFKMIISKYVFHLNDSGNLTL*

>Ip-taar12b

MGTLFMNNTDVEILLLCFPHQPDSCLKTTRFFVIRVIMYFLTLTAIVMTVFGNLLVIITISYFKQ
LHSPTNFIILSLAFVDFMLGCLVMPFSMVRWVEGCWFLGDLFCQIHSSLDMTLSISSILHLCLVS
IDRYMAITDPLGYKLKVTNGNTAVCIAVVWIFSGMFSFGIVFSKINIAGLDEQMLNTCVGNCVLI
FNKEWGIIAPLLNFYIPGAIMTCLYLKIFHVARKHARLISDRTAGLTSGETKKQVSDQRERKAAK
TLGIVMGVFLLCWLPFFLITVIDPFLNFSTPLDVFDALIWFGYFNSMFNPLIYGFFYPRFQKAFK
IIIVRYLFHLKSSSNLVLQ*

Lepisosteus oculatus

>Lo-taar12a

MNSTEVIEEMQYCFQHLKGSCPKVRRLAVIKVAMYVFMAGTIFVAVCGNCMVIISISHFKQLHSP
SNFLILSLGCVDCLLGSLIMPYSMIRSVESCWYFGEIFCAIHSSLDMMMSIASILHLGFIAADRY
FAICDPLRYRNKMTMFNITLFIVISWLFSFAFGFGVVLSKVNLAGIEEFVVSSSCVGACIVILNK
EWGMLSASVAFIIPGTVMISLYIKIFFVARRHARVINAAAPVDDKSSIADKREKKAAKTLGIVIG
VFLLCWLPFFITIIIDPFIDFSTPVIVFDTLMWLGYFNSACNPMIYGFFYPWFQKAFKIVVTGKV
FCVGSSSINIFTDKY*

>Lo-taar12b

MNSTTEFEEMQYCFQYLEGSCPKAHRTAVIKVAMYIFMVGTMLATVSGNLMVIISIAHFKQLHSP
TNFLTLSLGCADCLLGAFVMPYSMVRSVETCWYFGDLFCKIHSSLDMTISVASILHLTFIAVDRY
VAICDPLRYRSKITTFVVAIFIGISWIYSLGLGFGVVFSKINLTGIEEFVILNSCVGTCFLIFNK
HWGVLAALLAFLIPGTVMISLYVKIFMVASRHVRVLNDTSGKICARNDHKRKIVENRERKAAKTL
GIVMGVFLICWLPFFMATIIDPFIGFSTPVILFDALVWFGYFNSTCNPVIYGFFYPWFQKAFKII
ITGKVFCYNSSSTHLFTDKH*

>Lo-taar12c

MEFLVDHGLKRFIMNSTNEEMEYCFQHVKGSCLKARRLAAIKGAIYTFVLGTILVTVCGNLMVII
SIAHFKQLHSPTNFLILSLGCVDCLLGVLVMPYSMVRSVETCWYFGDLFCKIHSSLDMTISVASI
LHLNCIAVDRYFAICNPLRYQNIITTLVVAIFIGIVWLYSLLFGFGLVFFKGRLASTDELIISNS
CVGSCFIQFDKHWAVLGPLLVFFLPAVVMISLYVKILIVASRQAKVIKEISGTVVYQNGNKNKIK
ANRERKAVKTLSIVMGIYLLCYVPFSVTTVTDLFLIFSPPVVIFDTLIWLGYFNSTCNPIIYAFF
YPWFQKAFKIIITGNVFHFGSSSINLFSDKY*

Leuciscus waleckii

>Lw-taar12a

MGMYYIYVNLSLDIGLYQPIIYTIILLILCLITIKEMISASGVDVMMSNVTDSENVFLCYPLRLD
SCPKVHRLTVVKVAMYVFMVLMILTTVFGNLLIIISISH!
KHLQSPTHLIVRSLAASDCLLGSLVMPYSMVRSVEGCWYLGDFVCKVHSSLDLTFCISSI!
HLSLISIDRYWAICDPLRYKMRVTNNTVTVFTTFTWLFSFVYSFSIVFTGINAVGLEMLILQVYC
VGSCVLFFNKQWGLIAPILTFFLPGAIMSSLYMKIFHVARKHAKVMSERVTVAAAGGLKSQSSAQ
RERKAAKTLAIVMGVFLFCWLPFFTVAALSSFFTLLTPADFFDALVWFAYFNSTCNPLIYGFFYP
CFQNAFKILISVASSIQTP*

Pimephales promelas

>Pp-taar12a

VAMYVFMVLMILTTVFGNLLIIISISHFKHLQSPTHMIVQSLAACDCLLGSLVMPYSMVRSVEGC
WYLGDFVCKVHSSLDMTFSISSILHLSLVSIDRYWAICDPLRYKMRVTNNTVAVFITFTWLFSFV
YSFSVVFTGVNNVGLEELLLQISCFGGCFLLFNREWGLICALFVFLIPGTIMSSLYLSIFNVVKK
HAKVMSEKVSVAPTVGGNCQTSSQRERKAAKTLAIVMGVFYLCWLPFFTATAVDPFLNFVTPGDV
FDALVWFGYFNSTCNPLIYGFFYPRFQKAFKILISTYICGISESHILTFE*

>Pp-taar12b

DCLLGSLVMPYSMVRSVEGCWYLGDFVCKVHSSLDMTFSISSKIHLCLISVDRYWAICDPLMYKM
RVTNNIVTVFTTFTWLFSFLYSFSIVFSGVNTIGLESFIMQVYCVGSCVLFFNKQWGLICSLLTF
FLPVATMSSLYMKIFHVARRHAKVMSERVTVVTVGGLKSQSSAQREGKAAKTLAIVMGVFCICWL
PFFTATAVDPFLNFVTPVDVFDALVWFGYFNSTCNPLIYGFFYPRFQKAFKILISTYICGFKSSN
ALILE*

>Pp-taar12c

MITKKIILGFGTNVMTSNITDTKNMFLCYPLLLNSCAKSHRLTVVRVAMYVFMVLMILTTVFGNL
LIIISISHFKHLQSPTHLIVRSLAASDCLLGSLVMPYSMVRSVEGCWYLGDFVCKVHSSLDMTFC
ISSILHLSLVSIDRYWAICDPLRYKMRVTNNSVAVFITFTWLFSFLYSFSIVFSGVNKIGLESFI
MQVYCVGSCVLFFNKQWGLICPILTFFLPGMIMSSLYMKIFHVARKHAKVMSERVTVVTAGGLKS
QSSAQREGKAAKTLAIVMGVFYLCWLPIFIATIIDSSLNFVTPASVFDALVWFGYFNSTCNPLIY
GFFYSRFQKAFMILISSRIYGFSDSRTLTFE*

>Pp-taar12d

MYYINVNLSLDIGFYQAIVYKIILLILCLIKMKETISAYGADVMTSNETDSENMFLCYPLLPDSC
PKVHRLTVVRVAMYVFMVLMILTTVFGNLLIIISISHFKHLQSPTHLIVRSLAASDCLLGSLVMP
YSMVRSVEGCWYLGDFVCKVHSSLDMTFCISSILHLSLVSIDRYWAICDPLRYKMRVTNNIVTVF
TTFTWLFSFVYSFYVVFSGINAVGLEMLILQIYCVGSCVLFFNKQWSLTCSILTFFLPGTIMSSL
YMKIFHVARKHAKVMSERGLKSQRSAQREGKAAKTLAIVIGVFYVCWLPYFTVTAVDPFYNYLTP
ADVFDVVIWFAYFNSTCNPLIYGFFYPCFQNAFKTLISTYVCGVNDSNTLTLNE*

>Pp-taar12e

MYYINVNLSLDIGLYQAIIYKIILLILCLIKIISAYGADLITSNETDSENMFLCYPLLPDSCPKVHRLTVV
KVAMYVFMVLMILTTVFGNLLIIISISHFKHLQSPTHLIVRSLAASDCLLGSLVMPYSMVRSVEGCWYLGD
FVCKLHFSLDMTFCISSILHLSLVSIDRYWAICDPLRYKLRVTNNIVTIFTTFTWLFSFVYSFYVVFSGIN
AVGKMLILQISCVGSCVLFFNKQWGLICTVLLFFLPGMIMSSLYMKIFHVARRHAKVMSERVTVVTAGGLK
SQRSAHRERKAAKTLAIVMGVFYFCWLPYFTVTALDPFFNYLTPVDVFDALVWFAYFNSTCNPLIYGFFYP
CFQNAFKILISTYVCGINDSNTLTLNK*

Pygocentrus nattereri

>Pyn-taar12a

MNVLNENMTEADVENVLLCFPHQPDSCPRAHRFLALKVPMYFLMLASILMTVFGNLLVIITISHF
KQLHSPTNLIVLSLALVDCLLGCLVMPFSMVRWLERCWFLGDVFCKIHSSLDMTLSIVSILHLSL
VSIDRYLAICEPLSYRMRVTNGTVAVCIAFIWLFSLTFSFGVVLSEVNITGLENLLMLNSCGGNC
ALIFNKQWGVIAALVAFFIPGTIMSSLYLKIFHVARKQAKVMSERATVRRTSCEMNVQSSEQRER
KAAKTLGIVMGIFLLCWLPFFIATIIDPFLDFSTPVDVFDALVWFGYLNSTFNPLVYGFFYPRFQ
SAFKIIISKYVLHLRNASQLVL*

>Pyn-taar12b

MNVLNENMTEADVENVLLCFPHQPDSCPRAHRFLALKVAVYVLLLATILMTVFGNLLVIITISHF
KQLHSPTNLIVLSLALVDCLLGCLIMPFSMVRWLERCWFLGDVFCKIHSSLDMTLSIVSILHLSL
VSIDRYLAICEPLSYRMRVTNGTVAICIAFIWLFSLTFSFGVILSEVNITGLENLLILSSCVGNC
ALIYNKESGFILSFVAFFIPGAIMISLYLKIFHVVRQQAKVMSERATVRRTSCEMNVQSSEQRER
KAAKTLGIIMGIFLLCWLPFFIVTLTDPFQNFSTPLDVFDALVWFGYLNSTLNPLVYGFFYPRFQ
SAFKIIISKYVLRLSNANRLVL*

>Pyn-taar12c

MNVLNENMTEADVENVLLCFPHQPDSCPRAHRFLALKVAVYVLLLATILMTVFGNLLVIITISHF
KQLHSPTNLIVLSLALVDCLLGCLVMPFSMVRWLERCWFLGDVFCKIHSSLDMTLSIVSILHLSL
VSIDRYLAICEPLSYRMRVTNGTVAICIAFIWLFSLAYSFGVILSEVNITGLENLLILSSCVGNC

VLIFNKENAFILSFVLFFIPGTIMSSLYLKIFHVVRQQAKVMSERATVRRTSCEMNVQSSDHRER
KAAKTLGIIMGIFLLCWLPFFIVTLSDPFRNFSTPLDVFDALVWFGYLNSTLNPLVYGFFYPRFQ
RAFKIIISKYVLHLGNASQLVL*

>Pyn-taar12d

MDSFIANMTEADVKDVLLCFPHQPDSCPRVHRFSVLKFMMYAFMLVTIFMTVFGNLLVIITISHF
KQLHSPTNLIILSLALVDCMMGCLVMPFTMVRWVEGCWFLGDFLCQIHSSLDMTISIASILHLCL
VSVDRYVAICEPLQYRMKVTNSKIAVCLATIWLFSLSFGFGIVLSKVNALGLDTVVTPCVGYCAL
TFNKEWSVIGSLLNFFIPGTIMSCLYLKIFCVARKQAKVMSERTAVVKPCETSHSSEQRERKAAK
TLGIVMGFFLLCWLPFFMANLIDPFLNFMTPVDVFDALIWLAYFNSMCNPLIYGFFYPRFQRAFK
IIISRYVLRLSDANNLIL*

>Pyn-taar12e

MDMLFVNKTDVENVLLCFPHQPDSCAGKHRFLVLRVAMYVCMLLAILMTVFGNLLVIVSISHFKQ
LHSPTNFIILSLAWVDSLLGCLVMPFSMVRWVEGCWLLGELFCQIHSSLDMTLSIASILHLCLVS
IDRFVAICEPLSYRMKVTNANVAVCIAVIWLFSCVFSFGIVFSKVNVMDLDEHMLNPCVGNCALV
FNKEWGVIAPLLNFYIPGTIMSCLYLKIFYVARRQAKVISVGTAVTSGEKSQVSEQRERKAAKTL
GIVMGVFLLCWLPFFLTTVIDPFLGFSTPVEVFDALVWFGYFNSMFNPLIYGFFYPRFQKAFKII
ISRSVCRSTNSNFVLQ*

Sinocyclocheilus anshuiensis

>Sa-taar12a

MTSNETDIYPENVFFCYPLLPDSCPRAHRLPALKVAMYVLMVLMILTTVFGNLLIIISISHFKQL
QSPTHLIVRSLAASDCLLGSLVMPYSMVRSVEGCWYLGDFVCKVHSSLDMTFSISSLIHLSLISV
DRYWAICDPLRYKMRVTNNIVTVFTTFTWLFSFLYSFSIVFSGINKIGLESFIMQVYCVGSCVLF
FNKQWGLICSLLTFFLPGTIMSSLYMNIFYVARKHAKVMSERVTGGLKSQGSAQREGKAAKTLAI
VMGVFYLCWLPFFTATAVDPFLNFVTPGDVFDALVWFGYFNSTCNPLIYGFFYPRFQKAFKILIS
TYTCGCNDSSTLILE*

>Sa-taar12b

MNLVNVITVDFQKLSG**I**TVVFFCYPLLPDSCPRAHRLPALKVAMYVLMVLMILTTVFGNLLIIIS
ISHFKQLQSPTHLIVRSLAASDCLLGSLVMPYSMVRSVEGCWYLGDFVCKVHSSLDMTFSISSLI
HLSLISVDRYWAICDPLRYKMRVTNNIVTVFTTFTWLFSFLYSFSIVFSGINKIGLESFIMQVYC
VGSCVLFFNKQWGLICSLLTFFLPGTIMSSLYMNIFYVARKHAKVMSERVTGGLKSQGSAQREGK
AAKTLAIVMGVFYLCWLPFFTATAVDPFLNFVTPGDVFDALVWFGYFNSTCNPLIYGFFYPRFQK
AFKILISTYTCGCNDSSTLILE*

>Sa-taar12c

MTLNKTDIYFENMFLCYPLLPNSCPKLHRLTVVKVAIYILMVLMILTTVFGNLLIIISISHFKQL
QSPTHLIVRSLASSDCLLGSLVMPYSMVRSVEGCWYLGDFVCKVHSSLDMRFCISSILHLSLISV
DRYMAICDPLRYRMRVTDKTVAVFITFIWLFSILYSFFIVFSGVNKMGLESFILQVYCVGSCVLF
FNKQWGIICPILTFFLPGTIMSSLYMKIFYVAQKHAKMMSERVTVMAAGGLKSQSSVHRERKAAK
TLAIVMGVFLFYWLPIFIATIIDSFLNL!
TPASVFDALVWFGYFNSTCNPLIYGFFYSRFQKAFKILISSSIYGFIDSSTFT

>Sa-taar12d

MTSNETNTQIVFLCYPLRPDSCLKVQRYVVVKVAMYVFLVLMILTTVFGNLLIIISISHFKQLQS
PTHLIVRSLASSDCLLGSLVMPYSMVRSVEGCWYLGDFVCKVHSSLDMSFCISSILHLSLISVDR
YMAICDPLRYIMRVTNNTVTAFIIFIWLFSFVYSFSVVFSGINAVGLEMLILQTHCVGSCVLFFN
KQWGIICPILTFFLPGSIMSSLYMKIFYVAQKHAKVMSERVTGRLKSQSSAHRERKAAKTLAFVM
GVFLFCWLPFFTVTALDPFFNFFTPAIVFDAVIWFAYLNSTCNPLIYGFFYPCFQSAFKILISTY
ICGNKDCNTLVFE*

Sinocyclocheilus grahami

>Sg-taar12a

MTSNETDIYPENVFFCYPLLPDSCPRAHRLPALKVAMYVLMVLMILTTVFGNLLIIISISHFKQL
QSPTHLIVRSLAASDCLLGSLVMPYSMVRSVEGCWYLGDFVCKVHSSLDMTFSISSLIHLSLISV
DRYWAICDPLRYKMRVTNNIVTVFTTFTWLFSFLYSFSIVFSGINKIGLESFIMQVYCVGSCVLF
FNKQWGLICSLLTFFLPGTIMSSLYMIIFYVARKHAKVMSERVTGGLKSQGSAQREGKAAKTLAI
VMGVFYLCWLPFFTATAVDPFLNFVTPVDVFDALVWFGYFNSTCNPLIYGLFYPRFQKAFKILIS
TYICGCNDSSTLILELI*

>Sg-taar12b

MTSNETDIYPENVFFCYPLLPDSCPRAHRLPALKVAMYVLMVLMILTTVFGNLLIIISISHFKQL
QSPTHLIVYSLAASDCLLGALVMPYSMVRSVEGCWYLGDFVCKVHSSLDMTFSISSKIHLGLISV
DRYWAICDPLRYKMRVTNNIVTVFSTFAWLFSFLYSFSIVFSGINIIGLESFIMQVYCVGSCVLF
FNKQWGLICSLLTFFLPGTIMSSLYMKIFYVARKHAKAMSERVTGGLKSQGSAQREGKAAKTLAI
VMGVFYLCWLPFFTATAVDPFLNFVTPVDVFDALVWFGYFNSTCNPLIYGLFYPRFQKAFKILIS
TYICGCNDSSTLILELI*

>Sg-taar12c

MTLNKTDIYFENMFLCYPLLPDSCPKLHRLTVVKVAIYILMVLMILTTVFGNLLIIISISHFKQL
QSPTHLIVRSLASSDCLLGSLVMPYSMVRSVEGCWYLGDFVCKVHSSLDMSFCISSILHLSLISV
DRYMAICDPLRYRMRVTNNTVTVFITFIWLFSILYSFFIVFSGVNKIGLESFILQVYCVGSCVLF
FNKQWGIICPILTFFLPGTIMSSLYMKIFYVAQKHAKIMSERVTVMAARGLKSQSSVHRERKAAK
TLAIVMGVFLFCWLPIFISTIIDSFLNFVTPASVFDALVWFGYFNSTCNPLIYGFFYSRFQKAFK
ILISSCIYGFIDSSTFTFE*

>Sg-taar12d

MTSNETYTQIVFHCYPLRPDSCLKVQRYVVVKVAMYVFLVLMILTTVFGNLLIIISISHFKQLQS
PTHLIVRSLASSDCLLGSLVMPYSMVRSVEGCWYLGDFVCKVHSSLDTSFCISSLLHLSLISVDR
YMAICDPLRYRMRVTNNTVTAFIIFIWLFSFVFSFSVVFSGINAVGLEMLILQTHCVGSCVLFFN
KQWGIICPILTFFLPGTIMCSLYMKIFYVAQKHAKVMSERMTGGMKSQSSAHRERKAAKTLAIVM
GVFLFCWLPFFTVTALDPFFNFFTPAVVFDAVIWFAYLNSTCNPLIYGFFYPCFQSAFKILISTY
ICGNKDCNTLVFE*

>Sg-taar12e

MTSNETQTENMFLCYPLRLDSCLKPHRLTVVKVAMYTLMVLMILTTVFGNLLIIISISHFKQLQS
PTHLIVRSLAASDCLLGSLVMPYSMVRSVEGCWYLGDFVCKVHSSLDMSFCISSILHLSLISVDR
YMAICDPLRYGMRVTNNTVTVFIIFIWLFSFVYSFSVVFSGITAVGLEMLILQTYCVGSCAVFFN
KEWGIICSVLTLFLPGTIMSSLYLKIFYVARKHAKVMSERMTGGMKSQSSVHRERKAAKTLAIVM
GVFLLCWLPYFTAALLDPFFNFWTPAVVFDAVIWFAYFNSTCNPLIYGFFYPYFQKAFKILISTY
ICGIKHSNTLIFE*

>Sg-taar12f

MIANETDIESVFLCYPLRPDSCLRAHRLTVLKVAMYVFMVLMILTTVFGNLLVIISISHFKQLQS
PTHMIVQSLAACDCLLGSLVMPYSMVRSVEGCWYLGDVVCKVHSSLDMTFSISSILHLSLIAIDR
YWAICDPLRYKMRVTNNTVTVFITFTWLFSFVYSFSVVFTGVNNVGLEELILQISCFGGCVLFFN
KEWGLICALFVFLIPGTIMSSLYMSIFNVVKKHAKVLSEKVSVAPTVGGNCQTSSQRERKAAKTL
AIVMGVFYLCWLPFFTATAIDPFLNFVTPVDVFDALVWFGYFNSTCNPLIYGFFYPRFQKAFKIL
ISTYICGISESHTLTFE*

Sinocyclocheilus rhinocerous

>Sr-taar12a

MTSNETDIYPENVFFCYPLLPDSCPRAHRLPALKVAMYVLMVLMILTTVFGNLLIIISISHFKQL
QSPTHLIVRSLAASDCLLGSLVMPYSMVRSVEGCWYLGDFVCKVHSSLDMTFSISSLIHLSLISI
DRYWAICDPLRYKMRVTNNIVTVFTTFTWLFSFLYSFSIVFSGINKIGLESFIMQVYCVGSCVLF
FNKQWGLICSLLTFFLPGTIMSSLYMNIFYVARKHAKVMSERVTGGLKSQGSAQREGKAAKTLAI
VMGVFYLCWLPFFTATAVDPFLNFVTPGDVFDALVWFGYFNSTCNPLIYGFFYPRFQKAFKILIS
TYICGCNDSSTLILE*

>Sr-taar12b

MTSNETDIYPENVFFCYPLLPDSCPRAHRLPALKVAMYVLMVLMILTTVFGNLLIIISISHFKQL
QSPTHLIVCSLTASDCLLGSLVMPYSMVRSVEGCWYLGDFVCKVHSSLDMTFSISSLIHLSLISI
DRYWAICDPLRYKMRVTNNIVTVFTTFTWLFSFLYSFSIVFSGINKIGLESFIMQVYCVGSCVLF
FNKQWGLICSLLTFFLPGTIMSSLYMNIFYVARKHAKVMSERVTGGLKSQGSAQREGKAAKTLAI
VMGVFYLCWLPFFTATAVDPFLNFVTPGDVFDALVWFGYFNSTCNPLIYGFFYPRFQKAFKILIS
TYICGCNDSSTLILE*

>Sr-taar12c

MTLNKTDIYFENMFLCYPLLPNSCPKLHRLTVVKVAMYVLMVLMILTTVFGNLLIIISISHFKQL
QSPTHLIVRSLASSDCLLGSLVMPYSMVRSVEGCWYLGDFVCKVHSSLDMSFCISSILHLSLISV
DRYMAICDPLRYRMRVTNKTVAVCITFIWLFSILYSFFIVFSGVNKISLESFILQVYCVGSCVLF
FNKQWGIICPILTFFFPGTIMSSLYMKIFYVAQKHAKIMSERVTVMAAGGLKSQSSVHRERKAAK
TLAIVMGVFLFCWLPIFIATIIDSFLNFVTPASVFDALVWFGYFNSTCNPLIYGFFYSRFQKAFK
ILISSCIYGFIDSSTFIFE*

>Sr-taar12d

MTSNETNTQIVFLCYPLRPDSCLKVQRYVVVKVAMYVFLVLMILTTVFGNLLIIISISHFKQLQS
PTHLIVRSLAASDCLLGSLVMPYSMVRSVEGCWYLGDFVCKVHSSLDTSFCMSSLLHLSLISVDR

YMASCDPLRYRMRVTNNTVTAFIIFIWLFSFVYSFSVVFSGINAVGLEMLILQTHCVGSCVLFFN
KQWGIICPILTFFLPGTIMSSLYMKIFYVAQKHAKVMSERMTGRLKSQSSAHRERKAAKTLAFVM
GVFLFCWLPFFTVTALDPFFNFFTPAVVCCQVHSPDT*

>Sr-taar12e

MTSNETQTENMFLCYPLRPDSCLKVHRLTVIKVAMYVLVVLMILTTVFGNLLIIISISHFKQLQS
PTHLIVRSLASSDCLLGSLVMPYSMVRSVEGCWYLGDFVCKVHSSLDMSFCISSLLHLSLISVDS
YWAICDPLSQVRMRVTNNTVTVFITFIWLFSFVYSFSVVFSGITAVGLEMLILQTYCVGSCVVFF
NKQWGIVCPILTFFLPGTIMSSLYLKIFYVARKHAKVMSERMTGGMKNQSSAHRERKAAKTLAIV
MGVFLLCWLPYFTATVLDPFFSFWTPAVVFDALFWFAYFNSTCNPLIYGFFYPYFQKAFKILIST
YICGIKHSNTLIFE*

>Sr-taar12f

YSMVRSVEGCWYLGDVVCKVHSSLDMTFSISSILHLSLIAIDRYWAICDPLRYKMRVTNNTVTVF
ITFTWLFSFVYSFSVVFTGVNNVGLEELILQISCFGGCVLFFNKEWGLICALFVFLIPGTIMSSL
YMSIFNVVKKHAKVLSEKVSVAPTVGGNCQTSSQRERKAAKTLAIVMGVFYLCWLPFFTATAIDP
FLNFVTPVDVFDALVWFGYFNSTCNPLIYGFFYPRFQKAFKILISTYICGI

>Sr-taar12g

MYCIYINLSLHICLYHVIIYEIILGTIWSIFEWLFTFTKQTLCFSLAYQSLFEAHVMIANETDIE
NVFLCYPLRPDSCPRAHRLTILKVAMYVFMVLMILTTVFGNLLVIISISHFKQLQSPTHMIVQSL
AACDCLLGSLVMPYSMVRSVEGCWYLGDFVCKVHSSLDMTFSISSKIHLGLISVDRYWAICDPLR
YKMRVTNNIVTVFTTFTWLFSFLYSFSIVFSGINKIGLESFIMQVYCVGSCVLFFNKQWGLICSL
LTFFLPGTIMSSLYMNIFYVARKHAKVMSERVTGGLKSQGSAQREGKAAKTLAIVMGVFYLCWLP
FFTATAVDPFLNFVTPGDVFDALVWFGYFNSTCNPLIYGFFYPRFQKALTVNHSATLFHKPG*

TAAR 13

Anguilla anguilla

>Aa-taar13a

MDILKHQELNASQLCYPSVNGSCIKINQTWAAQFISYIFLVAGMIFTILGNLVVIISIAHFKQLH
TPTNILVMSLAAADLLLGIVVIPFSMIRSVDGCWYFGDAFCLLHSSFDLFLTCASLFHLIFIAID
RYQAVCNPLHYSTRITIPIAWLMSALSWVIAGVYSYGLLYSKANVKGLEEHIASIYCFGSCILLF
NSLWGVLDTLIAFFLPCCIMMGLYAKIFFVXPSSSNMNLFIER

>Aa-taar13b

MDILKHQDLNAPQFCFPSVNGSCIKISQTWAAQFILYIFFVAGMIFTILGNLVVIISIAHFKQLH
TPTNILVMSLAVADLLLGMVVMPFSMIRSVEGCWYFGDAFCLLHSSFDMFLTCASLFHLIFIAID
RYQAVCNPLHYSTRITIPIAWLMSALSWVIAGVYSYGLLYSKANVKGLEAHIASIYCLGSCILLF
NSLWGVLDTLIAFFLPCSIMMGFYVKIFFVAREHVRKIGDMNHQKQLNEENKNKLSRSSERKAAK
TLGIVMGVFILCWMPFFVNMIIDPYTNFSTPVIIFDVLVWLGYFNSTLNPIIYSLFYPWFQKTFK
IIVSLKILTPNSSNINLFTER

>Aa-taar13c

HSSFDMFLTSVSIFHLISIAIDRYQAVCNPLHYSTRITIPIAWLMSALSWVIAAAYSYGLLYSKA
NVKGLEDYIASIYCLGDCVLLFNALWGALDTLIAFFLPCFVMMGLYTKIFLVAREHARKIEDMKH
KITVNEENRNKPSQRSEQKAAKTLGIVVGVFIICWLPFFVNSLIDPYTNFATPAIVFDVFVWLGY
FNSTLNPIIYALFYPWFQKTLKLIVTFKIFTPHSSNINVFPER

>Aa-taar13d

MDDLVHQEPDAQEYYYPSTNGSCIKGNQSIAAQVMLYIFFVTGMIFTILGNLVVIISIAHFKQLH
SPTNILVMSLAVVDLLLGMVVMPFSMIRSVEGFWYFGDAFCLLHSSFDMFLTCASIFHLIVIAID
RYQAVCNPLHYSTRITIPIAFLMSALSWIIAAAYSYALLYSKANVKGVEDDVASINCLGGCVLLF
NAVWGPLDTMIEFFVPCFVMMGLYTKIILVAREHARK

>Aa-taar13e

DAQQYCYPAINGSCIKGNHTIAAQVILYIFFVAGIIFTVLGNLVVIISITHFKRLHTPTNILVMS
LAVADMLLGMLVMPFSMVRSVAGCWYFGEVFCQLHSSFDMFLATVSIFHLICIAIDRHEAVCNPL
HYSRRITIPIAFLMSTLSWSIAAAYSYGLLYSKADEKGVEDYIASIHCLGSCFPFVLSFNALWGV
LDSLIAFFVPCVIMMGLYAKIYLVAREHARKIEGMNNKRPVNEENRNKLSQGSEQKAAKTLGIVV
SAFIVCWLPFFINSLIDPYTTFSTNSPVLSDAFVWLGYFNSGLNPIIYGLFYPWFQKTLKLIITF
KIFTPHSSNINVFSER

Anoplopoma fimbria

>Af-taar13a

MMSRGLSQEQYCFPGSNASCVTAQFSMGTEVALYLLFSLGILITILGNSVVIVSIGHFKQLHNPT
NVLILSLALADLLVGVIVMPFSAIRTIHGCWFYGDDFCLLHSSFDMFLTSVSIFQLICIAVDRQQ
AICNPLHYSRNITMSVAWIMVCASWSLAAVYSYGLLYSKANVAGIEDYMSSINCLGSCNLVFNHI
WGILDGVTAFFFPCTVMVCLYTKIFIVAKDHVRKIGDMSKGSNNRGRGGLIKQSEHKAAKTLGIV
LGAFIFCWMPFFINSIIDAYTGFSTPASVFEAFVWLGYFNSTLNPIIYALFY

>Af-taar13b

IVSIGHFKQLHNPTNVLILSLALVDLLVGVIVMPFSAIRTIHGCWFYGDDFCLLHSCLDVFLTTL
SIFHLISIAIDRQQAICNPLHYSRNITXSVAWIMVCASWSLAAVYSXGLLYSKANVAGIEDYMSS
INCLGSCNLVFNHIWGILDGVTAFFFPCTVMVCLYTKIFIVAKDHVRKIGDMSKGSNNRGRGGLI
KQSEHKAAKTLGIVLGAFIFCWMPFFINSIIDAYTGFSTPASVFEAFVWLGYFNSTLNPIIYALF
Y

Anguilla japonica

>Aj-taar13a

MDILKHQELNAPQFCYPSVNGSCIKINQTWAAQFISYIFLVAGMIFTILGNLVVIISIAHFKQLH
TPTNILVMSLAAADLLLGIVVIPFSMIRSVDGCWYFGDAFCLLHSSFDLFLTCASLFHLIFIAID
RYQAVCNPLHYSTRITIPIAWLMSALSWVIAGVYSYGLLYSKANVKGMEEHIASIYCFGSCILLF
NSLWGVLDTLIAFFLPCCIMMGLYAKIFFVAREHVRKIGDMNHRKHLNEENKNKLSRSSERKAAK
TLGIVMGVFILCWMPFFVNMIIDLYTNLSTPFIIVEVLFWLGYFNSTLNPIIYSLFYPWFQKTFK
LIVSLKILNPNSSNMNLFTER*

>Aj-taar13b1

MDILKHQELNAPQFCFPSVNGSCIKISQTWAAQFILYIFFVAGMIFTILGNLVVIISIAHFKQLH
TPTNILVMSLAVADLLLGMVVMPFSIIRSVEGCWYFGDAFCLLHSSFDMFLTCASLFHLIFIAID
RYQAVCNPLHYSTRITIPIAWLMSALSWVIAGVYSYGLLYSKANVKGLEAHIASMYCLGSCILLF
NSLWGVLDTLIAFFLPCSIMMGF!
YVKIFFVAREHVRKIGDMNHQKHLNEENKNKLSRSSERKAAKTLGIVMGVFILCWMPFFVNMIID
PYTNFSTPLIVFDVLVWLGYFNSTLNPIIYSLFYPWFQKALKLIVSLKILTPSSSNMNLFIER*

>Aj-taar13b2

MDILKHQELNAPQFCYPSVNGSCIKISQTWPAQFILYIFFVAGMIFTILGNLVVIISIAHFKQLH
TPTNILLMSLAAADLLLGMVVMPFSMIRSVEGCWYFGDDFCLLHSSFDMFLTCASLFHLIFIAID
RYQAVCNPLHYSTRITIPIAWLMSALSWVIANVYSYGLLYSKANVKGLEDHIASMYCLGNCILLV
NSLWGVLDSLIAFFLPCSIMMGFYAKIFFVAREHVRKIGDMNHQKHLNEENKNKLSRSSERKAAK
TLGIVMGVFILCWMPFFVNLIIDPYINFSTPVIVFDVLVWLGYFNSTLNPIIYSLFYPWFQKTFK
IIVSLKILNPNSSNMNLFPER*

>Aj-taar13c

MEALVHQEPDAQQYCYPSTNGSCIKGNQPIAAQVILYIFFVTGMIITILGNLVVIISIAHFKQLH
TPTNILVMSLAVADLLLGMSVMPFSMIRSVEGCWYFGDAFCLLHSSFDMFLTSVSIFHLISIAID
RYQAVCNPLHYSTRITIPIAWLMSALSWIIAAAYSYGLLYSKANVKGLKDYIASIYCLGDCVLLF
NALWGALDTLIAFFLPCFVMMG

>Aj-taar13d1

MHRNTIIPQLXXXXXXXXXXXXXXXXXXXXXXXXXXXXXXXXXXXXXXXXXXXXXXXXXXXXXXX
XXXXXXXXXXXGMVVMPFTMIRSVEGFWYFGDAFCLLHSSFDMFLTCASIFHLIFIAIDRYQAVC
NPLHYSTRITIPIAFLMSALSWIIAAAYSYGLLYSKANVKGVEDDVASINCLGGCVLLFNALWGP
LDTFIEFFVPCFVMMGLYTKIFLVAREHARKIEDMKHKITVNEENRNKPSQRSEQKAAKTLGIVV
GVFIICWLPFFVNSLIDPYTNFATPAIVFDVFVWLGYFNSTLNPIIYALFYPWFQKTLKLIVTFK
IFAPHSSNINVFPER*

>Aj-taar13d2

DLVHQEPDAQEYYYPSTNGSCIKGNQSIAAQVTLYIFFVAGMILTILGNLVVIISIVHFKQLHTP
TNVLVMSLAVVDLLLGMVVMPFSMIRSVEGFWYFGDAFCLLHSSFDMFLTCASIFHLIFIAIDRI
AIDRYQAVCNPLHYSTRITIPIAFLMSALSWIIAAAYSYALLYSKANVKGVEDDVASIYCLGGCV
LLFNAVWGPLDTMIEFFVPCFVMMGLYTKIILVAREHARKIEDMKHKITVNEENRNKLSQRSEQK
AAKTLGIVVSAFIICWFPFFSNSLIDPYTDFATPPIVFDAFVWLGYFNSTLNPIIYGLFYPWFQK
TLKLIITLKIFTPHSSNINVFS

>Aj-taar13e

MDALVHQEPDAQQYCYPAINGSCIKGNHTIAAQVILYIFFVAGIIFTVLGNLVVIISITHFKQLH
TPTNILVMSLAAADMLLGMLVMPFSMVRSVAGCWYFGEVFCQLHSSFDMFLATVSIFHLICIAID
RHEAVCNPLHYSARITIPIAFLMSALSWSIAAAYSYGLLYSKADEKGVEDYIASIHCLGSCFPFV
LSFNALWGVLDSLIAFFVPCVIMMGLYAKIYLVAREHARKIEGMNRKRPVNEENKHKLSQGSEQK

AAKTLGIVVSAFIVCWLPFFINSLIDPYTTFSTNSPVLSDAFVWLGYFNSGLNPIIYGLFYPWFQ
KTLKLIITFKIFTPHSSNINVFSER

>Aj-taar13f

MDALVLQKPDAQQYCYPSTNGSCFKGNQPIAAQVILYIFFVTGMIITILGNLVVIISIAHFKQLH
TPTNILVMSLAVADLLLGMLVMPFSMIRSVDGCWYFGDAFCLLHSSYDMFLATASIFHLIFIAID
RHEAVCNPLHYSTRITIPIAFLMSALSWIIAAAYSYGLLYSKADVKGLEDYIASLHCLGRCVHFV
LSLNALWGVLDTLIAFFVPCFVMMGLYAKIYLVAREHARKIGGMNHKRPVNEENKHKLSQGSEQK
AAKX

>Aj-taar13g

MEALVHQEPDAQQYCYPSTNGSCFKGNQPIAAQVILYIFFVTGMIITILGNLVVIISIAHFKQLH
TPTNILVMSLAVADLLLGMLVMPFSMVRSVDGCWYFGDAFCLLHSSYDMFLATASIFHLIFIAID
RHQAVCNPLYYSTRITIPIAFLMSALSWSIAAAYSYGLLYSKADVKGLEDYIASIHCFGGCVHFV
LSFNALWGVLDTLIAFFVPCFVMMSLYAKIFLVAREHARKIGGINHKIPVNEENKHKLSQGSEQK
AAKTLGIVMSVFLICWLPCFVNSLIDPYTTFSANHVVSDAFVWLGYFNSTLNPIIYGLFYPWFQK
TLKLXKVIVCKVVLQANFTISKNAVL*

Astyanax mexicanus

>Am-taar13a

MESYLTEEADLSQYCYPESNASCVKSFYSVGAKSVLYFLLILAMAVTVLGNFVVIVSIAHFKQLH
TPTNILVMSLALADLFVGMIVMPFSTIRSVDGCWYFGQGFCLLHSSFDMLLTSASIFHLVCIATD
RYQAVCHPLHYPTRITIPTAWLMVALSWTTAAVYSYGLMYSKANVANLEEYMESIFCVGSCDLLF
NALWGALDTLIGFLLPCSVMGCLYAKIFLVSKKHATKIEGKKQCKKGTSMNMVSQSVKHENKAAK
TLGIVVGAFILCWMPFFVNSIVDPYINFSTPAVLFEVLVWLGYLNSTLNPIIYGLFYPWFRKSLY
LIVTLRIFTPHSSDTNIFLA*

Anguilla rostrata

>Ar-taar13a

MDILKHQELNASQFCYPSVNGSCIKINQTWAAQFISYIFLVAGMIFTILGNLVVIISIAHFKQLH
TPTNILVMSLAAADLLLGIVVIPFSMIRSVDGCWYFGDAFCLLHSSFDLFLTCASLFHLIFIAID
RYQAVCNPLHYSTRITIPIAWLMSALSWVIAGVYSYGLLYSKANVKGLEEHIASIYCFGSCILLF
NSLWGVLDTLIAFFLPCCIMMGLYAKIFFVAREHVRKIGDMNHRKHLNEENKNKLSRSSERKAAK
TLGIVMGVFILCWMPFFVNMIIDLYTNLSTPFIIVEVLFWLGYFNSTLNPIIYSLFYPWFQKTFK
LIVSLKILNPSSSNMNLFIER*

>Ar-taar13b1

MDILKHQELNAPQFCYPSVNGSCIKISQTWAAQFILYIFFVAGMIFTILGNLVVIISIAHFKQLH
TPTNILLMSLAAADLLLGMVVMPFSMIRSVEGCWYFGDAFCLLHSSFDMFLTCASLFHLIFIAID
RYQAVCNPLHYSTRITIPIAWLMSALSWVIAGVYSYGLLYSKANVKGLEAHIASIYCLGNCILLV
NSLWGVLDSLIAFFLPCSIMMGFYAKIFFVAREHVRKIGDMNHQKHLNEENKNKLSRSSERKAAK
TLGIVMGVFILCWMPFFVNLIIDPYINFSTPVIIFDVLVWLGYFNSTLNPIIYSLFYPWFQKTFK
IIVSLKILTPSSSNINLFIER*

>Ar-taar13b2

MDILKHQDLNAPQFCFPSVNGSCIKISQTWAAQFILYIFFVAGMIFTILGNLVVIISIAHFKQLH
TPTNILVMSLAVADLLLGMVVMPFSMIRSVEGCWYFGDAFCLLHSSFDMFLTCASLFHLIFIAID
RYQAVCNPLHYSTRITIPIAWLMSALSWVIAGVYSYGLLYSKANVEGLEAHIASIYCLGSCILLF
NSLWGVLDTLIAFFLPCSIMMGFYVKIFFVAREHVRKIGDMNHQKQLNEENKNKLSRSSERKAAK
TLGIVMGVFILCWMPFFVNMIIDPYTNFSTPVIIFDVLVWLGYFNSTLNPIIYSLFYPWFQKALK
LIVSLKILTPSSSNMNLFIER*

>Ar-taar13c

MDALVHQEPDAQQYCYPSTNGSCFKGNQPIAAQVILYIFFVTGMIITIFGNLVVIISIAHFKQLH
TPTNILVMSLAVADLLLGMSVMPFSMIRSVEGCWYFGDAFCLLHSSFDMFLTSVSIFHLISIAID
RYQAVCNPLHYSTRITIPIAWLMSALSWIIAAAYSYGLLYSKANVKGLEDYIASIYCLGDCVLLF
NAVWGALDTLIAFFLPCFVMMGLYTKIFLVAREHARKIEDMKHKITVNEENRNKPSQRSEQKAAK
TLGIVVGVFIICWLPFFVNSLIDPYTNFATPAIVFDVFVWLGYFNSTLNPIIYALFYPWFQKTLK
LIVTFKIFTPHSSNINVFPER*

>Ar-taar13d1

MDDLVHQEPDAQEYYYPSTNGSCIKGNQSIAAQVMLYIFFVTGMIFTILGNLVVIISIAHFKQLH
TPTNILVMSLAVVDLLLGMVVMPFSMIRSVEGFWYFGDAFCLLHSSFDMFLTCASIFHLIVIAID
RYQAVCNPLHYSRRITIPIAFLMSALSWVIAAAYSYALLYSKANVKGVEDDVASIYCLGGCVLLF
NAVWGPLDTMIEFFVPCFVMMGLYTKIILVAREHARKIEDMKHKITVNEENRNKLSQRSERKAAK
TLGIVVSVFIICWLPFFSNSLIDPYTDFGTSPIVFDAFVWLGYFNSTLNPIIYGLFYPWFQKTLK
LIITLKIFTPHSSNINVFSER*

>Ar-taar13d2

MDDLVHQEPDAQEYYYPSTNGSCIKGNQTIAAQVTLYIFFVTGMIFTILGNLVVIISIAHFKQLH
TPTNILVMSLAVVDLLLGMVVMPFTMIRSVEGFWYFGDAFCLLHSSFDMFLTCASIFHLIFIAID
RYQAVCNPLHYSTRITIPIAFLMSALSWIIAAAYSYALLYSKANVKGVEDDVASINCLGGCVLLF
NAVWGPLDTMIEFFVPCFVMMGLYTKIFLVAREHARKIEDMKHKIPVNEENRNKLSQRSEQKAAK
TLGIVVSAFIICWLPFFSNSLIDPYTNFATPPIVFDAFGWLGYFNSTLNPIIYGLFYPWFQKTLK
LIITLKIFTPHSSNINVFS*

>Ar-taar13e

MDALVHQEPDAQQYCYPAINGSCIKGNHTIAAQVILYIFFVAGIIFTVLGNLVVIISITHFKRLH
TPTNILVMSLAVADMLLGMLVMPFSMVRSVAGCWYFGEVFCQLHSSFDMFLATVSIFHLICIAID
RHEAVCNPLHYSRRITIPIAFLMSTLSWSIAAAYSYGLLYSKADEKGVEDYIASIHCLGSCFPFV
LSFNALWGVLDSLIAFFVPCVIMMGLYAKIYLVAREHARKIEGMNNKRPVNEENRNKLSQGSEQK
AAKTLGIVVSAFIVCWLPFFINSLIDPYTTFSTNSPVLSDAFVWLGYFNSGLNPIIYGLFYPWFQ
KTLKLIITFKIFTPHSSNINVFSER*

>Ar-taar13f

MDALVHQEPDAQQYCYPSTNGSCFKGNQPIAAQVILYIFFVTGMIITILGNLVVIISIAHFKQLH
TPTNILVMSLAVADLLLGMLVMPFSMIRSVDGCWYFGDAFCLLHSSYDMFLATASIFHLIFIAID

RHQAVCNPLHYSTRITIPIAFLMSTLSWIIAAAYSYGLLYSKADVKGLEDYIASIHCLGGCVHFV
LSFNALWGVLDTLIAFFVPCFVMMSLYAKIFLVAREHARKIGGMNRKIPVNAENKHKLSQGSEQK
AAKTLGIVMSVFIICWLPCFVNSLIDPYTTFSANHVVSDAFVWLGYFNSTLNPIIYGLFYPWFQK
TLKLIITFKIFTPHSSNINVFSER*

Cyprinus carpio

>Cc-taar13a

MDLSSQEYDSTQFCYPAVNKSCLKGTHHVSTQTVVYLLLVLAMIVTILGNLVVIISIAHFKQLQT
PTNILVMSLALADLLLGLVVMPFSMIRSVDGCWYYGDAFCLLHSTFDLFLTSVSIFHLIFIAIDR
HQAAVCYPLQYPTRITISVAWVMVMISWSMAALYSYGLVYSKANVEGLEEYIESIYCMGSCTLLF
NALWGAIDTLVAFLLPCSVMVGLYARIFVIAKQHARKLCEANQQENENMFKSSRRSERKAAKTLG
VVVGAFIICWLPFFINSLIDPYINFSTPIALFEVFVWLGYINSTINPIIYGLFYPWFRKTLSLII
TMRIFEPNSSDINVFTV*

>Cc-taar13b

MDLSSQEYDPTQFCYSAVNNSCLKGTHHVSTQTAVYLLLVLAMIVTILGNLVVIISIAHFKQLQT
PTNILVMSLALADLLLGLVVMPFSMIRSVDGCWYYGDAFCLLHSSFDLFLTSVSIFHLIFIAIDR
HEAVCYPLQYPTRITISVAWIMVMISWSMAALYSYGLVYSKANLEGLEEYIESIYCMGSCTLLFN
ALWSVLDTLLTFFLPCSVMIGLYARIFVVARKHIRTIGEANHHENESTFKSSRRSERKAAKTLGV
VVGAFIICWLPFFINSLIDPYISFSTPFALFEAFVWLGYINSTINPIIYGLFYPWFRKTLSLIIR
LKIFEPNSSDISVFTV*

>Cc-taar13c

MDLSSQEYSPTQFCFPAVNNSCLKGTHHVSTQTVVYLLLVSAMIVTILGNSVVIISIAHFKQLQT
PTNILVMSLALADLLLGLVVMPFSMIRSVDGCWYYGDAFCLLHSSFDLFLTSVSIFHLIFIAIDR
HQAVCYPLQYPTRITIPVAWVMVMISWSMAALYSYGLVYSKANVEGLEEYSESIYCMGSCTLLFN
SLWSVLDTLLTFFLPCSVMIGLYARIFVVAKKHIRTIGEASQHENENMFKSSRRSERKAAKTLGV
VVGAFIICWLPFFINSLMDPYINFSTPFVLFDAFGWFGYINSTINPIIYGLFYPWFRKSLSLIIT
MRIFEPNSSDINVFTV*

>Cc-taar13d

MDLSSQQYDPTQFCFPAVNNSCLKGTHHVSTQTVVYLLLVSAMIVTILGNSVVIISIAHFKQLQT
PTNILVMSLALADLLLGLVVMPFSMIRSVDGCWYYGDAFCLLHSTFDLFLTSVSILHLVCIAIDR
HQAVCYPLQYPTRITIPVAWVMVMISWAMIAVYSYGLLYSKANVEGLEEYIESIYCIGHCSLLFS
NLWSVLDTLITFFFPCSVMFGLYVRIFVVAKKHVRTISEANQHENENVFKSSRRSERKAAKTLGV
VVLPFFINSVMDPYINFSTPLALFEVFVWLGYINSTINPIIYGFFYPWFRKTLSLIVTRRIFEPN
SSDINVFTV*

>Cc-taar13e

KKQHFIGSSFLKLRGFKGTHHVSTQTVVYLLLVSAMIVTILGNSVVIISIAHFKQLQTPTNILVM
SLALADLLLGLVVMPFSMIRSVDGCWYYGDAFLFAAIPPLTLFLASVFYFPFLFVLLLSIAIDRH
QAVCYPLQYPTRITIPVAWVMVMISWAMIAVYSYGLLYSKANVEGLEEYIESIYCIGHCSLLFSN
LWSVLDTLITFFFPCSVMFGLYVRIFVVAKKHVRTISEANQHENENVFKSSRRSERKAAKTLGVV

VGAFIICWLPFFINSVMDPYINFSTPLALFEVFVWLGYINSTINPIIYGFFYPWFRKTLSLIVTR
RIFEPNSSDINVFTV*

Clupea harengus

>Ch-taar13a

MDTGISSDQYCFPSVNGSCMKGTYNQGTQVVLYIMFWTSMVITILGNLVVIISIMHFKQLHTPTN
LLVMSLALADLLLGLTVMPFSIVRSVDGCWYYGNAFCLLHSSFDMFLTSASIFHLISIAIDRHQA
VCYPLLYPTSITIPVAWLMIAVSWAIAAAYSFGLLYSRANVAGLDDFLKSIYCIGSCNLLFNALW
GALDTLIAFFLPCTIMVGLYAKIFSVAKGHIKKIEDVNRELNLKNEHVQSSERKAAKTLGIVVGA
FIFCWMPFFVNSIVDPYTNFSTPAILFEIFTWLGYFNSTLNPIIYGLFYPWFRKSLYLIVTLKIF
SPHSSDINIFPS*

Dicentrarchus labrax

>Dl-taar13a

MMTGSLSQEQYCFPGSNASCVMAQLRMGTKFALYLLFALGMLVTILGNSVVIVSISHFKQLHNPT
NMLILSLALVDLLVGVIVMPFSAFRTIHGCWFYGDAFCLLHSSFDVFFTTLSIFHLICIAVDRQQ
AICNPLHYSRKITMPVAWFMICVSWTLAAVYSYGLLYSKANIEGLEDYILSINCLGSCFLLFNPL
WGVLDSVICFFFPCTVMVCLYTKIFIVAKEHVRQIGDMSNFSIDRGKGGLIKQSEHKAAKTLGIV
LGAFIFCWMPFFITSVTDAFTGFSTPVAIFEAFVWLGYLNSTLNPIIYALFYPCFKKCFYCIATL
KIFSSNSSTMNVSVN*

>Dl-taar13b

MMTGSLSQELYCFPGSNASCVIAQFGVGTKATLTLLFAFGMLVTILGNSVVIVSISHFKQLHNPT
NMFILSLALADLLVGVIVMPFSAFRTIHGCWFYGDAFCLLHSSFDMFLTSVSIFHLICIAVDRQQ
AICNPLHYSRKITMPVAWFMICVSWTLAAVYSYGLLYSKANIEGLEDYILSINCLGSCNLLFNPL
WGVLDSITAFFFPCTVMVCLYTKIFIVAKEHVRKIGDMNNCSNDRGKGGLIKQSEHKAAKTLGIV
LGAFIFCWMPFFINSIIDAFTGFSTPVAIFEASVWLGYFNSTLNPIIYALFYPWFKKCFYLIVNL
KIFNPHSSTIKVHVLTHL*

Esox Lucius

>El-taar13a

MSHTCSCDYVAKISSNFECVLFMAMDVPPQPLDPKLFCYPETNASCTREILNEGAQIALYLLFVL
GILVTILGNGVVIISIAHIKQLHTPTNMLIMSLALADLLIGVTVMPFSSIRAVEGCWYFGEVFCL
LHSSFDMFLTSVSIFHLVFIAIDRYLAVCNPLRYTTNITLPTAWLMVLGSWCLSALYSYGLLYSK
AHVRGQDESIASVYCLGSCDLFFNALWGALDTLIAFFFPCSVMVGLYTKIFFVAKEHVRKIEDRQ
KKLVGGGVVSKRSERKAAKTLGIVVGVFIFCWLPFFVNSIVDPYTNFSHNDFTPDTVFEMFLWLG
YFNSTVNPIIYALFYPWFRKCLNVIVTLKIFNKNSSYINVFAAT*

Gasterosteus aculeatus

>Ga-taar13a

MSLGLSQEQYCFSGSNSSCFRAQFSVETKVTLQLIFSLGMLITILGNSVVIVSIGHFKQLHNPTN
VLIVSLAYADLLVGVTVMPFSAIRTIHGCWFYGEDLCLLHSSFDMFLTTVSIFHLICIAIDRQQA

ICNPLHYSRNITISVAWIMVCASWALAAIYSHGLIYSKANVAGIE!
LLDNIFCFFFPCILMVCLYTKVFIVAKEHVRKIEDMSKSSMNKESGGLIKQSEHKAAKTLGIVLG
GFIFCWMPFFLNSIIDAYTGFSTSAFVFEALVWLGYFNSTLNPIIYALFYPCFKKCFCLIVNLKI
FNSQSSTIKVHILTHV*

Gadus morhua

>Gm-taar13a

MLIMTDIINQVEQQLCYPGSNTSCPRAEFHLGAQVALYTLFVLGMLVTVVGNAVVIVSIAHFKQL
HSPTNVLVLSLALADLLLGVTVMPFSTVRAVQGCWFYGDTFCLLHSAFDMFLTSVSIFHLICIAV
DRHEAVCKPLHYSRNITMTVAWLMVSASWALAALYSYCLMYSKANIRGLEDYLASIYCLGSCNLL
FNKLWGTLDTIIAFFFPCTIMVGLYAKIFLVAREHARKIDDMGGHGGTLNGKGAGGQTKRSEHKA
AKTLGIVVGAFIFCWMPFFLNSIADAYTGFSTPEAVFEVFVWLGYFNSTLNPVIYALNYPWFRKT
SRLIFTLKIFTSGSSSMNVTVN*

>Gm-taar13b

MRVCVYLCMLIMIDRIYQIEQQLCYPGSNTSCPRAEFHLGAQVALYTLFVLGMLATVVGNAVVIV
SIAHFQQLHSPTNVLVLSLALADLLLGVTVMPLSTVRAVQGCWFYGDTFCLLHSAFDMFLTSVSI
FHLICIAVDRHEAVCKPLHYSRNITMTVAWLMVSASWALAALYSYCLIYSKANIRGLEDYLASMY
CLGSCNMLFNKLWGTLDTIIAFFFPCTVMVGLYAKIFLVAREHARKIDDMGGHGGTLNGKGAGGQ
TKRSEHKAAKTLGIVVVAFIFCWMPFFLNSIVDAYTGFSTPAAVFEVFSWLSYFNSILNPVIYSL
NYPWFRKTSRLIFTLKIFTSDSSSINVTVN*

>Gm-taar13c

MLCEAYTRCCAQIPCRAGISDCVCLCMLIMTDIINQVEQQLCYPGSNTSCPRADFHLGAQVALYT
LFVLGMLVTVVGNAIVIVSIAHFKQLHSPTNVLVLSLALADLLLGLTVMPFSTLRAVQGCWFYGD
TFCLLHSAFDMLLTCNSIFHLICIAVDRHEAVCKPLHYSRNITMTVAWLMVSASWALATLYSYCL
IYSKANIRGLEDYLASIYCLGSCNLLFNKLWGTLDTIIAFFFPCTIMVGLYAKIFLVAREHARKI
DDMGGHGGTLNGKGASGQTKRSEHKAAKTLGIVVGAFIFCWMPFFLNSIADAYTGFSTPAAVFEV
FVWLGYFNSTLNPAIYALNYPWFRKTSRLIFT*

Ictalurus punctatus

>Ip-taar13a

MPEVLNPSLLEGAHLEEYCFPDSNVSCLKASYHMAAQTAIYFLLLLATTITILGNSVVIISIAHF
KQLHTPTNILVMSLGLADLIVGVIVMPFSMIRSVDGCWYFGEEFCLWHSSFDVFLTTASVFHLVC
IAVDRYEAVSYPLQYPTRVTMPVAWFMVTVSWTAAALYSFGLLLSKANVENLDEYIASTYCLGYC
NILFNELWSVLDTCVCFLLPCSVMICLYAKIFLISKKHARKVDCARQGKNDVTKFSQKVKHENKA
AKTLGIVVGAFNLCWMPFYINSILDPYINFSTPLVLFDIFVWLGYINSTLNPIIYGLFYPWFRKT
LYLMVTLKIFAPDSSDIKVYAA*

>Ip-taar13b

MPEALNSSLPEEVDLKTYCFPDSNVSCVKASYNIASKTVLYFILVFAMTITILGNSVVIISIAHF
KQLHTPTNILVMSLALVDLLLGLTVMPFSMIRSVDGCWYYGEEFCFIHSSFDMCLTTASIFHLIS
IATDRYEAVCHPLQYPSRVTIPVAWLMVALSWTAAAVYSYGLLCSKANVEKLDEYIASISCLGYC

GLLFNALWAALDACICFFLPCSVMFCLYAQIFFISKKHARKIEGVKPGRNDMNLTKFSQRVKHEN
KAAKTLGIVVGAFICCWMPFFVTSLLDPYINFATPLIIFDVFVWLGYINSALNPIIYGLFYPWFR
KTLHLIVTLKIFAPHSSDIKVYAA*

>Ip-taar13c

MPEALNSSLLEEVDLKTYCFPDSNVSCVKASYNVASKTVLYFILVFAMTITILGNSVVIISIAHF
KQLHTPTNILVMSLALVDLLLGLTVMPFSMIRSVDGCWYYGEEFCFLHSSFDMCLTGASIFHLIS
IAIDRYQAVSYPLQYPSRVTIPVAWFMAVTSWTASVVYSYTLLCSKANVEKLDEYIASISCLGYC
GLLFNALWAALDACICFFLPCSVMFCLYAQIFFISKKHARKIEGVKPGRNDMNLTKFSQRVKHEN
KAAKTLGIVVGAFICCWMPFFVTSLLDPFINFATPLVLYDVFIWLGYINSTLNPIIYGLFYPWFR
KTLHLIVTLKIFCSHSSDMKLYAS*

>Ip-taar13d

MPDAPNSSLLEEAHLEEYCYPDSNVSCLKMSYHMTTKTVLYFILVFAMTITIIGNFFVIISIAHF
KQLQTPTNILVMSLALVDLLLGITVMPFSMIRSVDGCWYYSKEFCFLHSSFDMCLTGASIFHLIS
IAIDRYQAVCYPLQYPTRVTKPVAWFMVAVSWTVAVVYSYSLLLTKASVDNGFADCLGSCSLFLN
ELWAILSIVTGFILPCCVMIGLYAKIFFISKKHARKIEGVKQGRTDMALTKFSQRVKHENKAAKT
LGIVVGAFNFCWMPFFLTSLLDPFINFATPLLLYDVFVWLGYINSTLNPIIYGFFYPWFRKTLYV
IVTLKIFAPHSSDMKVYTS*

>Ip-taar13e

MPGSLSYLQEEADFKVYCFPDYNASCLKNPHNVATQTVLYFILVFAMTITIIGNSFVIISIAHFK
QLQTPTNILVMSLALVDLLLGITVMPFSMIRSVDGCWYYSKEFCFLHSSFDMCLTGASIFHLISI
AIDRYQAVCYPLQYPTRVTKPVAWFMVAVSWTVAVVYSYSLLLTKASVDNEFADCLGSCSLFMNA
LWAILSIVTGFFLPCSAMIGLYAKIFFISNKHARKIEGVKQGRTDMALTKFSQRVKHENKAVKTL
GIVVGAFNFCWLPYFLTSLLDPFINFATPILLYDVFVWLGYINSTLNPIIYGFFYPWFRKTLYLI
VTLKIFAPHSSDMKVYTS*

>Ip-taar13f

MLDAPNSSLLEAADLREYCYPESNVSCVKISQNVVARTVLYSLLVFATVITILGNSMVIISIAHF
KQLHTPTNILVMSLALVDLLLGLTVMPFSMVRSVDGCWYFSEEFCFWHSSFDFLFTGASIFHLIF
IAVDRYQAVCHPLQYPTRVTKRVAWLMAALSWTIAAGYSYSLLLTKANVENLDDYAASINCLGSC
IFLINALCAVLSTFIGLILPCSFMICLYAQIYLISTRHAIKIGATKQSRHNRTIKVSQRVKHENK
AAKTLGIVVSAFHFCWMPYFITSVVDPFLNFTTPPSLYEVFVWLGYINSTLNPIIYGLFYPWFRK
ALYLILTLKIFARNSSDIKVYAT*

>Ip-taar13g

MVEAQNSAVLEVEDFIEYCYPESNVSCVKMSHNVVAKTVLYSILVFAMAITIVGNFVVIISIAHF
KQLHTPTNILVMSLALVDLLLGITVMPFSMVRTVDGCWYFGKEFCYWHSTFDFLFTGASIFHLIS
IATDRYQAVCYPLQYPTRITLPVAGFMAALSWILATVYAFSTVGSKANEANLQDYIASIECFGNC
FFLVNAICASISTSLFFILPVCIMIGLYAQIFLVSEKHARKMEGTKQNRTDMTSNKIFQKVKHEK
KAAKTLGIVVGAFNLCWMPYFITSVVDPLYNFTTPAIVYELFVWLGYINSTFNPIIYGLFYPWFR
KTLYLIVTLKIFAPNSSDIKVYAA*

>Ip-taar13h

MEAQNSAVLEVEDFIEYCYPESNVSCVKMSHNVVAKTVLYSILVFVLAITIVGNFVVIISIAHFK
QLHTPTNILVMSLALVDLLLGVTIMPFSMVRTVDGCWYFGKEFCYWHSTFDFLFTGASIFHLISI
ATDRYQAVCYPLQYPTRITLPVAGFMAALSWILATVYAFSTVGSKANEANLQDHIASIDCFGKCF
LLVNAVCASICASLFFILPVCIMIGLYAQIFLVSEKHARKMEGTKQNRTDMTSNKIFQKVKHEKK
AAKTLGIVVGAFNLCWMPYFITSVVDPLYNFTTPAIVYELFVWLGYINSTFNPIIYGLFYPWFRK
TLYLIVTLKIFAPNSSDVKVYAA*

Lates calcarifer

>Lac-taar13a

MTGGLSPEQYCFPDSNASCVMAQFSVGTKVALYLLFALGMLVTILGNSVVIVSIAHFTQLQNPTN
VLVLSLALADLLVGVVVMPFSAIRTVHGCWFYGDAFCLLHSSFDMFLTSVSIFHLICIAVDRHQA
ICNPLHYSRKITISVACFMVCVSWALAALYSYGLLYSKANIAGLEDYIASIYCLGSCNLLFNQLW
GVLDSITAFFFPCTVMVCLYTQIFIVAKEHVRKIGDMNNSSNDRGRGGLIKQSEHKAAKTLGIVL
GAFIFCWMPFFITSITDAYTGFITPAAIFEAFVWLGYFNSTLNPVIYAFFYPWFKKCFYLIVSLK
IFNPNSSTIKVNIRTHS*

>Lac-taar13b

MTGGLSPEQYCFPDSNASCVMAQFSVGTKVALYLLFALGMLVTILGNFVVIVSIAHFTQLQNPTN
VLVLSLALVDLLVGVVVMPFSAIRTVHGCWFYGDAFCLLHSSFDVFFTTLSIFHLICIAVDRHQA
ICNPLHYSRKISISVACFMVCVSWALAALYSYGLLYSKANIAGLEDYIASIYCLGSCNLLFNQLW
GVLDSIICFFFPCTVMVCLYTQIFIVAKEHVRKIGDMNNSSNDRGRGGLIKQSEHKAAKTLGIVL
GAFIFCWMPFFITSITDAYTGFITPAAIFEAFVWLGYFNSTLNPVIYALFYPCFKNCFYCIVTLK
IFSSNSSNMHISLK*

Labrus bergylta

>Lb-taar13a

MMVVGFSQEQFCFPESNASCVLTQFSVGTKFILYLLFSLGMLITILGNTVVIVSISHFKQLHNST
NVLILSLALVDLLVGVIVMPFSAIRTVHGCWFYGEVFCQLHSSFDMFLTSVSIFHLICIAIDRHE
AICDPLHYSMKITMPVAWIMVCCSWALAAVYSFGLLYSKANVAGLEDYIESIYCLGSCNLLFNHL
WGILDSIIAFFFPCIVMVCLYTKIFIVARDHVRKIGDMKSSSNERGKIGFIKQSEHKAAKTLGIV
LGSFIFCWMPFFLNSIADAYTGFSTPVAVFEAFVWLGYFNSTLNPIIYALFYPWFRKCFYLIINL
KIFSPNSSSIKVHTLTQL*

>Lb-taar13b

MMVVGFSQEQFCFPESNASCVLTQFSVGTKFILYLLFSLGMLITILGNTVVIVSISHFKQLHNST
SVLILSLALVDLLVGVIVMPFSAIGTVHGCWFYGEVFCQLHSSFDMFLTSVSIFHLICIAIDRHE
AICDPLHYSMKITMPVAWIMVCCSWALAAVYSFGLLYSKANVAGLEDYIESINCLGSCNLLFNHL
WEILDSIIVFFFPCIVMVCLYTQIFIVAKEHLQKIGDVKHSSKDRGRIWVIKQSEHKAAKTLGIV
LLLLDAIFL!
NSVGDAYNGFSTPVAVFETFVWLGYFNSTLNPIIYALFYPCFKKCFYRIITLKIFSPNSSTMNVS
LK*

>Lb-taar13c

MMTGGLSEEHYCFPGFNVSCVRTQFSVGTKFALFMLFALSMGLTILGNTVVIVSISHFKQLHNPT
NVLILSLALVDLLVGVIVMPFSAIRTVHGCWFYGEVFCQLHSSFDMFLTTLSIFHLICIAVDRQQ
AICDPLHYSMKITMPVAWIMVCCSWALAAVFSFGLLYSKANVAGLEDYIESINCLGSCYLLFNHL
WGILDSIIVFFFPCIVMVCLYTQIFIVAKEHLQKIGDVKHSSKDRGRIWVIKQSEHKAAKTLGIV
LGAFIFCWMPFFLNSVGDAYNGFSTPVAVFETFVWLGYFNSTLNPIIYALFYPCFKKCFYCIITL
KIFSPNSSTMNVSLK*

>Lb-taar13d

MMTGGLSEEHYCFPGFNVSCVRTQFSVGTKFALFMLFALSMGITILGNTVVIVSISHFKQLHNPT
NVLILSLALVDLLVGVIVMPFSAIRTVHGCWFYGEVFCQLHSSFDMFLTTLSIFHLICIAVDRHE
AICDPMHYSMKITMPVAWIMVCCSWALAAVYCFGLLYSKASVAGLEDYIESIYCLGSCYLLFNHL
WGILDSILSFFFSCTVMIFLYTKIFIVAKAHAQKIGDVKKGTNEKGRAGLLKQSEHKAAKTLSIV
LGAFIFCWMPFFTISLFEMILGFSTPVAVFEAFFWLGYFNSTLNPIIYALFYPCFKKCFYCIITL
KIFSPSSSTMNVAFK*

Larimichthys crocea

>Lc-taar13a

MMTGGLSQEQYCFPGYNASCVRTQFSVGTKFALSLLFTIGMLITILGNSVVIVSIRHFKQLHNPT
NLLILSLALADFLVGVIVMPFSADRTINGCWFYGEAFCLLHSSFDMFLTSVSIFHLICIAVDRQQ
AICNPLHYYRKITMSVAWIMVCGSWALAAIYSYGLLYSKANIVGLEDYILSINCLGSCNLLFNPL
WGILDSITAFFFPCTVMVCLYAKIFITAREHVRKIETSNDRERGGLIKQSEHKAAKTLGIVLGAF
ILCWMPFFINSIIDAFTGFSTPAAVFEAFVWLGYFNSTLNPIIYAFFYPWFKKCFYLIVNLKIFQ
PNSSTINVHVLTQV*

>Lc-taar13b

MMTGGLSQEQYCFPGYNASCVRTQFSVGTKFALSLLFTIGMLITILGNSVVIVSIRHFKQLHNPT
NLLILSLALADFLVGVIVMPFSADRTINGCWFYGEAFCLLHSSFDMFLTSVSIFHLICIAVDRQQ
AICNPLHYYRKITMSVAWIMICGSWALAALFSYGLLYSKANIVGLEDYILSINCLGSCNLLFNPL
WGILDSITAFFFPCTVMVCLYAKIFITAREHVRKIETSNDRERGGLIKQSEHKAAKTLGIVLGAY
IVCWMPFFINSIIDAFTGFITPAAIFESFVWLGYFNSTLNPIIYAFFYPWFKKCFYLIVNLKIFQ
PKSSTIKVHVLTHS*

>Lc-taar13c

MFWHIFRLMMTAGLSQEQYCFPGYNASCVRTQFSVGIKFALSLLFALGILVTILGNSVVIVSICH
FKQLHNPTNLLILSLALADFLVGVIVMPFSADRTINGCWFYGEAFCLLHSCLDMSLTTVSIFHLI
CIAVDRQQAICNPLHYYRKITMSVAWIMICGSWALAALFSYGLLYSKANIVGLEDYILSINCLGS
CYLLFNPLWGIVDSITCFFFPCTVMVCLYAKIFITAREHVRKIGDMNCSNDRERGGLIKQSEHKA
AKTLGIVLGAYIVCWMPFFINSIIDAFTGFITPAAIFESFVWLGYFNSTLNPIIYAFFYPWFKKC
FYLIVNLKIFQPKSSTIKVHVLTHS*

Lepisosteus oculatus

>Lo-taar13a

MDVSMLQELRKENFCYPTINGSCTREVRSLTTYILLYILLSTGMIITIFGNLVVMISITYFKQLH
TPTNMLVLSLALADFLLGMSVMPFSMIRSVETCWYYGDMFCQLHSSFDMFLTTVSIFHLIFIALD
RYQAVCNPLHYYSKITNRVAWLMIGVSWGVAAIYTYGLLYSRGNAEGLNEYVSSTYCLGSCVLFY
NALWGSLDTLIAFFLPCCIMVGLYTKIFFIARKHVVQIGTKTDQEKKDRFSEKSEWKAAKTLGIV
VGVFILCWMPFFVNSIIDPFTNFSTPAILFELFVWLGYFNSTLNPIIYAFFYPWFQKSLKLIVTF
RIFTKHSSSTKLFSEQI*

>Lo-taar5-like

MKSTDLEINLLEYCFENMTLSCIKVVRSPAIKVILYCVFLLTVLITIFGNLVVIISISHFRQLHL
PSNLLILSLATADFLLGLFVLPFSMIRTVESCWYMGSFFCKLHTSIDLLLCLASVFHVFFIAFYR
YYAVCHPLHYTSKMTTQVTFRFIVVGWVFPTICSIAFISFHYQDKETENTGSKVGCEGSCILLFN
EIFNFMDSLTFYVACLITLLMYARIFTIARKQANHISMEARTTYLEHNKIHFKGGNDRKAVKTLG
IVVGCFLFCWLPFITDKTVDPYVNFDTPAVVYDSFIWLAYFNSTFNPFIYAFFFPWFRKALKIIA
TCKIFRPNSSRIQIYTE*

Leuciscus waleckii

>Lw-taar13a

MDLSSQEYDPTELCFPAVNNSCLKGTHLVSTQTVFYLILVSAMTVTILGNSVVIISIAHFKQLQT
PTNILVMSLALADLLLGLVVMPFSMIRSVNGCWYYGDTFCLLHSSFDLFLTSVSIFHLIFIAIDR
HQAVCYPLQYPTRITIPVAWVMVMISWSMAALYSYGLVYSKANVEGLEEFIESIYCMGSCTLLFN
ALWSVLDTLLTFFLPCSVMVGLYARIFVIARKHIRKIGDANQHENESTFKSSRRSERKAAKTLGV
VVGAFILCWLPFFINSIMDPYINFSTPVALFEAFVWLGYINSTINPIIYGLFYPWF!
KTLSLIITMRIFEPNSSDITVFTV*

>Lw-taar13b

MDLSSQEYDPTELCFPAVNNSCVKGTHLVSTQTVLYLILVSAMTVTILGNSVVIISIAHFKQLQT
PTNILVMSLALADLLLGLVVMPFSMIRSVNGCWYYGDTFCLLHSSFDLFLTSVSIFHLIFIAIDR
HQAVCYPLQYPTRITIPVAWVMVMISWSMAALYSYGLVYSKANVEGLEEFIASIYCMGSCSLLFN
ALWGALDTLIGFFLPCSVMVGLYARIFVIAKKHARKLDDANQHEKENVFKSSRRSERKAAKTLGV
VVGAFIICWLRFLL!
SMLDPYINFSTPVSLFEVFVWLGYINSTIKPIIYGLFYPWFRKTLSLILTMRIFEPNSSDINVFT
V*

Morone saxatilis

>Ms-taar13a

MMTGDFSQDLYCFPGSNASCVIAQFGVGTKFALYLLFALGMLVTILGNSVIVSISHFKQLHNPTN
MLILSLALVDLLVGVIVMPFSAFWTIHGCWFYGDAFCLLHSSFDVFLTTLSIFHLICIAVDRQQA
ICNPLHYSRKITMPVAWFMICVSWTLAAVYSYGLFSKANIFNPLWGVLDSVICFFFPCTVMVCLY
TKIFIVVKEVKELKRHL

Pimephales promelas

>Pp-taar13a

MDLSSQEYDLTEFCFPAVNNSCLKGTHLVSTQTVLYLILVLAMTVTILGNSVVIISIAHFKQLQT
PTNILVMSLALADLLLGLVVMPFSMIRSVDGCWYYGDAFCLLHSTFDLFLTSVSIFHLIFIAIDR
HQAVCYPLQYPRRITMSVAWVMVLISWSMAALYSYGLLYSKANVEGMEEFIASTYCLGSCTLFFN
ALWSVLDTLLTFFLPCSVMVGLYARIFVIARKHIRTIGDAKQHENETTFKSSRRSERKAAKTLGV
VVGAFIVCWLPFFINSMMDPYINFSTPVALFEAFVWLGYINSTINPIIYGLFYPWFRKTLSLIIT
MKIFEPNSSDITVFTV*

>Pp-taar13b

MDLSSQEYDLTEFCFPAVNNSCLKGTHLVSTQTVLYLILVLAMTVTILGNSVVIISIAHFKQLQT
PTNILVMSLALADLLLGLVVMPFSMIRSADGCWYYGDAFCLLHSTFDLFLTSVSIFHLIFIAIDR
HQAVCYPLQYPRRITISVAWVMVLISWSMAALYSYGLVYSKANVEGLEEFIASTYCLGSCTLFFN
ALWGALDTLIGFFLPCSVMVGLYARIFVIAKKHARKVDNANQHEKENVFKSSRRSERKAAKTLGV
VVGAFIICWLPFFINSMIDPYINFSTPVALFEVFVWLGYINSTINPIIYGLFYPWFRKTLSLIIT
MKIFEPNSCDINVFTV*

Pygocentrus nattereri

>Pyn-taar13a

MLQATNSSFLVEADLKEYCYPESNVSCVRSFYSVAARSVLYVLLVLAMSVTILGNSVVIISIAHF
KQLHTPTNMLVMSLALADLLLGVIVMPFSMIRSVDGCWYFGEAFCLLHSSFDMLLTTASIFHLMC
VATDRYQAVCHPLQYPTRITIPTAWLMVAVSWITAAGYSYGLMYSKANVEKLDEYIESISCMGSC
NLVFNALWGALDTLIGFLLPCTVMFCLYAQIFLVSKRHARKIEGTKQSRNETSLNKVSQSMKREN
KAAKTLGIVVGAFIFCWIPFFINSLIDPYINFSTPVVLFEVFVWLGYINSTLNPIIYGLFYPWFR
KCLNLIVTLRIFAPHSSDTNVFAA*

>Pyn-taar13b

MLQATNSSFLVEADLKEYCYPESNVSCVRSFYSVAAQSVLYVLLVLAMSVTILGNSVVIISIAHF
KQLHTPTNMLVMSLALADLLLGVIVMPFSMIRSVDGCWYFGEAFCLLHSSFDMLLTTASIFHLIC
ISVDRYQAVCHPLQYPTRITIPTAWLMVAVSWITAAGYSYGLMYSKANVENLDEYIESISCMGSC
NILFNTLWGPLDVLLCFLLPCTVMFCLYAQIFLVSKRHARKIEGTKQSKTETSLNNISQSMKREN
KAAKTLGIVVGTFIFCWIPFFINSIIDPYINFSTPVVLFEVFVWLSYINSTLNPIIYGRFSPWFR
KCLYLIVTLRIFAPHSSDTNVFAA*

>Pyn-taar13c

MLQATNSSFLVEADLKEYCYPESNVSCVRSFYSVAAQSVLYVLLVLAMSVTILGNSVVIISIAHF
KQLHTPTNMLVMSLALADLLLGVIVMPFSMIRSVDGCWYFGEAFCLLHSSFDIFLTSASTFHLMC
VAIDRYQAVCHPLQYPTRITIPTAWVMVAVSWITAAGYSYGLMYSKANVVNLDEYIESISCMGSC
NLVFNALWGPLDALLCFLSPCTVMFCLYAQIFLVSKRHARKIEGTKQSRNETSLNKVSQSMKREN
KAAKTLGIVVGTFIFCWMPFYVNSLMEPHINFSTPVELFEVFVWLGYINSTLNPLIYGLFYPWFR
KCLYLIVTLRIFAPHSSDTNVFAA*

>Pyn-taar13d

MLQATNSSFLVEADLKEYCYPESNMSCVRSFYSVVARSVLYVLLVLAMSVTILGNSVVIISIAHF
KQLHTPTNMLVMSLALADLLLGVIVMPFSMIRSVDGCWYFGEAFCLLHSSIDIFLTSASTFHLMC
VATDRYQAVCHPLQYPTRITIPTAWVMVAVSWITAAGYSYGLMYSKANVVNLDEYIESISCMGSC
NLVFNALWGPLDVLLCFLSPCTVMFCLYAQIFLVSKRHARKIEGTKQSRNELSLNKVSQSMKREN
KAAKTLGIVVGTFIFCWMPYYINSLTNPYINFSTPVELFEVFVWLGYINSTLNPIIYGLFYPWFR
KCLYLIVTLRIFAPHSSDTNVFAA*

Sinocyclocheilus anshuiensis

>Sa-taar13a

MDLSSQEYDPTQFCFPAVNNSCLKGTHHVSTQTVVYLLLVLAMTVTILGNLVVIISIAHFKQLQT
PTNILVMSLALADLLLGLVVMPFSMIRSVGGCWYYGDAFCLLHSTFDLFLTSVSIFHLIFIAIDR
HQAVCYPLQYPARITIPVAWVMVMISWCMAALYSYGLVYSKANVEGLEEYIESIYCMGSCTLLFN
ALWGAIDTLIAFLLPCSVMVGLYARIFVIAKQHARKLCETNQHEKENMFKSSRRSERKAAKTLGV
VVGAFIICWLPFFINSLIDPYINFSTPIALFEVFVWLGYINSTINPIIYGLFYPWFRKTLSLIIT
RRIFEPNSSDINVFTV*

>Sa-taar13b

KFCSLLYPTRITIPVAWVMVMISWSMAALYSYGLVYSKANVEGLEEYIESIYCMGSCTLLFNALW
SV*THYLTFFLPCSVMIGLYARIFVVAKKHIRTIGEANQQENVSTFKSSRRSERKAAKTLGVVVG
AFIICWLPFFINSMDPYINFSTPFVLFEAFVWLGYINSTINPIIYDLFYPWFRKTFLSL

>Sa-taar13c

MDLSQEYDLTQFCFPAVNNSCLRGTHHVSTQTVVYLLLVSAMTVTILGNSVVIISIAHFKQLQTP
TNILVMSLALADLLLGLVVMPFSMIRSVDGCWYYGDAFCLLHSSFDLFLTSVSIFHLIFIAIDRH
QAVCYPLQYPTRITIPVAWVMVMISWSIAALYSYGLVYSKANVEGLEEYSDSMYCMGSCTLLFNA
LWSVLDTLLTFFLPCSVMIGLYARIFVVAKKHIRTIGEANQYENENMFKTSRRSERKAAKTLGVV
VGAFIICWLPFFINSLMDPYINFSTPFVLFDTFGWLGYINSTINPIIYGLFYPWFRKSLSLIVTM
RIFEPNSSDISVFTV*

>Sa-taar13d

MDLSSQEYDPTQFCFPAVNNSCLKGTHHVSTQTVVYLLLVLAMTVTILGNLVVIISIAHFKQLQT
PTNILVMSLALADLLLGLVVMPFSMIRSVIGCWYYGDAFCLLHSTFDLFLTSVSILHLVCIAIDR
HQAVCYPLQYPTRITIPVAWVMVMISWALIAVYSYGLLYSEANVEGLEEYIESIYCIGRCSLLFS
KLWSVLDTLITFFFPCSVMIGLYVRIFVVAKKHVRIISEANQHEIQNVFKSSRRSERKAAKTLGV
VVGAFIICWLPFFINSVMDPYINFSTPLALFEVFVWLGYINSTINPIIYGFFYPWFRKTLSLIIT
RRIFEPNSSDINVFTV*

>Sa-taar13e

MDLSSQEYDPTQFCFPAVNNSCLKGTHHVSTQTVVYLLLVLAMTVTILGNLVVIISIAHFKQLQT
PTNILVMSLALADLLLGLVVMPFSMIRSVGGCWYFGDAFCLLHSSFDLFLTSVSIFHLIFIAIDR
HQAVCYPLLYPTKITIPVAWVMVMISWCMAALYSYGLVYSKANVEGLEEYIESIYCMGSCTLLFN
ALWSVLDTLLTFFLPCSVMIGLYARIFVVAKKHIRTIGEANHHENESTFKSSRRSERKAAKTLGV

VVGAFIICWLPFFINSLIDPYINFSTPFALFEAFVWLGYINSTINPIIYGLFYPWFRKTLSLIIT
LKIFEPNSSDISVFTV*

Sebastes aleutianus

>Sea-taar13a

MDLSLFGFFWVISEVLAYVFELMMSFDLSQEQYCFPGSNTSCVRAHFSVGTKVALYLLFALGMLV
TILGNSVVIVSIGHFKQLHNPTNVLIVSLALVDLLVGVTVMPFSTIRTIDGCWFYGDDFCLLHSS
FDVFFTTLSIFHLICIAVDRQQAICNPLHYSRNITMSVAWIMVCASWALAAVYSYGLLYSKANVA
GLEDYMASIDCLGSCYLLFNPLWGXLDSIVCFFFPCTVMVCLYTKIFIVAKEHVRKIGDMHNRSN
DRVGGGLIKQSEHKAAKTLGIVLGAYIFCWMPFFINSIIDVYIGFSTPVAIFEAFVWLGYFNSTL
NPIIYAFFYPCFKKCLYCIVTLKAFPSNP*

>Sea-taar13b

MSLDLSQEQYCFPGSNASCVRAHFSIGTKVALYLLFTLGMLVTILGNSVVIVSIGHFKQLHNPTN
VLIVSLALADLLVGLTVVPFSTIRTIHGCWFYGDDFCMLHSCFDMFLTTVSIFHLICIAVDRQQA
ICNPLHYSSNITMSVAWIMVCASWALAAVYSYGLLYSKANVAGLEDYIASIDCLGSCNILFNSLW
GILDSIASFFFPCTVMVCL

>Sea-taar13c

MTFGLSEEXYCFPGSNALCVREQFSVGAKVALYLLFT!
RMLVTILGNSVVIVSIGHFKQLHNPTNVLIVSLALVDLLVGVTVMPFSTIRTIHGCWFYGDDFCM
LHSSFDMFLTTVSIFHLICIAVDRQQAICNPL!
YSSNITMSVAWIMVCASWALAAVYTYGLLYSKANVAGLEDYMASIDCLGSCNLLFNSLWGILDS

Sebastes minor

>Sem-taar13a

ILGNSVVIVSIGHFKQLHNPTNVLIVSLALVDLLVGVTVMPFSTIRTIHGCWFYGDDFCLLHSSF
DVFFTTLSIFHLICIAVDRQQAICNPLHYSRNITISVAWIMVCASWALAAVYSYGLLYSKANVAG
LEDYMASIDCLGSCYLLFNPLWGVLDSIVCFFFPCTVMVCLYTKIFIVAKEHVRKIGDMHNxxxK
QSEHKAAKTLGIVLGAYIFCWMPFFINSIIDVYIGFSTPVAIFEAxxxLGYFNSTLNPIIYAFFY
PCFKKCLYCIVTLKAFPSNP*

>Sem-taar13b

VTILGNSVVIVSIGHFKQLHNPTNVLILSLALADLLVGLTVVPFSTIRTIHGCWFYGDDFCMLHS
SFDMFLTTVSIFHLICIAVDRQQAICNPLHYSSxxxSVAWIMVCASWALAAVYSYGLLYS

Sebastes nigrocinctus

>Sn-taar13a

MDLSLFGFCWVICEVLVYLFELMMSFDLSQEQYCFPGSNASCVRAHFSVGTKVALYLLFTLGMLV
TILGNSVVIVSIGHFKQLHNPTNVLIVSLALVDLLVGVTVMPFSTIRTIHGCWFYGDDFCLLHSS
FDVFFTTLSIFHLICIAVDRQQAICNPLHYSRNITMSVAWIMVCASWALAAVYSYGLLYSKANVA
GLEDYMASIDCLGSCYLLFNPLWGVLDSIVCFFFPCTVMVCLYTKIFIVAKEHVRKIGDMHNRSN

VREGGGLIKQSEHKAAKTLGIVLGAYIFCWMPFFINSIIDVYIGFSTPVAIFEAFVWLGYFNSTL
NPIIYAFFYPCFKKCLYCIVTLKAYPSNP*

Sebastes rubrivinctus

>Ser-taar13a

MDLSLFGFCWVICEVLVYLFELMMSFDLSQEQYCFPGSNASCVRAHFSVGAKVALYLLFTLGMLV
TILGNSVVIVSIGHFKQLHNPTNVLIVSLALVDLLVGVTVMPFSTIRTIHGCWFYGDDFCLLHSS
FDVFFTTLSIFHLICIAVDRQQAICNPLHYSRNITMSVAWIMVCASWALAAVYSYGLLYSKANVA
GLEDYMASIDCLGSCYLLFNPLWGVLDSIVCFFFPCTVMVCLYTKIFIVAKEHVRKIGDMHNRSN
VREGGGLIKQSEHKAAKTLGIVLGAFIFCWMPFFINSIIDVYIGFSTPVAIFEAFVWLGYFNSTL
NPIIYAFFYPCFKKCLYCIVTLKAYPSNP*

>Ser-taar13b

MMSFVLSQEQYCFPGSNASCVREQFSVGTKVALYLLFTLGMLVTILGNSVVIVSIGHFKQLHNPT
NVLILSLALSDLLVGVTVMPFSTIRTIHGCWFYGTKVALYLLFTLGMLVTILGNSVVIVSIGHFK
QLHNPTNVLILSLALSDLLVGLTVMPFSTIRTIHGCWFYSDDFCLLHSSFDMFLTSVSIFHLICI
AVDRQQAICNPLHYSRNITMSVAWIMVCASWALAAVYSYGLLYSKANVAGLEDYIASIDCLGSCN
LLFNSLWGILDSITAFFFPCTVMVCLYTKIFFVAKEHVRKIGX

Sebastes steindachneri

>Ses-taar13a

NPTNVLIVSLALVDLLVGVTVMPFSTIRTIHGCWFYGDDFCMLHSSFDVFFTTLSIFHLICIAVD
RQQAICNPLHYSRNITMSVAWxxxKANVAGLEDYIASIDCLGSCYLLFNPLWGVLDSIVCFFFPC
TVMVCLYTKIFIVAKEHVRKIGDMHNRSNDRVGGGLIKQSEHKAxxxIFCWMPFFINSIIDVYIG
FSTPVAIFEAFVWLGYF

Scleropages formosus

>Sf-taar13a

MNISREPDAEQYCYPASNRTCTKYIHSAAAYAALYAFFALGSAVTVTGNLVVLVSIAHFRQLHTP
ANMLIMSLAMADLLVGILVMPFSAIRTIEGCWYFGEAFCLLHSTFDLFLTSVSIFHLISIAIDRY
QAVCNPLRYPTTITIPVVWLMIFLSWGAAAAYSYSLLYTKANVAGLEEYIALINCLGSCNLLFNA
LWGALDSLVGFFLPCSVMMGLYAKIFLVARQHVRRLGDNSHQAHMNTENTIRKNHSSERKAARTL
GIVLGVFFLCWMPFFINSVIDPYINFGTPSIVFELLVWLAYFNSTLNPIIYALFYPWFQKSLKFI
VTMKIFTPHSSDFNVFPER*

>Sf-taar13b

MNISREPDAEQQYCYPAPNRTCTKYIHSAAAYAALYAFFALGSAVTVTGNLVVLVSIAHFRQLHT
PANMLIMSLAMADLLVGILVMPFSAIRTIEGCWYFGEAFCLLHSTFDLFLTSVSIFHLISIAIDR
YQAVCNPLRYPTTVTIPKAWLMVFLSWVAAAAYSYSLLYTKANVAGLGGYIALIKFF!
LGSCNLLFNALWGALDSLVGFFLPCSVMMGLYAKIFLVARQHVRRLGDNSHQAHMNMENTIRKNH
SSERKAARTLGIVMGAFFLCWMPIFINSIIDPYINFGTPSIVFELLVWLAYFNSTLNPIIYALFY
PWFQKSLKFIVTMKIFTPHSSDFNVFPER*

>Sf-taar13c

MNISREPDAEQYCYPASNRTCTKYIHSAAAYAALYAFFALGSAVTVTGNLVVLVSIAHFRQLHTP
ANMLIMSLAMADLLVGILVMPFSAIRTIEGCWYFGEAFCLLHSTFDLFLTSVSIFHLISIAIDRY
QAVCNPLRYPTTITIPVAWLMIFLSWGVAAAYSYSLLYTKANVAGLEEYIALINCLGSCNLLFNA
LWAALDSTTEFFLSFSVMMGVYAKIFLVARQHVRRLGDNSHQAHMNTENTIRKNHSSERKAARTL
GIVMGAFFLCWMPFFINSVIDPYINFGTPSIVFEVLVWLGYFNSTLNPIIYALFYPWFQKSLKFI
VTLKIFTPHSSDFNVFPER*

>Sf-taar13d

MNISREPDAEQYCYPASNRTCTKYIHSAAAYAALYAFFALGSAVTVTGNLVVLVSIAHFRQLHTP
ANMLIMSLAMADLLVGILVMPFSAIRTIEGCWYFG!
AWLMVFLSWGAAAAYSYSLLYTKANVAGLEEYIALINCLGSCNLLFNALWAALDTLAEFFLSCCV
MIGVYAKIFLVARQHVRRLGDNSHQAHMNTENTIRKNHSSERKAARTLGIVMGAFFLCWMPFFIN
SIIDPYINFGTPSIVFEVLVWLGYFNSTLNPIIYALFYPWFQKSLKFIVTMKIFTPHSSDFNVFP
ER*

Sinocyclocheilus grahami

>Sg-taar13a

MDLSSKEYDPTQFCYPAVNNSCLKGTHHVSTQTVVYLLLVLAMTVTILGNLVVIISIAHFKQLQT
PTNILVMSLALADLLLGLVVMPFSMIRSVGGCWYYGDAFCLLHSTFDLFLTSVSIFHLIFIAIDR
HQAVCYPLQYPARITIPVAWVMVMISWSMAALYSYGLVYSNANVEGLEEYIESIYCMGSCTLLFN
ALWGAIDTLIAFLLPCSVMVGLYARIFVIAKQHARKLCETNQHENENMFKSSRRSERKAAKTLGV
VVGAFIICWLPFFINSLIDPYINFSTPIALFEVFVWLGYINSTINPIIYGLFYPWFRKTLYLIIT
RRIFEPNSSDINVFTV*

>Sg-taar13b

FHLKGILKFCSLLYPTRITIPVAWVMVMISWSMAALYSYGLVYSKANLEGLEEYSDSIYCMGSCT
LLFNALWSVLDTL!TFFLPCSVMIGLYARIFVV!
KKHIRTIGEANQNENVSTFKSSRWSECKAAKTLGVVVGAFIICWLPFFINSMDPYINLSTPFVLF
EAFVWLGYINSTINPIIYGLFYPWFRKNLSVIITMKIFEPNSSDISVFTV*

>Sg-taar13c

MDLSQEYDLTQFCFPAVNNSCLRGTHHVSTQTVVYLLLVSAMTVTILGNSVVIISIAHFKQLQTP
TNILVMSLALADLLLGLVVMPFSMIRSVDGCWYYGDAFCLLHSSFDLFLTSVSIFHLIFIAIDRH
QAVCYPLQYPTRITIPVAWVMVMISWSIAALYSYGLVYSKANVEGLEEYSDSIYCMGSCTLLFNA
LWSVLDTLLTFFLPCSVMIGLYARIFVVAKKHIRTIGEANQHENENMFKTSRRSERKAAKTLGVV
VGAFIICWLPFFINSLMDPYINFSTPFVLFDTFGWLGYINSTINPIIYGLFYPWFRKSLSLIITM
RIFEPNSSDISVFTV*

>Sg-taar13d

MDLSSQEYDPTQFCFPAVNNSCLKGTHHVSTQTVVYLLLVLAMTVTILGNLVVIISIAHFKQLQT
PTNILVMSLALADLLLGLVVMPFSMIRSVGGCWYYGDAFCLLHSTFDLFLTSVSILHLVCIAIDR
HQAVCYPLQYPARITIPVAWVMVMISWALIAIYSYSLLYSEANVEGLEEYIESIYCIGHCSLLFS
KLWSVLDTLITFFFPCSVMIGLYVRIFVVAKKHVRIISEANQHEIENVFKSSRRSERKAAKTLGV

VVGAFIICWLPFFINSVMDPYINFSTPLALFEVFVWLGYINSTINPIIYGFFYPWFRKTLSLIIT
KRIFEPNSSDINVFTV*

>Sg-taar13e

MDLSSQEYDPTQFCFPAVNNSCLKGTHHVSTQTVVYLLLVLAMTVTILGNLVVIISIAHFKQLQT
PTNILVMSLALADLLLGLVVMPFSMIRSVGGCWYYGDAFCLLHSSFDLFLTSVSIFHLIFIAIDR
HQAVCYPLLYPTKITIPVAWVMVMISWCMAALYSYGLVYSKANVEGLEEYIESIYCMGSCTLLFN
ALWSVLDTLLTFFLPCSVMIGLYAIIFVVARKHIRTIGEANHHENESTFKSSRRSERKAAKTLGV
VVGAFIICWLPFFINSLIDPYINFSTPFALFETFVWLGYINSTINPIIYGLFYPWFRKTLSLIIT
LKIFEPNSSDISVFTV*

Sinocyclocheilus rhinocerous

>Sr-taar13a1

MDLSSQEYDPTQFCYPAVNNSCLKGTHHVSTQTVVYLLLVLAMTVTILGNLVVIISIAHFKQLQT
PTNILVMSLALADLLLGLVVMPFSMIRSVGGCWYYGNAFCLLHSTFDLFLTSVSIFHLIFIAIDR
HQAVCYPLQYPARITIPVAWVMVMISWSMAALYSYGLVYSKANVEGLEEYIESIYCMGSCTLLFN
ALWGAIDTLIAFLLPCSVMIGLYARIFVIAKQHARKLCETNQHENENMFKSSRRSERKAAKTLGV
VVGAFIICWLPFFINSLIDPYINFSTPIALFEVFVWLGYINSTINPIIYGLFYPWFRKTLSLIIT
RRIFEPNSSDINVFTV*

>Sr-taar13a2

SEANVEGLEEYIESMYCKGSCTLLFNVVWGAIDT*IAFLLPCSVMVGLYARIFVIVKQHARKLCK
DKNENMFKSS*RSEHKAAKTLSVVVGAFIICWLPFFINSLIDPYINFSTPLVLFEVFVWLGYINS
TINPIIYGFFWYRKTLSLIITSNKFITSSSDINVFTV*

>Sr-taar13b

KFCSLLYPTRITIPVAWVMVMVSWSMAALYSYGLVYSKANVEGLEEYIESIYCMGSCTLLFNALW
SVLDTL!
TFFLPCSVMIGLYARIFVVAKQHIRTIGEANQHENVSTFKSSRQSECKAAKSLGVVMGAFIICWL
PFFINSMDPYINFSTPFALFEAFVWLGYINSTINHIIYGLFYPWFKKNLSVIITLKIFEPNSSDI
SVFTV*

>Sr-taar13c1

FWNPVCYPLQYPTRITIPVAWVMVMISWSMAALYSYGLVYSKANVEGLEEYSDSIYCMGSCTLLF
NALWSVLDTLLTFFLPCSVMIGLYARIFVVAKKHIRTIGEANQHENENMFKTSRRSERKAAKTLG
VVVGAFIICWLPFFINSLMDPYINFSTPFVLFDTFGWLGYINSTINPIIYGLFYPWFRKSLSLII
TMRIFEPNSSDISVFTV*

>Sr-taar13c2

MDLSQEYDLTQFCFPAVNNSCLRGTHHVSTQTVVYLLLVSAMTVTILGNSVVIISIAHFKQLQTP
TNILVMSLALADLLLGLVVMPFSMIRSVDGCWYYGDAFCLLHSSFDLFLTSVSIFHLICIAIDRH
QAVCYPLQYPTRITIPVAWVMV

>Sr-taar13d

MDLSSQEYDPTQFCFPAVNNSCLKGTHHVSTQTVVYLLLVLAMTVTILGNLVVIISIAHFKQLQT
PTNILVMSLALADLLLGLVVMPFSMIRSVGGCWYYGDAFCLLHSTFDLFLTSVSILHLVCIAIDR
HQAVCYPLQYPTRITIPVAWVMVMISWALIAVYSYGLLYSEANVEGLEEYIESIYCIGRCSLLFS
KLWSVLDTLITFFFPCSVMIGLYVRIFVVAKKHVRIISEANHHETENVFKSSRRSERKAAKTLGV
VVGAFIICWLPFFINSVMDPYINFSTPLALFEVFVWLGYINSTINPIIYGFFYPWFRKTLSLIIT
RRIFEPNSSDINVFTV*

>Sr-taar13e

MSLFGKDATSSSHLALGILCHSSLQILSSSVRLDGKRWWTAIFRSLQRCSIGFNGDAFCLLHSSF
DLFLTSVSIFHLIFIAIDRHQAVCYPLLYPTKITIPVAWVMVMISWCMAALYSYGLVYSKANVEG
LEEYIESIYCMGSCTLLFNALWSVLDTLLTFFLPCSVMIGLYARIFVVARKHIRTIGEANHHENE
STFKSSRRSERKAAKTLGVVVGTFIICWLPFFINSLIDPYINFSTPFALFEAFVWLGYINSTINP
IIYGLFYPWFRKTLSLIITLKIFEPNSSDISVFTV*

Salmo salar

>Ss-taar13a

MDISSHQHLDPKMFCYPESNASCTRDIFSGGVQIALYFLFVSGMLVTILGNGVVIISIAHIKQLH
TPTNMLIMSLAVADLLLGVTVMPFSTMRAVEGCWYFGDAFCLLHSSFDMFLTSVSIFHLVFIAID
RYEAVCNPLRYSTKITIPIAWLMVLASWAVAALYSYCLLYSKANVRGLDEFIASIYCLGSCNLVF
NALWGALDTLIAFFFPCSVMVGLYTKIFLVAKEHLRKIEDSQKNSNERDKGVVSQRSERKAAKTL
GIVVGVFIFCWLPFFVNSIVDPYTNFSTPPILFEVFIWLGYFNSTANPIIYGLFYPWFRKCLNLI
VTLKIFNRNSSYINVFATT*

>Ss-taar13b

MNISSHQHLDPKMFCYPESNASCTRDIFSGGVQIALYFLFVSGMLVTILGNSVVIISIAHIKQLH
TPTNMLIMSLAVADLLLGVTVMPFSTMRPVEGCCYFGDAFCLLHSDMFLTSVSIFHLVFIAIDRY
EAVCNPLRYSTKITIPIAWLMVLASWAVAALYSYCLLYSKANVRGLDEFIASIYCLGSCNIVFNA
LWGTLDTLIAFFFPCSVMVGLYTKILLVAKEHLRKIDDSQKNSNERDKGVVSQRSERKAAKTLGI
VVGVFIFCWLPFFVNSIVDPYTNFST!
YSLFEVFIWLGYFNSTANPIIYALFYPWFRKCLNLIVTLKIFNRNSSYINVFAT

>Ss-taar13c

MDISSHQHLDPKLFCYPESNASCTREIVS*GVQIALYFLFVSGMLVTILGNSVVIISIAHIKQLH
TPTNMLIMSLAVADLLLGVTVMPFSTMRPVEGCCYFGDAFCLLHSDMFLTSVSIFHLVFIAIDRY
EAVCNPLHYSTKITIPIAWLMVFASWAVAALYSYCLLYSKANVRGLDEFIASIYCLGSCNIVFNA
LWGALDTLIAFFFPCSVMVGLYTKILLVAKEHLRKIEDSQKNSNERDKGVVSQRSERKAAKTLGI
VVGVFIFCWLPFFVNFIVDPYTNFSTPPILFEVFIWLGNFNSTANPIIYALFYPWFRKCLNLIVT
FYLFLIIYLFHLYLTR*

Reference TAAR sequences from all classes (class I, II, III) in several bony vertebrate species (zebrafish, stickleback, fugu and mouse)

Amino acid sequences were taken from Hussain et al., 2009, see also Suppl. Table 1.

>Mm-taar1

```
MHLCHAITNISHRNSDWSREVQASLYSLMSLIILATLVGNLIVIISISHFKQLHTPTNWLLHSMA
IVDFLLGCLIMPCSMVRTVERCWYFGEILCKVHTSTDIMLSSASIFHLAFISIDRYCAVCDPLRY
KAKINISTILVMILVSWSLPAVYAFGMIFLELNLKGVEELYRSQVSDLGGCSPFFSKVSGVLAFM
TSFYIPGSVMLFVYYRIYFIAKGQARSINRTNVQVGLEGKSQAPQSKETKAAKTLGIMVGVFLVC
WCPFFLCTVLDPFLGYVIPPSLNDALYWFGYLNSALNPMVYAFFYPWFRRALKMVLLGKIFQKDS
SRSKLFL
```

>Mm-taar2

```
MASFEAQQETFDCSEYGNGSCPENERSLGVRAAMYSLMACAIFITIFGNLAMIISISYFKQLHTP
TNLLILSMAVTDFLLGFTIMPYSMVRSVENCWYFGLTFCKIHYSFDLMLSITSIFHLCSVAVDRF
YAICHPLHYCTKMTIPVVRRLLLVCWSVPGAFAFGVVFSEAYADGIEGYDILVACSSSCPVMFNK
LWGTTLFVAGFFTPSSMMVGIYGKIFAVSKKHARVIDNLPENQNNQMRKDKKAAKTLGIVMGVFL
LCWFPCFFTILLDPFLNFSTPAVLFDALTWFGYFNSTCNPLIYGFFYPWFRRALKYILLGKIFSS
HFHNTNLFTQKETE
```

>Mm-taar3

```
MDLIYIPEDLSSCPKFGNKSCPPTNRSFRVRMIMYLFMTGAMVITIFGNLVIIISISHFKQLHSP
TNFLILSMATTDFLLGFVIMPYSMVRSVESCWYFGDSFCKFHASFDMMLSLTSIFHLCSIAIDRF
YAVCDPLHYTTTMTVSMIKRLLAFCWAAPALFSFGLVLSEANVSGMQSYEILVACFNFCALTFNK
FWGTILFTTCFFTPGSIMVGIYGKIFIVSRRHARALSDMPANTKGAVGKNLSKKKDRKAAKTLGI
VMGVFLACWLPCFLAVLIDPYLDYSTPIIVLDLLVWLGYFNSTCNPLIHGFFYPWFRKALQFIVS
GKIFRSNSDTANLFPEAH
```

>Mm-taar4

```
MNTPDPWSSPEVQFCFAAANSSCPRKARPALVVCAMYLIMIGAIVMTMLGNMAVIISIAHFKQLH
SPTNFLILSMATTDFLLSCVVMPFSMIRSIESCWYFGDLFCKVHSCCDIMLCTTSIFHLCFISVD
RHYAVCDPLHYVTQITTRVVGVFLLISWSVPIFFAFGLVFSELNLIGAEDFVAAIDCTGLCVLIF
NKLWGVLASFIAFFLPGTVMVGIYIHIFTVAQKHARQIGTGPRTKQALSESKMKATSKKESKATK
TLSIVMGVFVLCWLPFFVLTITDPFIDFTTPEDLYNVFLWLGYFNSTFNPIIYGMFYPWFRKALR
MIVTGTIFRSDSSTSSLHPAHP
```

>Mm-taar5

```
MRAVLLPGSGEQPTAFCYQVNGSCPRTVHPLAIQVVIYLACAVGVLITVLGNLFVVFAVSYFKVL
HTPTNFLLLSLALADMLLGLLVLPLSTVRSVESCWFFGDFLCRLHTYLDTLFCLTSIFHLCFISI
DRHCAICDPLLYPSKFTVRTALRYIVAGWGIPAAYTAFFLYTDVVERALSQWLEEMPCVGSCQLL
FNKFWGWLNFPAFFVPCLIMISLYLKIFVVATRQAQQIRTLSQSLAGAVKRERKAAKTLGIAVGI
```

YLVCWLPFTVDTLVDSLLNFITPPLVFDIFIWFAYFNSACNPIIYVFSYRWFRKALKLLLSREIF
SPRTPTVDLYHD

>Mm-taar6

MGSNSSPPTVLQLCYENVTGSCVKTPYSPGSRVILYAVFGFGAVLAVFGNLMVMISILHFKQLHS
PTNFLIASLACADFGVGISVMPFSMVRSIESCWYFGRSFCTFHTCCDVAFCYSSLFHLSFISIDR
YIAVTDPLVYPTKFTVSVSGICIGVSWILPLVYSGAVFYTGVYDDGLEELSSALNCVGGCQVVVN
QNWVLIDFLSFLIPTLVMIILYGNIFLVARQQAKKIENIGSKTESSSESYKARVARRERKAAKTL
GITVVAFMISWLPYSIDSLVDAFMGFITPAYIYEICVWCAYYNSAMNPLIYALFYPWFKKAIKVI
MSGQVFKNSSATMNLFSEQI

>Mm-taar8a

MTSNFSQPALQLCYENTNGSCIKTPYSPGPRVILYMVYGFGAVLAVCGNLLVVISVLHFKQLHSP
ANFLIASLASADFLVGISVMPFSMVRSIESCWYFGDAFCSLHSCCDVAFCYSSVLHLCFISVDRY
IAVTDPLVYPTKFTVSVSGICISISWILPLVYSSAVFYTGISAKGIESLVSALNCVGGCQIVINQ
DFVLISFLLFFIPTLVMIILYSKIFLVAKQQAVKIETSVSGNRGESSSESHKARVAKRERKAAKT
LGVTVVAFMVSWLPYTIDALVDAFMGFITPAYVYEICCWGTYYNSAMNPLIYAFFFPWFKKAIKL
ILSGEILKGHSSTANLFSE

>Mm-taar9

MTSDFSPEPPMELCYENVNGSCIKSSYAPWPRAILYGVLGLGALLAVFGNLLVIIAILHFKQLHT
PTNFLVASLACADFLVGVTVMPFSTVRSVESCWYFGESYCKFHTCFDTSFCFASLFHLCCISIDR
YIAVTDPLTYPTKFTVSVSGLCIALSWFFSVTYSFSIFYTGANEEGIEELVVALTCVGGCQAPLN
QNWVLLCFLLFFLPTVVMVFLYGRIFLVAKYQARKIEGTANQAQASSESYKERVAKRERKAAKTL
GIAMAAFLVSWLPYIIDAVIDAYMNFITPAYVYEILVWCVYYNSAMNPLIYAFFYPWFRKAIKLI
VSGKVFRADSSTTNLFSEEAGAG

>Dr-taar1b

MDLCYEAMNGSCWKYVRPHAIHVPMLIAIMLIISMTFIGNLLVIISIGHFRQLHTPTNQLILSLA
LCDFLIGLFVMPLSAVRSMQGCWYFGEFLCKLHTCIDITLSTSSIFHLVSVSAERFCAVCGPLRY
RSCFGLSTVLLMISISWLIPGIFAYVMTFLEINIHGGKDFYDAHVRCVGGCHVFFSHGPAVFTSV
FSFYIPGFIIVVIYSRIYMVARNQERSIRLQLNQLRRVYPSRDVQLQTRKATVTIAIVVGAFLVC
WTPFFLCNILNPFIGYATPPMLIDVLMWFGYANSTLNPFIYAFMHSWCRKAVRIIVTGEIFKNNS
SRKDLYT

>Dr-taar10a

MDDHINISQTESWEKPLLCYEFSNRSCQKFVYPLDTRILLYMLFSISSIITIIGNLLVIITVVHF
RQLHTPTNYLILSLAVADLLVGGVVMPPSMLRSIETCWYLGDLFCKIHSSLDVTLCTASILNLCI
ISLDRYYAICHPFQYHSKMTSLATLIMIIICWTVSAVLGFGMIFMELNILGVEDFYYENIRCDGG
CFVFQSKTGGTVFSLICFYIPAFVMLGVYLKILHEAQRQVQAIQSVNSELKKEGKATKTLAIIMG
VFLTFWIPFFLCNLIDPLIGYSVPSLVFDLFLWVGYYNSTCNPIVYAFFYSWFRHAFRVILSKRV
FQTNSSRTILM

>Dr-taar11

MMNQSHQWSAETFSLCFESINDSCVKTVYSPVFRAPLYLLFIIAIILIVFGNLWVICTISFFQQL
HTPTNYLILSMAVSDLLLGSFVMPPSMLRSLETCWYFGDFFCKFHSATDFTLCNASVLHLVFISI
DRYYAVCQPFHYQSRMTTRVSVFMILISWSFSAFFGFGIIFSELKIEKKRTEELHVACKGGCLAL
HGREIGVTYSLVFYFLPMFIIVSLYSRVFIIALKHVRVINSAASSLSATKMDLKATKTLAIIIGV
FMSCWTPYFMCNIIDPIVNHTIPALLYEVLMWVAYLNAVFNPLVYAFFYSWFRDKSKLLLEKLYK
LC

>Dr-taar12a

MIKKKIIQDVMTSNQTQTENILLCYPLLSNSCPKLHRLAVVQVGLYVFLLLMILTTVFGNLMIII
SISHFKHLQSPTHLIVQSLAACDCLMGSLVMPYSMVRSVEGCWYLGDVVCKVHFSFDVTFCISSL
LHLCLISVDRYLAICDPLRYKIRVTNTTMTVFIIFIWLFSVVYSFSIVFSGITAVGLEMLILQTY
CVGSCVLFFNKEWAVYPFLTFFITGAIMSSLYMKIFHVARKHAKVMSERVKGGLKSQRSAQRERK
AAKTLAIVMGVFMFCWLPYCAFTALYPFFTFLNSAEVFDVLFWFAYFNSSCNPLIYGFFYPCFQK
AFKILIFLWSQKCKHFSF

>Dr-taar12b

MTSNETQTDNILLCYPLLSNSCPKLHRLAVVQVGLYVCLLLMILTTVFGNLLIIISISHFKHLQS
PTHLIVQSLAACDCLMGSLVMPYSMVRSVEGCWYLGDVVCKVHSSLDMTFCMSSLLHLGLISVDR
YWAICDPLRYRLRVTNTTVTVFIIVIWLFSFIYNFSIVFSGITAVGLEMLILQTYCVGSCIVLFN
KEWAVYPFLTFFITGAIMSSLYMKIFHVAQKHAKVMSERVTGGLKSQSSAQRERKAAKTLAIVMG
VFMFCWLPYCAFTALYPFFTFLNSAEVFDVLFWFAYFNSSCNPVIYGFFYPCFQKAFKILISTYI
FGIRNANTSTYE

>Dr-taar12c

MTSNETQTDNVHLCFPLLPNSCSKLHHFVVVKVAMYVCLLLMILTTVFGNLLIIISISHFKHLQS
PTHLIVRSLAASDCLLGSLVMPYSMVRSVEGCWYLGDVVCKVHSSLDMTFCISSLLHLGLISVDR
YWAICDPLRYRLRVTNTTVTVFTVFIWLFAFVYSFSVVFSGIAAVGLEMLILQTYCVGSCVLFFN
KEWGLICPILTFFLPGAIMSFLYMKIFHVARKHAKVMSERVTGGLKSQSSAQRERKAAKTLAIVM
GVFMFCWLPYFTVTILGPFFNFATPADVFDALVWFAYLNSTCNPLIYGFFYPCFQKAFHILISTY
ICGVRNSDTLIYE

>Dr-taar12d

MTSNETQTDNVHLCFPLLPNSCSKLHHFVVVKVAMYVCLLLMILTTVFGNLLIIISISHFKHLQS
PTHLIVRSLAASDCLLGSLVMPYSMVRSVEGCWYLGDVVCKVHSSLDMTFCISSLLHLGLISVDR
YWAICDPLRYRLRVTNTTVTVFTVFIWLFAFVYSFSVVFSGIAAVGLEMLILQTYCVGSCVLFFN
KEWGLICPILTFFLPGAIMSFLYMKIFHVARKHAKVMSERVKGGLKSQSSAQRERKAAKTLAIVM
GVFMFCWLPYCAFTALYPFFTFLNSAEVFDVLFWFAYFNSSCNPLIYGFFYPCFQKAFKILIFLW
SQKCKHFSF

>Dr-taar12e

MMSLNETQTENILLCYPLLSNSCPKLHRLAVVQVGLYVFLLLMILTTVFGNLLIIISISHFKHLQ
SPTHLIVRSLAACDCLLGLLVMPYSMVRSVEGCWYLGDVVCKVHSSLDMTFCISSLLHLGLISVD
RYWAICDPLRYRLRVTNITVTVFIVFIWLFSFVYSFYVVFSGVNTVGLETFIMQVYCVGSCVLYF
NKQWGLICPILVFFLPGAIMSSLYMKIFHVARKHAKVMSERVTGGLKSQSSAQRERKAAKILAIV

MGVFLFCWLPFFTVNALDPFFNFFTPADIFDAVIWFAYLNSTCNPLIYGFFYPCFQKAFKILIFT
YICGVRNLDTFA

>Dr-taar12f

MKPSNETQTENILLCYPLLSNSCPKLHRLAVVQVGLYVFLLLMILTTVFGNLLIIISISHFKHLQ
SPTHLIVRSLAACDCLLGSLVMPYSMVRSVEGCWYLGDVVCKVHSSLDMTFCISSLLHLGLISVD
RYWAICDPLRYRLRVTNTTVTVFIVFIWLFSFVYSFYVVFSGVNTVGLETFIMQVYCVGNCVLYF
NKQWGLICPILTFFLPGTIMSSLYMKIFHVARKHAKVMSERVTGGLKSQSSAQRERKAAKILAIV
MGVFLFCWLPFFTVNALDPFFNFFTPADIFDAVIWFAYLNSTCNPLIYGLFYPCFQKAFKILIST
YLCGVRNLDTFA

>Dr-taar12g

MTSNESENIQLCYPLLSNSCPKLHRLAVVQVGLYICLLLMILTTVFGNLLIIISISHFKHLQSPT
HLIVRSLAACDCLLGSLVMPNSMVRSVEGCWYLGDVVCKVHSSLDMTLCISSLLHLGLISVDRYL
AICDPLRYRIRVTNTTVTVFTVFIWLFSVVYSFYVKFSGITAVGLEMLILQTYCVGRCVVFFNKQ
WGLICPVLAFFLPGAIMSSLYMKIFHVARKQAKVISERVTGGLKSQSSAQRERKAAKTLAIVMGV
FLFCWMPFFTLTALDPFFNFLSSADVFDALFWFAYLNSACNPLIYGFFYPCFQKAFKILIFTYIC
GVKKANTLTFE

>Dr-taar12h

MILNDTDIYSENVLLCYPLLPDSCPRTQRLPALKVAMYAVMVLMILTTVFGNLLVIISISHFKQL
QSPTHLIVQSLAACDCLLGSLVMPYSMVRSVEGCWYLGTVVCKVHSSLDMTFSISSILHLSLIAI
DRFWAISDPLRYKMRVTNTTVAGFITFTWLFSFVYSFSVVFTGVNNVGLEELILQISCFGGCVLF
FNKEWGLICALFVFLIPGTIMSSLYMSIFNVVKRHAKVMSEKVSAAATAGSHFQTSSHRERKAAK
TLAIVMGVFYLCWLPFFTATAVDPFLNFSTPGDVFDALVWFGYFNSTCNPLIYGFFYPRFQKAFK
ILISTYICGSSDSHTLTFE

>Dr-taar12i

MTSNETQTDNVHLCFPLLPNSCSKLHHFVVVKVAMYVCLLLMILTTVFGNLLIIISISHFKHLQS
PTHLIVRSLAASDCLLGSLVMPYSMVRSVEGCWYLGDVVCKVHSSLDMTFCISSLLHLGLVSIDR
YWAICDPLRYRIRVTNTTVTLYTIFIWLFSFLYSFVIVFSGVNRIGLETFIMQVYCVGSCVLFFN
KEWGLICPVLTFFIPGAIMSSLYMKIFHVARKHAKVMSERVTGGLKSQSSAQRERKAAKTLAIVM
GVFLLCWTPIFIASIIDSFLNFVTPAGVFDALIWFGYFNSTCNPLIYGFFYSCFQKAFKILLSSL
FLGFGNSSTLTFE

>Dr-taar12j

MTSNDTDVHSENVLLCYPLLSDSCPRTQRLTALTVTMYAFMVLMILTTVFGNLLIIISISHFKQL
QSPTHLIVRSLAASDCLLGSLVMPYSMVRSVEGCWYLGDVVCKVHSSLDMTFSISSLIHLSLVSV
DRYWAICDPLRYRMRVTNNTVIVFTVFTWLFSFVYSFSVVFSGVNRIGLETFIMQVYCVGSCVLF
FNKQWGLICSLLTFFLPCTIMSSLYMKIFHVARKHAKVMSERVTGGLKSQSSAQREGKAAKTLAI
VMGVFYLCWLPFFTATAVDPFLNFSTPVHVFDALVWFGYFNSTCNPLIYGFFYPRFQKAFNILIS
TYICGSSDSHTLIPE

>Dr-taar12k

MTSNQTQTENILLCYPLLSNSCPKLHRLAVVQVGLYVFLLLMILTTVFGNLMIIISISHFKHLQS
PTHLIVQSLAACDCLMGSLVMPYSMVRSVEGCWYLGDVVCKVHFSFDVTFCISSLLHLCLIAIDR
FWAISDPLRYKMRVTNTTVAGFITFTWLFSFVYSFSVVFTGVNNVGLEELILQISCFGGCVLFFN
KEWGLICALFVFLIPGTIMSSLYMSIFNVVKRHAKVMSEKVSAAATAGSHFQTSSHRERKAAKTL
AIVMGVFYLCWLPFFTATAVDPFLNFSTPGDVFDALVWFGYFNSTCNPLIYGFFYPRFQKAFKIL
ISTYICGSSDSHTLTFE

>Dr-taar12m

MAANETDVYAENVFLCFPLRLDSCLSAQRLTTVKVAMYILMLLIIFLTVFGNLLVVVSISYFKHL
QSPTHLIVQSLAACDCLLGSLVMPYSMVRSIEGCWYLGDVICKVHSSLDMTLSISSKMHLSLISV
DRYWAICDPLMYRTRVTNYSITVFIMCAWLFAFVYSFSIVFSEVNLIGLEVLVLQISCLGSCVLL
FNKPSALICSILTFFLPGAIMSSLYVKIFRVASKHAKVLSERVSVLEIKSQTYAQRERKAAKIVA
IVVGAFLLCWLPFFVATALDPFLDFWTPADVFDALVWFGYFNSTCNPLIYGFFYPRFQKAFKILM
STVMCGSSSSRTLAFG

>Dr-taar13a

MDLSSQEYDPSQFCFPAVNNSCLKGTHHVSTQTVVYLILASAMTVTILGNSVVIISIAHFKQLQT
PTNILVMSLALADLLLGLVVMPFSMIRSVDGCWYYGETFCMLHSTFDLFLTSVSILHLVCIAVDR
HQAVCYPLQYPTRITISVAWVMVMISWTLIAIYSYGLVYSKANVEGLEEYIESIYCMGHCSLLFS
KLWSVLDTFITFFFPSCIMVGLYIRIFVVANKHARVITEANPNENENVFKSSRRSERKAAKTLGI
VVGAFIICWLPFFINSLMDPYINFSTPVALFDAFVWLGYINSTINPIIYGFFYPWFRKTLYLIIT
RRIFEPNSSDINVFTV

>Dr-taar13b

MDLSSQEYDPSQFCFPAVNNSCLKGTHHVSTQTVVYLVLASAMTVTVLGNSVVIISIAHFKQLQT
PTNILVMSLALADLLLGLVVMPFSMIRSVDGCWYYGETFCLLHSTFDLFLTSVSILHLVCIAVDR
HQAVCYPLQYPTRITISVAWVMVMISWAVIAIFSYSLMYSKANMEGLEEYIESIYCMGHCSLLMN
KLWSVLDTFITFFFPSFVMVGLYIRIFVVAKKHARVITEANPNENENLFKSSRRSERKAAKTLGI
VVGAFIMCWLPFFINSTLDAYINFSTPVALFDALVWLGYVNSTINPIIYGFFYPWFRKTLSLIVS
LKIFEPNSSDINVFTV

>Dr-taar13c

MDLSSQEYDPSQFCFPAVNNSCLKGTHHVSTQTVVYLILASAMTVTVLGNSVVIISIAHFKQLQT
PTNILVMSLALADLLLGLVVMPFSMIRSVDGCWYYGETFCLLHTGFDLFLTSVSIFHLIFIAVDR
HQAVCFPLQYPTRITIPVAWVMVMISWSMAAFYSYGVVYSKANLEGLEEYIASVYCMGGCTLYFN
ALWSVLDTLLTFFLPCSVMVGLYARIFVVAKKHIKSITEANQNENENVFKNPRRSERKAAKTLGI
VVGAFILCWLPFFINSLVDPYINFSTPYALFDAFGWLGYTNSTLNPIIYGLFYPWFRKTLSLIVT
LRIFEPNSSDINLFTV

>Dr-taar13d

MDLSSQEYDPSQFCFPAVNNSCLKGTHHVSTQTVVYLILASAMTVTILGNSVVIISIAHFKQLQT
TTNILVMSLALADLLLGLVVMPFSMIRSVDGCWYYGETFCMLHSSFDLFLTSVSIFHLIFIAVDR
HQAVCFPLQYPTMITIPVAWVMVMISWSMAALYSYGLVYSKANMEGLDEYIQSMYCVGGCTLYFN

ALWSVLDTLITFFLPCSVMIGLYARIFVVAKKHARIINEANQNEIDGTFKSSRRSEQKAAKTLGI
VVGAFIMCWLPFFINSLMDPYINFSTPVALFEAFVWLGYINSTMNPIIYGFFYPWFRKTLYLIIT
RRIFEPNSSDINIFTL

>Dr-taar13e

MDLSAQEYDASQFCFPAVNNSCLKGTHHVSTQTVVYLVLASAMTVTILGNSVVIISIAHFKQLQT
PTNILVMSLALADLLLGLVVMPFSMIRSVDGCWYYGETFCLLHSSFDMFLTSVSIFHLIFIAVDR
HQAVCFPLQYPTMITIPVAWVMVIISWSMAAFYSYGLVYSKANVEGLEEYIESIYCMGGCTLLFN
ALWGAIDTLVAFFLPCFVMIGLYARIFMIAKKHARKLGEANQHDNENLFKSSRRSERKAAKTLGI
VVGAFVICWLPFFINSMMDPYINFSTPGVLFEAFVWLGYMNSAINPIIYGLFYPWFRKTLYLIIT
LRMFEPNSSDINVFTV

>Dr-taar14f

MNLTAVNQTDMCEDYSCPERSVSLSVYVILYVAAAAVALLTVCGNLLVIISVSHFKQLHTPANIL
ILSLAASDLLVGVFVIPLHLSWVIESCWISGPVVCLVFKLVNYQATSVSVHTVALIAVDRFLALS
FPFFYSEKISLTVVCTAALLNWLFSLIYNFTLFYVNENFTVVVCPGVCFHHLDGISSLIDLLIVF
VMPCTLIIILYTHVFVIAKKHATAIRALQVHNSTESSKNKISDKSERKAAMLLGILVFVFLLCLL
PYYITFLVIPYGSIIYMYVINVAVIFFFLNSTINPIIYALFYSWFKKSLKLIFTFKVFHKDSSLM
NVM

>Dr-taar15a

MEFQEHHCLSHWNTSCSLDFHLSYVNVFLFLYISAISVLTVCGNLLVIVSIAVFKQLHTPTNLLI
LSLAISDLLVGICLMPVESIRSINSCFYMGKSHCHIFHSIMSIVGSASLMNIMLVAIDRYFAVCN
PLLYMSKMTIRKALICVGLGWTVSICYNLVPVNLGNSDPTGAVVVCLRECAVAFSNSWGPADLIV
TFIAPCITLLILYIRILTVALKQAKAISNMTKKNGSQPSRKSEVKATKTLSTIVFVYLICWIPWY
ASMLNMEQFNKSPVSLTALLCLFYTNSCINPFIYAISYPWFKRSVKLVVSLRILQTATNQLNLFA
EDC

>Dr-taar16a

MKDKNLEIQFCFPNNNLSCFKEIKPQTDYVVLYIFIFLASVVTVFLNLLVIISISHFRQLHTPTN
LLILSLAVADLIVGLIVIPFMGIRFIDSCWYYGETFCSVFLFITFAVVSGSLGNLVLISLDRYIA
VSDPLRYTVKITTDRIVICIVVNWLCSSIYSFIFLYNSVFYPETQNGCYGDCRVSFKFEHFVTDL
LVTFVAPSCVIMSIYVKIFCVAKHQAKVVNSVTGVSKSQRKAGKTLGIVVMVFFMCWIPYYIVIL
IEGNELTESVEFNVTCWIVYMNSCMNPLIYSLFYKWFRISAKHILTLNILKLSSEYFNLFPEDK

>Dr-taar17a

NKLLTGAESLMAYETEETQYCYPAFNSSCVKGKRSNYESNILYVFFSLLSAWTVFLNLLVIFSIS
HFKKLHTPTNMIILSLSVADLLVGLIVMPIEATELIETCWYFGDTLCNLFLILMGLLTSTSLCSL
VLIAVDRYVAVCHPLMYPQTITMNRTLIIVCFSWFFSSAYNIILSITHTSDGCYGECNVQLTFEW
KATDLVLSFLLPCTVIITLHLRIFYVVHQQVKVINSQMKSEKCVMEGSVKRKSESKAALILGIIV
AIHLICFITYYILTLTDSMAIPPTVLSCLICFLYINSSLDPLLYALFYTWFRKSIKHILTLKIFQ
PESSQVNILTNH

>Dr-taar18a

MKGQKGEQNKLLTGGDSLMAYETEDQETQYCFPDINSSCVKENRSKHEYNIMYVFFSLLSAWTVF
LNLLVIISISHFKKLHTPTNMIILSLAVNDLLLGLIVMPVDAVKLIETCWYFGDILCDLYMIIMG
LLLSASLSNLILIAVDRYVAICHPLLYPQKITMTRTLISIFVCWFVCLTNNIAFLVSSRHFDILQ
KTNMCHGQCTLIVSFSWTFTDLFLSFLVPCTLIITLYLRIFYVAYQQVKVINAIIKGGKCAVEGS
VKRKSERKAALTLGIVVTVYLLCYIPYYILSVIGTTVISSTMKILIWSLYINSCLNPLIYALFYR
WFKISVKCVLTLKILEPASSLLDIFKDNS

>Dr-taar19a

NKLLTGGDLLMAYETEDQETQYCFPDINSSCVKGRHSSHGYIIIYLFVSLLSVWTVFLNLLVIIS
ISHFKKLHTPTNMIILSLAVNDLIIGLFVIPIQAIKLIETCWYFGDTFCGLYLFLIFELLSASLS
NLVLIAVDRYVAVCHPLLYPQKITITKTLMSICLSWVCYSAYNTALIINNRYSETSHRTDECYGR
CFIVMSFSWLVADLVMSFIFPCLIMITLYLMIFYVVHQQVKVMNSLMKGGKCVTEGSVKRKSESK
AALTLGIIVTIYLLCYIPYYICSLVVNSSTAIHVMSWTVYVNSGMNPLVYALFYPWFKKTAKIIF
TLKIFQPASSLINIFTE

>Dr-tar20a

MKGQKGEQNKLLTGGDSLMAYETEDQETQYCFPDINSSCVKEQHSSHGYIIIHLFVSLLSAWTVF
LNLLVIISISHFKKLHTPTNMIILSLAVNDLFIGIAMPVEAIRLIETCWYFGDTFCVLYLFFVAL
LLSPSLGNLFLIAVDRYVAVCHPLLYTQKTTIAKIFCLSICLSCVCFSAYFTALVNVNNQDFDTS
QRTDVCYGQCLVMITFSWIVIDLFVSFIFPCTLIMMFYLRIFYVVHQQVKVINSLMKGGKCVTEG
SVKRKSESKAALTLGIIVSVYLLCYIPYYICSLTVNSSTAINVLVWLVYSNSGMNPLVYAIFYPW
FKKTAKLILTLKIFQPASSLINIFTEL

>Ga-taar21a

MEPEETDNGFNAVSDLHPCYESDNGTYSFTSNFSISCVSLYVFLGGLSVVTICGNLLVIISVSYF
KQLHVATNFLILSLAVADLLVGVVVFPFSMVFTVTSCWHHEGLFCKVRGGFDVTLSTASILHLCC
ISIDRYYAVCQPLSYKSKVNDRVTGMMILMSWAVPLLIGICIIIAGFNQGKCEESCLIDALISTT
LACIFSFYIPVIIMLSIYLKIFLVAKRQAISIQITTGLTKKSGTTVSKRERKATKTLAIVLGVFL
LCWAPYFLCMIFQPITYNVTPIAVIETLNWLTLSNSMLNPFIYAFFYSWFRSAFKMIISGKVFWG
NLENSKLL

>Ga-taar22a

MDGMEGAQLCFPQLLNSSCRGLLRPRTESVLLYTLLSSMAVLTVLLNLLVIISISHFAQLHTPTN
LLLLSLAISDLLVGLLVMPSEMVSVIETCWLLGDLLCTLSTMMGSTLVSASVGNMVLISIDRYVA
ICYPLQYPIQITRSRISVLYNGLILKDRLRQPDRDNSCDGECKVVINNVSGAIDLVFTFFAPCSV
IVVLYVRVFTVAVSQARVGSHFTAVGAVKITAKKSERKAARTLGMVILVFLISFCPFYYPALAGQ
EISNNASSWVIVSWLLFFNSCLNPLIYALFYPWFRKAVKLIVTLKILEQNSSQENII

>Ga-taar23

MAEHASQLCFPQLLNVSCRKPSVLWNDAVLSLALMSVSALTVALNLVVIISVSLFRHLHTPTNIL
IVSLAVSDLFVGLLVMPVEILFRVSCWFLGDLVCALFNYVFFIITSVSVGTMVLISLDRYLSICE
PLHYATRVTVKRVRCSVCTCWLCSVCYSGVLASDGVTPSGRYSSCHGECLIVIDYVTVTVDLVVT
FGVPVSVIVVLYMRVFAVAASQARSLRSHVLVVTHQLPVGQRAKKSELKAARTLGVLVVLFLICF
CPYYIVSLVGYSQLSNLYMSFVLYLFYLNSCLNPLIYALFYPWFRKAVKRIVTLQTGS

>Ga-taar24

METLPGDYLCFPQLNSSCKKPAHPHTEAMLVYVLLSSISLITVVLNVLVIISISHFRQLHSPTNL
LILSLAVSDLLVGLLLMPVEIIYIEACWFLGDILCTTYYLVDYVITSASVANMVLISVDRYIAIC
EPLHYPTKVTKRRVQNCVCFCWIWSAIFRIFLLYDHLEKPGSSNSCLGECVVIINYAAGVADLVF
TFIIPILCIIVLYLRVFVVALSQARAMRSRVAASTTRRSGATTVKKTEMKAARTLGLVILVFLFC
FCPYYYPALAGKDTSIDASSAAFEIWLAHFNSCLNPVIYAFFYPWFRKSIQLILTLQILKPGSCD
ANVL

>Ga-taar25a

MKSLEEDELCFPHLLNSSCRKAMHPHFVSMLTYILLSSISLLTVTLNLLVIISISHFRQLHTPTN
LLLLSLAVSDFFVGFIVFFQIVVIEGCWFLGDLMCILYIFLDYIITSASVGTMVLISVDRYVAIC
EPLHYSNKVTQKRVKISVCLCWTCSAFLQTLLLKDNLEEPGRYNSCFGECVVVINYIAGLADLFF
TFIGPVTVIIVLYMRVFVVAVTQARVMRSHVAVLPHKFSMNITAKKSEMKAARTLGVVIVVFLLC
VCPYYCVLLSAQDTLFNISSAAFVTCVFYFNSCLNPLIYAFFYPWFTKSMKLIVTLEILQPGSCE
RNLL

>Ga-taar26a

METLVGTELCFPQLINTSCRKPIRPHLEAMLIYILLSSISLLTLALNLLVIISISHFKQLHTPTN
LILLSLAVSDFFVGLFVIFQIMIIEGCWLLGDLMCSLWLILSSIIMSSSIGTMVLISVDRYVAIC
YPLHYFTKVKPKRIRVCVCLCWMFAALFNSLLLKNNLQHPGRYNSCIGECVIEMNYIANVFDLIL
SFFFPITVIVILYIRIFGVVVYQARAMRPHIAVVTMKVAVKKSEMKAARNLGVVVVVFLICVCPY
YCFTLTSQNNLYTSLSVTILVWLFQFNSCLNPLIYATLYPWFRKSIKVIVTLQILKPGSYRTNML

>Ga-taar27

MEPVLCYESRNGSCLRTIYSLPIQITVYMILGVIVVLTVFGNLLVTVSIAYFKQLHTPTNYLISS
LAVCDLLLGLLVMFPSMVQCVESCWYFGDVFCKVYMSSDVMLCTTSILNLLFISIDRHYAICQPL
RYREKISVNVVLIMILLSWTISGLIGFGMIFLKLNFWGIEEFYSNHIVCEGECILFQTGLSSIMS
SVLSFYIPGIILLSVYLKIFLVARRQYSIQIASRTCSEGISNTSQKKATKTLAIIMGAFLSFWTP
FFVCNIVDPFISYSTPPALFKTLVWLGYFNSTVNPLIYAFFYSWFRKALKLFTSGIMFKADISET
TLFTL

>Tr-taar28a

MIYVFRHFQTTTNLLLLSMAVSDLLVGLAVMPLMIVTLDSCRCISSYLCFLFHLLSFVLTSASVG
NMVLISVDRYVAICYPLRYSSITPNTVKICVSVCWTSSIIYNLVLLKDNILYIDFFSSCNKKCPL
YINYILAIADIIITFYGPLTVIIVLYTRVFVVAVTQARAMRSQVSTTGSKAVSAMKSEMRAARTL
GIVILFFLICFFPYYISSLTGQGISNEDSTGQLFLFFSNSTINPIIYAFFYPWFRKTVKLFLSCQ
FCKL

Aminergic receptors used as outgroup (species names, gene IDs: Suppl. Table 1)

Adrenergic receptors

>Bbe-adra1a

MAGLPLELDNFTLDDNNGTWNVTEADHPSQILGLRMSLPASLLVGAVLSCICILTIAGNVLVVLA
VCLERSLRTNIGYFYINLAAADLLLGCLVLPFSATRELLNYWPFGEALCELWAAVDVLCCTASIF
SLCVISLDRYIGVTRPLHHNMIVTKRRVVLILFAVWATSAAISVGPLFGWKHPKSYQNMQCNINS
SPSYIIFSVVGSFYVPAFVVLYVYRQVYRAAVRETSKLMITLKAQEDRNQTPVESVRIHRGKASV
TAEKRLSPPRLSPNSSKPRMAREALSSVKNRIEKFTKEKKAAKTVGIIVGVFLVCWLPFFVMYPI
DSMIAKVPELLITMAFWLGYCNSFLNPIIYACSNGHFRRAFRRFLLCGKRQGRFPTMRTEDYTLS
SRSSFRRVHSSCSSRDGSRRQQLKIQLQELTVS

>Cm-adra1a

RAHKVIWLVLGSFILFALLGNILVILSVASHRHLHTATHYFIVNLAIADLLLSSTVLPFSALLEI
LGRWVFGRLFCNIWAAMDVLCSTASIMSLCVISIDRYIGVSYPLRHPAIMTEKRGVLTLVAVWLL
ALVISVGPLFGWKEPEPEDESVCKITEVPGYVLFSAFGSFYVPLAIILLMYCQVYVVAKRETRSL
MAGEKREGSDTNKVTLRIHRKNVGGDVSPRSGEHHSRGKGHHLKTHFSVQLLKFTRQKKAAKTLG
IVVGGFILCWLPFFVVLPLGSCFPAYRPPETVFKVTFWLGYFNSCLNPVIYRCFSQEFKRAFRNV
LRGECHRRKPPVNQGSSLSSSSAHSGAERPKEVVRTQVGTNEAFLGIARPQAPSQWSLLPARPDH
RTARSPRQPPRALLGSCCGGVVVVELQ

>Lec-adra1a

MFVTHENSTSHLNGTSEGISVSNETSPTVEPRRAVTLALVLGAFIIFAIAGNILVVLSVVCNKHL
RTVTNYFIINLASADLLLSTTVLPFSATFEILGYWVFGRIFCDIWAALDVLCCTASIMSLCAISI
DRYIGVSYPLRYPTIMTERRVLLILIAVWVLAIVISVGPLLGWKEPAPPDETVCEITEVPGYALF
SAMGSFYIPLVILLAMYCRVYVAARRQTKSLEEGIKRERSSSSEEVTLRIHRRTIAPGEYSLRDT
SKGPHFRSSLSVRFMKFSREKKAAKTLGIVVGVFILCWLPFFIALPIGSFFPSVRPSQTVFKVVF
WLGYFNSCINPLIYPCSSQEFKRAFLRALRCHCREGQRRRRQPWGLHHASSTGSSYSGGRKGSSH
LSQLHEVHGGCSPDGLCERSRSPDEPSPVSQDSLRGWRLLSTSKALSGTALILERANSPQPET*

>Mm-adra1a _

MVLLSENASEGSNCTHPPAQVNISKAILLGVILGGLIIFGVLGNILVILSVACHRHLHSVTHYYI
VNLAVADLLLTSTVLPFSAIFEILGYWAFGRVFCNIWAAVDVLCCTASIMGLCIISIDRYIGVSY
PLRYPTIVTQRRGVRALLCVWALSLVISIGPLFGWRQQAPEDETICQINEEPGYVLFSALGSFYV
PLTIILVMYCRVYVVAKRESRGLKSGLKTDKSDSEQVTLRIHRKNVPAEGSGVSSAKNKTHFSVR
LLKFSREKKAAKTLGIVVGCFVLCWLPFFLVMPIGSFFPNFKPPETVFKIVFWLGYLNSCINPII
YPCSSQEFKKAFQNVLRIQCLRRRQSSKHALGYTLHPPSQAVEGQHRGMVRIPVGSGETFYKISK
TDGVREWKFFSSMPQGSARITMPKDQSACTTARGFYP

>Mm-adra1b

MNPDLDTGHNTSAPAHWGELKDANFTGPNQTSSNSTLPQLDVTRAISVGLVLGAFILFAIVGNIL
VILSVACNRHLRTPTNYFIVNLAIADLLLSFTVLPFSATLEVLGYWVLGRIFCDIWAAVDVLCCT

ASILSLCAISIDRYIGVRYSLQYPTLVTRRKAILALLSVWVLSTVISIGPLLGWKEPAPNDDKEC
GVTEEPFYALFSSLGSFYIPLAVILVMYCRVYIVAKRTTKNLEAGVMKEMSNSKELTLRIHSKNF
HEDTLSSTKAKGHNPRSSIAVKLFKFSREKKAAKTLGIVVGMFILCWLPFFIALPLGSLFSTLKP
PDAVFKVVFWLGYFNSCLNPIIYPCSSKEFKRAFMRILGCQCRGGRRRRRRRRLGGCAYTYRPWT
RGGSLERSQSRKDSLDDSGSCMSGSQRTLPSASPSPGYLGRGTQPPVELCAFPEWKPGALLSLPE
PPGRRGRLDSGPLFTFKLLGEPESPGTEGDASNGGCDTTTDLANGQPGFKSNMPLAPGHF

>Le-adra1b

MTFAEFTNESLDGNCSNASALSYAVFPNDSVTANRSGNSEGLDVNQAVALGVILGAFILFAIVGN
IMVILSVACNRHLQTVTNYFIINLAIADLLLSTIILPFSASLEIFGYWAFGRIFCDVWAAVDVLC
CTASIMSLCVISIDRYIGVRYSLRYPTIMTEVKAVWILIVLWVTSIVVSVGPLLGWKEPAPADDT
MCSITEEPGYALFSSLFSFYLPLMVILVMYFRIYVVARRTTKSLEAGVKRERSKTMEVVLRIHCR
SVLDDSHSNFKNKGHHLRSSLSMRLLKFSREKKAAQTLGIVVGVFILCWLPFFTILPLGR*

>Bfl-adra2

QAMKGVQNLFLVSLACSDVTVGTLVMPFKLANELMGYWYFGQTWCDIYLALDVFACTASIFNLCA
ISIDRYWAITKPIKYLSMRTRRRVGIMIAIVWMSSALVCLPPLFGWKSGDDSGDEQEASETPGCQ
LNSSPGYVVFSSVSSFYIPLGVLIFVYTSIIRAVRKRFKFRNKNGEWKQICNVRLKDSYDVPGYV
RSRGAISSPGKKSRGHSQCGDTLQRPEKELQVLEALQPLKSPGFSPRGPYFDGKMVVLPTKTTPI
VDNVRKVREKLRAAQQKERKSLSIIFCVVSVFLICWLPFFVCYLVVTLCESCWVPPPLFMFVEWM
GYGNSALNPVIYALFNKEFRDGVKMLFRRNNYSIPLANT

>Cin-adra2

SSILYSEEQVVIFSALTSILCIIGLFGNLTVIVVIRRDRVIYKHRQNLYLLSLAVADVSLVVLVV
PFSITNELLGYWPFGTVYCRIYLSVDILLCTASIWNICMIGLDRYISVKYPMTYRKFRTMPKIRL
FIVSIWLFAAVVSLLPFVSEIETINSERGCFINASSWYIVVSCSLSFFIPSCIIWPVYIRIYMIG
NHIQMNMLIRRSDPKQDSTSKFETKVRRLSRTSRSLENLMTLLVVPNSTNNSRSVTSATTDQRQM
NRSQSFTECLNSGREDGNLLSSVAYDVTSQKSTRSDIYYISRSSSQSQQSSCTTAMSRRERRFVC
IISIITGCFMACWMPFFLTYMIYAVCRACCINNTLFKVFFWLGYLNSALNPVLYTAFNKDFRSAF
QRLFHLSGRRTIC

>Lec-adra2

MQNMSDPAAARSTLLSPYAAFNFTLAEPENVTGGTNSLNGTDEKLGWEYSVLAGLGLSSLVLILV
FATVFGNCLVIIAVLTRPSLKAPQNLFLVSLATADILVATLVIPFSLFNELMGYWYFGRVWCEIY
LALDVLFCTSSIVHLCAISLDRYWSVSQAVEYNIKRTPRRMKRIIVIVWFIAIAISLPPLISMNQ
KNRQQQEELGRPNCELNTNTWYILGSCIGSFFAPCLIMVLVYARIYHLVKWRAAKLRRDKEAPIT
SCRPMESIRMSEVNGNGGGGGGGGEGNGNGEKNCNGAGNKSRHHHHPPHHQHPPSHHHPEVDVEE
TSVSSDKNERRRDDASAGAKASRKPCSPTKDDYNPEMEILERQNKARERRKIAQARERRFTFVLS
VMIGVFVICWFPFFFTYSLSALCSPEFCVIPETLFKFFFWFGYCNSCLNPVIYTVFNRDFRRAFK
KILCMQFNTSF*

>Mm-adra2a

MFRQEQPLAEGSFAPMGSLQPDAGNSSWNGTEAPGGGTRATPYSLQVTLTLVCLAGLLMLFTVFG
NVLVIIAVFTSRALKAPQNLFLVSLASADILVATLVIPFSLANEVMGYWYFGKVWCEIYLALDVL
FCTSSIVHLCAISLDRYWSITQAIEYNLKRTPRRIKAIIVTVWVISAVISFPPLISIEKKGAGGG

QQPAEPSCKINDQKWYVISSSIGSFFAPCLIMILVYVRIYQIAKRRTRVPPSRRGPDACSAPPGG
ADRRPNGLGPERGAGPTGAEAEPLPTQLNGAPGEPAPAGPRDGDALDLEESSSSEHAERPPGPRR
PDRGPRAKGKTRASQVKPGDSLPRRGPGAAGPGASGSGHGEERGGGAKASRWRGRQNREKRFTFV
LAVVIGVFVVCWFPFFFTYTLIAVGCPVPSQLFNFFFWFGYCNSSLNPVIYTIFNHDFRRAFKKI
LCRGDRKRIV

>Rt-adra2a

NKDLGGDNMLVENIGGGIAVCKDSRARAGHGGAGSRARGLSVRGRGRGRLRERLGAPRLLMALTI
FGNVLVIIAVFTSRALRAPQNLFLVSLATADILVATLVIPFSLAKELMGYWYFGRLWCQVHLAID
VLFCTASIAHLCAISLDRYWSISQAIEYNLKRTPRRIKGIIVIVWVIAAAISCPPLITNISTNQS
QEPGTCHINDQVWYIISSCIGSFFAPCIIMILVYIRIYQIAKLRSRGRPGDIPNGLNQPPSPPGA
PEPGQHNGGDEQGVLAEVEDSSSSEQPPCPALAPSPSPAAHRQHCHPPQPQHTAPNPAKATGQVR
CVDGRPVTSLAPRSRWKGRQNREKRFTFVLAVVIGVFVLCWFPFFFTYSLTAICPKLCPVPEKLF
KFFFWMGYCNSSVNPLIYTVFNQDFRKAFKKILCRGQRRGQKL

>Mm-adrB1

MGAGALALGASEPCNLSSAAPLPDGAATAARLLVLASPPASLLPPASEGSAPLSQQWTAGMGLLL
ALIVLLIVVGNVLVIVAIAKTPRLQTLTNLFIMSLASADLVMGLLVVPFGATIVVWGRWEYGSFF
CELWTSVDVLCVTASIETLCVIALDRYLAITSPFRYQSLLTRARARALVCTVWAISALVSFLPIL
MHWWRAESDEARRCYNDPKCCDFVTNRAYAIASSVVSFYVPLCIMAFVYLRVFREAQKQVKKIDS
CERRFLGGPARPPSPEPSPSPGPPRPADSLANGRSSKRRPSRLVALREQKALKTLGIIMGVFTLC
WLPFFLANVVKAFHRDLVPDRLFVFFNWLGYANSAFNPIIYCRSPDFRKAFQRLLCCARRAACRR
RAAHGDRPRASGCLARAGPPPSPGAPSDDDDDDAGTTPPARLLEPWTGCNGGTTTVDSDSSLDEP
GRQGFSSESKV

>Rat-adrB

MEPHGNDSDFLLAPNGSRAPGHDITQERDEAWVVGMAILMSVIVLAIVFGNVLVITAIAKFERLQ
TVTNYFITSLACADLVMGLAVVPFGASHILMKMWNFGNFWCEFWTSIDVLCVTASIETLCVIAVD
RYVAITSPFKYQSLLTKNKARVVILMVWIVSGLTSFLPIQMHWYRATHKQAIDCYAKETCCDFFT
NQAYAIASSIVSFYVPLVVMVFVYSRVFQVAKRQLQKIDKSEGRFHAQNLSQVEQDGRSGHGLRR
SSKFCLKEHKALKTLGIIMGTFTLCWLPFFIVNIVHVIRANLIPKEVYILLNWLGYVNSAFNPLI
YCRSPDFRIAFQELLCLRRSSSKTYGNGYSSNSNGRTDYTGEQSAYQLGQEKENELLCEEAPGME
GFVNCQGTVPSLSIDSQGRNCNTNDSPL

>Alu-adrb

MSANTTVSPTQTTAILTTNSTDSPVGSCFAPNPYSAGVQAVLGFITVILILLTVVGNLLVILAVT
CHRKLRTVTNFFIISLACADLSVGITVLPFAATNDILGYWPFGGYCDVWVSFDVLNSTASILNLC
VIAFDRFLAITAPFTYHTRMTERTAGILIASVWGIAVVVSFLPVQAGWYRDNQSTEALAIYADPC
LCIFAASTAYTIVSSLISFYIPLVIMLVFYGYVFKAARDQARKINALEGRIEQENNRHGKKISLA
KEKKAAKTLGIIMGVFILCWLPFFVVNIVNPFCNRCVPPAGFIALTWLGWINSCFNPIIYAFNKE
FRKVFVKMICCYKCRGVTVGPNMADLTYDPVAMRLKKKGDNLNSSANGKANGNLDQVAGEGTSDS
*

>Cin-adrb

GLSILLGSICTVTVLGNLIVIFTAILFRQLRTIPNMFIISLASADLLMGTIVLPMWSHYVIGDFK
WSLGFFWCDVWTSIDVLSVTASIGTLCAISLDRFVAITMPFKYATKMTRPRARFIIGFIWMVSGT
IAFVPINLGWWKTNKPEDLECYDSPTCCEFRPNVIYAVVSSCISFYIPLIIMICAYSVVFKSKAI
FTHFTIVISTTVAIQKRGVLPWEKREHRAVITMGIIMGTFVVCWLPFFIVNIAQVFCQCVASTPF
LMVNCLGYTNSLFNPIIYCRSEEFRKAFKRVLMCGLC

>Cm-adrb1

MGDGMDCNSSSSSHHHHHNNNNNRSATLVSANSANGVSPNGPEQSSSEHQWVIVMGVIMAVIVLI
IVVGNLLVVLAIARNQRLQTLTNLFIVSLACADLVMGLLVVPFGATLVVRGTWLYGSFFCEFWTS
VDVLCVTASIETLCVIAIDRYIAITSPFRYQHLLTKTRARGIVCVVWAISALISFLPIMMHWWRD
ENPDAIVCYNDPCCCDFLTNKAYALASSIISFYLPLLIMIFVYARVFQEAKKQMQKIIKCEGRFY
SSYFIPNGRSARRRPSKTIAIKDQKALKTLGIIMGTFTLCWLPFFLVNVAKAFRSEIVDDKLFLF
LNWLGYVNSAFNPIIYCRSPDFRKAFKRLLFCPRRPGSRLDLDSGGFSRCSGMFGNTLDPRNLEM
WSDWNMALQSSSEGQGVGMGQGMGQGMGAGDGAGDGAGDGDGEGDGEGDGEGEGDSPGIAVINSA
FFFSLQRSKRD

>Csa-adrb

MFAFKKKNLKQQLNGLAEHSTGYRKLNGRRNRKLWFYFFGLIVLIHLFAIPVHASDTTQSFTPTP
NLTSNKFTDLRHSGITIPEYENQGLNAPLKAGLYFILSCVCIFTVIGNVMVILTATIFRKLRTIP
NMFIISLASADLTIGTVVLPMWSHYVIGGKWDLGAFWCDIWTSVDVLSVTASIGTLCAISLDRFV
AITMPFKYSTKMTRLRARFIIGFIWIVSAAIAFIPINLGLWKTNEPDDQKFYDDVTNCEFKPNTV
YAVVSSCISFYIPLIIMVCAYSIVFKIAIQKGSKIRNREGVYRRASMPGSRTLLWGKREYRAVFT
MGIIMGTFVICWLPFFIANIVQVFCEGCISAPAFMMINCLGYINSFFNPIIYCHSLEFRRAFKRL
LLCGLCRDGRYKANKFYSFLFNTLSLKMTV*

>Lec-adrb

MLNFQVIPHEDATPPCVVGNSSAWNDSDPATSVGVNCSSEGTGQKPAEMEWMVGLGMLMSLIVLF
IVFGNVLVITAVAKFRRLQTQTNYFVVSLACADLLMGLIVVPFGATPVVSGTWTWYFGPRFCSFW
TAVDIMCVTASIDTLCVIAVDRYIAVARPLRYETLMNKRRARFIIVAVWLVSALISFLPIEMGWW
KSEAAAAANNTAEDVCEFNISLEYAVASSSVSFYLPLTVMIILYSKVFREARKQMEKINTSEGRF
YNNANDSATIKNPARKLSRFLSRKEHRALKTLGLIMGTFTLCWLPFFVVNIVQVVWNCVPTGVFV
SLNWLGYINSAFNPIIYCRSPDFRSAFERLLCCGRLVGRRRRRRGRGRKDNNELGQCTSWPICPD
SGSTQGYVERCSETGTTLCGASTSSQGSSNSEQSLDTNGNSHGLLSAL*

>Rt-adrb-like

MGDRLSSEDCHTNKSSTILFVNPANGVSPHVPQQEFSEQWVIGMGTVMASIVLIIVLGNLLVVFA
IARNQRLQTLTNVFIVSLACADLIMGLLVVPFGATLVVRGNWLYGSVFCEIWTSVDVLCVTASIE
TLCVIAIDRYVAITSPFRYQSLLTKARARAIVCVVWLISGLISLLPIMMHWWRDDQPDAIRCYND
PCCCDFVTNKAYALASSIISFYLPLLIMIFVYARVFQEAKKQMKKIDKCEGRFYTSHFIPNGRSA
RRRPSKIIAIKDHKALKTLGIIMGTFTLCWLPFFLANVAKAFRTDIVPGKLFLFFNWLGYVNSAF
NPIIYCKSPDFRAAFKRLLFCPGQADSNRLCSGSGEFSRCSGMFGNTLDPRSLETWSEWNRSQEA
NGGGLDRHMGENPEDQESQF

Dopaminergic receptors

>Pm-drd1-like

MLMVVVAMMEEKGFKAEPACAAGNEDEDDTRRVMLNLTADEALGCEPGKGLSFRILAGCLLSLLI
LSTLFGNTLVCAAVIKFRHLRSKVTNYFVISLAVSDLFVALLVMPWKAITEVAGFWPFGAFCNVW
VAFDIMCSTASILNLCVISVDRYWAISSPFRYERKMTQRVAFVMISVAWTLSVLISFIPVQLNWH
KAQHVQQTWEFHNASGGADADASVPENCDSSLNRTYAISSSLISFYIPVAIMIVTYTKIYRIAQR
QIRRIASLERAAEHAHNCQLNPNDCPNEMSFKTSFKKETKVLKTLSVIMGVFVFCWLPFFILNCV
VPFCDSAGVEGDDEQQQQQHHPLCVSETTFSIFVWFGWANSSLNPIIYAFNADFRKAFSTLLGCN
TLCASTPVETVNLSNEVASYPHDVTSNHKDGGANYVQFIPHVVIVNDESDGDFDKVPEESRSMRE
DRSGIDHRLDCDEDVSLETITPFTPNGVHET

>Mm-drd1a

MAPNTSTMDETGLPVERDFSFRILTACFLSLLILSTLLGNTLVCAAVIRFRHLRSKVTNFFVISL
AVSDLLVAVLVMPWKAVAEIAGFWPFGSFCNIWVAFDIMCSTASILNLCVISVDRYWAISSPFQY
ERKMTPKAAFILISVAWTLSVLISFIPVQLSWHKAKPTWPLDGNFTSLEDAEDDNCDTRLSRTYA
ISSSLISFYIPVAIMIVTYTSIYRIAQKQIRRISALERAAVHAKNCQTTTGNGNPVECSQSESSF
KMSFKRETKVLKTLSVIMGVFVCCWLPFFISNCMVPFCGSEETQPFCIDSITFDVFVWFGWANSS
LNPIIYAFNADFQKAFSTLLGCYRLCPTTNNAIETVSINNNGAVMFSSHHEPRGSISKDCNLVYL
IPHAVGSSEDLKREEAGGIPKPLEKLSPALSVILDYDTDVSLEKIQPVTHSGQHST

>Cm-drd1

MDGRSVTTKDKDSSFRILTACFLSLLILSTLLGNTLVCVAVVRFRHLRSKVTNFFVISLAVSDLL
VAVLVMPWKAVSGVAGYWPFGSFCTIWVAFDIMCSTASILNLCVISVDRYWAISSPFRYERKMTP
KVAFIMIGVAWTLSVLISFIPVQLDWHKAKATSTLLDYNVSYQGQGRVDNCDSSLNRTYAISSSL
ISFYIPVAIMLVTYTRIYRIAQKQIRRISALERAAVHAKNCQTSCESGSLDGQQQQQQQSESSF
KMSFKRETKVLKTLSVIMGVFVCCWLPFFILNCIVPFCEHPGRVSTKDQLPCISDTTFDIFVWFG
WANSSLNPIIYAFNADFRKAFSTLLGCHRLCSGNAVETVTIHNNGVTGCNPEPSGQQQQQVLGNC
VYLIPHPVSCEAGNAPDPHHCCPRKLSPASLHGAVSLQSGYNTDCEADVSLEKITPITQNGQHNT

>Rt-drd1

MRLNTTTMDGSSLAAEQDSSFRVLTGCFLSLLILSTLLGNTLVCVAVIRFRHLRTKVTNFFVISL
AVSDLLVAVLVMPWKAVSEIAGFWPFGPFCNVWVAFDIMCSTASILNLCVISVDRYWAISSPFRY
ERKMTPKVAFVMISAAWSLSLLISFIPVQLDWHKARAPNGLHGNASSQRLGDNCDSSLNRTYAIS
SSLISFYIPVAIMIVTYTRIYRIAQKQIRRISALERAAVHAKNCQSNRSSASTTDIQSESSFKLS
FKRETKVLKTLSVIMGVFVCCWLPFFILNCMVPFCESHASPPPLPCVSGSTFDVFVWFGWANSSL
NPIIYAFNADFRKAFSTLLGCDTLCASNAVETVTIHNGVSSYHPGPSLDKAENSVYLIPHSVPCP
PETPTDPNHCAKLSPISQHGAVSLLSSNGDYETDVSLEKIIPITQNGQHNT*

>Alu-drd1,5

MAAANNSTNSTDMVQVWDDLDGLDLLRVVGIGGILYLIVLMTILGNLIVLTAVMKYPHLRTKVTN
YFICSLAVSDLMVGFLVLPWAAAQELLGYWPFGQFCSTFVSLDVMCCTASILNLCVISVDRYWAI
SDPFKYERDMTCCRAALMIGLAWCLSAMISFIPIGLGFHHIGETITVTQRQCESNMNQVYAITSS
FVSFYIPVIIMVMAYSKIMQIARRKYEDIVALHRSVNQNTKSVQESRTALRRETRIFKTLACILG

VFIVCWLPFFILNCLVPFCPLKTGGKANFTCVSDTVFTVFEWLGWVNSTLNPVIYAFNNDFRKAF
WRMLCRRVPFSRTEAAATSCPCCPAEAYEMAEYKTSNRSPKSV*

>Bfl-drd1,5

MTMVAVNNSTNSSDMFQGWEDLDGVDLLRVVGVGGTLYLIVLLTILGNLMVLTAVMKYPHLRSKV
TNYFICSLAVSDLMVGFLVLPWAAAQELLGYWPFGQFCSTFVSLDVMCCTASILNLCVISVDRYW
AISDPFKYERDMTCCRAAIMIGLAWCLSAMISLIPIGLGFHHIGETITVTQRQCESNMNEIYAIT
SSFVSFYIPVIIMVLAYSKIMQIARRKYEDIAALHRSVNQNTKSVQESRTALRRETRIFKTLACI
LGVFIVCWLPFFILNCLLPFCPLRTGGKANFTCIDHTVFTVFEWLGWANSMLNPIIYAFNNDFRK
AFWRMLCRRVAYRGSDTAVNFPCCPADTYEMAEYRTSNRAPRSV*

>Mm-drd2

MDPLNLSWYDDDLERQNWSRPFNGSEGKPDRPHYNYYAMLLTLLIFIIVFGNVLVCMAVSREKAL
QTTTNYLIVSLAVADLLVATLVMPWVVYLEVVGEWKFSRIHCDIFVTLDVMMCTASILNLCAISI
DRYTAVAMPMLYNTRYSSKRRVTVMIAIVWVLSFTISCPLLFGLNNTDQNECIIANPAFVVYSSI
VSFYVPFIVTLLVYIKIYIVLRKRRKRVNTKRSSRAFRANLKTPLKGNCTHPEDMKLCTVIMKSN
GSFPVNRRRMDAARRAQELEMEMLSSTSPPERTRYSPIPPSHHQLTLPDPSHHGLHSNPDSPAKP
EKNGHAKIVNPRIAKFFEIQTMPNGKTRTSLKTMSRRKLSQQKEKKATQMLAIVLGVFIICWLPF
FITHILNIHCDCNIPPVLYSAFTWLGYVNSAVNPIIYTTFNIEFRKAFMKILHC

>Dr-drd2

MEVFTAYAFNESFFENASRDFNATEQGGRHQYNYYAMLLTLLIFVIVFGNVLVCMAVSREKALQT
TTNYLIVSLAVADLLVATLVMPWVVYLEVVGEWRFSTIHCDIFVTLDVMMCTASILNLCAISIDR
YTAVAMPMLYNTRYSSKRRVTVMISVVWILSFAISCPLLFGLNNTVTHDDALCVIANPAFVVYSS
IVSFYVPFIITLLVYVQIYVVLRKRRKRVNTKRTCPVTDMDMSSTIKKCTHPDDVKLCTVIVKSS
GNCPVNNKNVIFIKEVVNNGDDIQMDEITNRNPSRQRKQDQSGGSQQNSRLVNSNLRETDISPPS
PEAGVKPERNGNTSSITKGAKAFEIQVSPTGKTQTSVKTLNKRKISQQKEKKATQMLAIVLGVFI
ICWLPFFITHIVNTYCQVPPELYTAFTWLGYVNSAVNPIIYTTFNIEFRKAFIKILHC

>Dr-drd3

MGFCFGSSLEKLNRSSTLMAMFSSGEWLWNDSEHSFTPGGNYSPASGVEEAKRNYYAMLYSLLIL
AIVFGNVLVCIAVLRERALQTTTNYLVVSLAVADLLVASLVMPWAVYLEVVGGAWLFSRLYCNVF
VTLDVMMCTASILNLCAISIDRYTAVVMPVLYNTTHSSRKRVSVMIATVWVLAFAVSCPLLFGFN
TTDDPAVCSISNPDFVIYSSVVSFYLPFAVTLLVYVRIYIFLRRRRKKITFRQGSGKVQPASAPP
SVEMCLQDDAHKEKRDLSPIRINVITESKEQVIRPRLLANCLRRKRPQTAPAENSLLPPVITLNY
CSISQASFARTEQDANREEEEGGDEEQVAVRSCEVKKLANGRTHTSLRPPRAAHAMVCPSQARCR
SMHSKEKKATQMLAIVLGVFLICWLPFFVTHILNTHCRACHIPPEVYSAFTWLGYVNSALNPVIY
TTFNIEFRRAFIKILSC

>Mm-drd3

MAPLSQISSHINSTCGAENSTGVNRARPHAYYALSYCALILAIIFGNGLVCAAVLRERALQTTTN
YLVVSLAVADLLVATLVMPWVVYLEVTGGVWNFSRICCDVFVTLDVMMCTASILNLCAISIDRYT
AVVMPVHYQHGTGQSSCRRVALMITAVWVLAFAVSCPLLFGFNTTGDPSICSISNPDFVIYSSVV
SFYVPFGVTVLVYARIYMVLRQRRRKRILTRQNSQCISIRPGFPQQSSCLRLHPIRQFSIRARFL
SDATGQMEHIEDKPYPQKCQDPLLSHLQPLSPGQTHGELKRYYSICQDTALRHPNFEGGGGMSQV

ERTRNSLSPTMAPKLSLEVRKLSNGRLSTSLKLGPLQPRGVPLREKKATQMVVIVLGAFIVCWLP
FFLTHVLNTHCQACHVSPELYRATTWLGYVNSALNPVIYTTFNIEFRKAFLKILSC

>Mm-drd4

MGNSSATEDGGLLAGRGPESLGTGAGLGGAGAAALVGGVLLIGLVLAGNSLVCVSVASERTLQTP
TNYFIVSLAAADLLLAVLVLPLFVYSEVQGGVWLLSPRLCDTLMAMDVMLCTASIFNLCAISVDR
FVAVTVPLRYNQQGQCQLLLIAATWLLSAAVASPVVCGLNDVPGRDPAVCCLENRDYVVYSSVCS
FFLPCPLMLLLYWATFRGLRRWEAARHTKLHSRAPRRPSGPGPPVSDPTQGPFFPDCPPPLPSLR
TSPSDSSRPESELSQRPCSPGCLLADAALPQPPEPSSRRRRGAKITGRERKAMRVLPVVVGAFLV
CWTPFFVVHITRALCPACFVSPRLVSAVTWLGYVNSALNPIIYTIFNAEFRSVFRKTLRLRC

>Dr-drd4a

MPANLTISTHTTNYNFPALIFGILLIIIIICGNVLVCLSVYTEKALKTTTNYFIVSLAVADLLLA
VLVLPLFVYAEFQDGVWSLNMTVCDGLMTMDVMLCTASIFNLCAISIDRFIAVSIPLNYNRKHVD
QRQIVLLSATWILALAVASPVMFGINNVPNRDHSECKLEDNNYVIYSSVCSFFVPCPIMLLLYCG
MFRGLRNWEEARKAKLRSNMEACRKLQEAAASLQPLSGLPPPLPPVIEKDITLEELDQDHYPDPD
SPDPVSMLAYPGGGYNQDQNQNRQRKRAKINGRERKAMRVLPVVVGAFLFCWTPFFVVHTMRALC
EDCEIPGSVTSIVTWLGYVNSAINPIIYTVFNTEFRKFFRKFLPTLPGCC

>Bfl-drd4

MLCTASIFNLVAISIDRFYAVTWPVRYSKHKSNTRLAITITLVWTLSFVICAPLLFGSNYPAKGE
RTPEMCGIFNNNYMIYSSMCSFFIPCTAMLVLYYKILKKIKERSKKMAKKMPKSTSAGMICGDTV
IANTTTSSGIALKENGASKKTLSTPVLDGHAGHAVSAVDANSRPSSECAKLPKTRLDAIKIKLKG
KVKDEFPNKREQKAINVMKIVIGVFLICWTPFFVTNVVRACSTCTIPDVLFSIVTWLGYLNSGMN
PIIYTIFNQEFRSAFRKLLCKTH

>Mm-drd5

MLPPGRNGTAHRARLGLQRQLAQVDAPGGSAAPLGPAQVVTAGLLTLLIVWTLLGNVLVCAAIVR
SRHLRAKMTNIFIVSLAVSDLFVALLVMPWKAVAEVAGYWPFGAFCDIWVAFDIMCSTASILNLC
IISVDRYWAISRPFRYERKMTQRVALVMVALAWTLSILISFIPVQLNWHRDKAGSQGREGLLSNE
TPWEEGWELDGRTENCDSSLNRTYAISSSLISFYIPVAIMIVTYTRIYRIAQVQIRRISSLERAA
EHAQSCRSRGACEPDPSLRASIKKETKVFKTLSVIMGVFVCCWLPFFILNCMVPFCSSGDAQGPR
TGFPCVSETTFDIFVWFGWANSSLNPIIYAFNADFRKVFAQLLGCSHLCFRTPVQTVNISNELIS
YNQDTVFHREIAAAYVHMIPNAVSSGDREVGEEEEAEEEGPFDHMSQISPTTPDGDLAAESVWEL
DCEEEVSLGKISPLTPNCFHKTA

>Rt-drd5

MRNGSLWKEPGGDKVLAGRVLTGALLSALILFTLLGNALVCVAVLRYHNLRAKVTNLFIVSLALS
DLLLAVLVMPWKAAAEVASGWPFGPFCKVWLAFDIMCSTASILNLCVISVDRYWAIASPFRYERR
MSRRLALLVVGLTWALAVLVSFVPVQLDWHTAPAARSSSAPRPPPRGQRPPANCDSSLNRAYAIS
SSLISFYIPVAIMLVTYDRIYRIARVQIRRIAALERAAQHARSCRQEQHRSLRSSFYKETKVLKT
LSVIMGVFVCCWLPFFVLNCLVPFCDRRSPSPPCVSETTFDVFVWFGWANSALNPVVYAFNAEFR
QAFSGLLGCTRVCCAPTAGRVHSANISNELVSYHQETALQREVACADMIPNAVQRAPAAFDSMSQ
ISAAADEDDEEEEPASDSAAELGLTFCKITPFTPNGVH*

>Le-drd5

MDGAPGVKSELATRILTGLLLSLLILFTLLGNALVCVAVVHFRHLRSKVTNVFIVSLALSDLLVA
VLVMPWKAVAEVAAYWPFGRRFCSVWVASDIMCSTASILNLCVISVDRYWAISSPFRYQRKMTHR
AALVMIAVAWAFSLLISFIPVQLSWHSIREPAGAGHSNNSSAAFAHPPESCDSSLNRTYAISSSL
VSFYIPVAIMLVTYTRIYRIARIQIRRIASLERAAEHAQSCRRRRLEPRQQQQPMHHHSALERSF
KKETKVLKTLSIIMGVFVCCWLPFFVLNCMVPFCDRSSPAAAARLPCVSDTTFDIFVWFGWANST
LNPVVYAFNAEFRKAFSSLLGCQDFCSGNQVETVNISNELVSYNQDTVFHREIVTAYVTMIPNVM
GCMEDDQIFDRMSQILATNEQATETASEMEGDADLSLGKITPCTPNGLH*

Histaminergic receptors

>Mm-hrh1

MSLPNTSSASEDKMCEGNRTAMASPQLLPLVVVLSSISLVTVGLNLLVLYAVRSERKLHTVGNLY
IVSLSVADLIVGAVVMPMNILYLIMTKWSLGRPLCLFWLSMDYVASTASIFSVFILCIDRYRSVQ
QPLRYLRYRTKTRASATILGAWFLSFLWVIPILGWHHFTPLAPELREDKCETDFYNVTWFKIMTA
IINFYLPTLLMLWFYVKIYKAVRRHCQHRQLTNGSLPTFLEIKLRSEDAKEGAKKPGKESPWGVQ
KRPSRDPTGGLDQKSTSEDPKVTSPTVFSQEGERETVTRPCFRLDVMQTQPVPEGDARGSKANDQ
TLSQPKMDEQSLSTCRRISETSEDQTLVDRQSFSRTTDSDTSIEPGLGKVKARSRSNSGLDYIKV
TWKRLRSHSRQYVSGLHLNRERKAAKQLGCIMAAFILCWIPYFIFFMVIAFCNSCCSEPVHMFTI
WLGYINSTLNPLIYPLCNENFKKTFKKILHIRS

>Dr-hrh1

MCFLTTLTSCDTLERPMESTTTLTTANSQFKRIDNTTLLIPSAPLHHHINNAVLGIFLGMLSLLT
VIMNLLVLFAVRKERTLHTVGNLYIVSLSIADLIVGATVMPLNLVYLLEDQWKLGHVVCQFWLVM
DYVASTASIFSLFILCLDRYRSVRHPLQYLKYRTRGRATLLICSAWLLSMTWTIPILGWRMFASV
DKKTELENQCDTDFRFVTWFKVLTAILNFYIPSFLMLVFYSQIFIAVRDHYREWENFAGPALKTD
ATDTLTNGLQLQITKASEKESLALNAYSQNEGLLDQYSLEQPYNSRENTEEMISEPKRKMCYKKK
AIFNLSKRMRKSVPDSEISFQSDDGETVAEGRPSLSLAFLQSENTAQPQTFINVSDCNVLVPNSV
GNICESTPTVDIHNYTAVLCTPPSPPSPWAENSPPLDASIALPAKQSWQKLCEQSKQSIHSMRIR
KERKAARQLGFIIGVFMVCWIPYFITFMVMALCETCVHHDLHMFTIWLGYINSTLNPFIYPLCNE
NFKRVFKKIFHMSR

>Dr-hrh2b

MTIRALCWVVLVAFIALTICGNILVCMAVATSRRLHQLSSCFILSLAVTDLLLGLLVLPLSAMLE
LRNGKWPLGGVFCNIYISMDVMLSSASILTLLAISVDRYLAISNPLFYPRRVTPRRVAIALTAIW
TCSLAVSFVSINLGWNSPDFRVQNLDWSMWDEGEEGRTCRYEWNNNYVLLKAFGIFYLPLLVMCG
MYHRIFCVAREQVRRIRAATPSSAQVANAAATAREHKATVTLAAVLGAFIICWFPYFTYFTYMGM
WAIHPNKLTHSIVLWLGYLNSALNPILYPALNRDFRQACGQLLCRCGKRGDFNTSRRKTFIALGT
IKVIKGSLAH

>Rat-hrh2

MEPNGTVHSCCLDSMALKVTISVVLTTLILITIAGNVVVCLAVSLNRRLRSLTNCFIVSLAATDL
LLGLLVLPFSAIYQLSFTWSFGHVFCNIYTSLDVMLCTASILNLFMISLDRYCAVTDPLRYPVLV

TPVRVAISLVFIWVISITLSFLSIHLGWNSRNGTRGGNDTFKCKVQVNEVYGLVDGLVTFYLPLL
IMCVTYYRIFKIAREQAKRINHISSWKAATIREHKATVTLAAVMGAFIICWFPYFTAFVYRGLRG
DDAINEAVEGIVLWLGYANSALNPILYAALNRDFRTAYQQLFHCKFASHNSHKTSLRLNNSLLPR
SQSREGRWQEEKPLKLQVWSGTELTHPQGNPIR

>Dr-hrh2

MTSQIALAVTLSVLILLTVSGNILVCLAVYATRRLRNVTNCFIVSLAVTDFLLGALVLPFSTLYQ
VTGDWPLGAHFCNIYISLDVMLCTASILNLFAISLDRYFAVTAPLRYPMLVLPWRVGAILATIWL
VSVGVSFVPIHLGWNTRDLSVQNIREGDHARDCRFELNPTYAIVDAFSTFYLPLLAMCWSYHRVF
RIARMRAKRIISTRRGSTSSQGVTILTLREHKATVTLAVVLGAFVVCWFPYFTFFTIMGIRNEEN
PPQTAQSVVLWLGYANSALNPVLYATLNRDFRSAYAKLLCGGRRCKTETTIPIAATAAGTMTGHA
HSSKGAGFLPRTCVLLKDSENRMLDDNSATNGATVTVVANGSKR

>Le-hrh2

MENGQNITSECESRSVLSTVLVGLVMGMITLITIFGNVLVCLVVGLNRKLQSHTNSLIVSLAATD
LLLGLLVMPFSTTLQLLNSEWPFGPTFCNIYTSMDVMLSTASILNLFAISLERYLAVTAPLRYNI
VITPNRVIVSLVFIWCISIMFSFLPINLGWNSKDALTQSSNETNQCHLEMNPSYAIVDAFITFYI
PLAIMCLTYYKIFKIAREQAKRISSATRSTLPGLKEHKATVTLATVMGVFIICWFPYFTVFLHAG
ISGIQSDKTVFAVVLWLGYVNSALNPIVYAALNREFRMAYQKLLFCRKRNACNRGSSINYQVGRL
RNEQCHQLVRILDDNEPSSDNTITKDICLCTEDSER*

>Rt-hrh2

MEVEGSQNATTSSENRTVLSTVLIGLVLGVIVIITIFGNVLVCL
AVGLNRKLQNHTNCFIVSLAATDLLLGLLVLPFSAISELFEWPFGDTFCNIYTSLDVMLCTASIL
NLFMISLDRYYAVTAPLRYAVFITRNRVVISLGLIWCISIMVSFLPINLGWNTKDLSVQSLANTN
QCHLELNKVYALIDAFITFYIPLPIMCLTYYRIFKIAREQAKRINTVTGCTALNRSLPTVKEHKA
TVTLAIVMGVFIICWFPYFTVFTHEGISGRQANKTVFAVVLWLGYVNSALNPILYATLNRDFRKA
YEKLLCCKKRNSYHKDPPDKPLSCRPLHNGDPRNERCHQLGPDMEENELTSEDRNGENVATYKED
SERSLDGIVHSREVFLEGETNTPQ

>Alu-hrh2

MCLLPHPDRAAGGSMDNLSTTTVTDNATLLPETTRAPYGGKTLFAAVVLGTVLGLITLGTVVGNV
LVCLAAVVNRRLRNITNYFVVSLAVADLLVGVLVLPFSTIFEVTRYWNFGLILCNMWVSTDVLCC
TASILNLFAISLDRYYAITRPFTYSNKMCRRKAFMAIAIVWTVSLLVSFLPIWVGWNTQDGRLQN
IDDPTKCNFDNTNVPYIMIVAFGTYYIPLIIMCVTYFRIFLIAKEQASRINALQPEVRDANRRQN
QNLANEHKATRTLAAVLGAFIICWTPYFTVFTITPLCGCTIPHQLYSVVLWLGYVNSLINPCVYA
FMNKEFRRAFKKLLCFQTNSICFFSNPRNRRLDTPTTSTAHRN*

>Bfl-hrh2

MENVSVAVTDNATLLPGGGVDARAPYGGKTLFAAVLVGTFLGIITLGTIIGNVLVCLAAVVNRRL
RNITNYFVVSLAVADLLVGVLVLPFSTIFEVTRYWNFGLIMCNLWVSLDVLCCTASILNLFAISL
DRYYAITRPFTYSNKMCRQKAFMAIAVVWIVSLLVSFLPIWVGWNTQDGRLQNVDDPTQCSFNNT
NVPYIMIVAFGTYYIPLIIMCVTYFRIFLIAKEQANRINALQPEVRDANRRQNQNLANEHKATRT
LAAVLGAFIICWTPYFTVFTITPLCGCTIPEQLYSVVLWLGYINSLLNPCVYAFMNKEFRRAFKK
LLCFQTGNTVCFCGNPRNWAQDEPTTSTAPRQS*

>Dr-hrh3

MQSSLLSVAHSPGIPTAVSLMWKSGNHQSSNWSGMERENATSLADLDTFENRRAQYGQFSPSTSI
FLTVLMTLLVFATVLGNALVILAFVVEKSLRTQGNFFFLNLAIADFLVGGFCIPVYIPYVLTGEW
RLGRGLCKLWLVVDYMLCTASVFNIVLISFDRFQSVTKAVSYRCQKGITKDAVLKMLCVWLAAFL
LYGPAIISWEHITGGSVVPDGECYAEFYFNWYFLMTASTVEFFTPFISVTYFNLSIYINIRNRCA
MREEQPTYVRLRSFKMKPLGAGDVQRVFFVRPVEESRVADLASRSRCCRLASTAKVSAAEFGNGR
QSKRRDSTLADLPPLQVEERILAASEAQFHYVDHSAGPHRHRPDMVASLANRFRLSRDKKVAKSL
AVIVCVFGLCWAPYTLLMIIRAACHGQCVQHYLYEISFWLLWINSSINPILYPLCHSSFKRAFSK
LLCPSKTKIQPQNIDQKY

>Rt-hrh3 _

MAKLGTLRAIAWTAPLLCASLGAEVNSSQRSLPNLAQSTGCLPFHQFSATSAGLLITLMVLLVII
TVLASVIPAFVVDKSLRSQNNFFLYFLVGAFCIPLYVPXALASRWMLGKTLCKFXLVMNYLLCTS
SVFNTVLISYVRFDCVARPFSYQAQQWKRRNAAIKIMLVRLTAFLLYGPAITAWEXVIGRNIVLV
GECYAEFYNNWYFLITASTVEFFVPFISVMYFNLSIYLNLQQRTRSQSNSFWNTKSKSSLEHLEA
GSNLACSLKICKSDQKTKLIKSTVCDLTTMSSEIKIKVERSETLDHLKRRNDLGRTIPSSEVKCL
MKEEISQSQLFSKRKIKLISQNVVQHFRLLRDKRTAKSLAVIVCVFGICWAPYTLLLVVRAGCQG
HYMAYDWYEASFWFLWINSAINPILYPLCHKNFKRAFMKLLCPTKLKMRPRSSRQ

>Dr-hrh4

MKTLLSSSAAGKASHDDFRPVAASPNVLDLTMVTCFNTSGSNGTTVSPAEAPFPGYTMVILAILM
ATLVVVVVVGNALVILAFVVDKSLRNQCNYFFLNLAISDFLVGAFCIPLYMPYILTGRWMLGRGL
CKLWLVMDYLLCTASVFNIVLISYDRFLSVTRAVRYRAQQGVTRLAVLKMLAVWVSAFLLYGPAI
IFWELLVGESIVPEDECFAEFYCTWYFLLSASTFEFFSPFISVAFFNFSIYLNIQRRNRSRMAHV
EAEKEDGWRVIDGQTSSVFFVKTRKVSSSEPAAVSAVIEDDEEVSPSSSGDPSSTHSVSLTMKTT
IKKRGLFGGWVRTRIIRAQEVTNPCAPCRSGGHARLSRDKKIAKSLAVIVCVFGVCWAPYTLLMI
IRAACSGSCVEHHWYEVTFWLLWLNSAINPFLYPLCHSSFRRAFAKILCPKRQSVQPHEESPSCP

Muscarinergic acetylcholine receptors

>Mm-chrm1

MNTSVPPAVSPNITVLAPGKGPWQVAFIGITTGLLSLATVTGNLLVLISFKVNTELKTVNNYFLL
SLACADLIIGTFSMNLYTTYLLMGHWALGTLACDLWLALDYVASNASVMNLLLISFDRYFSVTRP
LSYRAKRTPRRAALMIGLAWLVSFVLWAPAILFWQYLVGERTVLAGQCYIQFLSQPIITFGTAMA
AFYLPVTVMCTLYWRIYRETENRARELAALQGSETPGKGGGSSSSSERSQPGAEGSPESPPGRCC
RCCRAPRLLQAYSWKEEEEEDEGSMESLTSSEGEEPGSEVVIKMPMVDPEAQAPTKQPPKSSPNT
VKRPTKKGRDRGGKGQKPRGKEQLAKRKTFSLVKEKKAARTLSAILLAFILTWTPYNIMVLVSTF
CKDCVPETLWELGYWLCYVNSTVNPMCYALCNKAFRDTFRLLLLCRWDKRRWRKIPKRPGSVHRT
PSRQC

>Dr-chrm2a

MDTINFTFWNASDGNETMETADESPYKTVEVVFIVLVAGSLSLVTVIGNILVMLSIKVNRSLQTV
NNYFLFSLACADLIIGLCSMNLYTVYIVIGYWPLGPVVCDLWLALDYVVSNASVMNLLIISFDRY

FCVTKPLSYPVKRTTKMAGMMIAAAWVLSFILWAPAILFWQFIVGGRTVPEKECYIQFFSNAAVT
FGTAIAAFYLPVIIMMVLYWQVSRASKSRVKKDNRKPSGGNLDVASSNQIRENSANKPTNNNLTA
EETDRGQTQLTDDTINQHDAKLQNGKAPSTASGEAEEAGQANCIPAEEKESSNDSTSGSGAVTNQ
KEEAAPPSSAAANDSQTSTRHRAKAGGSKLTCIKIITKSPKGDCYAPSNATVEIVPAVERQNHVA
RKIVKMTKQPPKKKKAPSSREKKVTRTIMAILVAFVATWTPYNVMVLINTFCSSCIPNTVWTIGY
WLCYINSTINPACYALCNITFKKTFKQLLLCQYKNIRSTR

>Dr-chrm3b

MNLTLSAEPGLFLTNSSPGMRANPVTAFMSHDLHPANDSLHNITQIPSVLSDPLGGHSVLQVVMI
ALLCGSISLLTIIGNTLVLLSFKLNKQLKTVNNYYLLSLAFADLIIGVLSMNLYTTYIIMDQWAL
GNFACDLWLAIDYVASNASVMNLLVISFDRYFSVTRPLTYRAKRTTKRAMTMIGLAWSISFILWA
PAILFWQYFVGERTVEPDKCYIQFISEPITTFCTAIAAFYLPVTIMTVLYWRIYKETENRSRELA
GLKASGNVEEESVVVVPQTGSSRSHSSYELQPAAPMRKTKTRRLWFWSRGDVERSSSDSWNNNET
AISIDQSDDDEEEDAEHIYTIEKQDLEAKPSKFGVSKSSTLDSKDQPVKSARKTNKRKKLSLVKE
KKAAQTLSAILLAFIITWTPYNIMVLLNAFCDGCIPETLWALGYWLCYVNSTVNPMCYALCNKAF
RTTFRSILLCQWRKKKLNKAQIQKRAPAATYRKHTQSLD

>Bbe-chrm

MAFPTIPPPGPDYTTLLYGTEVVPVVYDAVNGTPVWSTSGTFNISCGNESTTLPAECNVTTAPPL
DPLGGHQVWEVVLICLLTCSLSIMTVGGNILVFVSFRVNRQLHTVNNYFLLSLAVADTLIGLLSM
NLYTVYMVMGRWALGPAICDLYLTVDYVSSNASVMNLCVISFDRYFSITRPLTYRAKRTPKRAAW
LIALAWVTSVLVWAPLIVGWQYIVGGRNVPENECYIHPEQPAKPLPSAAALAASRPKLSAAQLWK
RAAENTTRTKLKSDRARKISMVLKEKKAAKMLSAILLAFIITWLPYSVTVLFKPFYKVPTTVWNV
GYWLCYVNSTINPWCYALCNRTFRKTFKDILVCRVKKQNFRQLQSKRLY*

>Cin-chrm1-5

LLICILAGACSLLTIVGNVVVIVSFFINKSLRNFSNYLILSLAISDLTIGAFSMNVYTSYNVKNE
WILGEVMCKAWLAVDYTASNASVMNLLVICVDRYLAITKPVKYRKWCTPQRAAIAIAGVWILSFL
MWCPAIVLWDLFSTDTKMPTNDCYIPFMKDNAWVTVATIVGAFYIPATAMCALYYQVISKLRKRR
LKCFYREPQRVVRERRVTVLLKFILTCFIVLWLPYSLIIILVTSFWPSCMVPIYIWNLSYWLCYLN
STVNPFCYGFCNENFRQTFKVIIT

>Csa-chrm

VTICFLSALASILTIGGNVMVIVSFVINRALRTVNNYLILSLAAADLVIGVVSMNLFTVFIVSGS
WKLGAVLCDVWLALDYVASNASVMNLLIICLDRYYSVTKPVEYRNQRTPKRFITAIIGAWSISFI
LWAPWVLFWQFIVNKRTVPENASQTGADSPRSRTNSGRLLFIRRQIKKAPDTDSNLDSGGIIKVT
ERLKLQVVKKKLQSLNKETKAARLLASILAAFILLWLPYNIMALVEAFCPLAEKCIPDLAWNFGY
WFCYLNSTLNPVCYAMCNKNFQQTFKFLLS

>Csa-chrm1-5

AISIMAVILMVVTVTGNFLVIASYRSNRNLRTINNMFLVSLACSDLVIGVVSMTLYPIYMISRKW
MLGPILCDIWLCIDYTLSMASVANLMLICLDRYFSVTRPFTYRDSRTRYKTRVFILMAWVFSMLL
WSPAIVIWPIVRGRTVPDDQCQIQFFEDATITLVTAILAFYLPVLVMTVLYALIYRETRKCSQYL
EYLRNYGKSRSNTSSSPKAARTLSAILLAFIVTWLPYNVCAVYKSFCGEECVPSAAWDAAYYLCY
INSTVNPFCFALCNKTFRETFVKLLCGRKRGKLDDLPRSKRKCKSMKLQPSTS

>Mm-chrm4

MANFTPVNGSSANQSVRLVTTAHNHLETVEMVFIATVTGSLSLVTVVGNILVMLSIKVNRQLQTV
NNYFLFSLACADLIIGAFSMNLYTLYIIKGYWPLGAVVCDLWLALDYVVSNASVMNLLIISFDRY
FCVTKPLTYPARRTTKMAGLMIAAAWVLSFVLWAPAILFWQFVVGKRTVPDNQCFIQFLSNPAVT
FGTAIAAFYLPVVIMTVLYIHISLASRSRVHKHRPEGPKEKKAKTLAFLKSPLMKPSIKKPPPGG
ASREELRNGKLEEAPPPALPPPPRPVADKDTSNESSSGSATQNTKERPPTELSTTEAATTPALPA
PTLQPRTLNPASKWSKIQIVTKQTGSECVTAIEIVPATPAGMRPAANVARKFASIARNQVRKKRQ
MAARERKVTRTIFAILLAFILTWTPYNVMVLVNTFCQSCIPERVWSIGYWLCYVNSTINPACYAL
CNATFKKTFRHLLLCQYRNIGTAR

>Rt-chrm

MIPDYNSTASPFFFNSSFSWNHDSQTQVVLGSRPVDRSANGLFRSAVLSPTTSLGANGSLLSNST
TDPLGGHTVWQVVLIAFFTGILSLVTIIGNILVIVAFKVNKQLKTVNNYFLLSLAFADVIIGVIS
MNLYTTYIIMDRWALGNVACDLWLAIDYVASNASVMNLLVISFDRYLSITRPLTYRAKRTPKRAG
IMIGLAWIVSFVLWAPAILFWQYFVGERTVPPNECFIQFLSEPIITFGTAIAAFYLPVTIMTILY
WRIYKETEKRTKELAALQASTTDMETIHFVHQTGSSRSCSSNELQQAGMKEQRRLRRCRFWANSK
TWKSSTEGEQEGSSSDSWNNNDGGGSLDHSASSDEDDVVSETRAIYSIVLNLPGLKTAASLSRST
NANNIDLCSDELAKADNSVKERKFRSQEFKDPDEQGNSFHQHFAKLPVQSLPSIHPSKTSGGIAS
LAKSSTSMSLRETTVAKAFASKARTQITKRKRMSLVKEKKAAQTLSAILLAFIITWTPYNIMVLV
NTFCDKCIRDSLWQLGYWLCYVNSTVNPMCYALCNKTFRNTFKMLLLCHFEKRKKRRQQYQPKQA
VIFHKRIPCEPS*

>Pm-chrm

MEGINIQDEGIVWSPTSQNPSAPLTLNSSAADDPLAGEQNVAQVVVIAVVAAMFSLITVVGNTLV
MLSFRVSRQLKTVSNYFLLSLAAADLIVGLFSMNLYTAYILTGRWAMGAVMCDLWLTLDYVASNA
SVMNLLAISFDRYFSITRPLKYKAKRTKRRASIMIGLAWAVSLVLWAPAILFWQHMVGKRTVPQD
QCYIQFLSEPIITFGTAIAAFYLPITIMMVLYWRIYRETRRRSRQFATLQGSESKSRSHVPTGTR
GCLTFLKDDDCDDDYDEDCGARAHDGIRSPKSKTSQGSQPWKCSLQSCEGHVETTHEEEEDDDEE
EEEEAVSLGCAISSDDDEQEPANERADSCAKPAAAATDGSSPRPSAPSDVPDDPVAAADSVVGNG
GETCAEPQHARISSTVRGRDLGRNNCVTLADMRIEESLLPRDAERESPREDEPAADKIVLRGRSH
RGRCKGGKAHSSTARRHCSLLARDNKAVRILCAILLAFIVTWTPYNIMVLVSTFCDDCVPKKLWH
LGYWLCYVNSTVNPMCYALCNMHFRKTFKTILSCKWNAVT*

Serotonergic receptors

>Pm-htr1A

AAVTAMEAHNITGSAPALAVSAAQGNSSEPSSVAYTVISSLCLTALILCAIFGNSCVIAAIALER
TLQNVANYLIGSLAVTDLLVSILVLPMAALYQVLDRWTLGQVVCDIFIALDVLCCTSSILHLCVI
ALDRYWAITNPIDYVNKRTPRRAVIMISLTWLVGFSISIPPMLGWRTPEDRADPNACKISQDHGY
TIYSTFGAFYIPLILMLVLYSRIFRAARFRIRKNVKKATEKKSFDAPFAISNRAEMPSAAAEAET
AAPPSPAPCNGDSKQRSHRDLALPEAVGARSPSSLAHSSARPHLSPAMMPTQQQHGNGTGADAC
RRKTALLISPFCGERCHLPPGVFAVINWLGYSNSLLNPIIYAYFNKDFQNAFKKIIKCKFCRQ

>Dr-htr1a

MESYNNTTESQDWSGNATSVSEVALSYQIIGSLFLAALILFAILGNACVIAAIALERSLQNVANY
LIGSLAVTDLMVSVLVLPMAALYQVLNKWTLGQEMCDIFISLDVLCCTSSILHLCAIALDRYWAI
TDPIDYVNKRTPRRAAILISLTWLIGFSISIPPMLGWRKPEDRADPDACTISQDHGYTIYSTFGA
FYIPLILMLVLYGRIFRAARFRIRKTVKKTEKAKIADKCLAVSPALFPRKANGEVGKTWRRSVEP
CANGALKNSDDGESFEITEVQSISKNHLSLPNNPQPCFENRNEKNTEAKRKVALARERKTVKTLG
IIMGTFIFCWLPFFIVALVLPFCQDCFMPEWLGAVINWLGYSNSLLNPVIYAYFNKDFQNAFKKI
LKCKCIRQ

>Dr-htr1fb

MDPINCTSVDLSDVLASKMSPSKILLSLTLSFLAVATTAINSLVITAILITRKLHQPANYLICSL
AVTDLLVAVLVMPVSIVYIAEETWVLGPIVCHLWLGVDVTCCTCSILHLAAIALDRYRAITDAVA
YSQKRTYKRVIVTILSLWTLSILVSLPPLVWRKFPKVEFKDGKREPMDCLIEHDHVAFTVYSTFG
AFYIPLALILVLYYKIYKAAEMLRNRRGSSRLVKQTVSSVMLPGMSSDKDIALSPDSFCPIEKSF
SDPSTDGERVRITSSSGNLIRVRRNPGARERRAALTLGLILGAFVVCWLPFFLKEVIVNICPTCT
TSAVLADFLTWLGYLNSLINPLIYTIFNEDFKKAFKKLLPLCCSTVL

>Mm-htr1b

MEEQGIQCAPPPPAASQTGVPLTNLSHNCSADGYIYQDSIALPWKVLLVALLALITLATTLSNAF
VIATVYRTRKLHTPANYLIASLAVTDLLVSILVMPISTMYTVTGRWTLGQVVCDFWLSSDITCCT
ASIMHLCVIALDRYWAITDAVEYSAKRTPKRAAIMIVLVWVFSISISLPPFFWRQAKAEEEMLDC
FVNTDHVLYTVYSTVGAFYLPTLLLIALYGRIYVEARSRILKQTPNKTGKRLTRAQLITDSPGST
SSVTSINSRAPDVPSESGSPVYVNQVKVRVSDALLEKKKLMAARERKATKTLGIILGAFIVCWLP
FFIISLVMPICKDACWFHMAIFDFFNWLGYLNSLINPIIYTMSNEDFKQAFHKLIRFKCAG

>Rt-htr1

MNFSNCTNESCQGQSKTLTERTAIALILSLIAIMTTLLNSAVMVAICITKKLHQPANYLICSLAL
TDLLVAILVMPISITYITTDAWVLGYIVCEAWLSLDMTFCTCSILHLCVIALDRYWAITDAVEYT
PKRTAKRAGVMILTVWTISVFISMPPLFWRSHNVNSTGEKCIIQHDHVIYTIYSTFGAFYVPSAL
ILILYYRIYHAAKNLYQKRGSSRHFTGRKSESQISTCMNYKLTQTFCVSNLHSCEPVLKFHSDHR
TCRVPTLENRQGSTEERQQNSNSRERNAARILGLILGTFVFCWLPFFIKELCVGLHLWVASQVVT
DFLAWLGYINSLINPLLYTSFNEDFKLAFRKLIRCKTHG*

>Le-htr1

MNITDCPRGSGPERTKSFAEKTAVALTLSLVMILTVVLNGVVIAAISLTKKLHQPANYLICSLAL
TDLLVALTVMPLSILYISAETWLLGQAFCQAWLSLDMTFCTCSILHLSAIALDRYWAITDAVQYS
RRRTGRRVAGMILAAWVLSVSISMPPLFWRSRMVNGTMGQQCTIRHDHAIYTIYSTFGAFYAPLG
LILILYYRIYRAARTLHRRRASSCKLAHTSCPSEAPRPGSQLGREKGIGEQPVSMGRERRAARVL
GLILGAFVLCWLPFFVKELLAGMQVWAASPTLGDLLTWLGYLNSLVNPLLYTSFNQDFVTAFRRV
ASCGRYG*

>Rt-htr1b

MMNQSAECLAPNSVELQANSKHVNCSTKVNPPGGAGITAWGISASVFLCLITLATVLSNGFVIAT
IYQARKLHTPANILIASLAITDLLVSILVMPLSIVYTVSGTWSLGQVVCDIWLSSDITCCTASIL
HLCVIALDRYWAITDAVEYSMKRTPERAAGMIVTVWVISICISIPPLFWRQAKAGELMHCTVNTD

QIFYTIYSTFGAFYIPTLLLIALYGRIYVEARSRILKQTPKQTGKRLTKAQLVTHSSGSSSLSSV
NSRVNEFPTETGSPVNLNHVKVKVSDVLLEKKRISAARERKATKTLGIILGAFIVCWLPFFIITL
VLPICKEACWFHHAIFDFFNWLGYLNSLINPIIYTMSNEDFKQAFHKLTRCR*

>Cm-htr1

MMNRSIQCLEPNADQLQTNSKSTNCSTKLSTQEQESQITALGISLSVFLSLITLAIVLSNAFVVI
TISRTRKLQTPANILIASLAVTDLLVSILVLPISIVYTVSGTWTLGQIVCDIWLSSDITCCTASI
LHLCVIALDRYWAITDAVEYSSKRTPKRAAGMIVTVWVISICISMPPMFWRQSKAGELTHCVVNT
DQIFYTIYSTFGAFYIPTLLLIALYGRIYVEARSRILKQSPKRTGKRLTTAQLITNSPGSSSVSS
VNSRVNENSGETGSPVVLNHVKVKVSDTLLERKKILAARERKATKTLGIILGAFIVCWLPFFIIS
LVVPICKEACWFHPAIFDFFTWLGYLNSLINPIIYTMSNDDFKQAFQKLIRFRYFTS

>Cm-htr1

MDQQNFSTAASEYNTMSSAWDLLGLKVSLSVVMAVITLATILSNLFVIVAVCLTRKLHTPANYLI
ASLAVTDLLVALLVMPISIAFTMQRTWVLGQVMCNIWVLLDVTLCTVSILHLCIIALDRYWAITH
ALKYTKHRTPFRAGLMIAAAWGIAVCISTSPLFWISSRTQEKQDECVFNENVFYCIFATCVSFYI
PSLVLVFLYAKIYLAARSRILRAPSTKDSNIQHPEPTSSSQCSVNYNSQDMLNYYVEQPDRLIHI
KMSSNFLQKKNISAAKERKATKTLGLILGAFIFCWLPFFTTTLVITICQEACWSNPVLIDFFTWL
GFVNSLINPVIYTIFNKNFKRAFRKLVLSRLC

>Dr-htr2b

MANVRQTDSVDWPSHWAALLILLVIVPTIGGNILVILAVSLERKLQNATNFFLMSLAVADLLVGL
LVMPIALVTVLYNSTWPLADFLCPIWLFLDVLFSTASIMHLCAISLDRYIAIKKPIQHSQFKSRA
KVLAKIALVWLISIGIAIPIPIKGLQFFDHPNITFNKNHTCLLSPEGFRDFKVYGSLVAFFIPLA
IMMIIYLLTIQVLRKKAYLLRSRAARPSISTVFQQELSVLASPEKMVISNGIKRDRTLNPVNPIT
GDEVPLRRMSTIGKRSMQNLTNEQRASKVLGIVFMLFVVMWCPFFITNVTSVLCERCNGNLVDQL
LDIFQWVGYVSSGINPLVYTLFNRTFRLAFRRYITCNYKSVRTPKLQRRSKISFRSSVTENSKRF
MKHGMKNGISPVGYQSPIRHRSTQLQTSANIMLDTLLLTDNEDCKPDEHVSHV

>Csa-htr2b

WFMLFLIPIIVLVALGNVLVIISVCLDRRLRSSTNYFLTSLAVADLLVALVVMPPSLGMIMNNYK
WPFSDELCGVWAMLDVFFSTSSILHLCMISLDRYIALSRYRNYDKLPVAKSVAIRICTVWTMAIG
IAIPLPVLGAQDRSNLFIGDTCAINVREFAIFGSLVAFLLPLIIMFVTYTLTILTLRKQAREISA
AMDRPQGGTLRTARNVEQDGDGRSLLLTRQHSHVSGKEWKCSEVITPDYSALTKALRQVQALFGF
GVIIPTSDVKSPNTNRTRERNNNFASKISLQQTRSSTRRMSGSFKQRVMTSITNEQRASKVGFQV
LGLIFVLFCVFWCPFFITNVVSHLCDSCDQDLMGSFMNWFVWVGYVSSGVNPCVYTLFSKRFRET
FFNVLRGRF

>Rat-htr2A

MEILCEDNISLSSIPNSLMQLGDGPRLYHNDFNSRDANTSEASNWTIDAENRTNLSCEGYLPPTC
LSILHLQEKNWSALLTTVVIILTIAGNILVIMAVSLEKKLQNATNYFLMSLAIADMLLGFLVMPV
SMLTILYGYRWPLPSKLCAIWIYLDVLFSTASIMHLCAISLDRYVAIQNPIHHSRFNSRTKAFLK
IIAVWTISVGISMPIPVFGLQDDSKVFKEGSCLLADDNFVLIGSFVAFFIPLTIMVITYFLTIKS
LQKEATLCVSDLSTRAKLASFSFLPQSSLSSEKLFQRSIHREPGSYAGRRTMQSISNEQKACKVL
GIVFFLFVVMWCPFFITNIMAVICKESCNENVIGALLNVFVWIGYLSSAVNPLVYTLFNKTYRSA

FSRYIQCQYKENRKPLQLILVNTIPALAYKSSQLQVGQKKNSQEDAEQTVDDCSMVTLGKQQSEE
NCTDNIETVNEKVSCV

>Aj-htr4-like

RKKKTNYFIVSLAFADLLVALVVMPFAAIELTTGRWSYGETFCLVRTSLDVLLTTASILHLCCIA
LDRYYAICCQPLVYRNKMTPMRVALMLGGCWVIPTFISFLPIMQGWNSIGINHLIEERKFSGVAN
ETNCVFLVNRPYALICSAVAFYVPLGLMVLAYQRIYVTAMEHARQIGTLQRAGSAPDSYFGPDHQ
GSSRMKIETKAAKTLAVIMGCFCLCWAPFFITNVVDPFIRYRVPWQVWTAWLWLGYINSGLNPFL
YAFLNRAFRRAFLMILCCGDDRYTRHGSFSPSRPYSASVNGTSIALR

>Cm-htr4

MWPLTLMRSYFSLAFSASKSEPHDLTGLSMLQKTVLITFLAAVMMMTVLGNLLVMVAVCKDRQLR
KIKTNYFIVSLAFADLLVSVMVMPFGAIELIENNWAYGNTFCLVRTSLDVLLTTASILHLCCISL
DRYYAICCQPLVYSNKMTPIRVTIMLGGCWFIPLFISFLPIMKGWNSVGIQELIEDRKFRNDTKH
CVFMVNKPYAITCSVVAFYIPLVLMVLAYQRIYVTAREHVRQIDILKRAGASIDNGQQADQQGHH
RMKTETKAAKTLCIIMGCFCLCWAPFFITNVVDPFINYSVPDQLWTAFLWLGYINSGLNPFLYAF
LNKSFRCAFLTILCCGNRRYRRASFLGQTIPSSSTAINGSTQVLR

>Cm-htr4-like

MMTILGNLLVMVALCMDKQLRKIKTNYFIVSLAFTDLLVAILVMPFGTIETVQEHWTYGETFCLV
RTSLDVLLTTASILHLCCIALDRYYAICCKPLVYRNKMTPWRVALMLGGCWVIPCFISFLPILQG
WNTIGITETIEERKFNRAINETYCVFMVNKTYAITCSAVAFYIPLILMVLAYQRIYVTAMEHARR
IGNLQRAGAATDTDSTEHQGSSVMKRETKAAKTLGIIMGCFCLCWAPFFITNIVDPFIEYSTPMQ
LWNACLWLGYINSGLNPFLYAFLNKSFRRAFLIILCCGDHRYGRGAGYGATYTTSVNRTTVILR*

>Dr-htr4-like

MATASDSAEDSVDEVVSKHTSRITLSIVLVTIIIMTALGNLLVMVALCKDRQLRKKKTNFFIVSL
AFADLLVALVVMPLAAIELTTGKWNYGETFCLVRTSLDVLLTTASILHLCCIALDRYYAICCQPL
VYNNKMTPVRVSLMLVGCWVIPFFISFLPIMQSWNTIGIESFIEQRKLNSSRNSTCVFMVNQPYA
LVCSAVAFYVPLVLMVLAYQRIYVTAMGHARRIGSLHRAGSAPTSTYPNNDQHGSSRIKNETKAA
KTLAVIMGCFCLCWAPFFVTNVVDPFINYSVPWQMWTAWLWLGYINSGLNPFLYAFLNRAFRRAF
LMILCCGDERYARQGGFSPSRQCSESVNGTSISLRLSFLQSRRYSDNSNRFLSCELESQESAIAS

>Dr-htr4

MQGCEFPQEDLKSCHGFDPRAPHRNGPLRIDGYMNLSSTGQMPEMEEVDTNESNGLAKRVALISF
LSLVMLMSVLGNLLVMVAVCKDRQLRKIKTNYFIVSLAFADLLVSVLVMPFGAIELIHQNWIYGE
TFCLVRTSLDVLLTTASILHLCCISLDRYYAICCQPLVYRNKMTPLRVTLMIGGCWIIPTVISFL
PIMQGWNSIGIKDLIDKRKISGNSTVCVFMVNKPYALTCSVVAFYLPLVLMVLAYQRIYVTAREH
ARQISMLQRAGGAGNADSADHQRNHRMRTETKAAKTLCIIMGCFCLCWAPFFITNVVDPFIDYSV
PEQLWAACLWLGYINSMLNPILYAFLNKSFRRAFLIILCCGHKRYRRPSILGPGTTCTATQINGS
THVLKYAVLHNGNHNEQEKLSIQNDTESQESCF

>Ga-htr4

RKIKTNYFIVSLAFADLLVSVLVMPFGAIELVHQHWIYGETFCLVRTSLDVLLTTASILHLCCIA
LDRYYAICCQPLVYQNKMTPMRVALMMGGCWVIPTFISFLPIMLGWNSIGIDHLIEERRYSEGTN

STSCVFMVNKPYALTCSVVAFYIPLVLMVLAYQRIYVTARAHALQISVLQRAGGAGVGADSADHQ
RNHRMRTETKAAKTLCIIMGCFCLCWAPFFITNVVDPFIDYTVPDKLWVACLWLGYINSMLNPIL
YAFLNKSFRRAFLIILCCGRKRYRR

>Ga-htr4-like

LRKKKTNYFIVSLAFADLLVALLVMPFAAIELTTGEWRYGEIFCLVRTSLDVLLTTASILHLCCI
ALDRYYAICCQPLVYRHKMTPLRVAGMLSGCWLIPTFISFLPIMQSWNAIGIEDIIEDRRSSGGS
NDTSCVFLVNRPYALICSTVAFYVPLALMVLAYQRIYVTAMTHVRQIETLQRAGSAPVFGTTPVV
TVRSSTSSDPTDQYHLRTAASSSSSEHAPIANSRMRIETKAAKTLAVIMGCFCLCWAPFFITNVV
DPFIHYSVPWQLWTAWLWLGYINSGLNPFLYAFLNRAFRRAFLVILCCGDERYARQGSCSYGHTQ
RAYSAGSVNGTSMALR

>Lec-htr4

VPLCSCHPVSDDTSSQAEKYILAGIFSIIIAVTILGNLLVVVAVCKDRHLRKIKTNYFIVSLAVA
DLLVAILVMPFGAVERVQNDWPYGDKFCIIRTSFDVMFTTASIMHLCCIALDRFYAICCKPLVYQ
NKMTPLRVGLMLGACWVVPSLISFIPIANGWNIIGIEQQLELRLGQAKEANQTHCIFMVNKVYAV
ACSLVAFYIPLGLMAAAYQRIYVTARAHARQIGSLQRAVSKDTTGDEVTMVTSGAASPLRAQHLV
PVAQATMVQTQSTRFRHETKAAKTLAIIMGCFCLCWAPFFINNVIDPFVNYDTPPQLWDAWLWLG
YANSALNPLLYAFLNRSFRRAFLAILCCWDAARYRRSSFSGHSVRYSVSTNGSTVQPVP

>Le-htr4

FAFSARSDGKPHEIPGFSMVQKTILITFLSSIIIMTILGNLLVMVAVCKDRQLRKIKTNYFIVSL
AFADLLVSVMVMPFGAIELIQQNWIYGDTFCLVRTSLDVLLTTASILHLCCIALDRYYAICCQPL
VYRNKMTRSRVIIMLGGCWIIPSFISFLPIMQGWNSIGIQELVEIRKFQNDTNTTYCVFMVNKPY
AITCSVVAFYIPLILMVLAYQRIYVTARAHARQIEMLKRAGTSNESGQHADHHSNHRMKTETKAA
KTLCIIMGCFCLCWAPFFITNIVDPFIKYSVPDKIWTAFLWLGYINSGLNPFLYAYLNKSFRRAF
LTILCCGERYRRSSLLGQTIPCSTATINGSTHVLR

>Lo-htr4

MPVMEEADVNESTPEATPQALSSYSVVERIVLITFLSLVMLMTVLGNLLVMVAVCKDRQLRKIKT
NYFIVSLAFADLLVSVLVMPFGAIELIHQNWIYGETFCLVRTSLDVLLTTASILHLCCISLDRYY
AICCQPLVYRNKMTPMRVALMLGGCWVIPTFISFLPIMQGWNSIGINDLIEQRKFSGEANATLCV
FMVNKPYALTCSVVAFYIPLGLMVLAYQRIYVTAREHARQIGMLQRAGGANSGASDSADHQRNHR
MRTETKAAKTLCIIMGCFCLCWAPFFITNVVDPFIDYTVPEKLWTACLWLGYINSMLNPFLYAFL
NKSFRRAFLIILCCGQERYRRPSILGPSAPCTSTQINGSTHVLSGCSSASKLLLWFCNTRPVPV

>Lo-htr4-like

RKKKTNYFIVSLAFADLLVAVVVMPFAAIELSKGYWEYGETFCLVRTSLDVMLTTASILHLCCIA
LDRYYAICCQPLVYRNKMTPLRVALMLGGCWVISFFISFLPIMQSWNAIGIEKIIEERKFNNSTN
ETYCVFLVNRPYAIICSAVAFYIPLVLMVLAYQRIYITAMEHVRQIGTLQRAGSAPEAYDGSDHQ
GSSRMKIETKAAKTLAVIMGCFCLCWAPFFITNIIDPFIDYSVPWELWTAWLWLGYVNSGLNPFL
YAFLNRAFRRAFLMILCCGNERYVRPGSFGPSRHYTTSVNGTSIALRYFFKCF*

>Mm-htr4

MDKLDANVSSNEGFRSVEKVVLLTFLAVVILMAILGNLLVMVAVCRDRQLRKIKTNYFIVSLAFA
DLLVSVLVMPFGAIELVQDIWAYGEMFCLVRTSLDVLLTTASIFHLCCISLDRYYAICCQPLVYR
NKMTPLRIALMLGGCWVLPMFISFLPIMQGWNNIGIVDVIEKRKFSHNSNSTWCVFMVNKPYAIT
CSVVAFYIPFLLMVLAYYRIYVTAKEHAQQIQMLQRAGATSESRPQPADQHSTHRMRTETKAAKT
LCVIMGCFCFCWAPFFVTNIVDPFIDYTVPEQVWTAFLWLGYINSGLNPFLYAFLNKSFRRAFLI
ILCCDDERYKRPPILGQTVPCSTTTINGSTHVLRDTVECGGQWESRCHLTATSPLVAAQPSDT

>Np-htr4

MEELDSNVRPHEGFGVGEKIVLITFISAVILMTTLGNLLVMVAVCRDRQLRKIKTNYFIVSLAFA
DLLVSVLVMPFGAIELIQDKWIYGEMFCLVRTSLDVLLTTASILHLCCISLDRYYAICCQPLVYR
NKMTPLRIALMLGGCWIIPTFISFLPIMQGWNSIGILDLIEKRKFSKDSNSTYCIFMVNKPYAIT
CSVVAFYIPLFLMVLAYYRIYVTAREHARQIGVLQRAGAPADHRHQHPDQHTTHRMKTETKAAKT
LCIIMGCFCLCWAPFFITNVIDPFIDYSVPGQLWTAFLWLGYINSGFNPFLYAFLNKSFRRAFLI
ILCCGDEKYRRPSILGQTVPCSTTTINGSTHVLRYTVLYNGHHQEQDKLPIQIDPESQESCF

>Pm-htr4

MYGNNTTFNNDETSSQAEKYILAGIFSIIIAVTILGNLLVVVAVCKDRHLRKIKTNYFIVSLAVA
DLLVAILVMPFGAVERVQNDWPYGDKFCIIRTSFDVMFTTASIMHLCCIALDRFYAICCKPLVYQ
NKMTPLRVGLMLGACWVVPSLISFIPIANGWNIIGIEQQLELRLGQAKEANQTHCIFMVNKVYAV
ACSLVAFYIPLGLMAAAYQRIYVTARAHARQIGSLQRAVSKDTTGDEVTMVTSGAASPLRAQQLV
PVAQATMVQTQSTRFRHETKAAKTLAIIMGCFCLCWAPFFINNVIDPFVNYDTPQQLWDAWLWLG
YANSALNPLLYAFLNRSFRRAFLAILCCWDAARYRRSSFSSHSVRYSVSTNGSAVQPVPSARVRA
RARLPSRPRTRAAACPAVSKLVTHFCGAPSPV

>Rt-htr4

MDEADVNESTSDGEPHEIPGFSILQKTVLITFLSAIMVMTILGNLLVMVAVCKDRQLRKIKTNYF
IVSLAFADLLVSVMVMPFGAIELIQQNWIYGDRFCLVRTSLDVLLTTASILHLCCISLDRYYAIC
CQPLVYRNKMTPLRITIMLGGCWIIPSFISFLPIMQGWNIIGIQELVEERKSSNDTNITKCVFMV
NKPFAITCSVVAFYVPLLLMVLAYQRIYVTAREHARQIEMLKRAGTSTDSGQQADHSGNHRMKTE
TKAAKTLCIIMGCFCLCWAPFFITNIVDPFINYSVPEQLWTACLWLGYINSGFNPFLYAYLNKSF
RRAFLRILCCGDRYRRSSLLGQTIPCSATTINGSAHVLR

>Xt-htr4 _

MAHNINVTSNEGYGVAARIVLISFISAVILMTILGNLLVMVAVCRDRQLRKIKTNYFIVSLAFAD
LLVSVLVMPFGAIELVQEKWIYGEMFCLVRTSLDVLLTTASILHLCCISLDRYYAICCQPLVYRN
KMTPLRITLMLSGCWIIPTFISFLPIMQGWNSIGILDLIETRKYNKSSNSTNCIFMVNKPYAITC
SVVAFYIPFFLMVLAYYRIYITAREHARQIGVLQRAGAPADHRHQHPDQHTTHRMKTETKAAKTL
CIIMGCFCLCWAPFFITNVVDPFINYSVPVELWTAFLWLGYINSGLNPFLYAFLNKSFRRAFLII
LCCGDEKYRRPSILGQTVPCSTTTINGSTHVLRYTVLYNGHQQEHDTLPIHIDPESQESCF

>Xl-htr4.S _

MLCSEKSLVSMDICIYFIGPIFYRSNEGYGVAARIVLISFISAVILMTILGNLLVMVAVCRDRQL
RKIKTNYFIVSLAFADLLVSVLVMPFGAIELVQEKWIYGEMFCLVRTSLDVLLTTASILHLCCIS

LDRYYAICCQPLVYRNKMTPLRIALMLSGCWIIPTFISFLPIMQGWNSIGILDLIETRKYNKSSN
STNCIFMVNKPYAITCSVVAFYIPFLLMVLAYYRIYITAREHARQIGVLQRAGAPVDHRHQHPDQ
HTTHRMKTETKAAKTLCIIMGCFCLCWAPFFITNVVDPFINYSVPVELWTAFLWLGYINSGLNPF
LYAFLNKSFRRAFLIILCCGDEKYRRPSILGQTVPCSTTTINGSTHVLRYTVLYNGHHQEHDTLP
IHIDPESQESCF

>Mm-htr5A

MDLPVNLTSFSLSTPSSLEPNRSLDTEVLRPSRPFLSAFRVLVLTLLGFLAAATFTWNLLVLATI
LKVRTFHRVPHNLVASMAISDVLVAVLVMPLSLVHELSGRRWQLGRRLCQLWIACDVLCCTASIW
NVTAIALDRYWSITRHLEYTLRTRKRVSNVMILLTWALSTVISLAPLLFGWGETYSEPSEECQVS
REPSYTVFSTVGAFYLPLCVVLFVYWKIYRAAKFRMGSRKTNSVSPVPEAVEVKNATQHPQMVFT
VRHATVTFQTEGDTWREQKEQRAALMVGILIGVFVLCWFPFFVTELISPLCSWDVPAIWKSIFLW
LGYSNSFFNPLIYTAFNRSYSSAFKVFFSKQQ

>Dr-htr5ab

MTQPNVTALSANISGGSESGNLYRPFSVFSVLTLTLLAMLVVATFVWNLLVLVTILRVRTFHRVP
HNLVASMAISDVMVAGLVMPLSLVRELYGRRWILGRALCQVWISCDVLCCTASIWNVTAIALDRY
WSITRHLEYTLKTRKRISNVMIGLTWLLSSVISLSPLFGWGETYSEDSLACQVSQEPSYTVFSTF
GAFYLPLCVVLFVYWKIYKAAKFRIGSRKTNTITPMAEVIEVKEAERQPQMAFTVRHATVSFQTD
GETWREQKERRAALMVGILIGVFVLCWIPFFLAELIIPLCSCDIPPVWKSVFLWLGYSNSFFNPL
IYTAFNKNYNNAFRNLFSRQR

>Lec-htr5

MDGNRHSDDLSPNMSTWPMNGSDPWPDHNVSDQPQPFSFFSILTFTLLAILILATFAWNVLVIVT
ILRVKTFHCVPHNLVASMAVSDVLVAALVMPLSLVSELHRRRWRLGPVMCDVWISCDVLCCTASI
WNVTAVALDRFWSITRHLEYTIRVRKCISNLMIALTWLLSATISLSPLFGWGEGYYSDKSQHCHF
SQGPLYTVFSTVGAFYLPLCVVLFVYWKIYKATKFCFGSHKANSVMPISDEIEVRR

>csa-htr5

ALAILLVVIIVGSIFGNILVVAAVKSERNLQTTANFLICSLAITDFMVACLVMPFSALYEILGTW
VFGDILCQFWTAIDIACCTASILHLCAIAFERHRSITGAVRYFSQGRRRTRTQTTVGRVRFVDQG
NIACYPSGSNAVNGGIVPLVKRTQDTLQYRRIPSFEAQQKSTPRIAGIFNCCHKYSDRKQQRRLV
DQRRSSIMLAHSLSLRAKIKLLKTREVRAIKTLGTIVGAFVICWLPFFAVTLAAAFCDCEIPRSL
RSIVLWLGYCNSLVNPILYGAFNRDFHSAFKKLLTSHFYKIRCR*

>Cin-htr5

ALAILLAIIIIGSMFGNILVVIAVRSERNLQTTANFLICSLAITDFLVACLVMPFSALYEISGTW
VFGDILCQTWTAIDIACCTASILHLCAIAFERHRSITSAVRYFSQGRRHTVAPKIVLVWVLAICI
SIPPILGWKPSSQQQTQVNNVTTAASTVQRCEVGHYREYTLYATLGSFYIPLALLLTAYVRIYRR
LLNQRRSSIMLAHSLSLRAKVKLLKAREVRAIKTLGTIVGAFVICWLPFFAVTLAAAFCNCKMPH
TLTSIVLWLGYCNSLVNPILYGAFNRDFYAAFKKL

>Dr-htr6

MMSGSPPIPRVEAASDLWKIHRSFNDSSTISDGWSIGGSGPWLLAVMLSLIILVTACGNILLIAL
VFAHRSLRCTSNCFLVSLFLSDLMVALVVMPPAMLNVLCGTWVLAPGFCPVWLCFDVMCCSASIL

NLCVISLDRYLLIISPLRYKQHMTPPRALLLVGGAWGLAALTSFLPIKMDWHSLGRMQELTEDDP
GNATHLNSFYPSSYFQLSSSGMPSTQCRLRVTLPFALVATFLTFFLPSTAICFTYCRILLAARRQ
ARQVEALTHPAYPQHSLGEPSRPPSPGHAIQDGDDYSHQEPPGLRHAPLSVNSERRLAHRQRKRA
LKASLTLGVLLGLFFSAWLPFFITNMAQAVCECVPPSFFDAITWLGYCNSTMNPIIYPMFMRDFK
RALARLLPCCSSSQAPRRPSLPLSLSLRNSGEPQLPSEPPSLVSDPPQLPATATDAVNLLDAEHA
GIDLPLLLPNQVDTLDD

>Mm-htr6

MVPEPGPVNSSTPAWGPGPPPAPGGSGWVAAALCVVIVLTAAANSLLIALICTQPALRNTSNFFL
VSLFTSDLMVGLVVMPPAMLNALYGRWVLARGLCLLWTAFDVMCCSASILNLCLISLDRYLLILS
PLRYKLRMTAPRALALILGAWSLAALASFLPLLLGWHELGKARTSAPGQCRLLASLPYVLVASGV
TFFLPSGAICFTYCRILLAARKQAVQVASLTTGTATAGQALETLQVPRTPRPGMESADSRRLTTK
HSRKALKASLTLGILLSMFFVTWLPFFVASIAQAVCDCISPGLFDVLTWLGYCNSTMNPIIYPLF
MRDFKRALGRFVPCVHCPPEHRASPASPSMWTSHSGARPGLSLQQVLPLPLPPNSDSDSASGGTS
GLQLTAQLLLPGEATRDPPPPTRAPTVVNFFVTDSVEPEIRQHPLGSPMN*

>Rt-htr6

MDNGLWAEGKGPELGQGAAPEGRQWIAAFLCLIIILTTAGNSLLILLIFTQRSLRNTSNYFLVSL
FMSDLMVGLVVMPPAMLNELYGRWVLESELCSIWYSFDVMCCSASILNLCVISLDRYFLIISPLK
YKLRMTSCRALFLIFTTWTLAALASFLPIEMGWHEMDFDAQALNSTLELPEEAAQCRFLVSLPYA
LIASSLTFFLPSVAISFTYCRILLAAKKQAVQVASLTTNVATTSDEPVQVKLPLSQNPSARTSDS
RKFTKHSKKALKASLTLGVLLGMFFVAWLPFFVANVTQAVCECVPALLFDVLTWLGYCNSTMNPI
IYPLFMRDFKRAMAKYLPCCHRWWERRPTVVSLSMKNSNSGRRLGLSLPNMLTLPPQTGSGDSVI
QVNEHILLAASKDQQLTKDQQLEDSLQLFELEQTDQEFQGNPLNTPMD*

>Mm-htr7

MMDVNSSGRPDLYGHLRSLILPEVGRRLQDLSPDGGAHSVVSSWMPHLLSGFPEVTASPAPTWDA
PPDNVSGCGEQINYGRVEKVVIGSILTLITLLTIAGNCLVVISVCFVKKLRQPSNYLIVSLALAD
LSVAVAVMPFVSVTDLIGGKWIFGHFFCNVFIAMDVMCCTASIMTLCVISIDRYLGITRPLTYPV
RQNGKCMAKMILSVWLLSASITLPPLFGWAQNVNDDKVCLISQDFGYTIYSTAVAFYIPMSVMLF
MYYQIYKAARKSAAKHKFSGFPRVQPESVISLNGVVKLQKEVEECANLSRLLKHERKNISIFKRE
QKAATTLGIIVGAFTVCWLPFFLLSTARPFICGTSCSCIPLWVERTCLWLGYANSLINPFIYAFF
NRDLRTTYRSLLQCQYRNINRKLSAAGMHEALKLAERPERSEFVLQNCDHCGKKGHDT

>Dr-htr7

MISALTKDRVQSIVNSLTSEVMDVSFLNKIHDTTTTLPTPSFMDNGTRCGVPILSYGHVEKVLIG
GVLTMLTFLTVCGNLLVVISVCFVKKLKQPSNYLIVSLAVADLSVAVVVMPFVSITDLIGGQWIF
GRVFCNVFIAMDVMCCTASIMTLCVISIDRYLGITKPLTYPVRQSGKCMAKIVLSVWLLSASITL
PPLFGWAQNVNDDNVCLISQDLGYTIYSTAVAFYIPMSVMLIMYYRIYRVAKDSAAKHTIAGFPK
AEDEESMNCVTAALKLQREVEECVSFSRLLKSDRKNISIFKREQKAAATLGIVVGAFAVCWMPFF
LLSTARPFICGLQCSCVPLWVERTLLWLGYANSLINPFIYAFFNRDLRTTYHNLIRCRYRNINRK
LSAASMHEALKLAERPDLVL

>Cin-htr7

MSNLSFCPEDGNTTSAEGDAMLTTALIAALTSLLSLLIVVGNGLIIVSVALVKKLRQPANYLIVS
LALSDFLVGLVVLPLTIVYDIMGEWVFGPNVCDVHVSFDVICCTASIMNLCMISIDRYLMITQPM
TYPKRRTGKLMLLLIATAWVLSCLVIIPALFGFTKNVKDGKVHSFAINQTAGYENPMLGEFQVFG
IKKYGKACLISQERWFTIYSTLGAFYLPLAVMLCMYWKIYLEASRFNARHRLRSYSTTGSQDSTN
GNAVNLIRSSSGVVCGLGMKNGFPRNGTTRHNGMEWRFPTISEVDILTSLNEEGEQTNKETETET
INGKINEEYRARTLYTRASANSLLATEKTPKLVGKPSPRSRLFMLYGKPRLLRATSTPCPTTTNK
PNTQHTRKSSFRTLRQNSEITFPCNRRPTVFNQIRRRVSLATSRDRMRNVKATRTLGIVVGAFTF
CWLPFFIVTFLRPFACPIPESQDCIPLWLVRFVLWLGYLNSALNPLIYIGFSPDLRETFRFLICC
KCTNVDRRLAQIELRQAIAVERKASMASRSYAPESIV*

>csa-htr7

ALIAALTSLLSLLIVVGNGLIIVSVALVKKLRQPANYLIVSLALSDFLVGLVVLPLTIVYDIMGE
WVFGPNVCDVHVSFDVICCTASIMNLCMISIDRYLMITQPMTYPKRRTGKLMLLLIATAWLLSCL
VIIPALFGFTKNVKDGKRAIFEINKTAGFENPSLGEFQVFGIKKYGKTCLISQERWFTIYSTLGA
FYLPLAVMLCMYWKIYLEASRFNSRHRLRSYSTSGSQRANGVNGVELIRCESGIALEERNGASRH
QRANGVEWRFPTISKVNILTSLDENGEETIKRTITSPCDAQQQNGKFFSEYQARTLYTRASAQSL
IAAEQSLISSGKNGKIPDKTHSRSKLSLLYGQARCCRPRLLRTTSSPAQSPSNGSKLSSANVRKA
SFRTVRQNSEITFPCQRRPTVFNQIRRRVSLATSRDRMRNVKATRTLGIVVGAFTFCWLPFFIVT
FLRPFACPIPEAQDCIPLWLVRFVLWLGYLNSALNPLIYIGFSPDLRETFRFLICCKCTNVDRRL
AQIELRQAIAHERRNSVASRSYAAESIV

Unclassified aminergic receptors

>Cin-AmR

MIMESTNLVTALPEQNMSLFEVSMSTNTNSTYEPPFDSIGIAISAVIIFTFLVLGVVGNSLTLAV
ILTYKELNNNLFMRFIFSLCISDLLSALISWLFLYRRTWGFDVWDPIPAVFCKFYWATDIMTSYA
TALHVLSFAIVRFISVQYPVQYNKMKLIHANIWIVGIWVACIISGFIPSMFIFGAKARDRESSSP
DSRWPSCTANLADLDKYILYQKVAYPLFLYIPTIGVVITCILIAVTLKKRSGSANLNKKEERRLR
KERQAVLQLILIIISFLLGYIPFTAYEFWGANTHPRERYYFLVDNWFGMIEYFCLRFSECLNPVF
YNLGSTKMRTYTKMFLKKKVFCCIEWQPTTPMSPTSNTRGNKSGVNTIANSVDATLENTNHHNV

>Csa-AmR1

MANLALAVIYFTVFFFGIIGNAIVLFVLLRQRRGWSVTTAYLFNLALSDVLFLCVLPFWGHQYLN
GLDWAFGLGWCKIVGSVTSINMYASVFFLTAMSADRYMAVVHATSVNVVRSSYIARWVCVAVWFA
ALLLSLPQLIHQTLHPIFYVQTNSTNASQVSTPGESPMLRDVTSAMAEAPRSYAAPESPIACTFY
IPAGSSKALRMGLVEFTRSMLGFVVPMIVISVCYAKIVITVKKKVIGKRVRKDRVAKLAALVVLA
FFFCWLPMQIMKLFSALGGWWKLRVFEFDEDLYNTVYPFMVALAYSNSCINPVVYAFTTTNFQEN
IKDICGSDKAARPYKMTLSPQQGHNGDPKTGFVTKTEALNMYSPCPKRNMTAYSPAVQVHAAEVS
TIAQYIELFCRSITPVYVRPVSLVVKCVAF*

>Csa-AmR2

MREILTNIFSLTTLSLREMNSSILNQVLNEKNECKLLSVIYTQSDLQGVPEFLMNCTSIERLDIS
SNLISRILEHDFRRLTELKHLNLHNNRLQRIERGSFSSLVKLETVDISENKDVLYDNSLFSSQSK
LINVLGNDFIICCLARKTSSTSLRFCSPVDAISSCERLIAKDYIRALIWIQALIAIIGNVLVAIL
RAKEYPKQESRGGKLNSSFVGVLSIADLLMGLFLLIIAIVDSVYRVEFYKIAKTWRLDSALCRFC
GFLSLFSAEASVFTLLVITFTRMVSIVSPIYYLRANLHIFRFAMLIAWTVALSISVVPLFRFRTF
FSKTSLCLPAFYSSTSDDDWIYSIFVIILNLVAFLAMMTSYLVIIVATRRSQTRSEERRSSNPRP
KAKLGKRVLLIVATDFFCWMPVCMLGFVSIASDGLNFYNDIYPWTAVILLPINSSMNPLIYTFLT
RGTICQTITQMRSESKSGSRTASSTAK*

Deuterostome serotonergic receptor 4

>alu-htr4

MTTIVTMSGYNTTQALGIPGTDLTRTVQPVTNWTNGSLGCTETYIGPQYSLTV**QV**LLSSFLALVI
LLTIVGNILVISTVALFRSLRSLTNYF**VV**SLAVADLLVGIVMTLGTQYEVSGRWRHGEIFCLVF
TSMDVSLTTCSIMHLSCIAFDRYYAICHPFKYPTKMTHRKMRVMIAMCWVVPVIIAWVPIMNGWN
TLNIAxxxxxxxxxxxxxxxxxxxxxxxxxxxxxxxxxxxxxxAVLMILAYYQVYRVARRQMQRI
ISVEGAAPNNENTRREKKAAKTLGIIMGCFLVCWMPFFVTNIIDPLCGYCIAQSCELCLKFFLWL
GYINSTLNPMIYAFFNRSFRRAFKRILSCSSCCDLDKRDFDPAVSTALQGRTRSRRRPNGDVVNN
S*

>Bbe-htr4

MTTIVSMSDYNTTQALLVPETHDVRTVQPGTNDTNGSSGCTEAYLGSQYSLVI**QI**LLSCFLALVI
LLTIVGNILVISTVALFRSLRSLTNYF**VV**SLAVADLLVGIVMTLGTQYEVSGRWRHGEIFCLVF
TSMDISLTTCSIMHLSCIAFDRYYAICHPFKYPTKMSHRK**MV**LMIALCWIVPVIMAYVPIMNRWN
TIGIEPVVEDTKCALGPDSCVFLVNKEFSLIASTLSFYLPAVMMTLAYYQVYRAARRQMQQIISL
ERAMNNNENMRREKKAAKTLGIIMGCFLVCWTPFFVANIVEPLCGYCITNSCELCLKFFLWLGYI
NSTLNPMIYAFFNRSFRRAFKRILSCWSCCDVDNRDFDPAVSTALQTRTSRRHQNGEVVNNT*

>Bfl-htr4

MTTIVSMSDYNTTQALAVPETHHVRTVQPGANGTNGSSGCTEAYLGPQYSLEI**QV**LLSSFLALVI
LLTIVGNILVISTVALFRSLRSLTNYF**VV**SLAVADLLVGIVMTLGTQYEVSGRWRHGEIFCLIF
TSMDISLTTCSIMHLSCIAFDRYYAICHPFKYPTKMSHRKMVLMIAMCWVVPVIMAYVPIMNRWN
TIGIEPVVEDTKCALGPESCVFLVNKEFSLIASTLSFYLPAVMMTLAYYQVYRAARRQMQQIISL
ERSMNNNENMRREKKAAKTLGIIMGCFLVCWMPFFVANIVEPLCGYCITRSCDLCLKFFLWLGYI
NSTLNPMIYAFFNRSFRRAFKRILSCSSCCDLDNRDFDPAVSTALQRRTSTRHQNGEAVNNT*

>Bla-htr4

MTTIVSMSDFNTTQALVVPETHPLRTAPPVTNHTNGSTGCTESYLGPQYSLVI**QI**LLSCFLALVI
LLTIVGNILVISTVALFRSLRSLTNYF**VV**SLAVADLLVGIVMTLGTQYEVSGRWRHGEIFCLVF
TSMDISLTTCSIMHLSCIAFDRYYAICHPFKYPTKMSHRKMVLMIAMCWVVPVIMAYVPIMNRWN
TIGIEPVVEDTKCELGPDSCVFLVNKEFSLIASTLSFYLPAIMMTLAYYQVYRAARRQMQQIISL
ERAMNNNENMRREKKAAKTLGIIMGCFLVCWMPFFVANIVEPLCGYCITRSCALCSKFFLWLGYI
NSTLNPMIYAFFNRSFRRAFKRILSCSSCCDVDNRDFDPAVSTALQGRSSRRHQNGEVVNNT*

>Ptfl-htr4

MSTNNIHGLPYVFESSDTPATSAVFVVELETTRQPFLITNDNASTWAVSGGVQPGSFNVAERIVI
GFILSLIILTTIVGNILVCTSIIIFPSLRTLTNYFVFSLAIADVLVACLVMAISTIYIVTETWMY
GDTFCLVFISFDIAFCTASIMHLSCIAYDRYSAICNPFQYPIKMTSRRVAVMLAACWVVPMLISF
IPILLGWNTLGIENVIAEVKQHTGPNSCVFLVNRPYAAVASTIAFYIPCILMASAYLFIFRAARK
QAAQIKSLERATANWNNARNGESGGRGSVSLAAEKKAAKTLGIIMGCFTVCWCPFFVLNIIDPFC
DFCISPKVWPPITWLGYTNSMLNPILYAFFNRTFRRAFSRLLRCHVCKGSAAEFDPTITSHERRS
*

>Sako-htr4

MESGITSSTDAISYHSFALDKTSRVYVLEVFTDTGEPIVGAVNDNSTGLSGNVLQYGLAERIIIG
FVLTVIIILTIAGNILVCASIFIFPSLRTLTNYFVFSLAIADILVACLVMTFSTINVVTETWIYG
DTFCLIFISFDISFCTASIMHLSCIAFDRYSAICNPFQYPLKMTSRRVTIMLASCWVVPMFISFI
PILLQWNTIGIENVIADVKNFAGPYSCIFLVNKPYAVIASTIAFYMPCILMATAYFFIFRAARKQ
AQQIKSLERATMNWNNTRNGPGDGHPHHHGRRVSLAAEKKAAKTLGIIMGCFTICWCPFFVFNIV
DPFCDFCIDPGAWPPVTWLGYINSMLNPLLYAFFNRSFRRAFIRLLRCHVCKGTTAEFDPTISSR
ER*

SUPPLEMENTARY INFORMATION
SI Figure 1: Phylogenetic tree for htr4 indeuterostomes and inferred gene gain/loss events

Olfactory function in the trace amine-associated receptor family (TAARs) evolved twice independently

Milan Dieris[1], Daniel Kowatschew[1], and Sigrun I. Korsching[1]*

[1]Institute for Genetics, University at Cologne, Germany
*Corresponding author, sigrun.korsching@uni-koeln.de

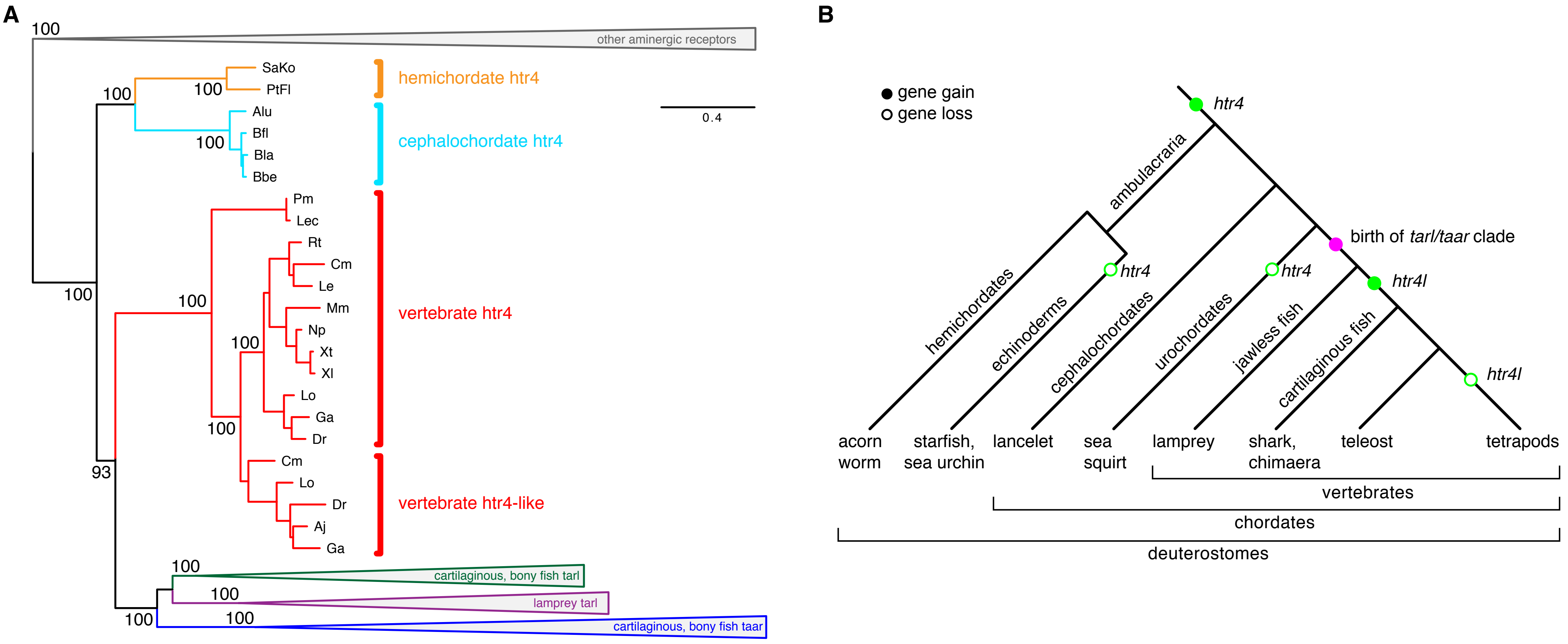

SI Figure 1 Phylogenetic tree for HTR4 in deuterostomes and inferred gene gain/loss events

A) A phylogenetic tree of deuterostome *htr4* genes was constructed using MAFFT for alignment and maximum likelihood algoritm PhyML-aLRT. For details see Methods. Representative *taar* and *tarl* genes are added as reference. Clades are collapsed as indicated.

B) A deuterostome species tree with predicted gene gain and loss events for *htr4* and the birth of the *taar/tarl* clade.

SUPPLEMENTARY INFORMATION
SI Figure 2: No correlation between genomic coverage and detection of *taar* genes

Olfactory function in the trace amine-associated receptor family (TAARs) evolved twice independently

Milan Dieris[1], Daniel Kowatschew[1], and Sigrun I. Korsching[1]*

[1]Institute for Genetics, University at Cologne, Germany
*Corresponding author, sigrun.korsching@uni-koeln.de

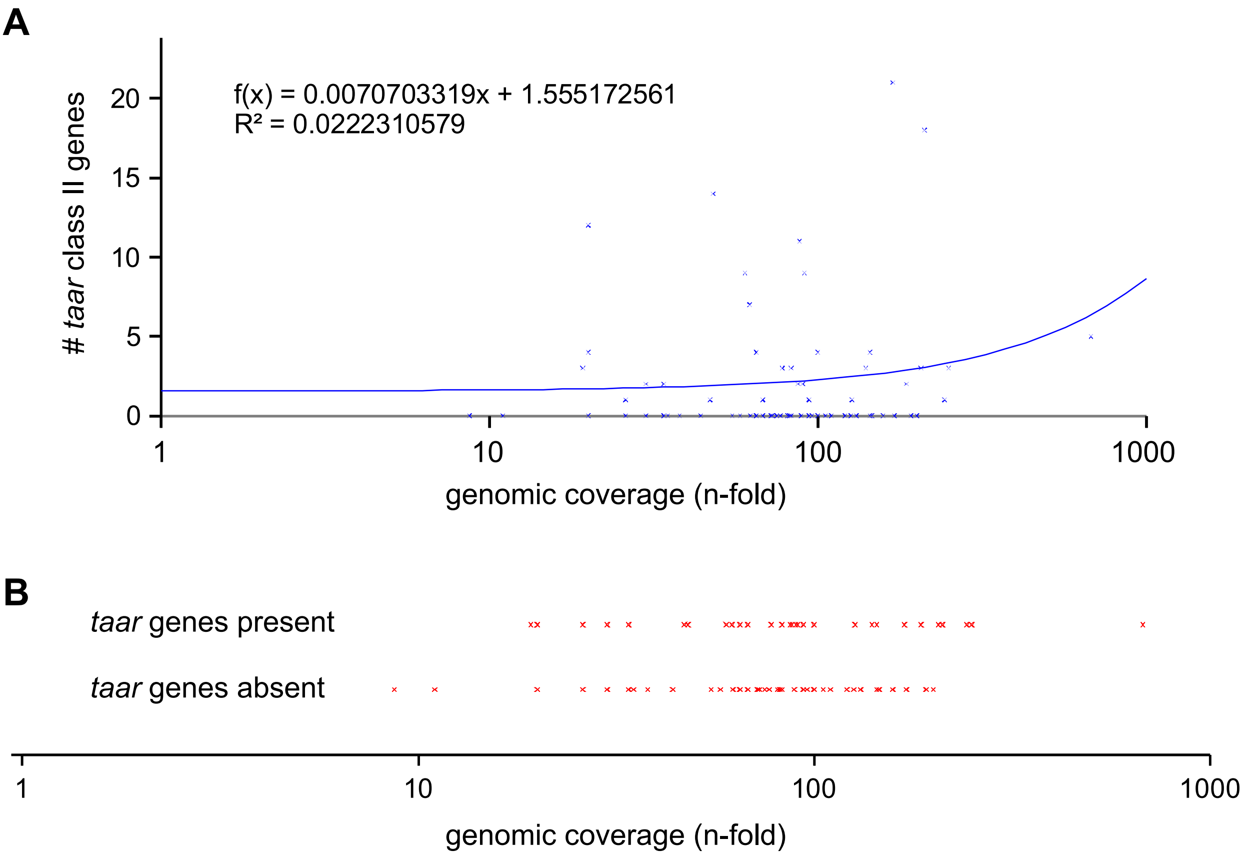

SI Figure 2 No correlation between genomic coverage and detection of taar genes
A) Number of *taar* genes is shown for 69 cartilaginous and bony fish species. Each blue cross represents one species. Linear regression and correlation coefficient R as indicated.
B) Presence respectively absence of *taar* genes is shown for 69 cartilaginous and bony fish species. Each red cross represents one species.

SUPPLEMENTARY INFORMATION
SI Table 1: Genomic location of all genes analysed

Olfactory function in the trace amine-associated receptor family (TAARs) evolved twice independently

Milan Dieris[1], Daniel Kowatschew[1], and Sigrun I. Korsching[1]*

[1]Institute for Genetics, University at Cologne, Germany
*Corresponding author, sigrun.korsching@uni-koeln.de

SUPPLEMENTARY INFORMATION
Table of Contents for SI Table 1: Genomic location of all genes analysed

Olfactory function in the trace amine-associated receptor family (TAARs) evolved twice independently

Milan Dieris[1], Daniel Kowatschew[1], and Sigrun I. Korsching[1]*

[1]Institute for Genetics, University at Cologne, Germany
*Corresponding author, sigrun.korsching@uni-koeln.de

SI table 1 consists of seven sheets:

Sheet 1 Overview: Presence of *htr4, taar* and *tarl* genes in non-vertebrate deuterostomes *htr4* genes in chordates and hemichordates

Sheet 2 Overview: Number of *taar* and *tarl* genes in aquatic vertebrates

Sheet 3 *tarl* genes of jawless vertebrates identified in this study

Sheet 4 *tarl* genes of jawed vertebrates identified in this study

Sheet 5 *taar* genes of jawed vertebrates identified in this study

Sheet 6 Representative reference TAAR sequences

Sheet 7 Aminergic receptors used as outgroup in phylogenetic analysis

Overview: Presence of *htr4*, *taar* and *tarl* genes in non-vertebrate deuterostomes (chordates, hemichordates and echinoderms)

Belongs to taxonomical group	Abbre-viation	Latin species name	Vernacular name	htr4	taar	tarl
echinoderm	AcPl	*Acanthaster planci*	Crown-of-thorns starfish	-	-	-
cephalochordate	Alu	*Asymmetron lucayanum*	Sharptail lancelet	+	-	-
echinoderm	ApPa	*Apostichopus parvimensis*	Warty sea cucumber	-	-	-
echinoderm	ApJa	*Apostichopus japonicus*	Japanese spiky sea cucumber	-	-	-
cephalochordate	Bbe	*Branchiostoma belcheri*	Chinese amphioxus	+	-	-
cephalochordate	Bfl	*Branchiostoma floridae*	Florida lancelet	+	-	-
cephalochordate	Bla	*Branchiostoma lanceolatum*	European lancelet	+	-	-
urochordate	Cin	*Ciona intestinalis*	Vase tunicate	-	-	-
urochordate	Csa	*Ciona savignyi*	Pacific transparent seq squirt	-	-	-
echinoderm	EuTr	*Eucidaris tribuloides*	Slate pencil urchin	-	-	-
echinoderm	HePu	*Hemicentrotus pulcherrimus*	a sea urchin	-	-	-
echinoderm	LyVa	*Lytechinus variegatus*	Green sea urchin	-	-	-
echinoderm	OpFa	*Ophionereis fasciata*	Mottled brittle star	-	-	-
echinoderm	OpSp	*Ophiothrix spiculata*	Spiny brittle star	-	-	-
echinoderm	PaMi	*Patiria miniata*	Bat star	-	-	-
echinoderm	PaRe	*Patiriella regularis*	Common cushion star	-	-	-
hemichordate	PtFl	*Ptychodera flava*	Hawaiian acorn worm	+	-	-
hemichordate	SaKo	*Saccoglossus kowalevskii*	Acorn worm	+	-	-
echinoderm	StPu	*Strongylocentrotus purpuratus*	Purple sea urchin	-	-	-

***htr4* genes in cephalochordates and hemichordates**

	Abbre-viation	Latin species name	Vernacular name	Gene name	Accession	length (full, partial)	Pseudo-gene (Ψ)	# exons	Database incl. Version number	chr	contig	location in contig (bp)	Strand (+/-)	Comments
**Cephalochordates**														
	Alu	*Asymmetron lucayanum*	Sharptail lancelet	*htr4*	-	partial	-	3	GCA_001663935.1	-	LZCU01069842.1, LZCU01138971.1	2645-4248, 3-464	+, +	2nd exon in two contigs, 35aa missing in between
	Bbe	*Branchiostoma belcheri*	Chinese amphioxus	*htr4*	GeneID:109485254	full	-	4	GCF_001625305.1	-	NW_017804398.1	521476-519001	-	annotated as 5-hydroxytryptamine receptor 4-like
	Bfl	*Branchiostoma floridae*	Florida lancelet	*htr4*	GeneID:118414956	full	-	3	GCF_000003815.2	4	NC_049982.1	14530149-14532452	+	annotated as 5-hydroxytryptamine receptor 4-like
	Bla	*Branchiostoma lanceolatum*	European lancelet	*htr4*	-	full	-	3	GCA_900088365.1	-	FLLO01000036.1	925959-928070	+	
**Hemichordates**														
	PtFl	*Ptychodera flava*	Hawaiian acorn worm	*htr4*	-	full	-	1	GCA_001465055.1	-	LD352455.1	57543-56371	-	
	SaKo	*Saccoglossus kowalevskii*	Acorn worm	*htr4*	GeneID:100374682	full	-	1	GCF_000003605.2	-	NW_003129357.1	24544-25722	+	annotated as 5-hydroxytryptamine receptor 4-like

Overview: Number of *taar* and *tarl* genes in aquatic vertebrates

				Class II taar genes					
Abbre-viation	Latin species name	Vernacular name	*tarl*	*taar12*	*taar13*	Other class II *taars*	genome	fold coverage	Database for species without *taar* and *tarl* genes
Aa	*Anguilla anguilla*	European eel	1	7	5	0	full	20.0x	
Ac	*Amphilophus citrinellus*	Midas cichlid	1	0	0	0	full	89.0x	
Af	*Anoplopoma fimbria*	Sablefish	1	0	2	0	full	186.0x	
Aj	*Anguilla japonica*	Japanese eel	2	9	9	0	full	211.0x	
Al	*Austrofundulus limnaeus*	Järvikilli	1	0	0	0	full	94.0x	
Am	*Astyanax mexicanus*	Mexican cavefish	1	3	1	0	full	65x	
Ar	*Anguilla rostrata*	American eel	2	13	8	0	full	169x	
Bp	*Boleophthalmus pectinirostris*	Giant blue-spotted mudskipper	1	0	0	0	full	144x	
Cc	*Cyprinus carpio*	Common carp	2	15	5	0	full	n/a	
Ch	*Clupea harengus*	Herring	1	0	1	0	full	127x	
Cm	*Callorhinchus milii*	Elephant shark	1	0	0	3	full	19.25x	
Cs	*Cynoglossus semilaevis*	Tongue sole	1	0	0	0	full	117x/94x	
Cn	*Cyprinodon nevadensis*	Amargosa pupfish	1	0	0	0	full	26.0x	
Cr	*Cottus rhenanus*	Rheingroppe	1	0	0	0	full	11.0x	
Cv	*Cyprinodon variegatus*	Sheepshead pupfish	1	0	0	0	full	81x	
Dr	*Danio rerio*	Zebrafish	1	12	5	0	full	n/a	
Dl	*Dicentrarchus labrax*	European seebass	1	0	2	0	full	30x	
El	*Esox Lucius*	Northern pike	2	0	1	0	full	243.0x	
Fh	*Fundulus heteroclitus*	Mummichog	1	0	0	0	full	75x	
Gm	*Gadus morhua*	Atlantic cod	2	0	3	0	full	83x	
Ga	*Gasterosteus aculeatus*	Three-spined stickleback	1	0	1	0	full	n/a	
Hb	*Haplochromis burtoni*	Burton's mouth-breeder	1	0	0	0	full	131x	
Hc	*Hippocampus comes*	Tiger tail seahorse	1	0	0	0	full	192x	
Ip	*Ictalurus punctatus*	Channel catfish	1	2	8	0	full	n/a	
Km	*Kryptolebias marmoratus*	Mangrove killifish	1	0	0	0	full	72.75x	
Lb	*Labrus bergylta*	Ballan wrasse	1	0	4	0	full	144x	
Lc	*Larimichthys crocea*	Yellow croaker	1	0	3	0	full	140x	
Lac	*Lates calcarifer*	Asian sea bass	2	0	2	0	full	90.0x	
Lo	*Lepisosteus oculatus*	Spotted gar	1	3	1	1 *taar5*-like	full	677.5x	
Lec	*Lethenteron camtschaticum*	Arctic lamprey	51	0	0	0	full	20.0x	
Lw	*Leuciscus waleckii*	Amur ide	1	1	2	0	full	250x	
Le	*Leucoraja erinacea*	Little skate	1	0	0	1	full	26.x	
Mz	*Maylandia zebra*	Zebra mbuna	1	0	0	0	full	65x	
Mc	*Mchenga conophoros*	Happy	1	0	0	0	full	n/a	
Mim	*Miichthys miiuy*	Mi-iuy croaker	1	0	0	0	full	158x	
Mom	*Mola mola*	Ocean sunfish	1	0	0	0	full	96x	
Moa	*Monopterus albus*	Asian swamp eel	1	0	0	0	full	146x	
Ms	*Morone saxatilis*	Striped bass	1	0	1	0	full	68.0x	
Nb	*Neolamprologus brichardi*	Lyretail cichlid	1	0	0	0	full	171x	
Nf	*Nothobranchius furzeri*	Turquoise killifish	1	0	0	0	full	100x	
Nc	*Notothenia coriiceps*	Black rockcod	0	0	0	0	full	38x	GCF_000735185.1
On	*Oreochromis niloticus*	Nile tilapia	1	0	0	0	full	44.0x	
Ol	*Oryzias latipes*	Medaka	1	0	0	0	full	99%	
Po	*Paralichthys olivaceus*	Bastard halibut, japanese flounder	1	0	0	0	full	100.0x	
Pa	*Pampus argenteus*	Silver pomfret	1	0	0	0	full	58.0x	
Ps	*Periophthalmodon schlosseri*	Giant mudskipper	1	0	0	0	full	65x	
Pem	*Periophthalmus magnuspinnatus*	Giant-fin mudskipper	1	0	0	0	full	77x	
Pm	*Petromyzon marinus*	Marine lamprey	32	0	0	0	full	62.36x	
Pp	*Pimephales promelas*	Fathead minnow	1	5	2	0	full	62.0x	
Pf	*Poecilia Formosa*	Amazon molly	1	0	0	0	full	82x	
Pl	*Poecilia latipinna*	Sailfin molly	1	0	0	0	full	34x	
Pom	*Poecilia Mexicana*	Shortfin molly	1	0	0	0	full	30x	
Pr	*Poecilia reticulata*	Guppy	1	0	0	0	full	110.0x	
Py	*Pseudopleuronectes yokohamae*	Marbled flounder	1	0	0	0	full	35x	
Pun	*Pundamilia nyererei*	Nyereres Viktoriabuntbarsch	1	0	0	0	full	126x	
Pyn	*Pygocentrus nattereri*	Red-bellied piranha	1	5	4	0	full	91x	
Rt	*Rhincodon typus*	Whale shark	2	0	0	4	full	20.0x	
Ss	*Salmo salar*	Atlantic salmon	3	0	3	0	full	206.0x	
Sh	*Scartelaos histophorus*	Blue mudskipper, walking goby	1	0	0	0	full	72x	
Sf	*Scleropages formosus*	Dragonfish, Asian bonytongue	2	0	4	0	full	100x	
Sea	*Sebastes aleutianus*	Rougheye rockfish	1	0	3	0	full	78.0x	
Sem	*Sebastes minor*	Akagaya	1	0	2	0	full	34.0x	
Ses	*Sebastes steindachneri*	Yanaginomai	1	0	1	0	full	47.0x	
Sn	*Sebastes nigrocinctus*	Tiger rockfish	1	0	1	0	full	94.0x	
Ser	*Sebastes rubrivinctus*	Flag rockfish	1	0	2	0	full	87.0x	
Sa	*Sinocyclocheilus anshuiensis*	Anshui sõõrhuul	2	4	5	0	full	60x	
Sg	*Sinocyclocheilus grahami*	Golden-line barbel	1	6	5	0	full	88x	
Sr	*Sinocyclocheilus rhinocerous*	Ninasarv-sõõrhuul	2	7	7	0	full	48x	
Stp	*Stegastes partitus*	Bicolor damselfish	1	0	0	0	full	121x	
Tf	*Takifugu flavidus*	Yellowbelly pufferfish	0	0	0	0	full	200x	GCA_003711565.2
Tr	*Takifugu rubripes*	Japanese pufferfish, torafugu	1	0	0	0	full	8.7x	
Tn	*Tetraodon nigroviridis*	Spotted green pufferfish	1	0	0	0	full	n/a	
To	*Thunnus orientalis*	Pacific bluefin tuna	2	0	0	0	full	54.9x	
Xl	*Xenopus laevis*	African clawed frog	1	n.d.	n.d.	n.d.	full	30x	
Xt	*Xenopus tropicalis*	Western clawed frog	1	4*	0	0	full	7.6x	* Syed et al., 2015 – DOI 10.1038/srep13935
Xc	*Xiphophorus couchianus*	Monterrey platyfish	1	0	0	0	full	77x	
Xh	*Xiphophorus hellerii*	Green swordtail	0	0	0	0	full	68x	GCA_003331165.2
Xm	*Xiphophorus maculatus*	Southern platyfish	1	0	0	0	full	83x	

***tarl* genes of jawless vertebrates identified in this study**

Species (Latin name and abbreviation)	Gene name	synonyms	Accession	length (full, partial)	Pseudo-gene (Ψ)	*, stop; !, frameshift	# exons	Database incl. Version number	chr	contig	location in contig (bp)	Strand (+/-)	Comments
**Jawless vertebrates**													
Lethenteron camtschaticum (Lec)	*tarl1a*	-	-	full	-	-	1	GCA_000466285.1	-	KE994369.1	40635-41954	+	
Lethenteron camtschaticum (Lec)	*tarl1b*	-	-	full	Ψ	1!	1	GCA_000466285.1	-	APJL01100547.1	1474-2801	+	
Lethenteron camtschaticum (Lec)	*tarl1c*	-	-	full	-	-	1	GCA_000466285.1	-	KE994369.1	23941-25266	+	
Lethenteron camtschaticum (Lec)	*tarl2a*	-	-	full	-	-	1	GCA_000466285.1	-	KE994369.1	162-1100	+	
Lethenteron camtschaticum (Lec)	*tarl2b*	-	-	partial	-	-	1	GCA_000466285.1	-	KE994480.1	13204-13944	-	
Lethenteron camtschaticum (Lec)	*tarl3a*	-	-	full	-	-	2	GCA_000466285.1	-	KE994369.1	8911-10535	+	
Lethenteron camtschaticum (Lec)	*tarl3b*	-	-	full	-	-	1	GCA_000466285.1	-	KE994369.1	49915-50784	-	
Lethenteron camtschaticum (Lec)	*tarl3c*	-	-	full	-	-	1	GCA_000466285.1	-	KE994369.1	66652-67515	-	
Lethenteron camtschaticum (Lec)	*tarl4a*	-	-	full	-	-	1	GCA_000466285.1	-	KE993775.1	1364576-1365646	+	
Lethenteron camtschaticum (Lec)	*tarl4b*	-	-	full	-	-	1	GCA_000466285.1	-	KE993775.1	1544889-1545953	+	
Lethenteron camtschaticum (Lec)	*tarl4c*	-	-	full	-	-	1	GCA_000466285.1	-	KE993775.1	1436910-1437974	+	
Lethenteron camtschaticum (Lec)	*tarl4d*	-	-	full	-	-	1	GCA_000466285.1	-	KE993775.1	1393333-1394397	+	
Lethenteron camtschaticum (Lec)	*tarl4e*	-	-	partial	-	-	1	GCA_000466285.1	-	KE993829.1	1308332-1308916	-	
Lethenteron camtschaticum (Lec)	*tarl5a*	-	-	full	-	-	1	GCA_000466285.1	-	KE993679.1	5764171-5765184	+	
Lethenteron camtschaticum (Lec)	*tarl5b*	-	-	full	-	-	1	GCA_000466285.1	-	KE993688.1	1032661-1033701	-	
Lethenteron camtschaticum (Lec)	*tarl5c*	-	-	full	-	-	1	GCA_000466285.1	-	KE993688.1	1019923-1020969	-	
Lethenteron camtschaticum (Lec)	*tarl5d*	-	-	partial	Ψ	1*	2	GCA_000466285.1	-	KE997555.1	6843-5134	-	
Lethenteron camtschaticum (Lec)	*tarl6a*	-	-	full	-	-	1	GCA_000466285.1	-	KE993775.1	473955-475010	+	
Lethenteron camtschaticum (Lec)	*tarl6b*	-	-	full	-	-	1	GCA_000466285.1	-	KE993775.1	486091-487152	+	
Lethenteron camtschaticum (Lec)	*tarl6c*	-	-	full	-	-	1	GCA_000466285.1	-	KE994203.1	259736-260932	-	
Lethenteron camtschaticum (Lec)	*tarl6d*	-	-	full	-	-	1	GCA_000466285.1	-	KE994203.1	296524-297528	-	
Lethenteron camtschaticum (Lec)	*tarl6e*	-	-	full	-	-	1	GCA_000466285.1	-	KE994203.1	278253-279257	-	
Lethenteron camtschaticum (Lec)	*tarl6f*	-	-	partial	-	-	1	GCA_000466285.1	-	KE993775.1	14829-15527	-	
Lethenteron camtschaticum (Lec)	*tarl6g*	-	-	full	-	-	1	GCA_000466285.1	-	KE993775.1	14829-15833	-	
Lethenteron camtschaticum (Lec)	*tarl6h*	-	-	full	-	-	1	GCA_000466285.1	-	KE993775.1	344070-345134	+	
Lethenteron camtschaticum (Lec)	*tarl6i*	-	-	full	-	-	1	GCA_000466285.1	-	KE993775.1	357384-358448	+	
Lethenteron camtschaticum (Lec)	*tarl6j*	-	-	full	-	-	1	GCA_000466285.1	-	KE993775.1	327596-328657	+	
Lethenteron camtschaticum (Lec)	*tarl6k*	-	-	full	-	-	1	GCA_000466285.1	-	KE994615.1	4070-5116	+	
Lethenteron camtschaticum (Lec)	*tarl6l*	-	-	full	-	-	1	GCA_000466285.1	-	KE994615.1	62137-63192	-	
Lethenteron camtschaticum (Lec)	*tarl6m*	-	-	full	-	-	1	GCA_000466285.1	-	KE994615.1	51233-52267	-	
Lethenteron camtschaticum (Lec)	*tarl6n*	-	-	full	-	-	1	GCA_000466285.1	-	KE993775.1	440758-441810	+	
Lethenteron camtschaticum (Lec)	*tarl6o*	-	-	full	-	-	1	GCA_000466285.1	-	KE993775.1	406856-407875	+	
Lethenteron camtschaticum (Lec)	*tarl6p*	-	-	full	-	-	1	GCA_000466285.1	-	KE993775.1	419329-420384	+	
Lethenteron camtschaticum (Lec)	*tarl6q*	-	-	full	-	-	1	GCA_000466285.1	-	APJL01103800.1	1115-45	-	
Lethenteron camtschaticum (Lec)	*tarl6r*	-	-	full	-	-	1	GCA_000466285.1	-	KE993775.1	380880-381923	+	
Lethenteron camtschaticum (Lec)	*tarl6s*	-	-	full	-	-	1	GCA_000466285.1	-	KE993775.1	218537-219592	+	
Lethenteron camtschaticum (Lec)	*tarl6t*	-	-	full	-	-	1	GCA_000466285.1	-	KE993775.1	204658-205671	+	
Lethenteron camtschaticum (Lec)	*tarl6u*	-	-	full	-	-	1	GCA_000466285.1	-	KE993775.1	279150-280196	+	
Lethenteron camtschaticum (Lec)	*tarl6v*	-	-	full	-	-	1	GCA_000466285.1	-	KE993775.1	303451-304497	+	
Lethenteron camtschaticum (Lec)	*tarl6w*	-	-	full	-	-	1	GCA_000466285.1	-	KE993775.1	452976-454028	+	
Lethenteron camtschaticum (Lec)	*tarl6x*	-	-	full	-	-	1	GCA_000466285.1	-	KE993775.1	465265-466299	+	
Lethenteron camtschaticum (Lec)	*tarl7a*	-	-	full	-	-	2	GCA_000466285.1	-	KE993775.1	570318-572603	+	
Lethenteron camtschaticum (Lec)	*tarl7b*	-	-	full	-	-	1	GCA_000466285.1	-	KE994615.1	10587-11681	-	
Lethenteron camtschaticum (Lec)	*tarl7c*	-	-	full	-	-	1	GCA_000466285.1	-	KE993904.1	434011-435126	-	
Lethenteron camtschaticum (Lec)	*tarl7d*	-	-	partial	-	-	1	GCA_000466285.1	-	APJL01179032.1	171-950	+	
Lethenteron camtschaticum (Lec)	*tarl7e*	-	-	full	-	-	1	GCA_000466285.1	-	KE993775.1	520997-522049	-	
Lethenteron camtschaticum (Lec)	*tarl7f*	-	-	full	-	-	1	GCA_000466285.1	-	KE993775.1	494975-496018	+	
Lethenteron camtschaticum (Lec)	*tarl7g*	-	-	full	-	-	1	GCA_000466285.1	-	KE993775.1	504937-506031	+	
Lethenteron camtschaticum (Lec)	*tarl7h*	-	-	full	-	-	1	GCA_000466285.1	-	KE993775.1	552562-553617	+	
Lethenteron camtschaticum (Lec)	*tarl7i*	-	-	full	-	-	1	GCA_000466285.1	-	KE993775.1	559513-560583	+	
Lethenteron camtschaticum (Lec)	*tarl7j*	-	-	full	-	-	1	GCA_000466285.1	-	KE993775.1	537538-538587	+	
Lampetra fluviatilis (Laf)	*tarl4a*	LOR12	AAC82384.1	partial	-	-	1	n.a.	-	AF069549.1	1-774	n.a.	
Lampetra fluviatilis (Laf)	*tarl6a*	LOR4	AAC82382.1	partial	-	-	1	n.a.	-	AF069547.1	1-1059	n.a.	
Lampetra fluviatilis (Laf)	*tarl6b*	LOR22	AAC82385.1	partial	-	-	1	n.a.	-	AF069550.1	1-768	n.a.	
Lampetra fluviatilis (Laf)	*tarl6c*	LOR6	AAC82388.1	partial	-	-	1	n.a.	-	AF069553.1	1-510	n.a.	
Lampetra fluviatilis (Laf)	*tarl6d*	LOR20	AAC82387.1	partial	-	-	1	n.a.	-	AF069552.1	1-543	n.a.	
Lampetra fluviatilis (Laf)	*tarl6e*	LOR14	AAC82383.1	partial	-	-	1	n.a.	-	AF069548.1	1-1020	n.a.	
Lampetra fluviatilis (Laf)	*tarl6f*	LOR25	AAC82386.1	partial	-	-	1	n.a.	-	AF069551.1	1-519	n.a.	
Lampetra fluviatilis (Laf)	*tarl6g*	LOR16	AAC82389.1	partial	-	-	1	n.a.	-	AF069554.1	1-522	n.a.	
Lampetra fluviatilis (Laf)	*tarl7a*	LOR3	AAC82381.1	partial	-	-	1	n.a.	-	AF069546.1	1-1056	n.a.	
Petromyzon marinus (Pm)	*tarl1a*	-	-	full	-	-	1	GCA_002833325.1	-	PIZI01000032.1	6275329-6276639	+	
Petromyzon marinus (Pm)	*tarl2a*	-	-	partial	-	-	1	GCA_002833325.1	-	PIZI01000032.1	6253583-6254518	+	
Petromyzon marinus (Pm)	*tarl2b*	-	-	partial	Ψ	1* 1!	1	GCA_002833325.1	-	PIZI01000103.1	284940-285702	+	
Petromyzon marinus (Pm)	*tarl2c*	-	-	full	Ψ	4!	2	GCA_002833325.1	-	PIZI01000103.1	284148-285702	+	
Petromyzon marinus (Pm)	*tarl3a*	-	-	partial	-	-	1	GCA_002833325.1	-	PIZI01000032.1	6299113-6300003	-	
Petromyzon marinus (Pm)	*tarl4a*	-	-	full	Ψ	1*	1	WGS_VDB://AEFG01	-	AEFG01051656.1	3845-2811	-	WGS, not found in genome assembly
Petromyzon marinus (Pm)	*tarl4b*	-	-	full	-	-	1	WGS_VDB://AEFG01	-	AEFG01045923.1	3064-4137	+	WGS, not found in genome assembly
Petromyzon marinus (Pm)	*tarl5a*	#		full	-	-	1	GCA_002833325.1	-	PIZI01001092.1	11747-12763	+	
Petromyzon marinus (Pm)	*tarl5b*	#		full	-	-	1	WGS_VDB://AEFG01	-	AEFG01032376.1	5123-6163	+	WGS, in genome assembly incomplete
Petromyzon marinus (Pm)	*tarl5c*	#		full	-	-	1	WGS_VDB://AEFG01	-	AEFG01032376.1	2293-3333	+	WGS, frame shift in genome assembly
Petromyzon marinus (Pm)	*tarl5d*	-	-	partial	-	-	1	WGS_VDB://AEFG01	-	PIZI01000009.1	10839713-10840192	-	
Petromyzon marinus (Pm)	*tarl6a*	#, TAAR353*		full	-	-	1	WGS_VDB://AEFG01	-	AEFG01071253.1	6274-7332	-	WGS, not found in genome assembly
Petromyzon marinus (Pm)	*tarl6b*	TAAR335*		full	-	-	1	GCA_002833325.1	-	PIZI01005860.1	3568-4575	-	
Petromyzon marinus (Pm)	*tarl6c*	#, TAAR355b*		full	-	-	1	WGS_VDB://AEFG01	-	AEFG01071256.1	5671-6783	-	WGS, 2 frame shifts in genome assembly
Petromyzon marinus (Pm)	*tarl6d*	#, TAAR355a*		full	-	-	1	WGS_VDB://AEFG01	-	AEFG01071255.1	1898-2965	-	WGS, not found in genome assembly
Petromyzon marinus (Pm)	*tarl6e*	#, TAAR354*		full	-	-	1	WGS_VDB://AEFG01	-	AEFG01071258.1	13628-14692	-	WGS, frame shift in genome assembly
Petromyzon marinus (Pm)	*tarl6f*	TAAR353*		full	-	-	1	WGS_VDB://AEFG01		AEFG01041529.1	3108-4169	-	WGS, not found in genome assembly
Petromyzon marinus (Pm)	*tarl6g*	TAAR360*		full	-	-	1	WGS_VDB://AEFG01	-	AEFG01028932.1	1473-2552	-	WGS, frame shift in genome assembly
Petromyzon marinus (Pm)	*tarl6h*	#, TAAR345*		full	-	-	1	GCA_002833325.1	-	PIZI01000032.1	322327-323364	-	
Petromyzon marinus (Pm)	*tarl6i*	TAAR351*		full	-	-	1	GCA_002833325.1	-	PIZI01000032.1	4902127-4903182	+	
Petromyzon marinus (Pm)	*tarl6j*	#, TAAR340*		full	-	-	1	WGS_VDB://AEFG01	-	AEFG01025162.1	5312-6334	-	WGS, frame shift in genome assembly
Petromyzon marinus (Pm)	*tarl6k*	#, TAAR358*		full	-	-	1	GCA_002833325.1	-	AEFG01025161.1	2059-3069	-	WGS, 2 frame shifts in genome assembly
Petromyzon marinus (Pm)	*tarl6l*	TAAR349a*		full	-	-	1	GCA_002833325.1	-	PIZI01000032.1	4747149-4748162	+	
Petromyzon marinus (Pm)	*tarl6m*	TAAR349b*		partial	-	-	1	WGS_VDB://AEFG01	-	AEFG01019733.1	1 – 663	-	WGS, frame shift in genome assembly
Petromyzon marinus (Pm)	*tarl6n*	#, TAAR351*		full	-	-	1	WGS_VDB://AEFG01	-	AEFG01033136.1	2692-3747	-	WGS, 2 frame shifts in genome assembly
Petromyzon marinus (Pm)	*tarl6o*	#, TAAR345*		full	-	-	1	WGS_VDB://AEFG01	-	AEFG01033134.1	1788-2825	-	WGS, frame shift in genome assembly
Petromyzon marinus (Pm)	*tarl7a*	-	-	full	-	-	2	GCA_002833325.1	-	PIZI01000586.1	41436-43748	-	
Petromyzon marinus (Pm)	*tarl7b*	#		partial	-	-	1	WGS_VDB://AEFG01		AEFG01041527.1	651-1601	+	WGS, not found in genome assembly
Petromyzon marinus (Pm)	*tarl7c*	-	-	partial	-	-	1	WGS_VDB://AEFG01	-	AEFG01042991.1	2131-3012	+	WGS, 2 frame shifts in genome assembly
Petromyzon marinus (Pm)	*tarl7d*	#, TAAR351*		full	-	-	1	GCA_002833325.1	-	PIZI01000032.1	5000570-5001625	+	
Petromyzon marinus (Pm)	*tarl7e*	#, TAAR365*		full	-	-	1	GCA_002833325.1	-	PIZI01000032.1	4990958-4992055	+	
Petromyzon marinus (Pm)	*tarl7f*	#		full	-	-	1	GCA_002833325.1	-	PIZI01000032.1	5008987-5010039	+	

#, described, but not named in Hashiguchi and Nishida, 2007
*, described in Libants et al., 2009

***tarl* genes of jawed vertebrates identified in this study**

Species (Latin name and abbreviation)	Gene name	automatted annotation	Accession	length (full, partial)	Pseudo-gene (Ψ)	*, stop; !, frameshift	# exons	Database incl. Version number	chr	contig	location in contig (bp)	Strand (+/-)	Comments
Cartilaginous fish													
Callorhinchus milii (Cm)	*tarl2*	5-HTR4-like	LOC103186492	full	-	-	1	GCF_000165045.1	-	NW_006890197.1	1614139-1615236	+	
Leucoraja erinacea (Le)	*tarl2*	-	-	full	Ψ	1* 3!	1	WGS_VDB://AESE01	-	AESE010035790.1; AESE010257460.1	124-760;4-312	+, -	
Rhincodon typus (Rt)	*tarl1*	-	LOC109911122	full	-	-	1	GCF_001642345.1	-	NW_018030254.1	4655-5698	+	
Rhincodon typus (Rt)	*tarl2*	5-HTR4-like	LOC109915544	full	-	-	1	GCF_001642345.1	-	NW_018027816.1	181321-182421	-	
Amphibians													
Xenopus laevis (Xl)	*tarl1*	TAAR1-like	LOC108719346	full	-	-	1	GCF_001663975.1	1L	NC_030724.1	2828991-2830067	-	
Xenopus tropicalis (Xt)	*tarl1*	TAAR1	LOC100496115	full	-	-	1	GCF_000004195.3	1	NC_030677.1	5537899-5538957	+	all five *taar* genes on chromosome 5, *htr4* on chromosome 3
**Bony fish**													
Anguilla anguilla (Aa)	*tarl1*	-	-	full	-	-	1	GCA_000695075.1	-	AZBK01800005.1	9-1076	+	
Anguilla japonica (Aj)	*tarl1a*	-	-	full	-	-	1	GCA_003597225.1	-	BEWY01000002.1	9612299-9613369	-	
Anguilla japonica (Aj)	*tarl1b*	-	-	full	-	-	1	GCA_003597225.1	-	BEWY01000015.1	12067318-12068430	+	
Anguilla rostrata (Ar)	*tarl1*	-	-	full	-	-	1	WGS_VDB://LTYT01	-	LTYT01002349.1	67811-68917	+	GCA_001606085.1
Anguilla rostrata (Ar)	*tarl2*	-	-	full	-	-	1	WGS_VDB://LTYT01	-	LTYT01004196.1	172162-173274	-	GCA_001606085.1
Anoplopoma fimbria (Af)	*tarl1*	-	-	full	-	-	1	GCA_000499045.1	-	AWGY01160213.1	10245-11303	-	
Amphilophus citrinellus (Ac)	*tarl1*	-	-	full	-	-	2	GCA_013435755.1		CM024197.1	22725795-22724719	-	
Astyanax mexicanus (Am)	*tarl1*	-	LOC103027537	full	-	-	1	GCF_000372685.2	-	NW_019172941.1	420606-421679	-	
Austrofundulus limnaeus (Al)	*tarl1*	5-HTR4-like	XP_013863315.1	full	-	-	1	GCF_001266775.1	-	XM_014007861.1	225-1280	+	
Boleophthalmus pectinirostris (Bp)	*tarl1*	5-HTR4-like	XP_020786106.1	full	-	-	1	GCF_000788275.1	-	NW_018356255.1	3197207-3198265	+	
Clupea harengus (Ch)	*tarl1*	-	LOC105903878	full	-	-	1	GCF_000966335.1	-	NW_012221853.1	1367956-1369011	-	
Cottus rhenanus (Cr)	*tarl1*	-	-	full	-	-	1	GCA_001455555.1	-	LKTN01076329.1	4308-5405	+	
Cynoglossus semilaevis (Cs)	*tarl1*	5-HTR4-like	XP_008315345.1	full	-	-	1	GCF_000523025.1	9	NC_024315.1	9847779-9848852	+	
Cyprinodon nevadensis pectoralis (Cn)	*tarl1*	-	-	full	-	-	1	GCA_000776015.1	-	JSUU01003036.1	31440-32498	-	
Cyprinodon variegatus (Cv)	*tarl1*	5-HTR4-like	XP_015228425.1	full	-	-	1	GCF_000732505.1	-	NW_015150531.1	763289-764344	-	
Cyprinus carpio (Cc)	*tarl1a*	-	LOC109066000; LOC109065999	full	Ψ	1!	1	GCF_000951615.1	-	NW_017538292.1	209579-210642	-	
Cyprinus carpio (Cc)	*tarl1b*	-	LOC109061621	full	Ψ	1*	1	GCF_000951615.1	-	NW_017538179.2	182099-183163	-	
Danio rerio (Dr)	*tarl1*	5-HTR4-like	XP_001337671.1	full	-	-	1	GCF_000002035.6	1	NC_007112.7	41949321-41950379	+	
Dicentrarchus labrax (Dl)	*tarl1*	-	-	full	-	-	1	GCA_000689215.1	-	HG916847.1	18587864-18588940	+	
Esox lucius (El)	*tarl1*	-	LOC105021074	full	-	-	1	GCF_000721915.3	-	NC_025992.3	3614001-3615086	-	
Esox lucius (El)	*tarl2*	5-HTR4-like	XP_010900554.1	full	-	-	1	GCF_000721915.3	-	NC_025971.3	13319582-13320742	+	
Fundulus heteroclitus (Fh)	*tarl1*	TAAR3-like	XP_012733128.2	full	-	-	1	GCF_000826765.1	-	NW_012234461.1	109571-110629	-	
Gadus morhua (Gm)	*tarl1a*	-	-	full	-	-	1	GCF_902167405.1	3	NC_044050.1	10445555-10444476	-	
Gadus morhua (Gm)	*tarl1b*	-	-	full	-	-	1	WGS_VDB://CAEA02	-	CAEA020003354.1	429215-428181	-	not in GCF_902167405.1
Gasterosteus aculeatus (Ga)	*tarl1*	-	-	full	-	-	1	GCA_000180675.1	-	AANH01003210.1	55276-56352	+	
Haplochromis burtoni (Hb)	*tarl1*	5-HTR4-like	XP_005928396.2	full	-	-	1	GCF_000239415.1	-	NW_005179495.1	110901-111977	-	
Hippocampus comes (Hc)	*tarl1*	5-HTR4-like	XP_019732039.1	full	-	-	1	GCF_001891065.1	-	NW_017805497.1	3571388-3572455	-	
Ictalurus punctatus (Ip)	*tarl1*	-	LOC108260501	full	-	-	1	GCF_001660625.1	29	NC_030444.1	13728150-13729223	+	
Kryptolebias marmoratus (Km)	*tarl1*	-	-	full	-	-	1	ASM164957v1	-	NW_016094240.1	1908256-1909314	+	
Labrus bergylta (Lb)	*tarl1*	-	LOC109994121	full	-	-	1	GCF_900080235.1	-	NW_018114694.1	280280-281356	+	
Larimichthys crocea (Lc)	*tarl1*	5-HTR4	XP_027138281.1	full	-	-	1	GCF_000972845.2	X	NC_040020.1	2591785-2592858	-	
Lates calcarifer (Lac)	*tarl1*	5-HTR4-like	XP_018551385.1	full	-	-	1	GCF_001640805.1	-	NW_017365769.1	1510510-1511586	+	
Lates calcarifer (Lac)	*tarl2*	-	-	full	Ψ	1 *	1	GCF_001640805.1	-	NW_017363881.1	4502697-4503698	-	
Lepisosteus oculatus (Lo)	*tarl1*	-	-	full	-	-	1	GCF_000242695.1	LG4	NC_023182.1	5406278-5407351	+	
Leuciscus waleckii (Lw)	*tarl1*	-	-	full	Ψ	1!	1	GCA_900092035.1	-	FLSR01004869.1	10142941-10144067	-	
Maylandia zebra (Mz)	*tarl1*	-	-	full	-	-	1	GCF_000238955.4		NC_036785	19082571-19083644	-	
Mchenga conophoros (Mc)	*tarl1*	-	-	partial	Ψ	-	n.d.	GCA_000150855.1	-	ABPJ01069830.1, ABPJ01067448.1	456-773, 726-604	+,-	
Miichthys miiuy (Mim)	*tarl1*	-	-	full	-	-	1	GCA_001593715.1	-	JXSJ01001024.1	6749-7822	+	
Mola mola (Mom)	*tarl1*	-	-	full	-	-	1	GCA_001698575.1	-	KV751317.1	19347442-19348629	-	
Monopterus albus (Moa)	*tarl1*	5-HTR4-like	XP_020457684.1	full	-	-	1	GCF_001952655.1	-	NW_018127934.1	1776181-1777257	-	
Morone saxatilis (Ms)	*tarl1*	-	-	full	-	-	1	GCA_001663605.1	-	JTCL01023305.1	2572-3654	+	
Neolamprologus brichardi (Nb)	*tarl1*	5-HTR4-like	XP_006787601.1	full	-	-	1	GCF_000239395.1	-	NW_006272012.1	3793196-3794272	-	
Nothobranchius furzeri (Nf)	*tarl1*	-	-	full	-	-	1	GCF_001465895.1	sgr02	NC_029650.1	83794088-83795149	+	
Oreochromis niloticus (On)	*tarl1*	5-HTR4-like	XP_025764064.1	full	-	-	1	GCF_001858045.2	-	NC_031971.2	20801250-20802326	-	
Oryzias latipes (Ol)	*tarl1*	TAAR1-like	XP_004085557.3	full	-	-	1	GCF_002234675.1	1	NC_019859.2	14263830-14264918	+	
Pampus argenteus (Pa)	*tarl1*	-	-	full	-	-	1	GCA_000697985.1	-	JHEK01029396.1	391-1470	-	
Paralichtys olivaceus (Po)	*tarl1*	-	-	full	-	-	1	GCF_001970005.1	-	NW_017859678.1	2600450-2601526	+	
Periophthalmodon schlosseri (Ps)	*tarl1*	-	-	full	-	-	1	GCA_000787095.1	-	JACM01022706.1	42492-41431	-	
Periophthalmus magnuspinnatus (Pem)	*tarl1*	-	-	full	-	-	1	GCA_000787105.1	-	KN462927.1	37253-38383	+	
Pimephales promelas (Pp)	*tarl1*	-	-	full	-	-	1	GCA_000700825.1	-	JNCD01012483.1	34693-35757	-	
Poecilia formosa (Pf)	*tarl1*	5-HTR4-like	XP_016526920.1	full	-	-	1	GCF_000485575.1	-	NW_006799983.1	1413491-1414549	-	
Poecilia latipinna (Pl)	*tarl1*	5-HTR4-like	XP_014889534.1	full	-	-	1	GCF_001443285.1	-	NW_015112902.1	147239-148297	-	
Poecilia mexicana (Pom)	*tarl1*	5-HTR4-like	XM_015004974.1	full	-	-	1	GCF_001443325.1	-	NW_015095551.1	360810-361865	-	
Poecilia reticulata (Pr)	*tarl1*	-	GeneID:103467537	full	-	-	1	GCF_000633615.1	-	NC_024331.1	20413752-20414810	+	
Pseudopleuronectes yokohamae (Py)	*tarl1*	-	-	full	-	-	1	GCA_000787555.1	-	BBOV01036133.1	602-1678	-	
Pundamilia nyererei (Pun)	*tarl1*	5-HTR4-like	XP_005741265.2	full	-	-	1	GCF_000239375.1	-	NW_005187508.1	23053-24129	-	
Pygocentrus nattereri (Pyn)	*tarl1*	-	LOC108434381	full	-	-	1	GCF_001682695.1	-	NW_016243911.1	1365121-1366194	-	
Salmo salar (Ss)	*tarl1a*	-	-	full	-	-	1	GCF_000233375.1	ssa04	NC_027303.1	20922696-20923904	+	
Salmo salar (Ss)	*tarl1b*	-	-	full	Ψ	1*	1	GCF_000233375.1	ssa08	NC_027307.1	4286494-4287624	+	
Salmo salar (Ss)	*tarl2*	-	-	full	-	-	1	GCF_000233375.1	ssa09	NC_027308.1	70306722-70307939	+	
Scartelaos histophorus (Sh)	*tarl1*	-	-	full	-	-	1	GCA_000787155.1	-	KN487589.1	9394 -10455	+	
Scleropages formosus (Sf)	*tarl1*	-	LOC108924382	full	-	-	1	GCF_001624265.1	-	NW_017371553.1	5548625-5549701	+	
Scleropages formosus (Sf)	*tarl2*	TAAR9-like	XP_018607201.1	partial	-	-	1	GCF_001624265.1	-	NW_017371831.1	4523316-4524268	-	
Sebastes aleutianus (Sea)	*tarl1*	-	-	full	-	-	1	GCA_001910805.2	-	LHVS01034712.1	8104-9174	+	
Sebastes minor(Sem)	*tarl1*	-	-	full	-	-	1	GCA_001910765.2	-	LHCJ01538447.1	456-1526	-	
Sebastes nigrocinctus (Sn)	*tarl1*	-	-	full	-	-	1	GCA_000475235.3	-	AUPR02010153.1	9020-10090	-	
Sebastes rubrivinctus (Ser)	*tarl1*	-	-	full	-	-	1	GCA_000475215.1	-	KI455860.1	2786-3856	+	
Sebastes steindachneri (Ses)	*tarl1*	-	-	full	-	-	1	GCA_001910785.2	-	LCWK010689365.1	647-1717	+	
Sinocyclocheilus anshuiensis (Sa)	*tarl1a*	5-HTR4-like	XP_016342958.1	full	-	-	1	GCF_001515605.1	-	NW_015540933.1	270706-271770	+	
Sinocyclocheilus anshuiensis (Sa)	*tarl1b*	5-HTR4-like	XP_016334547.1	full	-	-	1	GCF_001515605.1	-	NW_015619744.1	1-879	-	
Sinocyclocheilus rhinocerous (Sr)	*tarl1a*	-	LOC107719773	full	-	-	1	GCF_001515625.1	-	NW_015656168.1	47289-48353	-	
Sinocyclocheilus rhinocerous (Sr)	*tarl1b*	-	LOC107711518	full	-	-	1	GCF_001515625.1	-	NW_015650091.1	727057-728118	-	
Sinocyclocheilus grahami (Sg)	*tarl1*	5-HTR4.ps	Gene ID:107552355	full	-	-	1	GCF_001515645.1	-	NW_015505196.1	5614517-5615581	-	
Stegastes partitus (Stp)	*tarl1*	5-HTR4-like	XP_008296907.1	full	-	-	1	GCF_000690725.1	-	NW_007577760.1	271701-272777	+	
Takifugu rubripes (Tr)	*tarl1*	5-HTR4-like	XP_003976403.1	full	-	-	1	GCF_000180615.1	-	NW_004071248.1	83330-84367	-	
Tetraodon nigroviridis (Tn)	*tarl1*	-	CAF93600.1	full	-	-	1	GCA_000180735.1	-	CAAE01011002.1	7437-8495	-	
Thunnus orientalis (To)	*tarl1*	-	-	full	-	-	1	GCA_000418415.1	-	BADN01004245.1	4208-5284	-	
Thunnus orientalis (To)	*tarl2*	-	-	full	-	-	1	GCA_000418415.1	-	BADN01043078.1	6332-7453	+	
Xiphophorus couchianus (Xc)	*tarl1*	-	-	full	-	-	1	GCA_001444195.3	5	CM010730.1	15290538-15291596	+	
Xiphophorus maculatus (Xm)	*tarl1*	-	-	full	-	-	1	GCF_002775205.1	5	NC_036447.1	14312375-14313433	+	

***taar* genes of jawed vertebrates identified in this study**

Species (Latin name and abbreviation)	Gene name	Automatted annotation	Accession	Length (full, partial)	Pseudo-gene (Ψ)	*, stop; !, frameshift	# exons	Database incl. Version number	Chromo-some	Contig	Location in contig (bp)	Strand (+/-)	Comments
**Cartilaginous fish**													
Callorhinchus milii (Cm)	*taar-IIa*	TAAR4-like	XP_007897493.1	full	Ψ	1! 1*	1	GCF_000165045.1	-	NW_006890121.1	242855-243884	+	
Callorhinchus milii (Cm)	*taar-IIb*	TAAR2-like	XP_007897495.1	full	Ψ	2! 1*	1	GCF_000165045.1	-	NW_006890121.1	258544-259545	+	
Callorhinchus milii (Cm)	*taar-IIc*	TAAR4-like	XP_007897494.1	full	-	-	1	GCF_000165045.1	-	NW_006890121.1	248799-249827	+	
Leucoraja erinacea (Le)	*taar-IIa*	-	-	full	-	-	1	WGS_VDB://AESE01	-	AESE011082060.1, AESE010020089.1	1-856, 26-502	+,+	not present in genome assembly
Rhincodon typus (Rt)	*taar-IIa*	TAAR4-like	XP_020378973.1	full	Ψ	1!	1	GCF_001642345.1	-	NW_018045707.1	45125-46162	+	
Rhincodon typus (Rt)	*taar-IIb*	TAAR4-like	XP_020378971.1	full	Ψ	2! 2*	1	GCF_001642345.1	-	NW_018045707.1	21650-22701	+	
Rhincodon typus (Rt)	*taar-IIc*	TAAR4-like	XP_020378970.1	5' partial	-	-	1	GCF_001642345.1	-	NW_018045707.1	66985-67908	-	
Rhincodon typus (Rt)	*taar-IId*	TAAR4-like	XP_020378972.1	full	-	-	1	GCF_001642345.1	-	NW_018045707.1	37597_38635	+	
**Bony fish**													
Anguilla anguilla (Aa)	*taar12a*	-	-	full	-	-	1	GCA_000695075.1	-	AZBK01826618.1	794-1840	+	
Anguilla anguilla (Aa)	*taar12b*	-	-	full	-	-	2	GCA_000695075.2	-	AZBK01826619.1	1284-2473	+	
Anguilla anguilla (Aa)	*taar12c*	-	-	full	-	-	1	GCA_000695075.3	-	AZBK01826622.1	5586-6632	+	
Anguilla anguilla (Aa)	*taar12d*	-	-	partial	-	-	1	GCA_000695075.4	-	AZBK01826625.1	2-592	+	
Anguilla anguilla (Aa)	*taar12e*	-	-	partial	-	-	1	GCA_000695075.5	-	AZBK01503223.1	12-566	-	
Anguilla anguilla (Aa)	*taar12f*	-	-	full	-	-	1	GCA_000695075.6	-	AZBK01826622.1	1234-2283	+	
Anguilla anguilla (Aa)	*taar12g*	-	-	partial	-	-	1	GCA_000695075.7	-	AZBK01826620.1	2-835	+	
Anguilla anguilla (Aa)	*taar13a*	-	-	partial	-	-	1	GCA_000695075.1	-	AZBK01826618.1	9792-10505	-	
Anguilla anguilla (Aa)	*taar13b*	-	-	full	-	-	1	GCA_000695075.1	-	AZBK01826618.1	6365-7402	-	
Anguilla anguilla (Aa)	*taar13c*	-	-	partial	-	-	1	GCA_000695075.1	-	AZBK01826606.1	1-714	+	
Anguilla anguilla (Aa)	*taar13d*	-	-	partial	-	-	1	GCA_000695075.1	-	AZBK01826616.1	4358-5053	+	
Anguilla anguilla (Aa)	*taar13e*	-	-	full	-	-	1	GCA_000695075.1	-	AZBK01826603.1	2-1024	+	
Anoplopoma fimbria (Af)	*taar13a*	-	-	full	-	-	1	GCA_000499045.1	-	AWGY01187787.1	907-1842	+	
Anoplopoma fimbria (Af)	*taar13b*	-	-	partial	-	-	1	GCA_000499045.1	-	AWGY01171310.1	3-785	-	
Anguilla japonica (Aj)	*taar12a*	-	-	full	-	-	1	GCA_003597225.1	LG19	BEWY01000019.1	10043670-10044719	+	
Anguilla japonica (Aj)	*taar12b*	-	-	full	-	-	1	GCA_003597225.1	LG19	BEWY01000019.1	10038585-10039640	+	
Anguilla japonica (Aj)	*taar12c*	-	-	full	-	-	1	GCA_003597225.1	LG19	BEWY01000019.1	10023900-10024949	+	
Anguilla japonica (Aj)	*taar12d*	-	-	full	-	-	1	GCA_003597225.1	LG19	BEWY01000019.1	10006478-10007524	+	
Anguilla japonica (Aj)	*taar12e*	-	-	full	-	-	1	GCA_003597225.1	LG19	BEWY01000019.1	9993867-9994913	+	
Anguilla japonica (Aj)	*taar12f*	-	-	full	-	-	1	GCA_003597225.1	LG19	BEWY01000019.1	9986347-9987393	+	
Anguilla japonica (Aj)	*taar12g*	-	-	full	-	-	1	GCA_003597225.1	LG19	BEWY01000019.1	10033303-10034355	+	
Anguilla japonica (Aj)	*taar12h*	-	-	full	-	-	1	GCA_003597225.1	LG19	BEWY01000019.1	9977289-9978401	+	genomic sequence has 1 aa difference to WGS
Anguilla japonica (Aj)	*taar12i*	-	-	full	-	-	1	GCA_003597225.1	LG19	BEWY01000019.1	10001006-10002058	+	
Anguilla japonica (Aj)	*taar13a*	-	-	full	-	-	1	WGS_VDB://AVPY01	-	AVPY01144813.1	1202-262	-	not present in genome assembly
Anguilla japonica (Aj)	*taar13b1*	-	-	full	Ψ	1!	1	GCA_003597225.1	LG19	BEWY01000019.1	10053840-10052801	-	genomic sequence has 4 aa difference to WGS
Anguilla japonica (Aj)	*taar13b2*	-	-	full	-	-	1	GCA_003597225.2	LG19	BEWY01000019.2	10050453-10049413	-	
Anguilla japonica (Aj)	*taar13c*	-	-	partial	-	-	1	GCA_003597225.1	LG19	BEWY01000019.1	10072929-10073579	+	
Anguilla japonica (Aj)	*taar13d1*	-	-	partial	-	-	1	WGS_VDB://AVPY01	-	AVPY01144817.1	3-797	+	pseudogene in genomic database
Anguilla japonica (Aj)	*taar13d2*	-	-	full	-	-	1	GCA_003597225.1	LG19	BEWY01000019.1	10063062-10064087	+	
Anguilla japonica (Aj)	*taar13e*	-	-	full	-	-	1	GCA_003597225.1	LG19	BEWY01000019.1	10078261-10079310	+	
Anguilla japonica (Aj)	*taar13f*	-	-	partial	-	-	1	GCA_003597225.1	LG19	BEWY01000019.1	10070326-10071114	+	genomic sequence has 4 aa difference to WGS
Anguilla japonica (Aj)	*taar13g*	-	-	full	-	-	1	GCA_003597225.2	LG19	BEWY01000019.1	10066035-10067006	+	genomic sequence has 4 aa difference to WGS
Astyanax mexicanus (Am)	*taar12a*	TAAR4-like	XP_015463987.2	full	-	-	1	GCF_000372685.2	3	NC_035899.1	39668818-39669713	+	
Astyanax mexicanus (Am)	*taar12b*	TAAR4-like	XP_015463989.2	full	-	-	1	GCF_000372685.2	3	NC_035899.1	39667005-39665971	-	
Astyanax mexicanus (Am)	*taar12c*	TAAR4-like	XP_007259911.2	full	-	-	1	GCF_000372685.2	3	NC_035899.1	39661121-39662152	-	
Astyanax mexicanus (Am)	*taar13a*	TAAR13c-like	XP_015463986.2	full	-	-	1	GCF_000372685.2	3	NC_035899.1	39656351-39657388	+	
Anguilla rostrata (Ar)	*taar12a*	-	-	full	-	-	1	WGS_VDB://LTYT01	-	LTYT01001697.1	195538-196587	-	
Anguilla rostrata (Ar)	*taar12b*	-	-	full	-	-	1	WGS_VDB://LTYT01	-	LTYT01001697.1	200565-201620	-	
Anguilla rostrata (Ar)	*taar12c*	-	-	full	-	-	1	WGS_VDB://LTYT01	-	LTYT01001697.1	228169-229218	-	
Anguilla rostrata (Ar)	*taar12d*	-	-	partial	-	-	1	WGS_VDB://LTYT01	-	LTYT01007895.1	31310-32080	+	
Anguilla rostrata (Ar)	*taar12e*	-	-	full	-	-	1	WGS_VDB://LTYT01	-	LTYT01007895.1	15225-16259	+	
Anguilla rostrata (Ar)	*taar12f*	-	-	full	-	-	1	WGS_VDB://LTYT01	-	LTYT01007895.1	9659-10705	+	
Anguilla rostrata (Ar)	*taar12g1*	-	-	full	-	-	1	WGS_VDB://LTYT01	-	LTYT01007895.1	3930-4976	+	
Anguilla rostrata (Ar)	*taar12g2*	-	-	partial	-	-	1	WGS_VDB://LTYT01	-	LTYT01007895.1	3-749	+	
Anguilla rostrata (Ar)	*taar12h*	-	-	full	-	-	1	WGS_VDB://LTYT01	-	LTYT01006975.1	1392-2504	-	
Anguilla rostrata (Ar)	*taar12i*	-	-	full	-	-	1	WGS_VDB://LTYT01	-	LTYT01007895.1	19379-20431	+	
Anguilla rostrata (Ar)	*taar12j*	-	-	full	-	-	1	WGS_VDB://LTYT01	-	LTYT01001697.1	232505-233554	-	
Anguilla rostrata (Ar)	*taar12k*	-	-	full	-	-	1	WGS_VDB://LTYT01	-	LTYT01007895.1	34780-35829	+	
Anguilla rostrata (Ar)	*taar12l*	-	-	full	-	-	1	WGS_VDB://LTYT01	-	LTYT01007895.1	30123-31163	+	
Anguilla rostrata (Ar)	*taar13a*	-	-	full	-	-	1	WGS_VDB://LTYT01	-	LTYT01001697.1	184810-185850	+	
Anguilla rostrata (Ar)	*taar13b1*	-	-	full	-	-	1	WGS_VDB://LTYT01	-	LTYT01001697.1	190058-191098	+	
Anguilla rostrata (Ar)	*taar13b2*	-	-	full	-	-	1	WGS_VDB://LTYT01	-	LTYT01001697.1	181375-182415	+	
Anguilla rostrata (Ar)	*taar13c*	-	-	full	-	-	1	WGS_VDB://LTYT01	-	LTYT01001697.1	162511-163551	-	
Anguilla rostrata (Ar)	*taar13d1*	-	-	full	-	-	1	WGS_VDB://LTYT01	-	LTYT01001697.1	170738-171778	-	
Anguilla rostrata (Ar)	*taar13d2*	-	-	full	-	-	1	WGS_VDB://LTYT01	-	LTYT01001697.1	174330-175364	-	
Anguilla rostrata (Ar)	*taar13e*	-	-	full	-	-	1	WGS_VDB://LTYT01	-	LTYT01001697.1	156645-157697	-	
Anguilla rostrata (Ar)	*taar13f*	-	-	full	-	-	1	WGS_VDB://LTYT01	-	LTYT01001697.1	167001-168050	-	
Cyprinus carpio (Cc)	*taar12a*	TAAR4-like	XP_018959267.1	full	-	-	1	GCF_000951615.1	9	NC_031705.1	15759047-15760075	-	
Cyprinus carpio (Cc)	*taar12b*	TAAR4-like	XP_018959253.1	full	-	-	1	GCF_000951615.1	9	NC_031705.1	15752901-15753935	-	
Cyprinus carpio (Cc)	*taar12c*	-	LOC109075658	full	-	-	1	WGS_VDB://LHQP01	-	LHQP01008012.1	15916-14771	-	pseudogene in genomic database
Cyprinus carpio (Cc)	*taar12d*	-	LOC109080729	full	Ψ	1! 1*	1	GCF_000951615.1	9	NC_031705.1	15764159-15764995	-	
Cyprinus carpio (Cc)	*taar12e*	TAAR4-like	XP_018975893.1	full	-	-	1	GCF_000951615.1	33	NC_031729.1	11068745-11069761	-	
Cyprinus carpio (Cc)	*taar12f*	TAAR4-like	XP_018961602.1	full	-	-	1	GCF_000951615.1	12	NC_031708.1	6966749-6967759	+	
Cyprinus carpio (Cc)	*taar12g*	TAAR4-like	XP_018946046.1	full	-	-	1	WGS_VDB://LHQP01	-	LHQP01033775.1	15629-16645	+	not present in genome assembly
Cyprinus carpio (Cc)	*taar12h*	TAAR4-like	XP_018946046.1	full	-	-	1	GCF_000951615.1	-	NW_017540643.1	74888-75904	+	
Cyprinus carpio (Cc)	*taar12i*	TAAR3-like	XP_018959120.1	full	-	-	1	GCF_000951615.1	-	NW_017545985.1	8783-9934	+	
Cyprinus carpio (Cc)	*taar12j*	TAAR4-like	XP_018946046.1	partial	Ψ	1! 1*	1	WGS_VDB://SAUK01	-	SAUK01000530.1	33619-32874	-	not present in genome assembly
Cyprinus carpio (Cc)	*taar12k*	TAAR4-like	XP_018961608.1	full	-	-	1	GCF_000951615.1	12	NC_031708.1	6978116-6979132	+	
Cyprinus carpio (Cc)	*taar12l*	-	-	full	-	-	1	WGS_VDB://LHQP01	-	LHQP01055690.1	10065-11084	+	not present in genome assembly
Cyprinus carpio (Cc)	*taar12m*	TAAR4-like	XP_018961603.1	full	-	-	1	GCF_000951615.1	12	NC_031708.1	6988431-6989447	+	
Cyprinus carpio (Cc)	*taar12n*	TAAR4-like	XP_018941218.1	full	-	-	1	GCF_000951615.1	-	NW_017539046.1	750-1802	-	
Cyprinus carpio (Cc)	*taar12o*	-	LOC109092292	partial	-	-	1	GCF_000951615.1	12	NC_031708.1	6944320-6944959	+	
Cyprinus carpio (Cc)	*taar13a*	TAAR13c-like	XP_018959962.1	full	-	-	1	GCF_000951615.1	2	NC_031698.1	8856169-8857456	+	
Cyprinus carpio (Cc)	*taar13b*	TAAR13c-like	XP_018959967.1	full	-	-	1	GCF_000951615.1	2	NC_031698.1	8860794-8861813	+	
Cyprinus carpio (Cc)	*taar13c*	TAAR13c	XP_018932792.1	full	-	-	1	GCF_000951615.1	-	NW_017538119.1	334753-335778	-	
Cyprinus carpio (Cc)	*taar13d*	TAAR13c-like	XP_018932791.1	full	-	-	1	GCF_000951615.1	-	NW_017538119.1	332109-333113	-	
Cyprinus carpio (Cc)	*taar13e*	-	-	partial	-	-	1	GCF_000951615.1	7	NC_031703.1	4705416-4706438	+	
Clupea harengus (Ch)	*taar13a*	taar2	XP_012683945.1	full	-	-	1	GCF_000966335.1	-	NW_012218207.1	76993-78006	-	
Dicentrarchus labrax (Dl)	*taar13a*	-	-	full	-	-	1	GCA_000689215.1	-	HG916851.1	95392926-95393909	+	
Dicentrarchus labrax (Dl)	*taar13b*	-	-	full	-	-	1	GCA_000689215.1	-	HG916851.1	57561723-57562754	-	
Esox Lucius (El)	*taar13a*	TAAR13c-like	XP_012993972.2	full	-	-	1	GCF_000721915.3	-	NC_025985.3	16543278-16544387	+	
Gasterosteus aculeatus (Ga)	*taar13a*	-	-	full	Ψ	1!	1	GCA_000180675.1	-	AANH01006890.1	20685-21651	-	
Gadus morhua (Gm)	*taar13a*	-	-	full	-	-	1	GCA_000231765.1	-	HE569769.1	743882-744925	-	
Gadus morhua (Gm)	*taar13b*	-	-	full	-	-	1	GCA_000231765.1	-	HE569769.1	720836-721903	-	
Gadus morhua (Gm)	*taar13c*	-	-	full	-	-	1	GCA_000231765.1	-	HE569769.1	733644-734717	-	
Ictalurus punctatus (Ip)	*taar12a*	TAAR4-like	XP_017341644.1	full	-	-	1	GCF_001660625.1	2	NC_030417.1	36456374..36457420	+	
Ictalurus punctatus (Ip)	*taar12b*	TAAR4-like	XP_017339448.1	full	-	-	1	GCF_001660625.1	2	NC_030417.1	36501767-36502801	+	
Ictalurus punctatus (Ip)	*taar13a*	TAAR13c-like	XP_017341732.1	full	-	-	1	GCF_001660625.1	2	NC_030417.1	36495889-36496932	+	
Ictalurus punctatus (Ip)	*taar13b*	TAAR13c-like	XP_017341719.1	full	-	-	1	GCF_001660625.1	2	NC_030417.1	36491561-36492610	+	
Ictalurus punctatus (Ip)	*taar13c*	TAAR13c-like	XP_017341711.1	full	-	-	1	GCF_001660625.1	2	NC_030417.1	36488929-36489978	+	
Ictalurus punctatus (Ip)	*taar13d*	TAAR13c-like	XP_017341696.1	full	-	-	1	GCF_001660625.1	2	NC_030417.1	36484353-36485387	+	
Ictalurus punctatus (Ip)	*taar13e*	TAAR13c-like	XP_017341685.1	full	-	-	1	GCF_001660625.1	2	NC_030417.1	36480581-36481612	+	
Ictalurus punctatus (Ip)	*taar13f*	TAAR13c-like	XP_017341662.1	full	-	-	1	GCF_001660625.1	2	NC_030417.1	36472284-36473330	+	
Ictalurus punctatus (Ip)	*taar13g*	TAAR13c-like	XP_017341651.1	full	-	-	1	GCF_001660625.1	2	NC_030417.1	36464856-36465905	+	
Ictalurus punctatus (Ip)	*taar13h*	TAAR13c-like	XP_017341672.1	full	-	-	1	GCF_001660625.1	2	NC_030417.1	36475867-36476913	+	
Lates calcarifer (Lac)	*taar13a*	TAAR13c-like	XP_018555677.1	full	-	-	1	GCF_001640805.1	-	NW_017363841.1	2551627-2552655	-	
Lates calcarifer (Lac)	*taar13b*	TAAR13c-like	XP_018555664.1	full	-	-	1	GCF_001640805.1	-	NW_017363841.1	2546203-2547222	-	
Labrus bergylta (Lb)	*taar13a*	TAAR13c-like	XP_020496621.1	full	-	-	1	GCF_900080235.1	-	NW_018114570.1	87924-88955	+	
Labrus bergylta (Lb)	*taar13b*		LOC109989273	full	Ψ	1!	1	GCF_900080235.1	-	NW_018114570.1	25196-26207	-	
Labrus bergylta (Lb)	*taar13c*	TAAR13c-like	XP_020496622.1	full	-	-	1	GCF_900080235.1	-	NW_018114570.1	94469-95485	+	
Labrus bergylta (Lb)	*taar13d*	TAAR13c-like	XP_020496624.1	full	-	-	1	GCF_900080235.1	-	NW_018114570.1	99727-100749	+	
Larimichthys crocea (Lc)	*taar13a*	TAAR13c-like	XP_027138405.1	full	-	-	1	GCF_000972845.2	X	NC_040020.1	19809619-19810638	-	
Larimichthys crocea (Lc)	*taar13b*	TAAR13c-like	XP_019117237.1	full	-	-	1	GCF_000972845.1	-	NW_017608435.1	1-879	-	not in GCF_000972845.2
Larimichthys crocea (Lc)	*taar13c*	TAAR13c-like	XP_019117238.2	full	-	-	1	GCF_000972845.2	X	NC_040020.1	19814894-19815943	-	
Lepisosteus oculatus (Lo)	*taar12a*	-	-	full	-	-	1	GCF_000242695.1	LG1	NC_023179.1	68708840-68709862	+	
Lepisosteus oculatus (Lo)	*taar12b*	-	-	full	-	-	1	GCF_000242695.1	LG1	NC_023179.1	68698466-68699503	+	
Lepisosteus oculatus (Lo)	*taar12c*	-	-	full	-	-	1	GCF_000242695.1	LG1	NC_023179.1	68742836-68743906	+	
Lepisosteus oculatus (Lo)	*taar13a*	-	-	full	-	-	1	GCF_000242695.1	LG1	NC_023179.1	68772650-68773678	+	

Lepisosteus oculatus (Lo)	*taar5-like*	-	-	full	-	-	1	GCF_000242695.1	LG1	NC_023179.1	69059546-69060574	+
Leuciscus waleckii (Lw)	*taar12a*	-	-	full	Ψ	2!	1	GCA_900092035.1	-	FLSR01004880.1	3072502-3073642	-
Leuciscus waleckii (Lw)	*taar13a*	-	-	full	Ψ	1!	1	GCA_900092035.1	-	FLSR01000467.1	1995-2945	-
Leuciscus waleckii (Lw)	*taar13b*	-	-	full	Ψ	1!	1	GCA_900092035.1	-	FLSR01000467.1	10828-11851	-
Morone saxatilis (Ms)	*taar13a*	-	-	partial	-	-	1	GCA_001663605.1	-	JTCL01016091.1	18020-18655	-
Pimephales promelas (Pp)	*taar12a*	-	-	partial	-	-	1	GCA_000700825.1	-	JNCD01017678.1	86739-87671	-
Pimephales promelas (Pp)	*taar12b*	-	-	partial	-	-	1	GCA_000700825.1	-	JNCD01002744.1	122449-123246	-
Pimephales promelas (Pp)	*taar12c*	-	-	full	-	-	1	GCA_000700825.1	-	JNCD01017678.1	29732-30802	-
Pimephales promelas (Pp)	*taar12d*	-	-	full	-	-	1	GCA_000700825.1	-	JNCD01017678.1	89609-90748	-
Pimephales promelas (Pp)	*taar12e*	-	-	full	-	-	1	GCA_000700825.1	-	JNCD01017678.1	21261-22409	-
Pimephales promelas (Pp)	*taar13a*	-	-	full	-	-	1	GCA_000700825.1	-	JNCD01004450.1	57751-58776	-
Pimephales promelas (Pp)	*taar13b*	-	-	full	-	-	1	GCA_000700825.2	-	JNCD01017678.1	6857-7882	+
Pygocentrus nattereri (Pyn)	*taar12a*	TAAR4-like	XP_017562409.1	full	-	-	1	GCF_001682695.1	-	NW_016243879.1	1733682-1734725	+
Pygocentrus nattereri (Pyn)	*taar12b*	TAAR4-like	XP_017562407.1	full	-	-	1	GCF_001682695.1	-	NW_016243879.1	1718560-1719591	+
Pygocentrus nattereri (Pyn)	*taar12c*	TAAR4-like	XP_017562408.1	full	-	-	1	GCF_001682695.1	-	NW_016243879.1	1729237-1730268	+
Pygocentrus nattereri (Pyn)	*taar12d*	TAAR3-like	XP_017562412.1	full	-	-	1	GCF_001682695.1	-	NW_016243879.1	1737731-1738762	+
Pygocentrus nattereri (Pyn)	*taar12e*	TAAR4-like	XP_017562414.1	full	-	-	1	GCF_001682695.1	-	NW_016243879.1	1747895-1748920	+
Pygocentrus nattereri (Pyn)	*taar13a*	TAAR13c-like	XP_017562388.1	full	-	-	1	GCF_001682695.1	-	NW_016243879.1	1758494-1759543	+
Pygocentrus nattereri (Pyn)	*taar13b*	TAAR13c-like	XP_017562390.1	full	-	-	1	GCF_001682695.1	-	NW_016243879.1	1774862-1775911	+
Pygocentrus nattereri (Pyn)	*taar13c*	TAAR13c-like	XP_017562387.1	full	-	-	1	GCF_001682695.1	-	NW_016243879.1	1751534-1752583	+
Pygocentrus nattereri (Pyn)	*taar13d*	TAAR13c-like	XP_017562389.1	full	-	-	1	GCF_001682695.1	-	NW_016243879.1	1766788-1767837	+
Salmo salar (Ss)	*taar13a*	-	-	full	-	-	1	GCF_000233375.1	ssa15	NC_027314.1	30597459-30598328	-
Salmo salar (Ss)	*taar13b*	-	-	full	Ψ	1!	2	GCF_000233375.1	ssa15	NC_027314.1	30597304-30598328	-
Salmo salar (Ss)	*taar13c*	-	-	full	-	-	1	GCF_000233375.1	ssa15	NC_027314.1	30659257-30660219	-
Sebastes aleutianus (Sea)	*taar13a*	-	-	full	-	-	1	GCA_001910805.2	-	LHVS01040848.1	11319-12383	-
Sebastes aleutianus (Sea)	*taar13b*	-	-	partial	-	-	1	GCA_001910805.2	-	LHVS01040851.1	3-641	-
Sebastes aleutianus (Sea)	*taar13c*	-	-	full	Ψ	2!	1	GCA_001910805.2	-	LHVS01040852.1	3-602	-
Sebastes minor (Sem)	*taar13a*	-	-	full	-	-	1	GCA_001910765.2	-	LHCJ01837248.1	2-574	-
Sebastes minor (Sem)	*taar13b*	-	-	partial	-	-	1	GCA_001910765.2	-	LHCJ01837248.1	203..574	-
Sebastes rubrivinctus (Ser)	*taar13a*	-	-	full	-	-	1	GCA_000475215.1	-	AUPQ01075218.1	1668-2732	+
Sebastes rubrivinctus (Ser)	*taar13b*	-	-	partial	-	-	1	GCA_000475215.1	-	AUPQ01038002.1	908-3	-
Sebastes nigrocinctus (Sn)	*taar13a*	-	-	full	-	-	1	GCA_000475235.3	-	AUPR02083636.1	12257-13321	-
Sebastes steindachneri (Ses)	*taar13a*	-	-	partial	-	-	n.d.	GCA_001910785.2	-	LCWK010354496.1; LCW	1-144;1-114;2-256;3-107	+
Scleropages formosus (Sf)	*taar13a*	TAAR13c-like	XP_018599052.1	full	-	-	1	ASM162426v1	-	NW_017371616.1	39141-40175	+
Scleropages formosus (Sf)	*taar13b*	-	LOC108929171	full	Ψ	1!	1	ASM162426v1	-	NW_017371616.1	51463-52507	+
Scleropages formosus (Sf)	*taar13c*	TAAR13c-like	XP_018599050.1	full	-	-	1	ASM162426v1	-	NW_017371616.1	25892-26926	+
Scleropages formosus (Sf)	*taar13d*	-	LOC108929166	full	Ψ	1!	1	ASM162426v1	-	NW_017371616.1	18411-19305	+
Sinocyclocheilus anshuiensis (Sa)	*taar12a*	TAAR4-like	XP_016353209.1	full	-	-	1	GCF_001515605.1	-	NW_015544213.1	2578639-2579658	-
Sinocyclocheilus anshuiensis (Sa)	*taar12b*	TAAR4-like	XP_016353208.1	full	-	-	2	GCF_001515605.1	-	NW_015544213.1	2575897-2576889	-
Sinocyclocheilus anshuiensis (Sa)	*taar12c*	-	LOC107697102	full	Ψ	1!	1	GCF_001515605.1	-	NW_015544213.1	2616124-2617023	-
Sinocyclocheilus anshuiensis (Sa)	*taar12d*	TAAR4-like	XP_016353229.1	full	-	-	1	GCF_001515605.1	-	NW_015544213.1	2611370-2612386	-
Sinocyclocheilus anshuiensis (Sa)	*taar13a*	TAAR13c-like	XP_016353236.1	full	-	-	1	GCF_001515605.1	-	NW_015544213.1	2664594-2665619	+
Sinocyclocheilus anshuiensis (Sa)	*taar13b*	-	LOC107685375	partial	Ψ	1*	1	GCF_001515605.1	-	NW_015539878.1	409227-409793	-
Sinocyclocheilus anshuiensis (Sa)	*taar13c*	TAAR13c	XP_016337433.1	full	-	-	1	GCF_001515605.1	-	NW_015539878.1	412165-413187	-
Sinocyclocheilus anshuiensis (Sa)	*taar13d*	TAAR13c-like	XP_016353237.1	full	-	-	1	GCF_001515605.1	-	NW_015544213.1	2667473-2668498	+
Sinocyclocheilus anshuiensis (Sa)	*taar13e*	TAAR13c-like	XP_016353238.1	full	-	-	1	GCF_001515605.1	-	NW_015544213.1	2670779-2671801	+
Sinocyclocheilus grahami (Sg)	*taar12a*	TAAR4-like	XP_016087126.1	full	-	-	1	GCF_001515645.1	-	NW_015505576.1	896804-897832	+
Sinocyclocheilus grahami (Sg)	*taar12b*	TAAR4-like	XP_016087127.1	full	-	-	1	GCF_001515645.1	-	NW_015505576.1	902334-903362	+
Sinocyclocheilus grahami (Sg)	*taar12c*	TAAR4-like	XP_016087138.1	full	-	-	1	GCF_001515645.1	-	NW_015505576.1	871953-872987	+
Sinocyclocheilus grahami (Sg)	*taar12d*	TAAR4-like	XP_016087141.1	full	-	-	1	GCF_001515645.1	-	NW_015505576.1	874541-875557	+
Sinocyclocheilus grahami (Sg)	*taar12e*	TAAR4-like	XP_016087140.1	full	-	-	1	GCF_001515645.1	-	NW_015505576.1	882475-883491	+
Sinocyclocheilus grahami (Sg)	*taar12f*	TAAR4-like	XP_016087142.1	full	-	-	1	GCF_001515645.1	-	NW_015505576.1	891479-892504	+
Sinocyclocheilus grahami (Sg)	*taar13a*	TAAR13c-like	XP_016087134.1	full	-	-	1	GCF_001515645.1	-	NW_015505576.1	810574-811599	-
Sinocyclocheilus grahami (Sg)	*taar13b*	-	LOC107593282	partial	Ψ	2!1*	1	GCF_001515645.1	-	NW_015505347.1	359338-359967	+
Sinocyclocheilus grahami (Sg)	*taar13c*	TAAR13c	XP_016138370.1	full	-	-	1	GCF_001515645.1	-	NW_015505347.1	354967-355989	+
Sinocyclocheilus grahami (Sg)	*taar13d*	TAAR13c-like	XP_016087133.1	full	-	-	1	GCF_001515645.1	-	NW_015505576.1	805830-806855	-
Sinocyclocheilus grahami (Sg)	*taar13e*	TAAR13c-like	XP_016087132.1	full	-	-	1	GCF_001515645.1	-	NW_015505576.1	800744-801769	-
Sinocyclocheilus rhinocerous (Sr)	*taar12a*	TAAR4-like	XP_016429344.1	full	-	-	1	GCF_001515625.1	-	NW_015642822.1	7441-8463;	+
Sinocyclocheilus rhinocerous (Sr)	*taar12b*	TAAR4-like	XP_016429345.1	full	-	-	1	GCF_001515625.1	-	NW_015642822.1	11768-12790	+
Sinocyclocheilus rhinocerous (Sr)	*taar12c*	TAAR4-like	XP_016400476.1	full	-	-	1	GCF_001515625.1	-	NW_015667042.1	16881-17915	+
Sinocyclocheilus rhinocerous (Sr)	*taar12d*	TAAR4-like	XP_016400475.1	full	Ψ	1*	1	GCF_001515625.1	-	NW_015667042.1	21894-22787	+
Sinocyclocheilus rhinocerous (Sr)	*taar12e*	TAAR4-like	XP_016384356.1	full	-	-	1	GCF_001515625.1	-	NW_015657344.1	740-1759	-
Sinocyclocheilus rhinocerous (Sr)	*taar12f*	TAAR4-like	XP_016402534.1	partial	-	-	1	GCF_001515625.1	-	NW_015769517.1	2-739	-
Sinocyclocheilus rhinocerous (Sr)	*taar12g*	TAAR4-like	XP_016429343.1	full	-	-	1	GCF_001515625.1	-	NW_015642822.1	2050-3213	+
Sinocyclocheilus rhinocerous (Sr)	*taar13a1*	TAAR13c	XP_016382319.1	full	-	-	1	GCF_001515625.1	-	NW_015656152.1	39210-40235	+
Sinocyclocheilus rhinocerous (Sr)	*taar13a2*	-	LOC107742731	partial	Ψ	2*	1	GCF_001515625.1	-	NW_015632707.1	688968-689471	+
Sinocyclocheilus rhinocerous (Sr)	*taar13b*	TAAR13c-like	XP_016382289.1	partial	Ψ	1!1*	1	GCF_001515625.1	-	NW_015656152.1	46150-46764	+
Sinocyclocheilus rhinocerous (Sr)	*taar13c1*	TAAR13c-like	XP_016411245.1	partial	-	-	1	GCF_001515625.1	-	NW_015632707.1	696850-697488	+
Sinocyclocheilus rhinocerous (Sr)	*taar13c2*	TAAR13c-like	XP_016402954.1	partial	-	-	1	GCF_001515625.1	-	NW_015779853.1	605-1060	+
Sinocyclocheilus rhinocerous (Sr)	*taar13d*	TAAR13c-like	XP_016382320.1	full	-	-	1	GCF_001515625.1	-	NW_015656152.1	42268-43293	+
Sinocyclocheilus rhinocerous (Sr)	*taar13e*	TAAR13c-like	XP_016382289.1	full	-	-	1	GCF_001515625.1	-	NW_015656152.1	45877-46764	+

Representative reference TAAR sequences from all classes (class I, II, III) in several bony vertebrate species (mouse, zebrafish, stickleback, and fugu) used for phylogenetic analysis

Amino acid sequences were taken from Hussain et al., PNAS 2009

	Species (Latin name and abbreviation)	Gene name	Synonyms/autom. predictions	Accession No/gene ID/protein ID	chromosomal location
M	Mus musculus (Mm)	taar1	TAAR1	NP_444435.1	10
o	Mus musculus (Mm)	taar2	TAAR2	NP_001007267.1	10
u	Mus musculus (Mm)	taar3	TAAR3	NP_001008429.1	10
s	Mus musculus (Mm)	taar4	TAAR4	NP_001008499.1	10
e	Mus musculus (Mm)	taar5	TAAR5	NP_001009574.1	10
	Mus musculus (Mm)	taar6	TAAR6	NP_001010828.1	10
	Mus musculus (Mm)	taar8	TAAR8a	NP_001010830.1	10
	Mus musculus (Mm)	taar9	TAAR9	NP_001010831.1	10
Z	Danio rerio (Dr)	taar1b	TAAR1	NP_001076373.1	20
e	Danio rerio (Dr)	taar10a	TAAR10a	NP_001076367.1	20
b	Danio rerio (Dr)	taar11	TAAR11	NP_001076546.1	20
r	Danio rerio (Dr)	taar12a	TAAR12a	NP_001076578.1	20
a	Danio rerio (Dr)	taar12b	TAAR12b	NP_001076573.1	20
f	Danio rerio (Dr)	taar12c	TAAR12c	NP_001076564.1	20
I	Danio rerio (Dr)	taar12d	TAAR12c	NP_001076564.1	20
s	Danio rerio (Dr)	taar12e	TAAR12e	NP_001076563.1	20
h	Danio rerio (Dr)	taar12f	TAAR12f	NP_001124143.1	20
	Danio rerio (Dr)	taar12g	TAAR12g	NP_001076377.1	20
	Danio rerio (Dr)	taar12h	TAAR12h	NP_001076378.1	20
	Danio rerio (Dr)	taar12i	TAAR12i	NP_001076554.1	20
	Danio rerio (Dr)	taar12j	TAAR12j	NP_001076380.1	20
	Danio rerio (Dr)	taar12k	TAAR12h	NP_001076378.1	20
	Danio rerio (Dr)	taar12m	TAAR4	XP_001920844.2	15
	Danio rerio (Dr)	taar13a	TAAR64	NP_001076571.1	20
	Danio rerio (Dr)	taar13b	TAAR13b	NP_001076511.1	20
	Danio rerio (Dr)	taar13c	TAAR13c	NP_001076509.1	20
	Danio rerio (Dr)	taar13d	TAAR13d	NP_001076510.1	20
	Danio rerio (Dr)	taar13e	TAAR13e	NP_001076512.1	20
	Danio rerio (Dr)	taar14f	TAAR14f	NP_001076382.1	20
	Danio rerio (Dr)	taar15a	TAAR13c-like	XP_009300776.1	20
	Danio rerio (Dr)	taar16a	TAAR6-like	XP_009303909.1	10
	Danio rerio (Dr)	taar17a	TAAR13c-like	XP_021334896.1	10
	Danio rerio (Dr)	taar18a	TAAR13c-like	XP_021335131.1	10
	Danio rerio (Dr)	taar19a	TAAR13c-like	XP_017213596.1	10
	Danio rerio (Dr)	tar20a	TAAR13c-like	XP_017213576.1	10
S	Gasterosteus aculeatus (Ga)	taar21a	-	VDFJ01000095.1	
ti	Gasterosteus aculeatus (Ga)	taar22a	-	VDFJ01000016.1	
ck	Gasterosteus aculeatus (Ga)	taar23	-	VDFJ01000278.1	
le	Gasterosteus aculeatus (Ga)	taar24	-	VDFJ01000206.1	
ba	Gasterosteus aculeatus (Ga)	taar25a	-	VDFJ01000180.1	
ck	Gasterosteus aculeatus (Ga)	taar26a	-	VDFJ01003622.1	
	Gasterosteus aculeatus (Ga)	taar27	-	VDFJ01000276.1	
F	Takifugu rubripes (Tr)	taar28a	-	GeneID:115247326	
u					
g					
u					

Aminergic receptors used as outgroup in phylogenetic analysis

Species (Latin name and abbreviation)	Gene name	Accession or genomic location (in case of missing accession)	Comments
**Adrenergic receptors**			
Branchiostoma belcheri (Bbe)	*adra1a*	GeneID:109485326	
Callorhinchus milii (Cm)	*adra1a*	GeneID:103191393	
Lethenteron camtschaticum (Lec)	*adra1a*	GCA_000466285.1, contig KE993747.1, range 33178-34107 (+)	
Mus musculus (Mm)	*adra1a*	GeneID:11549	
Mus musculus (Mm)	*adra1b*	GeneID:11548	
Leucoraja erinacea (Le)	*adra1b*	AESE000000000.1, contig AESE012504078.1 range 1321-2283 (+)	
Branchiostoma floridae (Bfl)	*adra2*	Gene ID:7251580	
Ciona intestinalis (Cin)	*adra2*	GeneID:100184338	Annotated as htr1a
Lethenteron camtschaticum (Lec)	*adra2*	GCA_000466285.1, contig KE993680.1, range 3745198-3746598 (+)	
Mus musculus (Mm)	*adra2a*	GeneID:11551	
Rhincodon typus (Rt)	*adra2a*	GeneID:109933335	
Asymmetron lucayanum (Alu)	*adrB*	GCA_001663935.1, contig LZCU01211521.1, range 1805-2977 (+)	
Callorhinchus milii (Cm)	*adrB1*	GeneID:103186583	
Ciona intestinalis (Cin)	*adrB*	GeneID:100177523	
Ciona savignyi (Csa)	*adrB*	GCA_000149265.1, contig CH002630.1, range 98843-100777 (+)	
Lethenteron camtschaticum (Lec)	*adrB*	GCA_000466285.1, contig KE993680.1, range 3147094 to 3148410(+)	
Mus musculus (Mm)	*adrB1*	GeneID:11554	
Rattus norvegicus (Rat)	*adrB*	GeneID:24176	
Rhincodon typus (Rt)	*adrB*	GeneID:109923571	
**Dopaminergic receptors**			
Asymmetron lucayanum (Alu)	*drd1,5*	GCA_001663935.1, contig LZCU01179855.1, range 3013-4119 (+)	
Branchiostoma floridae (Bfl)	*drd1,5*	GCA_000003815.1, contig GG666484.1, range 3478069-3479178 (-)	
Callorhinchus milii (Cm)	*drd1*	GeneID:103187244	
Mus musculus (Mm)	*drd1a*	GeneID:13488	
Petromyzon marinus (Pm)	*drd1*	GCA_002833325.1, contig PIZI01000015.1, range 6404870-6406297 (-)	
Rhincodon typus (Rt)	*drd1*	GeneID:109937715	
Danio rerio (Dr)	*drd2*	GeneID:282557	
Mus musculus (Mm)	*drd2*	GRCm38.p4, contig NC_000075.6, range 49395616-49407091 (+)	
Danio rerio (Dr)	*drd3*	GRCz11, contig NC_007136.7, range 4708538-4732991 (-)	
Mus musculus (Mm)	*drd3*	GeneID:13490	
Branchiostoma floridae (Bfl)	*drd4*	GeneID:7249502	
Danio rerio (Dr)	*drd4a*	GeneID:503564	
Mus musculus (Mm)	*drd4*	GeneID:13491	
Leucoraja erinacea (Le)	*drd5*	WGS_VDB://AESE01, contig AESE011486231.1, range 406-1725 (+)	
Mus musculus (Mm)	*drd5*	GeneID:13492	Synonym drd1b
Rhincodon typus (Rt)	*drd5*	GeneID:109916214	Annotated as drd1-like
**Histaminergic receptors**			
Danio rerio (Dr)	*hrh1*	GeneID:735302	
Mus musculus (Mm)	*hrh1*	GeneID:15465	
Asymmetron lucayanum (Alu)	*hrh2*	GCA_001663935.1, contig LZCU01123976.1, range 103-1209 (-)	
Branchiostoma floridae (Bfl)	*hrh2*	GCA_000003815.1, contig GG666476.1, range 1325027-1326103 (+)	
Danio rerio (Dr)	*hrh2*	GeneID:25461	
Danio rerio (Dr)	*hrh2b*	GeneID:100005590	
Leucoraja erinacea (Le)	*hrh2*	WGS_VDB://AESE01, contig AESE010707911.1, range 742-1827 (-)	
Rattus norvegicus (Rat)	*hrh2*	GeneID:25461	
Rhincodon typus (Rt)	*hrh2*	GeneID:109918887	
Danio rerio (Dr)	*hrh3*	GeneID:561773	
Rhincodon typus (Rt)	*hrh3*	GeneID:109927117	
Danio rerio (Dr)	*hrh4*	Gene ID:100334962	
**Muscarinergic acetylcholine receptors**			
Branchiostoma belcheri (Bbe)	*chrm*	GeneID:109479226	
Ciona intestinalis (Cin)	*chrm1-5*	GeneID:100182166	
Ciona savignyi (Csa)	*chrm1-5*	GCA_000149265.2, contig CH001357.1, range 38054-39553 (-)	
Ciona savignyi (Csa)	*chrm*	GCA_000149265.1, contig CH002155.1, range 846677-855548 (+)	
Mus musculus (Mm)	*chrm1*	ENSMUSG00000032773	
Danio rerio (Dr)	*chrm2a*	GeneID:352938	
Danio rerio (Dr)	*chrm3b*	GeneID:100149598	
Mus musculus (Mm)	*chrm4*	GeneID:12672	
Petromyzon marinus (Pm)	*chrm*	GCA_002833325.1, contig PIZI01000025.1, range 2511456-2513136 (-)	
Rhincodon typus (Rt)	chrm	GeneID:109911563	
**Serotonergic receptors**			
Callorhinchus milii (Cm)	*htr1b*	GeneID:103175228	
Callorhinchus milii (Cm)	*htr1d*	GeneID:103179607	
Danio rerio (Dr)	*htr1a*	GeneID:100001828	
Danio rerio (Dr)	*htr1fb*	GeneID:100004819	
Leucoraja erinacea (Le)	*htr1*	WGS_VDB://AESE01, contig AESE011561756.1, range 155-1153 (+)	
Mus musculus (Mm)	*htr1b*	GeneID:319405	
Petromyzon marinus (Pm)	*htr1a*	GCA_002833325.1, contig PIZI01000013.1, range 14953024-14954282 (+)	
Rhincodon typus (Rt)	*htr1*	GeneID:109924543	
Rhincodon typus (Rt)	*htr1b*	GeneID:109927187	
Ciona savignyi (Csa)	*htr2b*	GCA_000149265.1, contig CH003080.1, range 117045-118924 (-)	
Danio rerio (Dr)	*htr2b*	GeneID:751784	
Rattus norvegicus (Rat)	*htr2a*	GeneID:29595	
Anguilla japonica (Aj)	*htr4-like*	GCA_003597225.1, contig BEWY01000739.1, range 143720-158412 (+)	
Asymmetron lucayanum (Alu)	*htr4*	GCA_001663935.1, contigs LZCU01069842.1, LZCU01138971.1, ranges 2645-4248, 3-464 (+)	
Branchiostoma belcheri (Bbe)	*htr4*	GeneID:109485254	
Branchiostoma floridae (Bfl)	*htr4*	GeneID:118414956	
Branchiostoma lanceolatum (Bla)	*htr4*	GCA_900088365.1, contig FLLO01000036.1, range 925959-928070 (+)	
Callorhinchus milii (Cm)	*htr4*	GeneID:103191306	
Callorhinchus milii (Cm)	*htr4-like*	GeneID:103185614	
Danio rerio (Dr)	*htr4*	Gene ID:101882850, chromosome 14	
Danio rerio (Dr)	*htr4-like*	GeneID:559089	
Gasterosteus aculeatus (Ga)	*htr4*	GCA_000180675.1, contig AANH01006599.1, range 101622-116517 (-)	
Gasterosteus aculeatus (Ga)	*htr4-like*	GCA_000180675.1, contig AANH01004594.1, range 57696-60366 (-)	
Lepisosteus oculatus (Lo)	*htr4*	LepOcu1, LG6, contig NC_023184.1, range 37736330-37712459 (-)	
Lepisosteus oculatus (Lo)	*htr4-like*	LepOcu1, LG2, contig NC_023180.1, range 51326623-51338351 (+)	
Lethenteron camtschaticum (Lec)	*htr4*	GCA_000466285.1, contig KE993680.1, range 1331383-1338029 (-)	
Leucoraja erinacea (Le)	*htr4*	WGS_VDB://AESE01, contig AESE010074862.1;AESE011513946.1 33-236;397-960 (+-)	
Mus musculus (Mm)	*htr4*	GeneID:15562	
Nanorana parkeri (Np)	*htr4*	GeneID:108784291	
Petromyzon marinus (Pm)	*htr4*	XP_032806628.1	
Ptychodera flava (PtFl)	*htr4*	GCA_001465055.1, contig LD352455.1, range 57543-56371 (-)	
Rhincodon typus (Rt)	*htr4*	GeneID:109927282	
Saccoglossus kowalevskii (SaKo)	*htr4*	GeneID:100374682	
Xenopus tropicalis (Xt)	*htr4*	GeneID:100493952, chromosome 3	
Xenopus laevis (Xl)	*htr4.S*	GeneID:496394	
Ciona intestinalis (Cin)	*htr5*	GeneID:104265465	
Ciona savignyi (Csa)	*htr5*	GCA_000149265.1, contig CH003386.1, range 620733-623491 (-)	
Danio rerio (Dr)	*htr5ab*	GeneID:368475	
Lethenteron camtschaticum (Lec)	*htr5*	GCA_000466285.1, contig KE993731.1, range 1661553-1662305 (-)	
Mus musculus (Mm)	*htr5a*	GeneID:15563	
Danio rerio (Dr)	*htr6*	GeneID:568269	
Mus musculus (Mm)	*htr6*	GeneID:15565	
Rhincodon typus (Rt)	*htr6*	GeneID:109917804	
Ciona intestinalis (Cin)	*htr7*	GCF_000224145.3, NC_020173.2, range 2187887-2192745 (-)	
Ciona savignyi (Csa)	*htr7*	GCA_000149265.1, contig CH000164.1, range 44580-52046 (-)	
Danio rerio (Dr)	*htr7*	GeneID:100536080	
Mus musculus (Mm)	*htr7*	GeneID:15566	
**Unclassified aminergic receptors (AmR)**			
Ciona intestinalis (Cin)	*AmR*	GeneID:101242922	
Ciona savignyi (Csa)	*AmR1*	GCA_000149265.1, contig CH002901.1, range 1249970-1251418 (-)	
Ciona savignyi (Csa)	*AmR2*	GCA_000149265.1, contig CH000120.1, range 16409-17671 (-)	

SUPPLEMENTARY INFORMATION
SI Table 2: Intron position in *htr4, taar* and *tarl* genes

Olfactory function in the trace amine-associated receptor family (TAARs) evolved twice independently

Milan Dieris[1], Daniel Kowatschew[1], and Sigrun I. Korsching[1]*

[1]Institute for Genetics, University at Cologne, Germany
*Corresponding author, sigrun.korsching@uni-koeln.de

SI Table 2 MSA shows differentially conserved intron borders in chordate and vertebrate htr4

```
Sako-htr4  MESGITSSTDAIS-----YHSFALDKTSRVYVLEVFTDTGEPIVGAVNDNSTGLSGNVL-
PtFl-htr4  M------STNNIHGLPYVFESSDTPATSAVFVVELETTRQPFLITNDNASTWAVSGGVQP
alu-htr4   M--------------TTIVTMSGYNTTQALGIPGTDLTRTVQPVTNWTNGSLGCTETYIG
bfl-htr4   M--------------TTIVSMSDYNTTQALAVPETHHVRTVQPGANGTNGSSGCTEAYLG
Bla-htr4   M--------------TTIVSMSDFNTTQALVVPETHPLRTAPPVTNHTNGSTGCTESYLG
Pm-htr4    --------------------MYGNNTTF-------------------NNDE---------
Rt-htr4    --------------------MDEADVNE-------------------STSD---GEPHEI
Dr-htr4    MQGCEFPQEDSCHGTGQMPEMEEVDTNE-------------------SN-----------
Mm-htr4    --------------------MDKLDANV-------------------SSNE---------
Dr-tarl1   --------------------ME--NTSE------------------WSNNSLDSDFEACA
Pm-tarl1a  --------------------MDPPSSSV------------AAPSSPASSNSVQGSFSCPG
Rt-taarIId --------------------MN-LSDHE--------NSDNVQYCFEFVNTS------CAR
Dr-taar13c --------------------MD-LSSQE---------YDPSQFCFPAVNNS------CLK


Sako-htr4  -QYGLAERIIIGFVLTVIIILTIAGNILVCASIFIFPSLRTL-TNYFVFSLAIADILVAC
PtFl-htr4  GSFNVAERIVIGFILSLIILTTIVGNILVCTSIIIFPSLRTL-TNYFVFSLAIADVLVAC
alu-htr4   PQYSLTVQVLLSSFLALVILLTIVGNILVISTVALFRSLRSL-TNYFVVSLAVADLLVGV
bfl-htr4   PQYSLEIQVLLSSFLALVILLTIVGNILVISTVALFRSLRSL-TNYFVVSLAVADLLVGV
Bla-htr4   PQYSLVIQILLSCFLALVILLTIVGNILVISTVALFRSLRSL-TNYFVVSLAVADLLVGV
Pm-htr4    -TSSQAEKYILAGIFSIIIAVTILGNLLVVVAVCKDRHLRKIKTNYFIVSLAVADLLVAI
Rt-htr4    PGFSILQKTVLITFLSAIMVMTILGNLLVMVAVCKDRQLRKIKTNYFIVSLAFADLLVSV
Dr-htr4    ---GLAKRVALISFLSLVMLMSVLGNLLVMVAVCKDRQLRKIKTNYFIVSLAFADLLVSV
Mm-htr4    -GFRSVEKVVLLTFLAVVILMAILGNLLVMVAVCRDRQLRKIKTNYFIVSLAFADLLVSV
Dr-tarl1   TLRSFGSRVALYLFIITGILCTVVGNFLVVLAIAYFKQLQCP-TNSFVMSLAVADFLVGL
Pm-tarl1a  SALSSAQRAAWLAMMSVAMASAVLGNLLVVASVLHFRRLQTR-TNAFTASLAVVDLLVGL
Rt-taarIId VTRSIAINVALYIFIITSILITIFGNLVVIISVLHFKQLQTP-TNFLLSSLAVVDFLVGF
Dr-taar13c GTHHVSTQTVVYLILASAMTVTLGNSVVIISIAHFKQLQTP-TNILVMSLALADLLLGL
                                   **  *         *    **    ***  * *

Sako-htr4  LVMTFSTINVVTETWIYGDTFCLIFISFDISFCTASIMHLSCIAFDRYSAI-CNPFQYPL
PtFl-htr4  LVMAISTIYIVTETWMYGDTFCLVFISFDIAFCTASIMHLSCIAYDRYSAI-CNPFQYPI
alu-htr4   IVMTLGTQYEVSGRWRHGEIFCLVFTSMDVSLTTCSIMHLSCIAFDRYYAI-CHPFKYPT
bfl-htr4   IVMTLGTQYEVSGRWRHGEIFCLIFTSMDISLTTCSIMHLSCIAFDRYYAI-CHPFKYPT
Bla-htr4   IVMTLGTQYEVSGRWRHGEIFCLVFTSMDISLTTCSIMHLSCIAFDRYYAI-CHPFKYPT
Pm-htr4    LVMPFGAVERVQNDWPYGDKFCIIRTSFDVMFTTASIMHLCCIALDRFYAICCKPLVYQN
Rt-htr4    MVMPFGAIELIQQNWIYGDRFCLVRTSLDVLLTTASILHLCCISLDRYYAICCQPLVYRN
Dr-htr4    LVMPFGAIELIHQNWIYGETFCLVRTSLDVLLTTASILHLCCISLDRYYAICCQPLVYRN
Mm-htr4    LVMPFGAIELVQDIWAYGEMFCLVRTSLDVLLTTASIFHLCCISLDRYYAICCQPLVYRN
Dr-tarl1   VVMPYSMVRTVEGCWYFGPTFCHLHSSLDVMLCTASIFHLSCIAFDRYYAV-CNPLVYSF
Pm-tarl1a  LVMPFKMTRSAYGCWFYGTAFCAAHTCLDIALCTASILHLACIAFDRHVAV-CDPLRYAQ
Rt-taarIId IVLPYSMVRSVEKCWYFGEVFCKIHSITEIVLTVVSIYTLCFIAIDRYYAM-CDPLFYSV
Dr-taar13c VVMPFSMIRSVDGCWYYGETFCLLHTGFDLFLTSVSIFHLIFIAVDRHQAV-CFPLQYPT
            *            *  *  **             **  *  *  **  *  * *  *

Sako-htr4  KMTSRRVTIMLASCWVVPMFISFIPILLQWNTIGIENVIADVKNF--AGPYSCIFLVNKP
PtFl-htr4  KMTSRRVAVMLAACWVVPMLISFIPILLGWNTLGIENVIAEVKQH--TGPNSCVFLVNRP
alu-htr4   KMTHRKMRVMIAMCWVVPVIIAWVPIMNGWNTLNIAXXXXXXXXX--XXXXXXXXXXXXX
bfl-htr4   KMSHRKMVLMIAMCWVVPVIMAYVPIMNRWNTIGIEPVVEDTKCA--LGPESCVFLVNKE
Bla-htr4   KMSHRKMVLMIAMCWVVPVIMAYVPIMNRWNTIGIEPVVEDTKCE--LGPDSCVFLVNKE
Pm-htr4    KMTPLRVGLMLGACWVVPSLISFIPIANGWNIIGIEQQLELRGQAKEANQTHCIFMVNKV
Rt-htr4    KMTPLRITIMLGGCWIIPSFISFLPIMQGWNIIGIQELVEERKSSNDTNITKCVFMVNKP
Dr-htr4    KMTPLRVTLMIGGCWIIPTVISFLPIMQGWNSIGIKDLIDKRKIS--GNSTVCVFMVNKP
Mm-htr4    KMTPLRIALMLGGCWVLPMFISFLPIMQGWNNIGIVDVIEKRKFSHNSNSTWCVFMVNKP
Dr-tarl1   KMSRKRVGLLIVVCWAIPFLISFGPILFGLHKLGVDIPLPE---------NMCVFLVNRI
Pm-tarl1a  RVTARHVAAMVALSWCCGAVISVVTVSLGWNVVGVPDEVVAA-----SCADSCDFLLGAP
Rt-taarIId KITLPVTVMTIILIWLFAVFYGLSVFLLDFSKKSVDDYVAVR-----SCEGSCI-AYHKF
Dr-taar13c RITIPVAWVMVMISWSMAAFYSYGVVYSKANLEGLEEYIASV-----YCMGGCTLYFNAL
                         *
```

```
Sako-htr4   YAVIASTIAFYMPCILMATAYFFIFRAARKQAQQIKSL-ERATMNWNN-TRNGPGDGH--
PtFl-htr4   YAAVASTIAFYIPCILMASAYLFIFRAARKQAAQIKSL-ERATANWNN-ARNGESGGR--
alu-htr4    XXXXXXXXXXXXXAVLMILAYYQVYRVARRQMQRIISV-EGAAPNNEN------------
bfl-htr4    FSLIASTLSFYLPAVMMTLAYYQVYRAARRQMQQIISL-ERSMNNNEN------------
Bla-htr4    FSLIASTLSFYLPAIMMTLAYYQVYRAARRQMQQIISL-ERAMNNNEN------------
Pm-htr4     YAVACSLVAFYIPLGLMAAAYQRIYVTARAHARQIGSL-QRAVSKDTT-GDEVTMVTSGA
Rt-htr4     FAITCSVVAFYVPLLLMVLAYQRIYVTAREHARQIEML-KRAGTSTDS-GQQ--------
Dr-htr4     YALTCSVVAFYLPLVLMVLAYQRIYVTAREHARQISML-QRAGGAGNA--DS--------
Mm-htr4     YAITCSVVAFYIPFLLMVLAYYRIYVTAKEHAQQIQML-QRAGATSESRPQP--------
Dr-tarl1    YAVMASLVAFYLPMVTMLVAYWKIYKAAKRQAMQISAM-EAQMAAGVG-KDS--------
Pm-tarl1a   YAVGSSVCSFFGPAAFVVVAYARILREARRQGRAIACEQEKQQQQNEDQGRQEDGVGEGG
Rt-taarIId  EGHIDALIVFFIPIFIILGIYVKIFFVTRKRCRKIENMPNDSHCTEEN------------
Dr-taar13c  WSVLDTLLTFFLPCSVMVGLYARIFVVAKKHIKSIT---EANQNENEN------------
                                *             *

Sako-htr4   ---------------PHHHGRRVSLAAEKKAAKTLGIIMGCFTICWCPFFVFNIVDPFCD
PtFl-htr4   --------------------GSVSLAAEKKAAKTLGIIMGCFTVCWCPFFVLNIIDPFCD
alu-htr4    ------------------------TRREKKAAKTLGIIMGCFLVCWMPFFVTNIIDPLCG
bfl-htr4    ------------------------MRREKKAAKTLGIIMGCFLVCWMPFFVANIVEPLCG
Bla-htr4    ------------------------MRREKKAAKTLGIIMGCFLVCWMPFFVANIVEPLCG
Pm-htr4     ASPLRAQQLVPVAQATMVQTQSTRFRHETKAAKTLAIIMGCFCLCWAPFFINNVIDPFVN
Rt-htr4     ----------------ADHSGNHRMKTETKAAKTLCIIMGCFCLCWAPFFITNIVDPFIN
Dr-htr4     ----------------ADHQRNHRMRTETKAAKTLCIIMGCFCLCWAPFFITNVVDPFID
Mm-htr4     ----------------ADQHSTHRMRTETKAAKTLCVIMGCFCFCWAPFFVTNIVDPFID
Dr-tarl1    ---------------SKKQKHRNSIRRERKAAKTLGIIMGVFLLFWLPFFTVNIIDPFIE
Pm-tarl1a   RGGARGEMTAPAGKATQAVGRAADAKSERNATKMLSIVVGIFLASWLPFFLMNVSDPLLG
Rt-taarIId  -------------------NVTIMHKKDQIVVRKQDILIGIFTFSWLPFYVNSILNPYFD
Dr-taar13c  -------------------VFKNPRRSERKAAKTLGIVVGAFILCWLPFFINSLVDPYIN
                                                       * *   * **       *

Sako-htr4   FCIDPG---AWPPVTWLGYINSMLNPLLYAFFNRSFRRAFIRLLRC--------------
PtFl-htr4   FCISPK---VWPPITWLGYTNSMLNPILYAFFNRTFRRAFSRLLRC--------------
alu-htr4    YCIAQSCELCLKFFLWLGYINSTLNPMIYAFFNRSFRRAFKRILSCS-------------
bfl-htr4    YCITRSCDLCLKFFLWLGYINSTLNPMIYAFFNRSFRRAFKRILSCS-------------
Bla-htr4    YCITRSCALCSKFFLWLGYINSTLNPMIYAFFNRSFRRAFKRILSCS-------------
Pm-htr4     YDTPQQ---LWDAWLWLGYANSALNPLLYAFLNRSFRRAFLAILCCWDARYRRSSFSSHS
Rt-htr4     YSVPEQ---LWTACLWLGYINSGFNPFLYAYLNKSFRRAFLRILCCGD-RYRRSSLLGQT
Dr-htr4     YSVPEQ---LWAACLWLGYINSMLNPILYAFLNKSFRRAFLIILCCGHKRYRRPSILGPG
Mm-htr4     YTVPEQ---VWTAFLWLGYINSGLNPFLYAFLNKSFRRAFLIILCCDDERYKRPPILGQT
Dr-tarl1    YGTAVV---IWDVFLWLGYINSSLNPFLYGFFNRSFRRAFLMIMGCR-------------
Pm-tarl1a   YSIDPR---AWEAVTWLGYANSAANPVIYGIFSPNFRAAFRVIAAR--------------
Rt-taarIId  FLIPPV---LDSVFAWFGFFNSTLNPLLYAFLYPWFRKSLKLILSC--------------
Dr-taar13c  FSTPYA---LFDAFGWLGYTNSTLNPIIYGLFYPWFRKTLSLIVTL--------------
                           * *  **  **  *      **

Sako-htr4   -----HVCKGTTAEFDPTISSRER-------------------------
PtFl-htr4   -----HVCKGSAAEFDPTITSHER-RS----------------------
alu-htr4    -----SCCDLDKRDFDPAVSTALQGRTRSRRRPNGDVVN--------NS
bfl-htr4    -----SCCDLDNRDFDPAVSTALQRRTSTRHQ-NGEAVN--------NT
Bla-htr4    -----SCCDVDNRDFDPAVSTALQGRSSRRHQ-NGEVVN--------NT
Pm-htr4     VRYS-VSTNGSAVQPVPS--ARVRARARLPSRPRTRAAAPAVSKAPSPV
Rt-htr4     IPCSATTINGSAHVLR---------------------------------
Dr-htr4     TTCTATQINGSTHVLKYAVLH----NGNHNEQEKLSIQNDTESQESCF-
Mm-htr4     VPCSTTTINGSTHVLRDTVEC----GGQWESRCHLTATSPLVAAQPSDT
Dr-tarl1    -----ICLHGSAQGMDLSHSKRDG---------NERTDN----------
Pm-tarl1a   -----SAFRAGSRDVQLG--F----------------------------
Rt-taarIId  -----QIFNTDPSTVNFLSE-----------------------------
Dr-taar13c  -----RIFEPNSSDINLFTV-----------------------------
```

SI Table 2 Multiple sequence alignment (MSA) of 9 HTR4, 2 TARL and 2 TAAR sequences.

Sequences are given by species abbreviation and gene name, for full species names and further details see SI Table 1. Introns were predicted with Genewise 2 (Birney et al., 2004). Note the differential conservation of intron borders in **lancelet** (alu, bfl, bla) vs. **vertebrate** (Pm, Rt, Dr, Mm) *htr4*. The MSA was generated using MAFFT v7.471, E-INS-i setting, (Katoh and Standley, 2013), and sequence positions with over 85% gaps were removed using Gapstreeze (http://hcv.lanl.gov/content/se- quence/GAPSTREEZE/gap.html).

References

Birney E., Clamp, M. & Durbin, R. (2004) GeneWise and Genomewise. *Genome Research* **14**, 988–995.

Katoh K. & Standley D.M. (2013) MAFFT Multiple Sequence Alignment Software Version 7: Improvements in Performance and Usability. *Molecular Biology and Evolution* **30**, 772–780.
